# Assembly of CF_3_‑Pyrazole–Triazole Hybrids through (3 + 3)-Cycloaddition/Ring Contraction and Click Chemistry

**DOI:** 10.1021/acs.joc.5c03120

**Published:** 2026-02-16

**Authors:** Kamil Świątek, Greta Utecht-Jarzyńska, Barbara Olszewska, Katarzyna Gach-Janczak, Marcin Jasiński

**Affiliations:** ‡ Department of Organic and Applied Chemistry, Faculty of Chemistry, 49602 University of Lodz, Tamka 12, 91403 Łódź, Poland; § Department of Biomolecular Chemistry, Medical University of Lodz, Mazowiecka 6/8, 92215 Łódź, Poland

## Abstract

A concise three-step approach for the construction of trifluoromethylated pyrazole–1,2,3-triazole hybrids, considered a promising platform for the discovery of new pharmaceuticals and agrochemicals, is described. The sequence involves (*i*) (3 + 3)-cycloaddition of *in situ*-generated nitrile imines with mercaptoacetaldehyde followed by spontaneous ring contraction, (*ii*) a C(5)-selective azide transfer, and (*iii*) a copper-catalyzed Huisgen–Meldal–Sharpless click cycloaddition. The devised protocol enables the preparation of a broad array of target structures, including drug-inspired analogues and chiral representatives, obtained in moderate to high overall yields. Subsequent functional group interconversions on the hybrids provide access to synthetically valuable motifs such as aldehydes, amines, carboxylic acids, esters, and amide. The structure of a representative product was confirmed by X-ray analysis.

## Graphical abstract

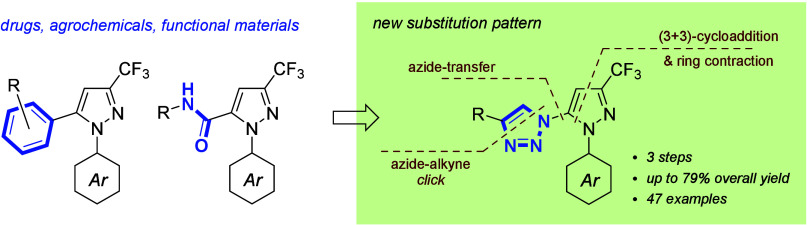

## Introduction

Fluorinated and fluoroalkylated pyrazoles have attracted remarkable attention as privileged scaffolds in the design of pharmaceutical agents, crop protection chemicals, and advanced functional materials.^1^ In this context, the 1-aryl-3-trifluoromethylpyrazole core represents a particularly appealing structural motif for the discovery of new bioactive compounds (Figure 1).^2^ Introduction of a (hetero)­aryl substituent at C(5) has provided access to a broad spectrum of medicinally relevant molecules, including anti-inflammatory agents (e.g., celecoxib),^2a^ anticancer candidates (SC-560),^2b^ antibacterial^2c^ and antifungal compounds,^2d^ and veterinary medicines (mavacoxib).^2e^ Derivatives bearing a furan2-yl unit (e.g., MPA14) exhibit pronounced COX-1 inhibitory activity,^2f^ whereas analogues incorporating pyridine or thiophene rings have been applied in the treatment of asthma.^2g^ In addition, more complex architectures featuring an additional azole ring, such as 1,3,4-oxadiazole or pyrazole, located at C(5) have been reported as promising coactivator-associated arginine methyltransferase 1 (CARM1) antagonists,^2h^ and ATP-binding cassette transporting modulators,^2i^ respectively.

**Figure 1:**
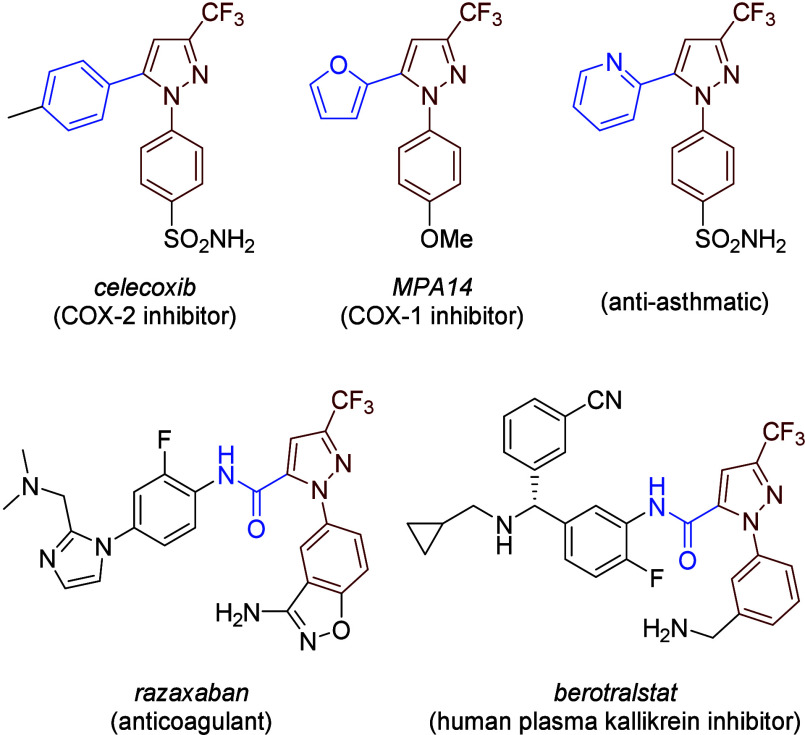
Selected bioactive 1-aryl-3-CF_3_-pyrazoles.

On the other hand, installation of an amide functionality at C(5) of the 1-aryl-3-CF_3_-pyrazole framework has furnished a diverse series of compounds with considerable potential in both agrochemical and medicinal chemistry.^3^ Accordingly, materials featuring herbicidal,^3a^ parasiticidal,^3b^ insecticidal,^3c^ and nematicidal^3d^ as well as antiproliferative^3e^ and CARM1 inhibitory^3f^ properties have been described. Among these, particular attention should be drawn to two marketed drugs: razaxaban,^3g^ an orally active inhibitor of coagulation factor Xa, and berotralstat,^3h^ a human plasma kallikrein inhibitor employed for the prophylactic treatment of hereditary angioedema (HAE).

Prompted by the well-documented biological activity of both aforementioned subclasses of 1-aryl-3-trifluoromethylpyrazoles, and taking into account the established significance of the 1,2,3-triazole motif in medicinal chemistry, frequently employed as bioisosteric replacement for amide (peptide) and related groups,^4^ we envisaged that the structurally related pyrazole–triazole hybrids of type **1** might represent a valuable platform for the discovery of new bioactive molecules (Scheme 1).

**Scheme 1:**
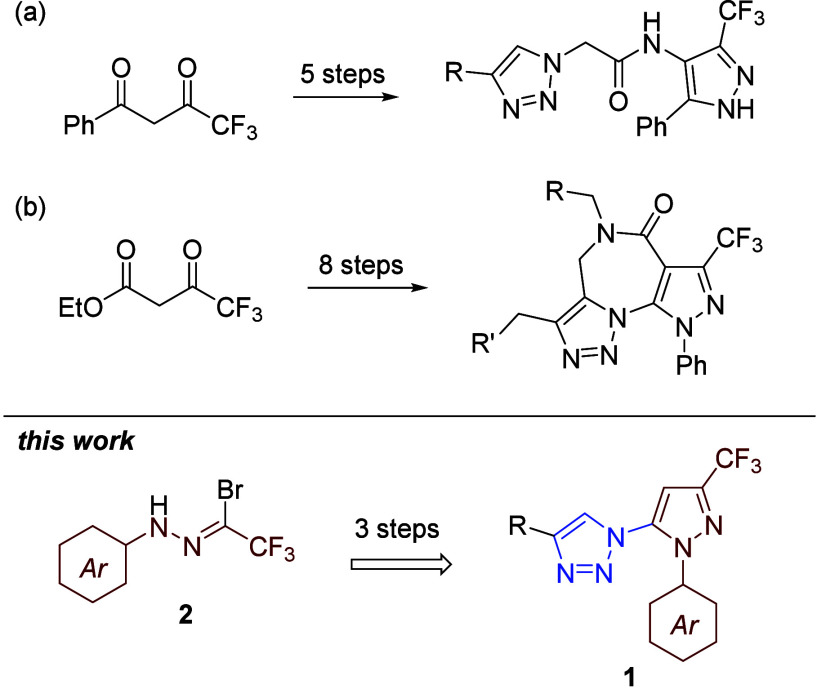
Known Trifluoromethylated Pyrazole–[1,2,3]­Triazoles and the Hybrids **1** Reported Herein

A variety of non-fluorinated pyrazole-based compounds bearing either a 1,2,3- or 1,2,4-triazole ring have been extensively investigated in recent years.^5^ The reported promising activities encompass anticonvulsant,^5a^ antimicrobial,^5b^ antifungal,^5c^ anticancer,^5d,5e^ and tyrosinase inhibitory properties.^5f^ An elegant approach to cyclooxygenase inhibitors obtained via *in situ* click chemistry was demonstrated by Wuest,^5g^ in which the active site of the COX-2 isozyme served as a reaction vessel, enabling the identification of two highly potent and selective products. Because of their high nitrogen content, several pyrazole–triazole hybrids functionalized with amino, azo and/or nitro groups have also been described as thermally stable energetic materials of potential relevance to military, mining, and aerospace applications.^6^

In contrast, the corresponding fluoromethylated structural motifs remain only sparsely explored.^7^ In 2015, Atmakur et al. reported the synthesis of a library of CF_3_-functionalized pyrazoles linked to a 1,2,3-triazole ring through an acetamide-derived spacer (Scheme 1).^7a^ The designed products were obtained via a five-step sequence using 4,4,4-trifluoro-1-phenylbutane-1,3-dione as the key fluorinated substrate. Preliminary biological evaluation revealed several promising lead compounds exhibiting an *in vitro* antimycobacterial activity. Shortly thereafter, the same group disclosed a multistep synthesis of cytotoxic tricyclic diazepine derivatives comprising directly bonded pyrazole and 1,2,3-triazole units, starting with 4,4,4-trifluoro-3-oxobutanoate (Scheme 1).^7b^ This ester was also employed by the Rajashakar group in the synthesis of fully substituted 5-(1,2,3-triazol-1-yl)-3-CF_3_-pyrazole-4-carbaldehyde derivatives.^7c^

However, a general protocol for the synthesis of trifluoromethylated pyrazole–triazole hybrids of type **1**, closely resembling the well-established 5-(hetero)­aryl- and 5-aminocarbonyl-functionalized pyrazoles of practical significance, has not been described. Moreover, CF_3_-functionalized hydrazonoyl bromides **2** have emerged as a convenient alternative to the classical 1,3-dicarbonyl substrates typically employed in pyrazole synthesis.^8^ Thus, in continuation of our study aimed at development of useful synthetic methodologies for fluorinated azoles,^9^ here we report a rapid route to pyrazoles **1** via a three-step sequence comprising the formation of the 1-aryl-3-CF_3_-pyrazole unit, its selective azidation, and a copper-catalyzed azide–alkyne cycloaddition.

## Results and Discussion

We commenced our study with 1-aryl-3-trifluoromethylpyrazoles **3**, prepared according to the protocol recently developed in our laboratory.^9a^ The method relies on a one-pot (3 + 3)-cycloaddition of mercaptoacetaldehyde with the nitrile imine generated *in situ* via base-mediated dehydrohalogenation of the corresponding bromides **2**, followed by a spontaneous Eschenmoser-type ring contraction of the first formed 1,3,4-thiadiazine intermediate (Scheme 2). Thus, starting from 1,4-dithiane-2,5-diol (**4**) (the dimer of mercaptoacetaldehyde) and nitrile imine precursors **2a**–**2g**, we obtained a series of eight model pyrazoles **3a**–**3g** bearing selected *para* substituents, i.e., X = Me (96%), ^*i*^Pr (85%), OMe (92%), OBn (97%), Cl (86%), CF_3_ (70%), and CN (71%). All products were isolated in high yield.

**Scheme 2:**
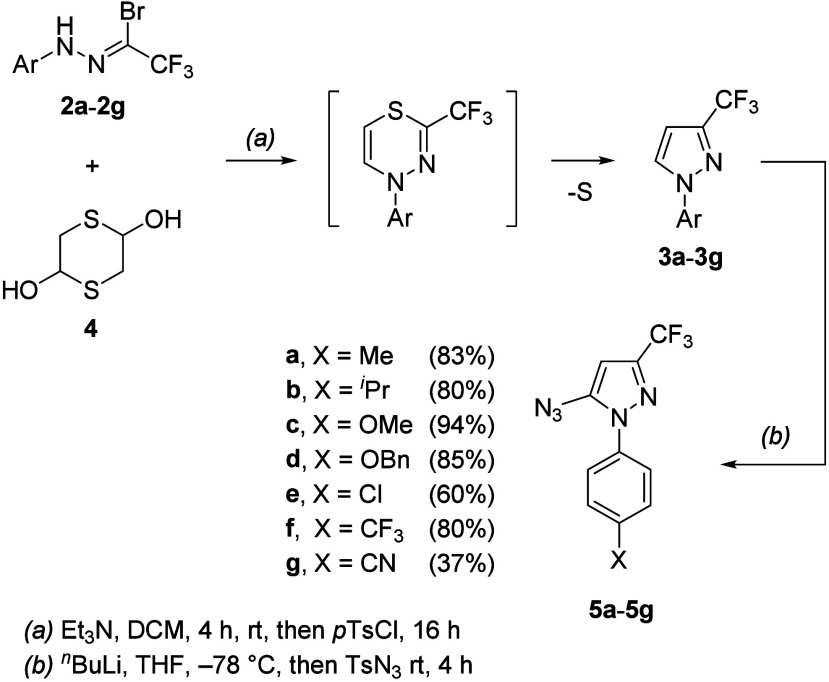
Synthesis of 5-Azido-3-CF_3_-pyrazoles **5a**–**5g**

In the synthesis of the key azides **5**, we benefited from the pronounced acidity of the C(5)–*H* in pyrazoles **3**.^9b^ Selective deprotonation with a slight excess of *n*-BuLi at −78 °C furnished the corresponding lithium 5-pyrazolides, which upon treatment with *p*-toluenesulfonyl azide as an N_3_-transferring reagent underwent smooth functionalization to afford the desired series of 5-azidopyrazoles **5a**–**5g**. No significant influence of electronic effects on the reaction efficiency was observed; pyrazoles with aryl residues bearing strongly electron-donating (**5c**; X = OMe) and strongly electron-withdrawing (**5f**; X = CF_3_) substituents delivered the expected azides in 94% and 80% yield, respectively. The chloro- and cyano-substituted substrates also provided the desired azides **5e** (60%) and **5g** (37%) in somewhat lower yields, presumably due to side reactions with *n*-BuLi (i.e., halogen–lithium exchange and nucleophilic addition, respectively). In the latter case, the formation of an unidentified byproduct was noted; despite repeated attempts, it could not be removed by standard chromatography or recrystallization due to limited stability of **5g**, and the material (ca. 90% purity) was therefore used in the next step without further purification. The reaction proved to be readily scalable, delivering **5c** (X = OMe) in a comparable 90% yield when performed on a gram scale (1.9 g, 6.6 mmol).

For the initial click experiments, we employed azide **5a** and phenylacetylene (Scheme 3). A brief screening of literature-reported conditions,^7a,10^ during which the solvent (DCM, MeCN, aqueous MeOH) and the copper source (CuSO_4_, (AcO)_2_Cu, CuI/DIPEA) were evaluated, allowed us to identify optimal reaction parameters. Thus, running the reaction with a slight excess of the alkyne and a CuSO_4_/ascorbate catalytic system, in a MeOH/H_2_O (10:1 mixture) at 55 °C, ensured complete consumption of the starting azide. The analytically pure sample of the expected (3 + 2)-cycloadduct **1aa** was isolated in high 85% yield after simple filtration of the crude product through a short plug of silica gel, followed by recrystallization from hexanes. With these conditions in hand, a first set of phenylacetylene-derived products **1aa**–**1ag** were obtained in good yields (67–86%), regardless of the electronic nature of the substituents or functional groups present in the starting azides.

Next, we examined the scope with respect to the alkyne reaction partners using 4-methoxyphenyl-substituted azide **5c** (Scheme 3). A series of *para*-substituted phenylacetylenes was checked, affording the corresponding 1,2,3-triazoles bearing primary amino (**1ba**), alkyl (**1bc**-**1bd**, Me, and n-Pent), halogen (**1be**, Cl), or cyano (**1bf**) groups. Introduction of an electron-rich OMe donor at the *para*, *meta*, or *ortho* position of the aromatic ring exerted only a marginal influence on the reaction efficiency, with the *ortho*-substituted product **1bg** isolated in 91% yield and an excellent 79% overall yield (for three steps). The structure of *para*-functionalized isomer **1bb** was unambiguously confirmed by X-ray diffraction (CCDC 2425718).

Both increased substitution, as in **1bi** (2,4,6-trimethyl), and extension of the π system, as in naphthyl derivative **1bj**, were well-tolerated. Electron-deficient fluorinated phenylacetylenes bearing one or two substituents, i.e., fluorine atoms or fluoroalkyl groups (CF_3_, OCF_3_), did not hamper the reaction, consistently providing products **1bk**–**1bo** in yields exceeding 80%. In the case of 1,4-diethynylbenzene, the applied conditions furnished a mixture of two products, **1bt** (53%) and **1bu** (15%). Fine-tuning of the reactant stoichiometry provided excellent control over the reaction outcome, enabling the selective formation of the corresponding monocycloadducts (using 1.6 equiv of alkyne) and biscycloadducts (using 2.5 equiv of azide), which were isolated in 83% and 56%, respectively. Incorporation of pyridyl and thienyl units, as exemplary six- and five-membered heteroaryl substituents, furnished **1br** and **1bs** in 73% and 87% yield, respectively. Moreover, reaction of an acetylene bearing ferrocene moiety, selected as a representative organometallic counterpart, also proceeded smoothly to afford the expected cycloadduct (**1bq**, 66%).

Aliphatic alkynes were checked as well; the (3 + 2)-cycloaddition reactions of 1-octyne and *tert*-butylacetylene delivered the expected products **1ca** and **1cb**, although in the latter case, the sterically crowded product was obtained in diminished yield. Trimethylsilylacetylene was used as a surrogate for acetylene; due to spontaneous cleavage of the C–Si bond during the aqueous workup, the desired hybrid **1cc**, lacking a substituent at C(4) of the 1,2,3-triazole ring, was isolated. Introduction of functional groups such as hydroxymethyl, diethoxymethyl, and methoxycarbonyl was readily accomplished by employing the respective acetylenes, although product **1ce** bearing a masked carbonyl group was accessed in a markedly lower yield (35%) due to partial hydrolysis of the acetal moiety. In addition, two multifunctional building blocks, BMK-alkyne and hydroxyl-diazirine-alkyne, often used as probes for selective modification of biological targets, were checked in reactions with model azide **5c** under the optimal reaction conditions (CuSO_4_/sodium ascorbate, MeOH/H_2_O, 55 °C, 5 h). Accordingly, new derivatives **1cg** (41%) and **1ch** (81%) suitable for potential ^19^F labeling of biomolecules were obtained.^11^

To further assess the synthetic potential of 5-azido-CF_3_-pyrazole **5c** in the modification of more complex systems, a series of well-known pharmaceuticals bearing terminal alkyne functionality was involved in the study (Scheme 3). Two monoamine oxidase inhibitors, pargyline and rasagiline,^12a^ featuring secondary and tertiary amine groups, respectively, provided the expected cycloadducts **1da** (68%) and **1dc** (77%). Notably, the optical purity of the latter substrate remained unchanged during the (3 + 2)-cycloaddition step, leading to an enantiopure pyrazole–triazole hybrid. Moreover, erlotinib,^12b^ a tyrosine kinase inhibitor used as first-line therapy for advanced nonsmall-cell lung cancer, as well as gestrinone,^12c^ a synthetic steroid hormone approved for the treatment of endometriosis, provided the expected cycloadducts in excellent yields of 84% (**1db**) and 87% (**1dd**).

The presence of hydroxyl and cyano functionalities in the pyrazole–triazole hybrids **1bp** and **1bf**, respectively, offers several opportunities for postcyclizative modifications of the heterocyclic framework (Scheme 4). For example, straightforward acylation of the hydroxy group in **1bp** with Ac_2_O afforded ester **1bv** in 91% yield, whereas oxidation with the Corey–Suggs reagent furnished the corresponding aldehyde **1bw** almost quantitatively. The methoxycarbonyl moiety in **1bx** was introduced efficiently by permanganate-mediated oxidation in wet MeCN, followed by Fischer esterification, giving the product in an overall 93% yield.

**Scheme 3:**
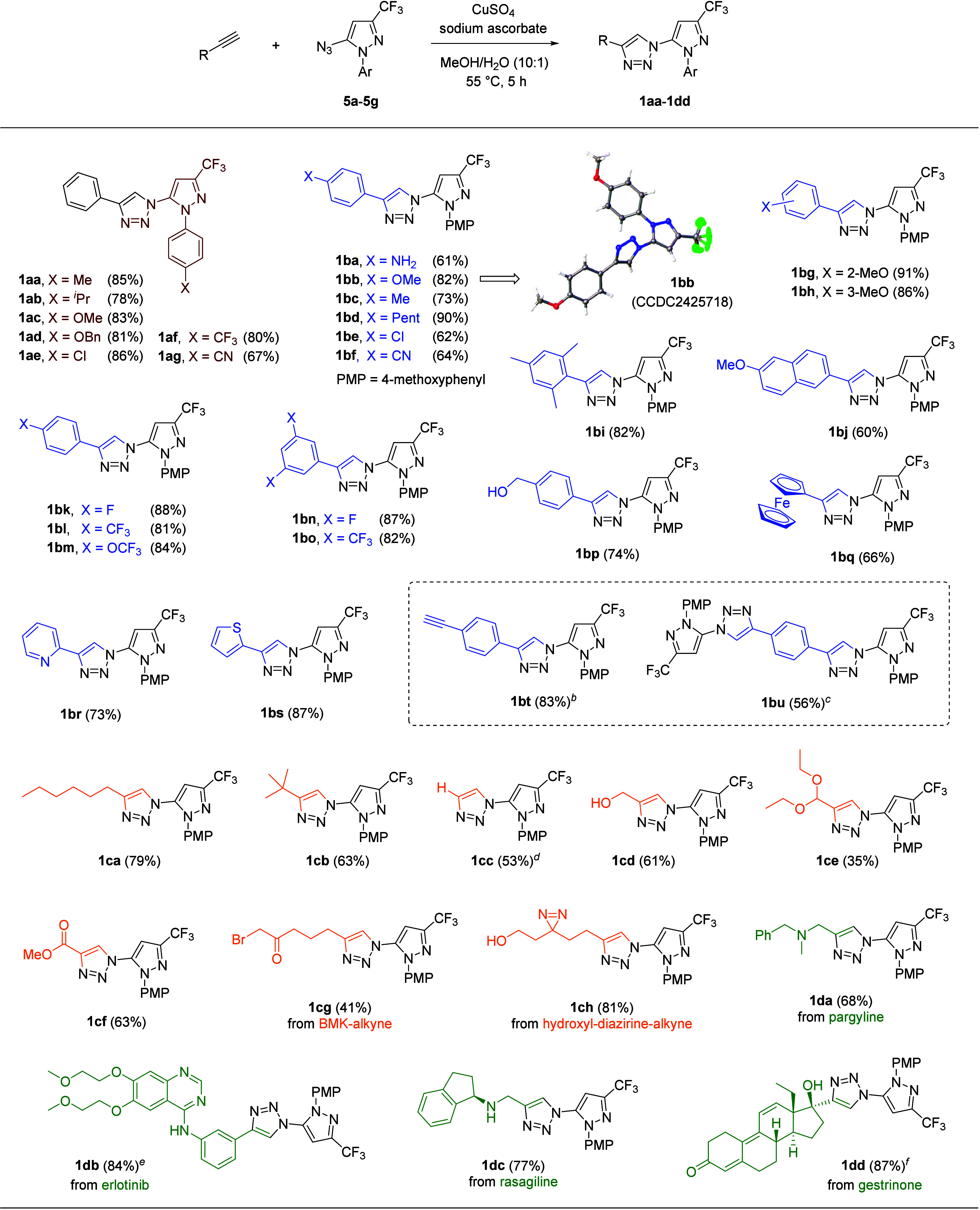
Synthesis of Pyrazole–Triazole Hybrids **1aa**–**1dd** via Click Azide–Alkyne (3 + 2)-Cycloaddition: Scope of Substrates^a^

a Yields refer to analytically pure samples obtained by chromatography, followed by recrystallization.
b Using 1.6 equiv of 1,4-diethynylbenzene.
c Using 2.5 equiv of azide **5c**.
d TMS-acetylene was used as a reaction partner.
e 1.5 equiv of azide **5c**, reaction time of 40 h.
f 1.5 equiv of azide **5c**, reaction time of 16 h.

For the transformation of the cyano group into the primary amide, we employed classical conditions of the Radziszewski-type reaction,^13^ relying on H_2_O_2_-induced hydrolysis under basic conditions. Thus, treatment of **1bf** with an excess of hydrogen peroxide in the presence of Na_2_CO_3_ at room temperature furnished amide **1by** in an excellent yield of 94%.

Exhaustive reduction of the cyano group, proceeding *via* reductive deamination of the initially formed amine,^14^ was observed upon catalytic hydrogenation at elevated pressure (70 psi) using palladium on charcoal as the catalyst. The corresponding product bearing the *p*-tolyl substituent was obtained quantitatively, and its identity with the original sample **1bc**, prepared independently through azide–alkyne (3 + 2)-cycloaddition reaction (Scheme 3) was confirmed by NMR analysis. The desired amine was accessible under milder hydrogenation conditions (H_2_, slight positive pressure from balloon) employing Raney-Ni as the catalyst; the first formed product was subsequently acylated with isobutyryl chloride to furnish the corresponding amide **1bz** in a fair overall yield of 74%. These experiments clearly demonstrate the remarkable stability of the designed 1-aryl-5-(1,2,3-triazol-1-yl)-3-CF_3_-pyrazole core under both harsh reductive and oxidative conditions and establish these pyrazole hybrids as robust building blocks for construction of more elaborate molecular architectures.

**Scheme 4:**
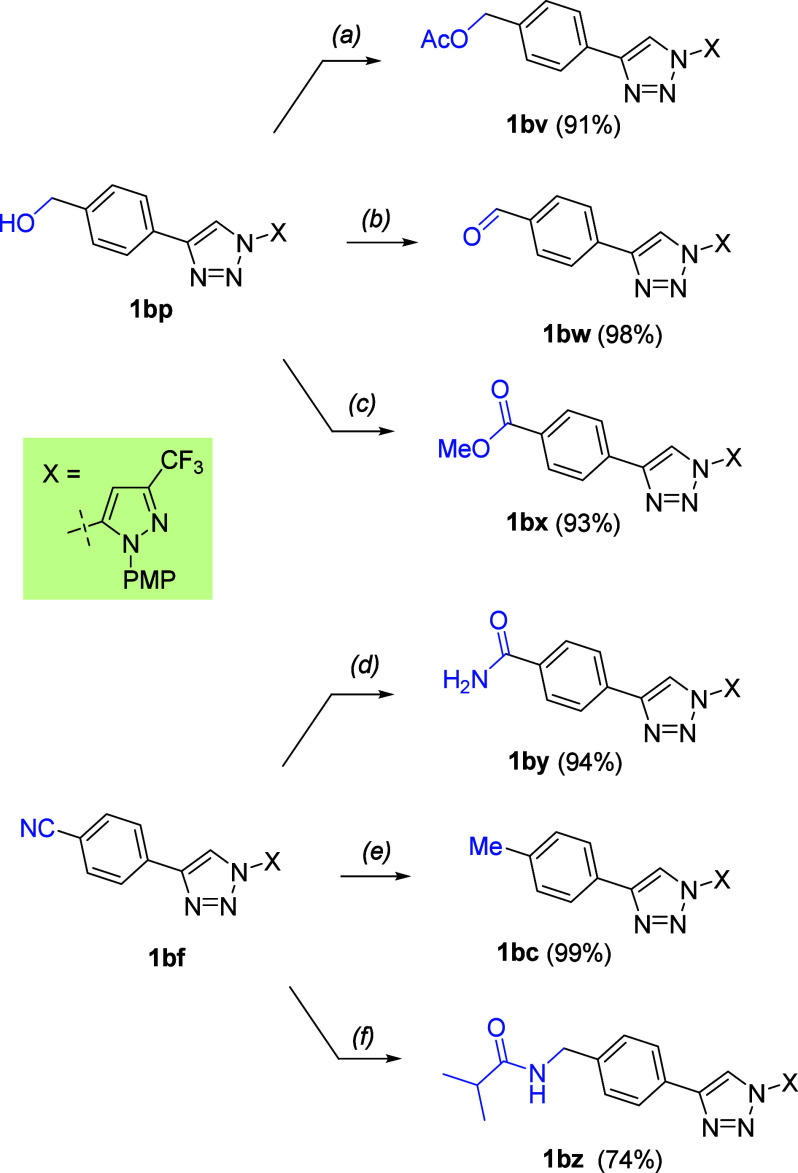
Functional Group Transformations in **1bp** and **1bf**

a Ac_2_O, DCM, 40 °C, 90 min.
b PCC, DCM, rt, 3 h.
c KMnO_4_, MeCN, 80 °C, 2 h, then MeOH, H_2_SO_4_, reflux 12 h.
d H_2_O_2_, Na_2_CO_3_, acetone rt, 2 h.
e H_2_ (70 psi), Pd/C, THF, rt, 5 h.
f H_2_, Raney-Ni, NH_3_aq, rt, 2 h, then i-PrCOCl, Et_3_N, THF, rt, 1 h.

Finally, to check whether the isomeric hybrids bearing the 1,2,3-triazole moiety at C(4) of the pyrazole core could be accessed, a plausible C(4)-iodination/lithiation/azide-transfer sequence was investigated. As depicted in Scheme 5, the representative substrate **3a** was converted in fully regioselective fashion into the corresponding iodide **7** using a slight excess of elemental iodine and ceric ammonium nitrate (CAN) as a mild oxidant.^9b^ To our delight, the subsequent iodine–lithium exchange in **7** (X = I) proceeded smoothly, and after N_3_ transfer the expected azide **8** (X = N_3_) was obtained, albeit in a moderate yield of 42%. Cu-catalyzed (3 + 2)-cycloaddition of **8** with phenylacetylene, furnishing the 4-(1,2,3-triazol-1-yl)­pyrazole derivative **9** (68%), thus demonstrated the feasibility of the designed approach.

**Scheme 5:**
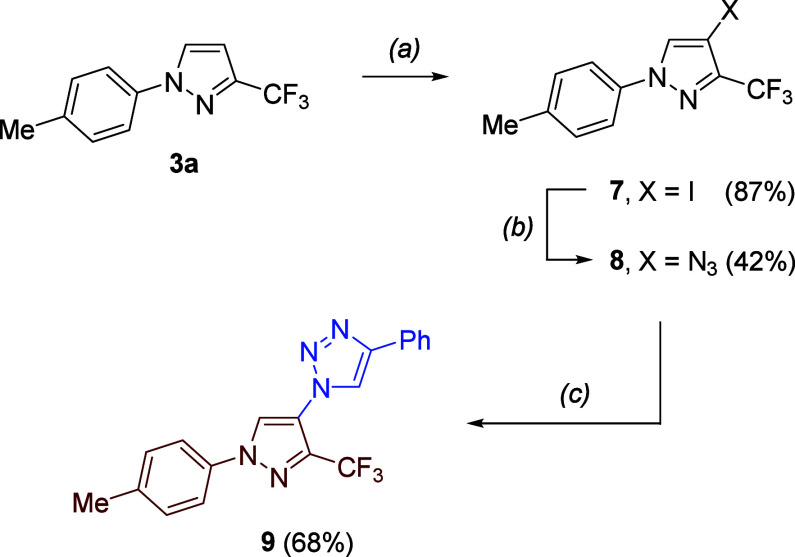
Access to Isomeric Pyrazole–Triazole Hybrid **9**

a I_2_ (1.3 equiv), CAN (1.1 equiv), MeCN, reflux, 16 h.
b
^*n*^BuLi, THF, −78 °C, then TsN_3_ rt, 4 h.
c Ph−C≡CH, CuSO_4_, sodium ascorbate, MeOH/H_2_O (10:1), 55 °C, 5 h.

## Conclusions

In summary, a method for the rapid assembly of trifluoromethylated pyrazole–triazole hybrids inspired by the structural motifs of numerous biologically relevant 3-CF_3_-pyrazole derivatives of practical significance is presented. By employing a three-step sequence comprising a (3 + 3)-cycloaddition/Eschenmoser-type ring contraction cascade affording 1-arylpyrazoles, followed by C(5)-selective azidation and Cu-catalyzed Huisgen cycloaddition, a broad set of variously functionalized hybrids was obtained in high overall yields. Sterically demanding substrates, including the synthetic steroidal drug gestrinone, as well as more challenging alkynes bearing additional (photo)­labile functionalities or strongly electron-deficient and -rich substituents, were generally well tolerated. Moreover, selected functional group interconversions performed under harsh reductive or oxidative conditions demonstrated the remarkable stability of the bis-heterocyclic core and highlighted its robustness. Thus, the developed protocol can be recommended for the preparation of more advanced analogues. Biological evaluation of selected hybrids is currently underway in our laboratories and will be reported in a separate study.

## Experimental Section

### General

All commercially available reagents and solvents were used as received. Products were purified by filtration through a short plug of silica gel (FCC) or standard column chromatography (CC) (SiO_2_, 230–400 mesh) by using freshly distilled solvents and recrystallized from appropriate solvents. NMR spectra were taken on a Bruker AVIII instrument (^1^H at 600 MHz, ^13^C at 151 MHz, and ^19^F NMR at 565 MHz); chemical shifts are reported relative to the solvent residual peaks [for CDCl_3_: ^1^H NMR: δ = 7.26, ^13^C NMR: δ = 77.16; for DMSO-*d*_6_: ^1^H NMR: δ = 2.50, ^13^C NMR: δ = 39.52] or to CFCl_3_ (δ = 0.00) used as an external standard. The IR spectra were taken with an Agilent Cary 630 FTIR spectrometer, in neat. (ESI)-MS was performed with a Varian 500-MS LC ion trap; high-resolution MS (ESI-TOF) measurements were performed with a Waters Synapt G2-Si mass spectrometer. Combustion analyses were obtained with a Vario EL III (Elementar Analysensysteme GmbH) instrument. Optical rotations were determined with a PerkinElmer 241 polarimeter at the temperatures indicated. Melting points were determined in capillaries with a MEL-TEMP apparatus (Laboratory Devices) or with a polarizing optical microscope (POM) (Opta-Tech) and are uncorrected. Single crystals of **1bb** were measured on a XtaLAB Synergy, Dualflex, Pilatus 300 K diffractometer using mirror-focused Cu Kα radiation. Crystallographic data have been deposited at the Cambridge Crystallographic Data Center as supplementary publication number CCDC 2425718. These data can be obtained free of charge from the CCDC, 12 Union Road, Cambridge CB2 1EZ, U.K.; fax: + 44 (0) 1223 336 033; email: deposit@ccdc.cam.ac.uk (or via http://www.ccdc.cam.ac.uk/conts/retrieving.html).

#### General Procedure for Synthesis of Pyrazole–Triazole Hybrids **1**

A mixture of 5-azidopyrazole **5** (1.00 mmol), acetylene (1.20 mmol), copper­(II) sulfate pentahydrate (38 mg, 0.15 mmol), and sodium l-ascorbate (59.5 mg, 0.30 mmol) in MeOH/H_2_O (10:1, 14 mL) was stirred at 55 °C (oil bath) until the starting pyrazole was fully consumed (typically up to 5 h; TLC monitoring). The solvents were then evaporated, and the crude reaction mixture was dissolved in DCM (20 mL), dried over Na_2_SO_4_, and filtered through a Celite pad, which was washed with additional portions of DCM (2 × 8 mL). After the solvent was removed *in vacuo*, product **1** was purified by flash column chromatography (FCC) and recrystallized.

#### 4-Phenyl-1-(1-(*p*-tolyl)-3-(trifluoromethyl)-1*H*-pyrazol-5-yl)-1*H*-1,2,3-triazole (**1aa**)

FCC (SiO_2_, hexane/EtOAc 4:1); colorless solid, 314 mg (85%); mp 120–122 °C (hexane). ^1^H NMR (600 MHz, CDCl_3_) δ: 7.80–7.79 (m, 2H), 7.75 (s, 1H), 7.45–7.43 (m, 2H), 7.39–7.36 (m, 1H), 7.20–7.17 (m, 4H), 7.00 (s, 1H), 2.35 (s, 3H). ^13^C­{^1^H} NMR (151 MHz, CDCl_3_) δ 148.5, 143.0 (q, ^2^ *J*_C–F_ = 39.6 Hz), 140.2, 135.8, 134.5, 130.3, 129.4, 129.2, 129.1, 126.1, 124.3, 121.6, 120.7 (q, ^1^ *J*_C–F_ = 269.6 Hz), 103.0 (q, ^3^ *J*_C–F_ = 2.2 Hz), 21.3. ^19^F NMR (565 MHz, CDCl_3_) δ −62.76 (s, CF_3_). IR (neat): ν 3153, 2963, 1580, 1502, 1364, 1238, 1156, 1139, 1014 cm^–1^. (+)-ESI-MS (*m*/*z*): 370.4 (100, [M + H]^+^). Anal. Calcd for C_19_H_14_F_3_N_5_ (369.4): C, 61.79; H, 3.82; N, 18.96. Found: C, 61.70; H, 3.98; N, 19.07.

#### General Procedure for Synthesis of Azides **5**

To a solution of 1-aryl-3-(trifluoromethyl)­pyrazole **3** (1.00 mmol) in anhydrous THF (10 mL), at −78 °C, under argon, was added *n*-BuLi (2.5 M in hexane, 0.52 mL, 1.30 mmol). After 5 min, a solution of tosyl azide (405 mg, 2.05 mmol) in dry THF (5 mL) was added dropwise. The reaction mixture was allowed to warm to room temperature and stirred for 4 h. The reaction was quenched with 1 M NH_4_Cl­(aq) solution (15 mL) and extracted with DCM (3 × 20 mL). The combined organic layers were washed with water (3 × 10 mL), dried over Na_2_SO_4_, and filtered, and the solvents were removed *in vacuo*. The crude product **5** was purified by standard column chromatography on silica gel (CC).

#### 5-Azido-1-(*p*-tolyl)-3-(trifluoromethyl)-1*H*-pyrazole (**5a**)

CC (SiO_2_, hexane/DCM 4:1); red solid, 222 mg (83%); mp 45–47 °C. ^1^H NMR (600 MHz, CDCl_3_) δ: 7.48–7.45 (m, 2H), 7.29–7.27 (m, 2H), 6.45 (s, 1H), 2.41 (s, 3H). ^13^C­{^1^H} NMR (151 MHz, CDCl_3_) δ 142.9 (q, ^2^ *J*_C–F_ = 38.7 Hz), 139.4, 139.0, 134.9, 129.8, 124.0, 120.9 (q, ^1^ *J*_C–F_ = 268.7 Hz), 94.1 (q, ^3^ *J*_C–F_ = 2.5 Hz), 21.3. ^19^F NMR (565 MHz, CDCl_3_) δ −63.02 (s, CF_3_). IR (neat): ν 2922, 2136 (N_3_), 1517, 1472, 1282, 1233, 1162, 1107, 972, 816 cm^–1^. (+)-ESI-MS (*m*/*z*): 268.3 (100, [M + H]^+^); Anal. Calcd for C_11_H_8_F_3_N_5_ (267.2): C, 49.44; H, 3.02; N, 26.21. Found: C, 49.40; H, 3.02; N, 26.19.

## Supplementary Material





## Data Availability

The data underlying this study are available in the published article and its .

## Notes

Synthetic procedures, characterization data, NMR spectra of all new compounds, and crystallographic analysis (PDF)
FAIR data, including the primary NMR FID files, for compounds **1aa**–**1dd**, **5a**–**5g**, **8**, and **9** (ZIP)
