# Supplementary material for: Assembly of CF3‑Pyrazole–Triazole Hybrids through (3 + 3)-Cycloaddition/Ring Contraction and Click Chemistry
Source: J Org Chem. 2026 Feb 16;91(8):3321–8. doi: 10.1021/acs.joc.5c03120 (PMC13298894; doi:10.1021/acs.joc.5c03120)
Supplement: Supplementary file 1 [file jo5c03120_si_001.pdf]

Supporting Information

for

Assembly of CF_3-pyrazole-triazole hybrids through (3+3)-cycloaddition/ring contraction and *click* chemistry

Kamil Świątek,[a] Greta Utecht-Jarzyńska,[a] Barbara Olszewska,[a] Katarzyna Gach-Janczak,[b] and Marcin Jasiński[a,*]

[a] *University of Lodz, Faculty of Chemistry, Department of Organic and Applied Chemistry, Tamka 12, 91403 Łódź, Poland;*
[b] *Department of Biomolecular Chemistry, Medical University of Lodz, Mazowiecka 6/8, 92215 Łódź, Poland*

* Corresponding author:

Marcin Jasiński – *University of Lodz, Faculty of Chemistry, Department of Organic and Applied Chemistry, Tamka 12, 91403 Łódź, Poland;* https://orcid.org/0000-0002-8789-9690; Phone: +48 42 635 5766;
Email: marcin.jasinski@chemia.uni.lodz.pl

Content

1. General information S2

2. Synthetic procedures and characterization data S3

3. Copies of NMR spectra S26

4. Crystallographic analysis S80

5. References S87

1. General information

Experimental procedures: All commercially available reagents and solvents were used as received. Products were purified by filtration through a short plug of silica gel (FCC) or standard column chromatography (CC; SiO_2, 230-400 mesh) by using freshly distilled solvents, and recrystallized from appropriate solvents. NMR spectra were taken on a Bruker AVIII instrument (^{1}H at 600 MHz, ^{13}C at 151 MHz, and ^{19}F NMR at 565 MHz); chemical shifts are reported relative to the solvent residual peaks [for $CDCl_3$: ^{1}H NMR: δ = 7.26, ^{13}C NMR: δ = 77.16; for DMSO-d_6: ^{1}H NMR: δ = 2.50, ^{13}C NMR: δ = 39.52][1] or to $CFCl_3$ (δ = 0.00) used as external standard. The IR spectra were taken with an Agilent Cary 630 FTIR spectrometer, in neat. (ESI)-MS were performed with a Varian 500-MS LC Ion Trap; high resolution MS (ESI-TOF) measurements were performed with a Waters Synapt G2-Si mass spectrometer. Combustion analyses were obtained with a Vario EL III (Elementar Analysensysteme GmbH) instrument. Optical rotations were determined with a Perkin-Elmer 241 polarimeter at the temperatures indicated. Melting points were determined in capillaries with a MEL-TEMP apparatus (Laboratory Devices) or with a polarizing optical microscope (POM, Opta-Tech) and are uncorrected. Single crystals of **1bb** were measured on a XtaLAB Synergy, Dualflex, Pilatus 300K diffractometer using mirror-focused Cu Kα radiation. Crystallographic data have been deposited at the Cambridge Crystallographic Data Center as supplementary publication number CCDC-2425718. These data can be obtained free of charge from the CCDC, 12 Union Road, Cambridge CB2 1EZ, UK; fax: +44 (0) 1223 336 033; email: deposit@ccdc.cam.ac.uk (or via http://www.ccdc.cam.ac.uk/conts/retrieving.html).

Starting materials: The hydrazonoyl bromides **2a**-**2g** were prepared by NBS-mediated bromination of the azomethine group of the corresponding fluoral arylhydrazones.[2] Key 1-aryl-3-trifluoromethylpyrazoles **3a**-**3g** were obtained following a general literature procedure via condensation of mercaptoacetaldehyde (used in the form of its dimer, 1,4-thiane-2,5-diol) with nitrile imines generated *in situ* by base-mediated dehydrohalogenation of the corresponding bromides **2**, followed by spontaneous ring-contraction of the initially formed 1,3,4-thiadiazines (Scheme S1).[3] The desired pyrazoles were isolated in high yield: **3a** (X = Me; 96%), **3b** (X = *i*Pr; 85%), **3c** (X = OMe; 92%), **3d** (X = OBn; 97%), **3e** (X = Cl; 86%), **3f** (X = CF_3; 70%), **3g** (X = CN; 71%). The NMR data of the products **3a**-**3g** were in full agreement with those reported in the literature.[3]

Cautionary note: Organic azides can be explosive and toxic, and they should be handled on small scale according to safety protocols. Direct heating of organic azides as well as treatment with strong acids should be avoided.[4]

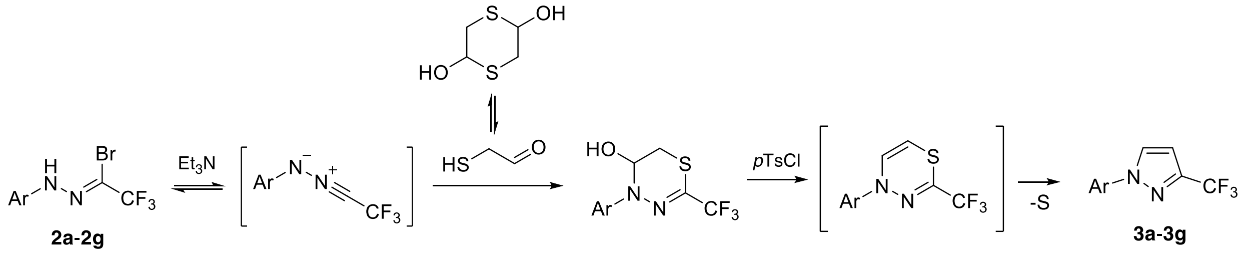

Scheme S1. One-pot synthesis of key 3-CF_3-pyrazoles **3a**-**3g**.[3]

2. Synthetic procedures and characterization data

General procedure for synthesis of pyrazole-triazole hybrids 1: A mixture of 5-azidopyrazole **1** (1.00 mmol), acetylene (1.20 mmol), copper(II) sulfate pentahydrate (38 mg, 0.15 mmol) and sodium L-ascorbate (59.5 mg, 0.30 mmol) in MeOH/H_2O (10:1, 14 mL) was stirred at 55 °C (oil bath) until the starting pyrazole was fully consumed (typically up to 5 h; TLC monitoring). The solvents were then evaporated, and the crude reaction mixture was dissolved in DCM (20 mL), dried over Na_2SO_4, and filtered through a Celite pad, which was washed with an additional portions of DCM (2 × 8 mL). After removal of the solvent in vacuo, product **1** was purified by flash column chromatography (FCC) and recrystallized from hexane.

4-Phenyl-1-(1-(*p*-tolyl)-3-(trifluoromethyl)-1*H*-pyrazol-5-yl)-1*H*-1,2,3-triazole (**1aa**):

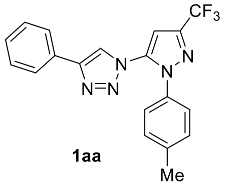

1aa

FCC (SiO_2, hexane/EtOAc 4:1); colourless solid, 314 mg (85%); mp 120-122 °C. ^1H NMR (600 MHz, $CDCl_3$) δ 7.80-7.79 (m, 2H), 7.75 (s, 1H), 7.45-7.43 (m, 2H), 7.39-7.36 (m, 1H), 7.20-7.17 (m, 4H), 7.00 (s, 1H), 2.35 (s, 3H). $^{13}C\{^1H\}$ NMR (151 MHz, $CDCl_3$) δ 148.5, 143.0 (q, $^2J_{C-F}$ = 39.6 Hz), 140.2, 135.8, 134.5, 130.3, 129.4, 129.2, 129.1, 126.1, 124.3, 121.6, 120.7 (q, $^1J_{C-F}$ = 269.6 Hz), 103.0 (q, $^3J_{C-F}$ = 2.2 Hz), 21.3. ^{19}F NMR (565 MHz, $CDCl_3$) δ –62.76 (s, CF_3). IR (neat) ν 3153, 2963, 1580, 1502, 1364, 1238, 1156, 1139, 1014 cm^{-1}. (+)-ESI-MS (*m/z*): 370.4 (100, $[M+H]^+$). Anal. calcd. for $C_{19}H_{14}F_3N_5$ (369.4): C 61.79, H 3.82, N 18.96; found: C 61.70, H 3.98, N 19.07.

1-(1-(4-Isopropylphenyl)-3-(trifluoromethyl)-1*H*-pyrazol-5-yl)-4-phenyl-1*H*-1,2,3-triazole (**1ab**)

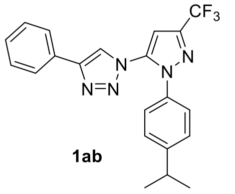

1ab

FCC (SiO_2, hexane/EtOAc 4:1); colourless solid, 309 mg (78%); mp 89-91 °C. ^1H NMR (600 MHz, $CDCl_3$) δ 7.80-7.78 (m, 3H), 7.45-7.42 (m, 2H), 7.39-7.36 (m, 1H), 7.25-7.21 (m, 4H), 6.99 (s, 1H), 2.91 (hept, *J* = 6.9 Hz, 1H), 1.22 (d, *J*= 6.9 Hz, 6H). $^{13}C\{^1H\}$ NMR (151 MHz, $CDCl_3$) δ 150.9, 148.4, 142.9 (q, $^2J_{C-F}$ = 39.5 Hz), 135.7, 134.6, 129.3, 129.2, 129.1, 127.8, 126.1, 124.3, 121.7, 120.6 (q, $^1J_{C-F}$ = 269.2 Hz), 103.0 (q, $^3J_{C-F}$ = 2.2 Hz), 34.0, 23.9. ^{19}F NMR (565 MHz, $CDCl_3$): δ –62.75 (s, CF_3). IR (neat) ν 3131, 2967, 2930, 1599, 1506, 1379, 1241, 1141, 1021, 969 cm^{-1}. (+)-ESI-MS (*m/z*): 398.3 (100, $[M+H]^+$), 420.3 (69, $[M+Na]^+$). Anal. calcd. for $C_{21}H_{18}F_3N_5$ (397.4): C 63.47, H 4.57, N 17.62; found: C 63.42, H 4.47, N 17.68.

1-(1-(4-Methoxyphenyl)-3-(trifluoromethyl)-1*H*-pyrazol-5-yl)-4-phenyl-1*H*-1,2,3-triazole (**1ac**):

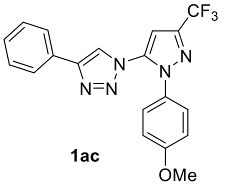

1ac

FCC (SiO_2, hexane/EtOAc 4:1); colourless solid, 320 mg (83%); mp 100-102 °C. ^1H NMR (600 MHz, $CDCl_3$) δ 7.79-7.78 (m, 3H), 7.44-7.42 (m, 2H), 7.38-7.35 (m, 1H), 7.24-7.21 (m, 2H), 6.98 (s, 1H), 6.88-6.85 (m, 2H), 3.78 (s, 3H). $^{13}C\{^1H\}$ NMR (151 MHz, $CDCl_3$) δ 160.5, 148.4, 142.8 (q, $^2J_{C\text{-}F}$ = 39.5 Hz), 135.8, 129.7, 129.3, 129.14, 129.09, 126.1, 126.0, 121.6, 120.6 (q, $^1J_{C\text{-}F}$ = 269.5 Hz), 114.8, 102.8 (q, $^3J_{C\text{-}F}$ = 2.7 Hz), 55.7. ^{19}F NMR (565 MHz, $CDCl_3$) δ –62.71 (s, CF_3). IR (neat) ν 3124, 3075, 2968, 1584, 1502, 1238, 1156, 1126, 1021, 969 cm^{-1}. (+)-ESI-MS (*m/z*): 386.3 (100, $[M+H]^+$), 408.2 (30, $[M+Na]^+$). Anal. calcd. for $C_{19}H_{14}F_3N_5O$ (385.4): C 59.22, H 3.66, N 18.17; found: C 59.17, H 3.57, N 18.09.

1-(1-(4-(Benzyloxy)phenyl)-3-(trifluoromethyl)-1*H*-pyrazol-5-yl)-4-phenyl-1*H*-1,2,3-triazole (**1ad**):

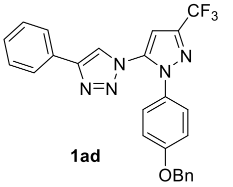

1ad

FCC (SiO_2, hexane/EtO Ac4:1); colourless solid, 373 mg (81%); mp 132-133 °C. ^1H NMR (600 MHz, $CDCl_3$) δ 7.80-7.79 (m, 2H), 7.76 (s, 1H), 7.46-7.43 (m, 2H), 7.40-7.35 (m, 5H), 7.33-7.31 (m, 1H), 7.25-7.23 (m, 2H), 6.99 (s, 1H), 6.97-6.94 (m, 2H), 5.05 (s, 2H). $^{13}C\{^1H\}$ NMR (151 MHz, $CDCl_3$) δ 159.7, 148.4, 142.9 (q, $^2J_{C\text{-}F}$ = 39.8 Hz), 136.2, 135.8, 129.9, 129.3, 129.2, 129.1, 128.8, 128.4, 127.6, 126.1, 126.0, 121.6, 120.6 (q, $^1J_{C\text{-}F}$ = 269.8 Hz), 115.8, 102.8 (q, $^3J_{C\text{-}F}$ = 2.2 Hz), 70.5. ^{19}F NMR (565 MHz, $CDCl_3$) δ –62.74 (s, CF_3). IR (neat): ν 3096, 2941, 2887, 1580, 1521, 1502, 1390, 1238, 1141, 1100, 1006, 973 cm^{-1}. (+)-ESI-MS (*m/z*): 462.4 (100, $[M+H]^+$). Anal. calcd. for $C_{25}H_{18}F_3N_5O$ (461.4): C 65.07, H 3.93, N 15.18; found: C 65.08, H 3.81, N 15.16.

1-(1-(4-Chlorophenyl)-3-(trifluoromethyl)-1*H*-pyrazol-5-yl)-4-phenyl-1*H*-1,2,3-triazole (**1ae**):

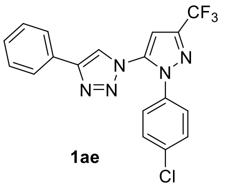

1ae

FCC (SiO_2, hexane/EtOAc 4:1); colourless solid, 335 mg (86%); mp 117-118 °C. ^1H NMR (600 MHz, $CDCl_3$) δ 7.84 (s, 1H), 7.82-7.80 (m, 2H), 7.46-7.44 (m, 2H), 7.40-7.35 (m, 3H), 7.26-7.24 (m, 2H), 7.00 (s, 1H). $^{13}C\{^1H\}$ NMR (151 MHz, $CDCl_3$) δ 148.7, 143.4 (q, $^2J_{C\text{-}F}$ = 39.8 Hz), 135.9, 135.7, 135.4, 130.0, 129.3, 129.2, 129.0, 126.1, 125.5, 121.7, 120.5 (q, $^1J_{C\text{-}F}$ = 269.2 Hz), 103.5 (q, $^3J_{C\text{-}F}$ = 2.7 Hz). ^{19}F NMR (565 MHz, $CDCl_3$): δ –62.87 (s, CF_3). IR (neat) ν 3124, 3083, 2975, 1595, 1491,1387, 1241, 1185, 1137, 1088, 1014, 969 cm^{-1}. (+)-ESI-MS (*m/z*): 390.3 (100, $[M\{^{35}Cl\}+H]^+$), 392.3 (25, $[M\{^{37}Cl\}+H]^+$). Anal. calcd. for $C_{18}H_{11}ClF_3N_5$ (389.8): C 55.47; H 2.84; N 17.97 found: C 55.38; H 2.69, N 17.94.

4-Phenyl-1-(3-(trifluoromethyl)-1-(4-(trifluoromethyl)phenyl)-1*H*-pyrazol-5-yl)-1*H*-1,2,3-triazole (**1af**):

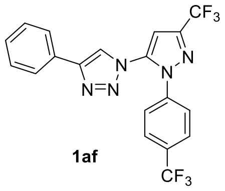

1af

FCC (SiO_2, hexane/EtOAc 4:1); colourless solid, 338 mg (80%); mp 136-138 °C. ^1H NMR (600 MHz, $CDCl_3$) δ 7.89 (s, 1H), 7.84-7.82 (m, 2H), 7.68-7.66 (m, 2H), 7.48-7.39 (m, 5H), 7.03 (s, 1H). $^{13}C\{^1H\}$ NMR (151 MHz, $CDCl_3$) δ 148.9, 143.9 (q, $^2J_{C-F}$ = 40.1 Hz), 139.7, 135.8, 131.7 (q, $^2J_{C-F}$ = 33.2 Hz), 129.4, 129.3, 128.9, 127.0 (q, $^3J_{C-F}$ = 3.8 Hz), 126.1, 124.3, 123.5 (q, $^1J_{C-F}$ = 272.5 Hz), 121.8, 120.4 (q, $^1J_{C-F}$ = 269.8 Hz), 104.0 (q, $^3J_{C-F}$ = 2.7 Hz). ^{19}F NMR (565 MHz, $CDCl_3$) δ –62.84, –62.96 (2s, $2CF_3$). IR (neat) ν 3142, 2972, 1580, 1506, 1480, 1390, 1323, 1249, 1159, 1126, 1066, 1018, 973 cm^{-1}. (+)-ESI-MS (*m/z*): 424.3 (100, $[M+H]^+$). Anal. calcd. for $C_{19}H_{11}F_6N_5$ (423.3): C 53.91, H 2.62, N 16.54; found: C 53.97, H 2.46, N 16.60.

4-(5-(4-Phenyl-1*H*-1,2,3-triazol-1-yl)-3-(trifluoromethyl)-1*H*-pyrazol-1-yl)benzonitrile (**1ag**):

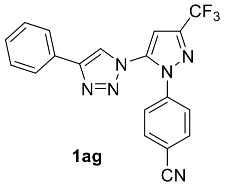

1ag

FCC (SiO_2, hexane/EtOAc 4:1); colourless solid, 255 mg (67%); mp 167-169 °C. ^1H NMR (600 MHz, $CDCl_3$) δ 7.93 (s, 1H), 7.85-7.82 (m, 2H), 7.70-7.67 (m, 2H), 7.48-7.45 (m, 2H), 7.43-7.40 (m, 3H), 7.03 (s, 1H). $^{13}C\{^1H\}$ NMR (151 MHz, $CDCl_3$) δ 149.0, 144.1 (q, $^2J_{C-F}$ = 40.3 Hz), 140.2, 135.8, 133.7, 129.5, 129.3, 128.8, 126.1, 124.3, 121.8, 120.3 (q, $^1J_{C-F}$ = 269.8 Hz), 117.5, 113.5, 104.2 (q, $^3J_{C-F}$ = 2.2 Hz). ^{19}F NMR (565 MHz, $CDCl_3$) δ –62.99 (s, CF_3). IR (neat) ν 3086, 3049, 2935, 2233 (CN), 1595, 1498, 1387, 1241, 1129, 1018, 969 cm^{-1}. (+)-ESI-MS (*m/z*): 381.3 (100, $[M+H]^+$), 403.3 (29, $[M+Na]^+$). Anal. calcd. for $C_{19}H_{11}F_3N_6$ (380.3): C 60.00, H 2.92, N 22.10; found: C 60.01, H 2.98, N 22.06.

4-(1-(1-(4-Methoxyphenyl)-3-(trifluoromethyl)-1*H*-pyrazol-5-yl)-1*H*-1,2,3-triazol-4-yl)aniline (**1ba**):

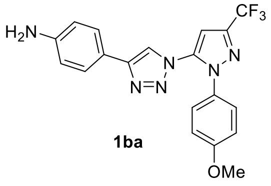

1ba

FCC (SiO_2, washed with 5% Et_3N in hexane prior to use; hexane/EtOAc 1:1); yellow solid, 245 mg (61%); mp 160-162 °C. ^1H NMR (600 MHz, $CDCl_3$) δ 7.61 (s, 1H), 7.57-7.56 (m, 2H), 7.23-7.21 (m, 2H), 6.95 (s, 1H), 6.87-6.85 (m, 2H), 6.71-6.69 (m, 2H), 3.82 (s_{br}, 2H), 3.78 (s, 3H). $^{13}C\{^1H\}$ NMR (151 MHz, $CDCl_3$) δ 160.4, 148.7, 147.4, 142.7 (q, $^2J_{C-F}$ = 39.5 Hz), 136.0, 129.8, 127.3, 125.9, 120.7 (q, $^1J_{C-F}$ = 269.3 Hz), 120.1, 119.4, 115.3, 114.8, 102.6 (q, $^3J_{C-F}$ = 2.7 Hz), 55.7. ^{19}F NMR (565 MHz, $CDCl_3$) δ –62.70 (s, CF_3). IR (neat) ν 3347, 3235, 3109, 2967, 2936, 1584, 1495, 1238, 1133, 1103, 1025, 977 cm^{-1}. (+)-ESI-MS (*m/z*): 401.4 (100, $[M+H]^+$). Anal. calcd. for $C_{19}H_{15}F_3N_6O$ (400.4): C 57.00, H 3.78, N 20.99; found: C 56.90, H 3.61, N 20.77.

4-(4-Methoxyphenyl)-1-(1-(4-methoxyphenyl)-3-(trifluoromethyl)-1*H*-pyrazol-5-yl)-1*H*-1,2,3-triazole (**1bb**):

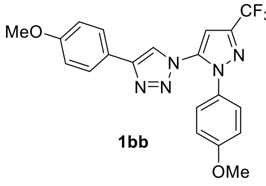

1bb

FCC (SiO_2, hexane/EtOAc 4:1); colourless solid, 340 mg (82%); mp 103-105 °C. ^{1}H NMR (600 MHz, $CDCl_3$) δ 7.72-7.70 (m, 2H), 7.66 (s, 1H), 7.25-7.23 (m, 2H), 6.97 (s, 1H), 6.97-6.95 (m, 2H), 6.89-6.86 (m, 2H), 3.84 (s, 3H), 3.79 (s, 3H). ^{13}C{^{1}H} NMR (151 MHz, $CDCl_3$) δ 160.5, 160.4, 148.3, 142.8 (q, $^2J_{C-F}$ = 39.5 Hz), 135.9, 129.8, 127.5, 126.0, 121.9, 120.7, 120.7 (q, $^1J_{C-F}$ = 269.8 Hz), 114.8, 114.6, 102.7 (q, $^3J_{C-F}$ = 2.2 Hz), 55.7, 55.5. ^{19}F NMR (565 MHz, $CDCl_3$) δ –62.74 (s, CF_3). IR (neat) *v* 3070, 2956, 2941, 2840, 1599, 1498, 1297, 1238, 1148, 1085, 1025, 969 cm^{-1}. (+)-ESI-MS (*m/z*): 416.3 (100, $[M+H]^+$). Anal. calcd. for $C_{20}H_{16}F_3N_5O_2$ (415.4): C 57.83, H 3.88, N 16.86; found: C 57.85, H 3.66, N 16.76. Crystals of **1bb** suitable for X-ray measurements were obtained from hexane solution by slow evaporation of the solvent.

1-(1-(4-Methoxyphenyl)-3-(trifluoromethyl)-1*H*-pyrazol-5-yl)-4-(*p*-tolyl)-1*H*-1,2,3-triazole (**1bc**):

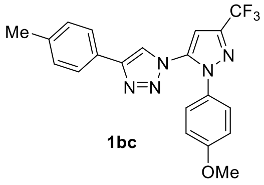

1bc

FCC (SiO_2, hexane/EtOAc 4:1); colourless solid, 291 mg (73%); mp 136-138 °C. ^{1}H NMR (600 MHz, $CDCl_3$) δ 7.73 (s, 1H), 7.68-7.67 (m, 2H), 7.24-7.22 (m, 4H), 6.97 (s, 1H), 6.87-6.85 (m, 2H), 3.78 (s, 3H), 2.38 (s, 3H). ^{13}C{^{1}H} NMR (151 MHz, $CDCl_3$) δ 160.5, 148.4, 142.7 (q, $^2J_{C-F}$ = 39.8 Hz), 139.1, 135.9, 129.8, 129.7, 126.4, 126.0*, 121.2, 120.7 (q, $^1J_{C-F}$ = 269.2 Hz, CF_3), 114.8, 102.7 (q, $^3J_{C-F}$ = 2.7 Hz), 55.6, 21.4; *higher intensity. ^{19}F NMR (565 MHz, $CDCl_3$) δ –62.71 (s, CF_3). IR (neat) *v* 3135, 3086, 2945, 2908, 2855, 1580, 1498, 1301, 1241, 1126, 1103, 1025, 969 cm^{-1}. (+)-ESI-MS (*m/z*): 400.3 (100, $[M+H]^+$), 422.2 (70, $[M+Na]^+$). Anal. calcd. for $C_{20}H_{16}F_3N_5O$ (399.4): C 60.15, H 4.04, N 17.54; found: C 60.24, H 4.08, N 17.45.

1-(1-(4-Methoxyphenyl)-3-(trifluoromethyl)-1*H*-pyrazol-5-yl)-4-(4-pentylphenyl)-1*H*-1,2,3-triazole (**1bd**):

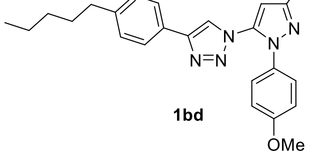

1bd

FCC (SiO_2, hexane/EtOAc 4:1); colourless solid, 410 mg (90%); mp 111-113 °C. ^{1}H NMR (600 MHz, $CDCl_3$) δ 7.73 (s_{br}, 1H), 7.71-7.69 (m, 2H), 7.26-7.23 (m, 4H), 6.98 (s, 1H), 6.88-6.86 (m, 2H), 3.79 (s, 3H), 2.63 (t, *J* = 7.7 Hz, 2H), 1.63 (quint, *J* = 7.4 Hz, 2H), 1.37-1.30 (m, 4H), 0.90 (t, *J* = 7.0 Hz, 3H). ^{13}C{^{1}H} NMR (151 MHz, $CDCl_3$) δ 160.5, 148.5 (br), 144.2, 142.8 (q, $^2J_{C-F}$ = 39.6 Hz), 135.9, 129.7,

129.2, 126.7, 126.00, 125.97, 121.3 (br), 120.7 (q, $^{1}J_{C\text{-}F}$ = 269.2 Hz), 114.8, 102.8 (q, $^{3}J_{C\text{-}F}$ = 2.2 Hz), 55.7, 35.9, 31.6, 31.2, 22.7, 14.1. ^{19}F NMR (565 MHz, $CDCl_3$) δ –62.73 (s, CF_3). IR (neat) v 3086, 2930, 2855, 1603, 1502, 1461, 1375, 1234, 1156, 1126, 1100, 1018, 973 cm^{-1}. (+)-ESI-MS (m/z): 456.4 (100, $[M+H]^+$). Anal. calcd. for $C_{24}H_{24}F_3N_5O$ (455.5): C 63.29, H 5.31, N 15.38; found: C 63.52, H 5.32, N 15.32.

4-(4-Chlorophenyl)-1-(1-(4-methoxyphenyl)-3-(trifluoromethyl)-1*H*-pyrazol-5-yl)-1*H*-1,2,3-triazole (**1be**):

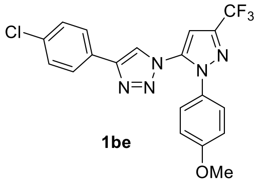

1be

FCC (SiO_2, hexane/EtOAc 4:1); colourless solid, 260 mg (62%); mp 141-143 °C. ^{1}H NMR (600 MHz, $CDCl_3$) δ 7.75 (s, 1H), 7.73-7.71 (m, 2H), 7.42-7.39 (m, 2H), 7.25-7.23 (m, 2H), 6.98 (s, 1H), 6.89-6.87 (m, 2H), 3.80 (s, 3H). $^{13}C\{^{1}H\}$ NMR (151 MHz, $CDCl_3$) δ 160.7, 147.4, 142.9 (q, $^{2}J_{C\text{-}F}$ = 39.8 Hz), 135.7, 135.0, 129.7, 129.4, 127.8, 127.4, 126.1, 121.7, 120.7 (q, $^{1}J_{C\text{-}F}$ = 269.2 Hz), 114.9, 102.8 (q, $^{3}J_{C\text{-}F}$ = 2.7 Hz), 55.7. ^{19}F NMR (565 MHz, $CDCl_3$) δ –62.78 (s, CF_3). IR (neat) v 3127, 2967, 2932, 2844, 1580, 1498, 1465, 1387, 1238, 1178, 1129, 1100, 1021, 969 cm^{-1}. (+)-ESI-MS (m/z): 420.2 (100, $[M\{^{35}Cl\}+H]^+$), 422.3 (34, $[M\{^{37}Cl\}+H]^+$). Anal. calcd. for $C_{19}H_{13}ClF_3N_5O$ (419.8): C 54.36, H 3.12, N 16.68; found: C 54.32, H 2.95, N 16.48.

4-(1-(1-(4-Methoxyphenyl)-3-(trifluoromethyl)-1*H*-pyrazol-5-yl)-1*H*-1,2,3-triazol-4-yl)benzonitrile (**1bf**):

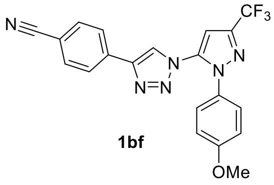

1bf

FCC (SiO_2, hexane/DCM 1:4); colourless solid, 263 mg (64%); mp 162-164 °C. ^{1}H NMR (600 MHz, $CDCl_3$) δ 7.91-7.90 (m, 2H), 7.87 (s, 1H), 7.73-7.71 (m, 2H), 7.26-7.23 (m, 2H), 7.00 (s, 1H), 6.89-6.88 (m, 2H), 3.80 (s, 3H). $^{13}C\{^{1}H\}$ NMR (151 MHz, $CDCl_3$) δ 160.7, 146.5, 142.9 (q, $^{2}J_{C\text{-}F}$ = 39.8 Hz), 135.4, 133.6, 133.0, 129.5, 126.5, 126.1, 122.8, 120.6 (q, $^{1}J_{C\text{-}F}$ = 269.2 Hz), 118.5, 114.9, 112.6, 102.9 (q, $^{3}J_{C\text{-}F}$ = 2.5 Hz), 55.7. ^{19}F NMR (565 MHz, $CDCl_3$) δ –62.74 (s, CF_3). IR (neat) v 3127, 2971, 2937, 2225 (CN), 1580, 1502, 1387, 1241, 1141, 1103, 1029, 969 cm^{-1}. (+)-ESI-MS (m/z): 411.4 (39, $[M+H]^+$), 433.3 (100, $[M+Na]^+$). Anal. calcd. for $C_{20}H_{13}F_3N_6O$ (410.4): C 58.54, H 3.19, N 20.48; found: C 58.57, H 3.14, N 20.45.

4-(2-Methoxyphenyl)-1-(1-(4-methoxyphenyl)-3-(trifluoromethyl)-1*H*-pyrazol-5-yl)-1*H*-1,2,3-triazole (**1bg**):

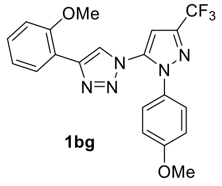

FCC (SiO_2, hexane/EtOAc 4:1); yellow solid, 378 mg (91%); mp 98-100 °C. ^{1}H NMR (600 MHz, $CDCl_3$) δ 8.36-8.34 (m, 1H), 8.06 (s, 1H), 7.36-7.33 (m, 1H), 7.26-7.25 (m, 2H), 7.11-7.08 (m, 1H), 6.99 (s_{br}, 1H), 6.98-6.96 (m, 1H), 6.89-6.86 (m, 2H), 3.85 (s, 3H), 3.78 (s, 3H). ^{13}C{^{1}H} NMR (151 MHz, $CDCl_3$) δ 160.4, 155.9, 143.7, 142.7 (q, $^2J_{C-F}$ = 39.8 Hz), 136.2, 129.9, 129.8, 127.9, 126.2, 125.0, 121.3, 120.7 (q, $^1J_{C-F}$ = 269.2 Hz), 118.2, 114.7, 111.0, 102.4 (q, $^3J_{C-F}$ = 2.5 Hz), 55.7, 55.4. ^{19}F NMR (565 MHz, $CDCl_3$) δ –62.71 (s, CF_3). IR (neat) ν 3161, 3127, 2937, 2840, 1580, 1502, 1454, 1360, 1238, 1159, 1126, 1021, 973 cm^{-1}. (+)-ESI-MS (*m/z*): 416.3 (100, [M+H]$^+$), 438.2 (48, [M+Na]$^+$). Anal. calcd. for $C_{20}H_{16}F_3N_5O_2$ (415.4): C 57.83, H 3.88, N 16.86; found: C 57.93, H 3.89, N 16.89.

4-(3-Methoxyphenyl)-1-(1-(4-methoxyphenyl)-3-(trifluoromethyl)-1*H*-pyrazol-5-yl)-1*H*-1,2,3-triazole (**1bh**):

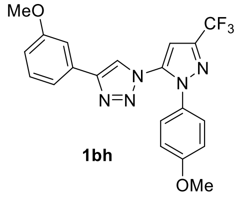

FCC (SiO_2, hexane/EtOAc 4:1); colourless solid, 357 mg (86%); mp 123-125 °C. ^{1}H NMR (600 MHz, $CDCl_3$) δ 7.75 (s, 1H), 7.41 (m_c, 1H), 7.34-7.29 (m, 2H), 7.25-7.22 (m, 2H), 6.98 (s, 1H), 6.93-6.91 (m, 1H), 6.89-6.86 (m, 2H), 3.86 (s, 3H), 3.79 (s, 3H). ^{13}C{^{1}H} NMR (151 MHz, $CDCl_3$) δ 160.5, 160.3, 148.2, 142.8 (q, $^2J_{C-F}$ = 39.8 Hz), 135.8, 130.5, 130.2, 129.7, 126.0, 121.8, 120.6 (q, $^1J_{C-F}$ = 269.2 Hz), 118.4, 115.0, 114.8, 111.4, 102.8 (q, $^3J_{C-F}$ = 2.7 Hz), 55.7, 55.5. ^{19}F NMR (565 MHz, $CDCl_3$) δ –62.73 (s, CF_3). IR (neat) ν 3159, 3004, 2959, 2930, 2840, 1580, 1521, 1484, 1357, 1312, 1252, 1156, 1129, 1021, 969 cm^{-1}. (+)-ESI-MS (*m/z*): 416.3 (100, [M+H]$^+$), 438.2 (33, [M+Na]$^+$). Anal. calcd. for $C_{20}H_{16}F_3N_5O_2$ (415.4): C 57.83, H 3.88, N 16.86; found: C 57.69, H 3.87, N 16.96.

4-Mesityl-1-(1-(4-methoxyphenyl)-3-(trifluoromethyl)-1*H*-pyrazol-5-yl)-1*H*-1,2,3-triazole (**1bi**):

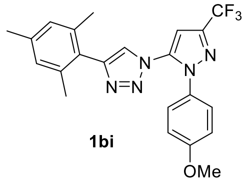

FCC (SiO_2, hexane/EtOAc 4:1); colourless solid, 350 mg (82%); mp 126-128 °C. ^{1}H NMR (600 MHz, $CDCl_3$) δ 7.35 (s, 1H), 7.22-7.19 (m, 2H), 7.08 (s, 1H), 6.92 (s_{br}, 2H), 6.89-6.86 (m, 2H), 3.80 (s, 3H), 2.31 (s, 3H), 2.00 (s, 6H). ^{13}C{^{1}H} NMR (151 MHz, $CDCl_3$) δ 160.6, 146.2, 142.9 (q, $^2J_{C-F}$ = 39.5 Hz), 139.0, 137.8, 136.1, 129.6, 128.6, 126.1, 125.7, 124.8, 120.7 (q, $^1J_{C-F}$ = 269.5 Hz), 114.9,

102.6 (q, $^3J_{C\text{-}F}$ = 2.2 Hz), 55.8, 21.2, 20.7. ^{19}F NMR (565 MHz, $CDCl_3$) δ –62.75 (s, CF_3). IR (neat) ν 3135, 3101, 2982, 2937, 1580, 1502, 1364, 1301, 1241, 1137, 1100, 1025, 977 cm^{-1}. (+)-ESI-MS (*m/z*): 428.3 (100, $[M+H]^+$), 450.3 (33, $[M+Na]^+$). Anal. calcd. for $C_{22}H_{20}F_3N_5O$ (427.4): C 61.82, H 4.72, N 16.39; found: C 61.88, H 4.78, N 16.44.

4-(6-Methoxynaphthalen-2-yl)-1-(1-(4-methoxyphenyl)-3-(trifluoromethyl)-1*H*-pyrazol-5-yl)-1*H*-1,2,3-triazole (**1bj**):

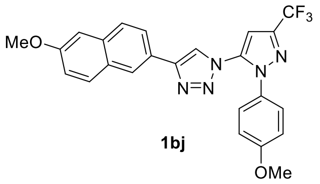

1bj

FCC (SiO_2, hexane/DCM 1:1); pale yellow solid, 278 mg (60%); mp 169-171 °C. ^{1}H NMR (600 MHz, $CDCl_3$) δ 8.24 (s, 1H), 7.85-7.81 (m, 1H), 7.80-7.74 (m, 3H), 7.27-7.24 (m, 2H), 7.18-7.16 (m, 1H), 7.14-7.12 (m, 1H), 6.99 (s, 1H), 6.88-6.86 (m, 2H), 3.93 (s, 3H), 3.77 (s, 3H). ^{13}C{^{1}H} NMR (151 MHz, $CDCl_3$) δ 160.5, 158.5, 148.6, 142.8 (q, $^2J_{C\text{-}F}$ = 39.5 Hz), 135.9, 134.9, 129.9, 129.0, 127.7, 126.9, 126.0, 125.1, 124.4, 124.3, 121.4, 120.7 (q, $^1J_{C\text{-}F}$ = 269.2 Hz), 119.8, 114.8, 106.0, 102.8 (q, $^3J_{C\text{-}F}$ = 2.2 Hz), 55.7, 55.5. ^{19}F NMR (565 MHz, $CDCl_3$) δ –62.71 (s, CF_3). IR (neat) ν 3131, 2922, 2855, 1599, 1506, 1450, 1390, 1305, 1238, 1152, 1092, 1025 cm^{-1}. (+)-ESI-MS (*m/z*): 466.3 (40, $[M+H]^+$), 488.3 (100, $[M+Na]^+$). HRMS (ESI-TOF) *m/z* calcd for $C_{24}H_{19}F_3N_5O_2$ ($[M+H]^+$): 466.1491; found: 466.1490.

4-(4-Fluorophenyl)-1-(1-(4-methoxyphenyl)-3-(trifluoromethyl)-1*H*-pyrazol-5-yl)-1*H*-1,2,3-triazole (**1bk**):

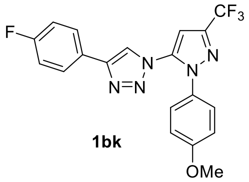

1bk

FCC (SiO_2, hexane/EtOAc 4:1); pale yellow solid, 355 mg (88%); mp 122-123 °C. ^{1}H NMR (600 MHz, $CDCl_3$) δ 7.77-7.75 (m, 2H), 7.73 (s, 1H), 7.25-7.22 (m, 2H), 7.13-7.10 (m, 2H), 6.98 (s, 1H), 6.89-6.86 (m, 2H), 3.79 (s, 3H). ^{13}C{^{1}H} NMR (151 MHz, $CDCl_3$) δ 163.2 (d, $^1J_{C\text{-}F}$ = 249.0 Hz), 160.6, 147.5, 142.8 (q, $^2J_{C\text{-}F}$ = 39.5 Hz), 135.7, 129.7, 127.9 (d, $^3J_{C\text{-}F}$ = 8.2 Hz), 126.0, 125.5 (d, $^4J_{C\text{-}F}$ = 3.3 Hz), 121.4, 120.6 (q, $^1J_{C\text{-}F}$ = 269.8 Hz), 116.2 (d, $^2J_{C\text{-}F}$ = 21.8 Hz), 114.8, 102.8 (q, $^3J_{C\text{-}F}$ = 2.5 Hz), 55.7. ^{19}F NMR (565 MHz, $CDCl_3$) δ –62.73 (s, CF_3), –111.99 (m_c, F). IR (neat) ν 3079, 2968, 2929, 1584, 1495, 1390, 1238, 1148, 1122, 1088, 1025, 969 cm^{-1}. (+)-ESI-MS (*m/z*): 404.3 (100, $[M+H]^+$). Anal. calcd. for $C_{19}H_{13}F_4N_5O$ (403.3): C 56.58, H 3.25, N 17.36; found: C 56.45, H 3.43, N 17.45.

1-(1-(4-Methoxyphenyl)-3-(trifluoromethyl)-1*H*-pyrazol-5-yl)-4-(4-(trifluoromethyl)phenyl)-1H-1,2,3-triazole (**1bl**):

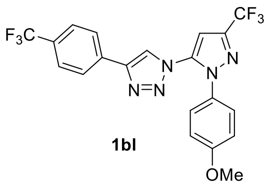

1bl

FCC (SiO_2, hexane/EtOAc 4:1); colourless solid, 367 mg (81%); mp 151-153 °C. ^{1}H NMR (600 MHz, $CDCl_3$) δ 7.92-7.90 (m, 2H), 7.83 (s, 1H), 7.71-7.69 (m, 2H), 7.26-7.24 (m, 2H), 7.00 (s, 1H), 6.90-6.87 (m, 2H), 3.80 (s, 3H). ^{13}C{^{1}H} NMR (151 MHz, $CDCl_3$) δ 160.6, 147.0, 142.9 (q, $^2J_{C-F}$ = 39.8 Hz), 135.5, 132.7, 131.0 (q, $^2J_{C-F}$ = 33.0Hz), 129.6, 126.3, 126.2 (q, $^3J_{C-F}$ = 4.1 Hz), 126.1, 124.0 (q, $^1J_{C-F}$ = 271.9 Hz), 122.4, 120.6 (q, $^1J_{C-F}$ = 269.2 Hz), 114.9, 102.9 (q, $^3J_{C-F}$ = 2.7 Hz), 55.7. ^{19}F NMR (565 MHz, $CDCl_3$) δ –62.81 (s, CF_3), –62.84 (s, CF_3). IR (neat) ν 3105, 2971, 2919, 2848, 1580, 1498, 1327, 1238, 1156, 1103, 1066, 1014, 977 cm^{-1}. (+)-ESI-MS (*m/z*): 454.2 (100, [M+H]$^+$). Anal. calcd. for $C_{20}H_{13}F_6N_5O$ (453.3): C 52.99, H 2.89, N 15.45; found: C 52.88, H 2.74, N 15.24.

1-(1-(4-Methoxyphenyl)-3-(trifluoromethyl)-1*H*-pyrazol-5-yl)-4-(4-(trifluoromethoxy)phenyl)-1*H*-1,2,3-triazole (**1bm**):

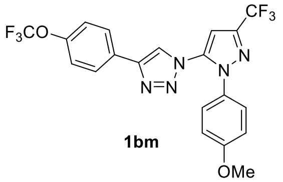

1bm

FCC (SiO_2, hexane/EtOAc 4:1); pale yellow solid, 394 mg (84%); mp 83-85 °C. ^{1}H NMR (600 MHz, $CDCl_3$) δ 7.83-7.80 (m, 2H), 7.77 (s, 1H), 7.29-7.27 (m, 2H), 7.25-7.22 (m, 2H), 6.99 (s, 1H), 6.89-6.86 (m, 2H), 3.79 (s, 3H). ^{13}C{^{1}H} NMR (151 MHz, $CDCl_3$) δ 160.6, 149.7 (q, $^3J_{C-F}$ = 2.2 Hz), 147.1, 142.9 (q, $^2J_{C-F}$ = 39.8 Hz), 135.6, 129.6, 128.0, 127.6, 126.0, 121.8, 121.7, 120.62 (q, $^1J_{C-F}$ = 269.2 Hz), 120.56 (q, $^1J_{C-F}$ = 257.8 Hz), 114.9, 102.8 (q, $^3J_{C-F}$ = 2.7 Hz), 55.7. ^{19}F NMR (565 MHz, $CDCl_3$) δ –57.85 (s, OCF_3), –62.76 (s, CF_3). IR (neat) ν 3146, 2940, 2908, 1580, 1525, 1498, 1241, 1203, 1156, 1129, 1014, 977 cm^{-1}. (+)-ESI-MS (*m/z*): 470.2 (100, [M+H]$^+$), 492.2 (26.5, [M+Na]$^+$). Anal. calcd. for $C_{20}H_{13}F_6N_5O_2$ (469.3): C 51.18, H 2.79, N 14.92; found: C 51.22, H 2.80, N 14.84.

4-(3,5-Difluorophenyl)-1-(1-(4-methoxyphenyl)-3-(trifluoromethyl)-1*H*-pyrazol-5-yl)-1*H*-1,2,3-triazole (**1bn**):

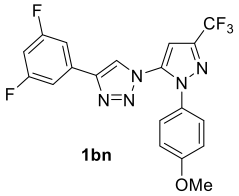

1bn

FCC (SiO_2, hexane/EtOAc 4:1); pale yellow solid, 366 mg (87%); mp 151-153 °C. ^{1}H NMR (600 MHz, $CDCl_3$) δ 7.77 (s, 1H), 7.33-7.30 (m, 2H), 7.25-7.22 (m, 2H), 6.99 (s, 1H), 6.90-6.87 (m, 2H), 6.83-6.80 (m, 1H), 3.80 (s, 3H). ^{13}C{^{1}H} NMR (151 MHz, $CDCl_3$) δ 163.5 (dd, $^{1,3}J_{C-F}$ = 249.3, 12.8 Hz), 160.7,

146.4 (t, $^{4}J_{C-F}$ = 3.5 Hz), 142.9 (q, $^{2}J_{C-F}$ = 39.8 Hz), 135.4, 132.3 (t, $^{3}J_{C-F}$ = 10.6 Hz), 129.5, 126.1, 122.4, 120.6 (q, $^{1}J_{C-F}$ = 269.5 Hz), 114.9, 109.0 (dd, $^{2,4}J_{C-F}$ = 21.0, 5.7 Hz), 104.4 (t, $^{2}J_{C-F}$ = 25.3 Hz), 102.9 (q, $^{3}J_{C-F}$ = 2.7 Hz), 55.7. ^{19}F NMR (565 MHz, $CDCl_3$) δ –62.76 (s, CF_3), –108.47 - –108.42 (m, 2F). IR (neat) ν 3135, 3101, 3027, 2948, 2851, 1577, 1525, 1465, 1249, 1182, 1133, 1092, 1029, 973 cm^{-1}. (+)-ESI-MS (*m/z*): 422.2 (100, $[M+H]^+$). Anal. calcd. for $C_{19}H_{12}F_5N_5O$ (421.3): C 54.16, H 2.87, N 16.62; found: C 54.06, H 2.85, N 16.47.

4-(3,5-Bis(trifluoromethyl)phenyl)-1-(1-(4-methoxyphenyl)-3-(trifluoromethyl)-1*H*-pyrazol-5-yl)-1*H*-1,2,3-triazole (**1bo**):

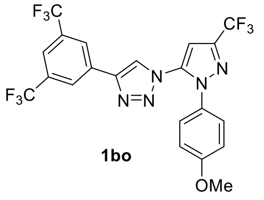

1bo

FCC (SiO_2, hexane/EtOAc 4:1); pale yellow solid, 427 mg (82%); mp 97-99 °C. ^{1}H NMR (600 MHz, $CDCl_3$) δ 8.25 (m, 2H), 7.97 (s, 1H), 7.87 (m, 1H), 7.26-7.23 (m, 2H), 7.01 (s, 1H), 6.90-6.87 (m, 2H), 3.79 (s, 3H). ^{13}C{^{1}H} NMR (151 MHz, $CDCl_3$) δ 160.7, 145.7, 142.9 (q, $^{2}J_{C-F}$ = 39.8 Hz), 135.2, 132.7 (q, $^{2}J_{C-F}$ = 33.5 Hz, i-C), 131.5, 129.5, 126.1, 126.0 (m), 123.2 (q, $^{1}J_{C-F}$ = 273.0 Hz), 122.9, 122.5 (m), 120.6 (q, $^{1}J_{C-F}$ = 269.2 Hz), 114.9, 103.1 (q, $^{3}J_{C-F}$ = 2.7 Hz), 55.7. ^{19}F NMR (565 MHz, $CDCl_3$) δ –62.79 (s, CF_3), –63.10 (s, 2CF_3). IR (neat) ν 3142, 2952, 2905, 1525, 1502, 1383, 1275, 1241, 1185, 1126, 1029, 969 cm^{-1}. (+)-ESI-MS (*m/z*): 522.3 (100, $[M+H]^+$). Anal. calcd. for $C_{21}H_{12}F_9N_5O$ (521.3): C 48.38, H 2.32, N 13.43; found: C 48.51, H 2.29, N 13.37.

(4-(1-(1-(4-Methoxyphenyl)-3-(trifluoromethyl)-1*H*-pyrazol-5-yl)-1*H*-1,2,3-triazol-4-yl)phenyl)methanol (**1bp**):

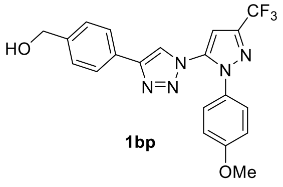

1bp

FCC (SiO_2, hexane/EtOAc 1:1); colourless solid, 307 mg (74%); mp 123-125 °C. ^{1}H NMR (600 MHz, $CDCl_3$) δ 7.78-7.75 (m, 3H), 7.43-7.41 (m, 2H), 7.24-7.22 (m, 2H), 6.98 (s, 1H), 6.88-6.85 (m, 2H), 4.72 (s, 2H), 3.78 (s, 3H), 1.92 (s_{br}, 1H). ^{13}C{^{1}H} NMR (151 MHz, $CDCl_3$) δ 160.5, 148.1, 142.8 (q, $^{2}J_{C-F}$ = 39.5 Hz), 141.9, 135.8, 129.7, 128.5, 127.6, 126.2, 126.0, 121.6, 120.6 (q, $^{1}J_{C-F}$ = 269.2 Hz), 114.8, 102.8 (q, $^{3}J_{C-F}$ = 2.2 Hz), 65.0, 55.7. ^{19}F NMR (565 MHz, $CDCl_3$) δ –62.72 (s, CF_3). IR (neat) ν 3403 (OH), 3109, 2907, 1580, 1506, 1245, 1193, 1148, 1107, 1029, 977 cm^{-1}. (+)-ESI-MS (*m/z*): 416.4 (100, $[M+H]^+$), 438.3 (40, $[M+Na]^+$). Anal. calcd. for $C_{20}H_{16}F_3N_5O_2$ (415.4): C 57.83, H 3.88, N 16.86; found: C 57.78, H 3.83, N 16.91.

4-(Ferrocenyl)-1-(1-(4-methoxyphenyl)-3-(trifluoromethyl)-1*H*-pyrazol-5-yl)-1*H*-1,2,3-triazole (**1bq**):

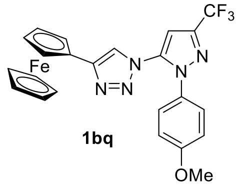

FCC (SiO_2, hexane/EtOAc 4:1); orange solid, 326 mg (66%); mp 155-157 °C. ^{1}H NMR (600 MHz, $CDCl_3$) δ 7.40 (s, 1H), 7.24-7.22 (m, 2H), 6.99 (s, 1H), 6.91-6.88 (m, 2H), 4.69 (s, 2H), 4.32 (s, 2H), 4.01 (s, 5H), 3.77 (s, 3H). ^{13}C{^{1}H} NMR (151 MHz, $CDCl_3$) δ 160.6, 148.0, 142.8 (q, $^2J_{C\text{-}F}$ = 39.8 Hz), 136.0, 129.7, 126.0, 120.7 (q, $^1J_{C\text{-}F}$ = 269.8 Hz), 120.5, 114.8, 102.7 (q, $^3J_{C\text{-}F}$ = 2.2 Hz), 73.6, 69.8, 69.3, 67.0, 55.7. ^{19}F NMR (565 MHz, $CDCl_3$) δ –62.73 (s, CF_3). IR (neat) ν 3131, 1588, 1521, 1498, 1465, 1375, 1308, 1238, 1163, 1133, 1092, 969, 820 cm^{-1}. (+)-ESI-MS (*m/z*): 493.2 (70, [M]$^+$), 494.2 (100, [M+H]$^+$), 516.2 (60, [M+Na]$^+$). Anal. calcd. for $C_{23}H_{18}F_3FeN_5O$ (493.3): C 56.00, H 3.68, N 14.20; found: C 56.06, H 3.60, N 14.41.

2-(1-(1-(4-Methoxyphenyl)-3-(trifluoromethyl)-1*H*-pyrazol-5-yl)-1*H*-1,2,3-triazol-4-yl)pyridine (**1br**):

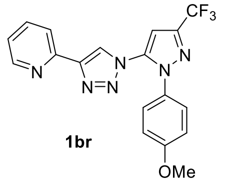

FCC (SiO_2, hexane/EtOAc4:1); pale yellow solid, 282 mg (73%); mp 109-111 °C. ^{1}H NMR (600 MHz, $CDCl_3$) δ 8.55-8.54 (m, 1H), 8.19 (s, 1H), 8.18-8.16 (m, 1H), 7.80-7.78 (m, 1H), 7.27-7.22 (m, 3H), 6.97 (s, 1H), 6.87-6.84 (m, 2H), 3.77 (s, 3H). ^{13}C{^{1}H} NMR (151 MHz, $CDCl_3$) δ 160.5, 149.7, 149.1, 148.9, 142.7 (q, $^2J_{C\text{-}F}$ = 39.8 Hz), 137.2, 135.7, 129.7, 126.0, 124.3, 123.7, 120.7, 120.6 (q, $^1J_{C\text{-}F}$ = 269.2 Hz), 114.8, 102.9 (q, $^3J_{C\text{-}F}$ = 2.2 Hz), 55.6. ^{19}F NMR (565 MHz, $CDCl_3$) δ –62.70 (s, CF_3). IR (neat) ν 3094, 2972, 2949, 1595, 1506, 1469, 1428, 1375, 1238, 1163, 1133, 1098, 1025, 973 cm^{-1}. (+)-ESI-MS (*m/z*): 387.3 (100, [M+H]$^+$), 409.3 (75, [M+Na]$^+$). Anal. calcd. for $C_{18}H_{13}F_3N_6O$ (386.3): C 55.96, H 3.39, N 21.75; found: C 55.93, H 3.37, N 21.67.

1-(1-(4-Methoxyphenyl)-3-(trifluoromethyl)-1*H*-pyrazol-5-yl)-4-(thiophen-3-yl)-1*H*-1,2,3-triazole (**1bs**):

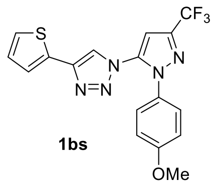

FCC (SiO_2, hexane/EtOAc 1:1); colourless solid, 341 mg (87%); mp 116-118 °C. ^{1}H NMR (600 MHz, $CDCl_3$) δ 7.72 (m$_c$, 1H), 7.66 (s, 1H), 7.40-7.39 (m, 2H), 7.25-7.22 (m, 2H), 6.97 (s, 1H), 6.89-6.86 (m, 2H), 3.80 (s, 3H). ^{13}C{^{1}H} NMR (151 MHz, $CDCl_3$) δ 160.5, 144.5, 142.8 (q, $^2J_{C\text{-}F}$ = 39.5 Hz), 135.7, 130.4, 129.7, 127.0, 126.0, 125.8, 122.5, 121.3, 120.6 (q, $^1J_{C\text{-}F}$ = 269.5 Hz), 114.9, 102.8 (q, $^3J_{C\text{-}F}$ = 2.5 Hz), 55.7. ^{19}F NMR (565 MHz, $CDCl_3$) δ –62.74 (s, CF_3). IR (neat) ν 3101, 2971, 2937, 2840, 1588, 1506, 1387, 1238, 1182, 1126, 1029, 973 cm^{-1}. (+)-ESI-MS (*m/z*): 392.3 (100, [M+H]$^+$), 414.3 (12, [M+Na]$^+$). Anal. calcd. for $C_{17}H_{12}F_3N_5OS$ (391.4): C 52.17, H 3.09, N 17.89, S 8.19; found: C 52.17, H 3.12, N 17.77, S 8.18.

4-(4-Ethynylphenyl)-1-(1-(4-methoxyphenyl)-3-(trifluoromethyl)-1*H*-pyrazol-5-yl)-1*H*-1,2,3-triazole (**1bt**):

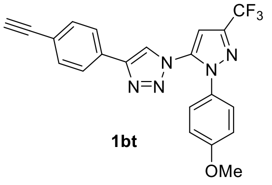

1bt

Following the general procedure, azide **5c** (283 mg, 1.00 mmol) was reacted with 1,4-diethynylbenzene (202 mg, 1.60 mmol). FCC (SiO_2, hexane/EtOAc 4:1); pale yellow solid, 340 mg (83%); mp 143-145 °C. ^{1}H NMR (600 MHz, $CDCl_3$) δ 7.77 (s, 1H), 7.76-7.74 (m, 2H), 7.56-7.54 (m, 2H), 7.25-7.22 (m, 2H), 6.98 (s, 1H), 6.89-6.87 (m, 2H), 3.79 (s, 3H), 3.15 (s, 1H). ^{13}C{^{1}H} NMR (151 MHz, $CDCl_3$) δ 160.6, 147.6, 142.9 (q, $^2J_{C\text{-}F}$ = 39.5 Hz), 135.7, 132.9, 129.64, 129.55, 126.0, 125.9, 122.8, 121.9, 120.6 (q, $^1J_{C\text{-}F}$ = 269.2 Hz), 114.9, 102.8 (q, $^3J_{C\text{-}F}$ = 2.2 Hz), 83.2, 78.7, 55.7. ^{19}F NMR (565 MHz, $CDCl_3$) δ –62.74 (s, CF_3). IR (neat) v 3295, 3150, 2961, 2948, 1580, 1502, 1238, 1129, 1010, 977 cm^{-1}. (+)-ESI-MS (*m/z*): 410.3 (60, $[M+H]^+$), 432.2 (100, $[M+Na]^+$). Anal. calcd. for $C_{21}H_{14}F_3N_5O$ (409.4): C 61.61, H 3.45, N 17.11; found: C 61.75, H 3.46, N 17.25.

1,4-Bis(1-(1-(4-methoxyphenyl)-3-(trifluoromethyl)-1*H*-pyrazol-5-yl)-1*H*-1,2,3-triazol-4-yl)benzene (**1bu**):

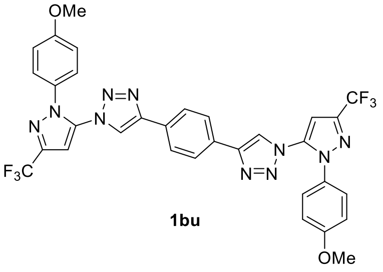

1bu

Following the general procedure, azide **5c** (708 mg, 2.50 mmol) was reacted with 1,4-diethynylbenzene (126 mg, 1.00 mmol). Crude product **1bu** was washed with hexane; yellow solid, 388 mg (56%); mp 227 °C (decomp.). ^{1}H NMR (600 MHz, DMSO-d_6) δ 9.09 (s, 2H), 7.97 (s, 4H), 7.64 (s, 2H), 7.35-7.32 (m, 4H), 7.02-6.99 (m, 4H), 3.76 (s, 6H). ^{13}C{^{1}H} NMR (151 MHz, DMSO-d_6) δ 159.9, 146.2, 140.9 (q, $^2J_{C\text{-}F}$ = 38.4 Hz), 136.0, 129.43, 129.39, 126.2, 126.1, 124.9, 120.8 (q, $^1J_{C\text{-}F}$ = 268.7 Hz), 114.6, 103.6 (br), 55.5. ^{19}F NMR (565 MHz, $CDCl_3$) δ –61.28 (s, CF_3). IR (neat) v 3131, 2949, 1580, 1502, 1390, 1238, 1133, 1029, 969 cm^{-1}. (+)-ESI-MS (*m/z*): 693.3 (100, $[M+H]^+$). Anal. calcd. for $C_{32}H_{22}F_6N_{10}O_2$ (692.6): C 55.50, H 3.20, N 20.22; found: C 55.54, H 3.12, N 20.19.

4-Hexyl-1-(1-(4-methoxyphenyl)-3-(trifluoromethyl)-1*H*-pyrazol-5-yl)-1*H*-1,2,3-triazole (**1ca**):

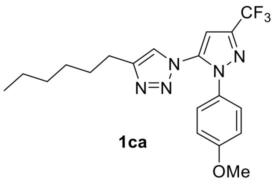

1ca

FCC (SiO_2, hexane/EtOAc4:1); pale yellow solid, 311 mg (79%); mp 66-68 °C. ^{1}H NMR (600 MHz, $CDCl_3$) δ 7.24 (s, 1H), 7.17-7.15 (m, 2H), 6.92 (s, 1H), 6.87-6.84 (m, 2H), 3.80 (s, 3H), 2.70 (t, $^3J_{H\text{-}H}$ ≈ 7.6 Hz, 2H), 1.65-1.60 (m, 2H), 1.30-1.24 (m, 6H), 0.89-0.85 (m, 3H). ^{13}C{^{1}H} NMR (151 MHz, $CDCl_3$) δ 160.4, 149.1, 142.7 (q, $^2J_{C\text{-}F}$ = 39.8 Hz), 136.1, 129.8, 125.9, 123.0, 120.7 (q, $^1J_{C\text{-}F}$ = 269.8 Hz), 114.7,

102.6 (q, $^{3}J_{C-F}$ = 2.7 Hz), 55.7, 31.6, 29.1, 28.7, 25.4, 22.6, 14.1. ^{19}F NMR (565 MHz, $CDCl_3$) δ –62.78 (s, CF_3). IR (neat) ν 2948, 2926, 2851, 1506, 1464, 1387, 1238, 1133, 1092, 1040, 973 cm^{-1}. (+)-ESI-MS (*m/z*): 394.3 (15, [M+H]$^+$), 416.3 (100, [M+Na]$^+$). Anal. calcd. for $C_{19}H_{22}F_3N_5O$ (393.4): C 58.01, H 5.64, N 17.80; found: C 57.95, H 5.66, N 17.78.

4-(*Tert*-butyl)-1-(1-(4-methoxyphenyl)-3-(trifluoromethyl)-1*H*-pyrazol-5-yl)-1*H*-1,2,3-triazole (**1cb**):

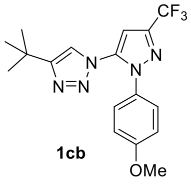

1cb

FCC (SiO_2, hexane/EtOAc 4:1); thick yellow oil, 230 mg (63%). ^{1}H NMR (600 MHz, $CDCl_3$) δ 7.22 (s, 1H), 7.17-7.15 (m, 2H), 6.92 (s, 1H), 6.87-6.85 (m, 2H), 3.80 (s, 3H), 1.31 (s, 9H). ^{13}C{^{1}H} NMR (151 MHz, $CDCl_3$) δ 160.4, 158.5, 142.7 (q, $^{2}J_{C-F}$ = 39.5 Hz), 136.2, 129.9, 125.8, 121.0, 120.7 (q, $^{1}J_{C-F}$ = 269.2 Hz), 114.6, 102.6 (q, $^{3}J_{C-F}$ = 2.5 Hz), 55.7, 31.0, 30.2. ^{19}F NMR (565 MHz, $CDCl_3$) δ –62.78 (s, CF_3). IR (neat) ν 2967, 1580, 1502, 1461, 1364, 1245, 1170, 1139, 1093, 1029, 973 cm^{-1}. (+)-ESI-MS (*m/z*): 366.4 (100, [M+H]$^+$). Anal. calcd. for $C_{17}H_{18}F_3N_5O$ (365.4): C 55.89, H 4.97, N 19.17; found: C 55.98, H 4.99, N 19.13.

1-(1-(4-Methoxyphenyl)-3-(trifluoromethyl)-1*H*-pyrazol-5-yl)-1*H*-1,2,3-triazole (**1cc**):

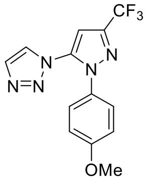

1cc

Following the general procedure, azide **5c** (283 mg, 1.00 mmol) was reacted with TMS-acetylene (118 mg, 1.20 mmol). FCC (SiO_2, hexane/EtOAc4:1); colourless solid, 164 mg (53%); mp 115-117 °C. ^{1}H NMR (600 MHz, $CDCl_3$) δ 7.76 (d, $^{3}J_{H-H}$ = 0.9 Hz, 1H), 7.57 (d, $^{3}J_{H-H}$ = 0.9 Hz, 1H), 7.18-7.15 (m, 2H), 6.96 (s, 1H), 6.88-6.85 (m, 2H), 3.80 (s, 3H). ^{13}C{^{1}H} NMR (151 MHz, $CDCl_3$) δ 160.6, 142.8 (q, $^{2}J_{C-F}$ = 39.5 Hz), 135.8, 134.3, 129.6, 126.1, 126.0, 120.6 (q, $^{1}J_{C-F}$ = 269.5 Hz), 114.8, 102.8 (q, $^{3}J_{C-F}$ = 2.5 Hz), 55.7. ^{19}F NMR (565 MHz, $CDCl_3$) δ –62.77 (s, CF_3). IR (neat) ν 3135, 1595, 1506, 1387, 1238, 1170, 1126, 1018, 969 cm^{-1}. (+)-ESI-MS (*m/z*): 310.2 (100, [M+H]$^+$), 332.1 (40, [M+Na]$^+$). HRMS (ESI-TOF) *m/z* calcd for $C_{13}H_{11}F_3N_5O$ ([M+H]$^+$): 310.0916; found: 310.0915.

(1-(1-(4-Methoxyphenyl)-3-(trifluoromethyl)-1*H*-pyrazol-5-yl)-1*H*-1,2,3-triazol-4-yl)methanol (**1cd**):

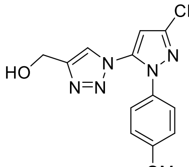

1cd FCC (SiO_2, hexane/EtOAc 3:7); colourless solid, 207 mg (61%); mp 73-75 °C. ^1H NMR (600 MHz, $CDCl_3$) δ 7.54 (s, 1H), 7.21-7.18 (m, 2H), 6.93 (s, 1H), 6.89-6.86 (m, 2H), 4.80 (s, 2H), 3.81 (s, 3H), 2.17 (s_{br}, 1H). $^{13}C\{^1H\}$ NMR (151 MHz, $CDCl_3$) δ 160.6, 148.3, 142.8 (q, $^2J_{C-F}$ = 39.5 Hz), 135.8, 129.6, 126.1, 124.1, 120.6 (q, $^1J_{C-F}$ = 269.8 Hz), 114.8, 102.7 (q, $^3J_{C-F}$ = 2.7 Hz), 56.4, 55.7. ^{19}F NMR (565 MHz, $CDCl_3$) δ –62.76 (s, CF_3). IR (neat) ν 3269, 3060, 2967, 2926, 1521, 1498, 1387, 1238, 1178, 1133, 1089, 1018, 973 cm^{-1}. (+)-ESI-MS (*m/z*): 340.2 (50, $[M+H]^+$), 362.2 (100, $[M+Na]^+$). Anal. calcd. for $C_{14}H_{12}F_3N_5O_2$ (339.3): C 49.56, H 3.57, N 20.64; found: C 49.70, H 3.60, N 20.49.

4-(Diethoxymethyl)-1-(1-(4-methoxyphenyl)-3-(trifluoromethyl)-1*H*-pyrazol-5-yl)-1*H*-1,2,3-triazole (**1ce**):

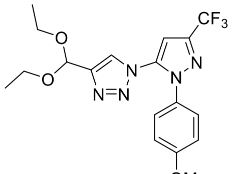

1ce FCC (SiO_2, hexane/EtOAc4:1); colourless solid, 144 mg (35%); mp 75-77 °C. ^1H NMR (600 MHz, $CDCl_3$) δ 7.58 (s, 1H), 7.19-7.17 (m, 2H), 6.93 (s, 1H), 6.87-6.84 (m, 2H), 5.70 (s, 1H), 3.80 (s, 3H), 3.58 (m, 4H), 1.20 (t, $^3J_{H-H}$ ≈ 7.1 Hz, 6H). $^{13}C\{^1H\}$ NMR (151 MHz, $CDCl_3$) δ 160.6, 147.8, 142.8 (q, $^2J_{C-F}$ = 39.8 Hz), 135.8, 129.7, 126.1, 124.8, 120.6 (q, $^1J_{C-F}$ = 269.2 Hz), 114.7, 102.8 (q, $^3J_{C-F}$ = 2.7 Hz), 96.3, 61.9, 55.7, 15.2. ^{19}F NMR (565 MHz, $CDCl_3$) δ –62.78 (s, CF_3). IR (neat) ν 2974, 2919, 1599, 1502, 1357, 1238, 1133, 1036, 1006, 969 cm^{-1}. (–)-ESI-MS (*m/z*): 410.4 (100, $[M–H]^-$). Anal. calcd. for $C_{18}H_{20}F_3N_5O_3$ (411.4): C 52.55, H 4.90, N 17.02; found: C 52.53, H 4.81, N 17.09.

Methyl 1-(1-(4-methoxyphenyl)-3-(trifluoromethyl)-1*H*-pyrazol-5-yl)-1*H*-1,2,3-triazole-4-carboxylate (**1cf**):

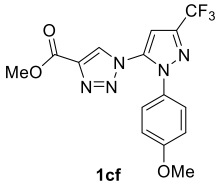

1cf FCC (SiO_2, hexane/EtOAc 4:1); colourless solid, 232 mg (63%); mp 124-126 °C. ^1H NMR (600 MHz, $CDCl_3$) δ 8.08 (s, 1H), 7.20-7.17 (m, 2H), 7.00 (s, 1H), 6.90-6.87 (m, 2H), 3.96 (s, 3H), 3.81 (s, 3H). $^{13}C\{^1H\}$ NMR (151 MHz, $CDCl_3$) δ 160.8, 160.3, 142.9 (q, $^2J_{C-F}$ = 39.8 Hz), 140.5, 134.8, 129.8, 129.1, 126.1, 120.5 (q, $^1J_{C-F}$ = 269.2 Hz), 115.0, 103.2 (q, $^3J_{C-F}$ = 2.7 Hz), 55.7, 52.7. ^{19}F NMR (565 MHz, $CDCl_3$) δ –62.79 (s, CF_3). IR (neat) ν 3142, 2960, 1730 (C=O), 1506, 1439, 1338, 1238, 1129, 1033, 973 cm^{-1}. (+)-ESI-MS (*m/z*): 390.2 (52, $[M+Na]^+$), 406.2 (100, $[M+K]^+$). Anal. calcd. for $C_{15}H_{12}F_3N_5O_3$ (367.3): C 49.05, H 3.29, N 19.07; found: C 49.00, H 3.14, N 18.92.

1-Bromo-5-(1-(1-(4-methoxyphenyl)-3-(trifluoromethyl)-1*H*-pyrazol-5-yl)-1*H*-1,2,3-triazol-4-yl)pentan-2-one (**1cg**):

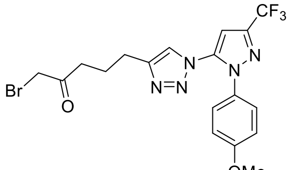

1cg FCC (SiO_2, hexane/EtOAc1:1); colourless oil, 191 mg (41%). ^{1}H NMR (600 MHz, $CDCl_3$) δ 7.29 (s, 1H), 7.18-7.16 (m, 2H), 6.93 (s, 1H), 6.89-6.86 (m, 2H), 3.85 (s, 2H), 3.81 (s, 3H), 2.75 (t, $^3J_{H-H}$ = 7.6 Hz, 2H), 2.69 (t, $^3J_{H-H}$ = 7.1 Hz, 2H), 1.98 (quint, $^3J_{H-H}$ ≈ 7.3 Hz, 2H). ^{13}C{^{1}H} NMR (151 MHz, $CDCl_3$) δ 201.6, 160.5, 147.8, 142.8 (q, $^2J_{C-F}$ = 39.8 Hz), 136.0, 129.7, 126.0, 123.3, 120.6 (q, $^1J_{C-F}$ = 269.2 Hz, CF_3), 114.8, 102.6 (q, $^3J_{C-F}$ = 2.2 Hz), 55.7, 38.6, 34.2, 24.4, 23.2. ^{19}F NMR (565 MHz, $CDCl_3$) δ –62.77 (s, CF_3). IR (neat) ν 3228, 2937, 1730 (C=O), 1580, 1502, 1238, 1133, 1096, 1029, 973 cm^{-1}. HRMS (ESI-TOF) *m/z* calcd for $C_{18}H_{18}F_3N_5O_2Br$ ([M+H]$^+$): 472.0596; found: 472.0594.

2-(3-(2-(1-(1-(4-Methoxyphenyl)-3-(trifluoromethyl)-1*H*-pyrazol-5-yl)-1*H*-1,2,3-triazol-4-yl)ethyl)-3*H*-diazirin-3-yl)ethan-1-ol (**1ch**):

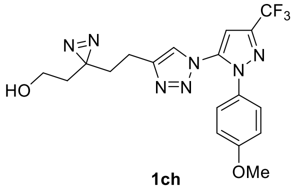

1ch FCC (SiO_2, hexane/EtOAc 1:1); thick colourless oil, 341 mg (81%). ^{1}H NMR (600 MHz, $CDCl_3$) δ 7.36 (s, 1H), 7.15-7.12 (m, 2H), 6.90 (s, 1H), 6.85-6.82 (m, 2H), 3.76 (s, 3H), 3.40 (t, $^3J_{H-H}$ ≈ 6.1Hz, 2H), 2.59 (s$_{br}$, 1H), 2.49 (t, $^3J_{H-H}$ ≈ 7.5Hz, 2H), 1.84 (t, $^3J_{H-H}$ ≈ 7.5Hz, 2H), 1.55 (t, $^3J_{H-H}$ ≈ 6.1Hz, 2H). ^{13}C{^{1}H} NMR (151 MHz, $CDCl_3$) δ 160.4, 146.9, 142.6 (q, $^2J_{C-F}$ = 39.5 Hz, C-3), 135.8, 129.6, 125.9, 123.6, 120.6 (q, $^1J_{C-F}$ = 269.2 Hz, CF_3), 114.7, 102.6 (q, $^3J_{C-F}$ = 2.7 Hz, C-4), 57.0, 55.6, 35.8, 32.3, 26.7, 19.7. ^{19}F NMR (565 MHz, $CDCl_3$) δ –62.74 (s, CF_3). IR (neat) ν 3392 (OH), 3135, 2926, 2848, 1584, 1502, 1383, 1238, 1133, 1092, 973 cm^{-1}. HRMS (ESI-TOF) *m/z* calcd for $C_{18}H_{19}F_3N_7O_2$ ([M+H]$^+$): 422.1552; found: 422.1552.

N-Benzyl-1-(1-(1-(4-methoxyphenyl)-3-(trifluoromethyl)-1*H*-pyrazol-5-yl)-1*H*-1,2,3-triazol-4-yl)-*N*-methylmethanamine (**1da**):

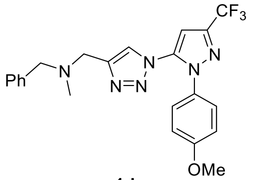

1da FCC (SiO_2, hexane/EtOAc 1:1); thick light yellow oil, 301 mg (68%). ^{1}H NMR (600 MHz, $CDCl_3$) δ 7.46 (s, 1H), 7.31-7.28 (m, 2H), 7.26-7.21 (m, 3H), 7.18-7.15 (m, 2H), 6.96 (s, 1H), 6.84-6.81 (m, 2H), 3.72, (s, 2H), 3.70 (s, 3H), 3.46 (s, 2H), 2.19 (s, 3H). ^{13}C{^{1}H} NMR (151 MHz, $CDCl_3$) δ 160.5, 146.3, 142.8 (q, $^2J_{C-F}$ = 39.5 Hz, C-3), 138.6, 136.0, 129.7, 129.0, 128.5, 127.4, 125.9, 124.9, 120.7 (q, $^1J_{C-F}$ = 269.5

Hz, CF_3), 114.8, 102.7 (q, $^3J_{C-F}$ = 2.2 Hz, C-4), 61.3, 55.6, 51.8, 42.3. ^{19}F NMR (565 MHz, $CDCl_3$) δ –62.76 (s, CF_3). IR (neat) ν 3135, 2937, 2840, 2796, 1599, 1502, 1368, 1238, 1181, 1133, 1092, 1025, 973 cm^{-1}. HRMS (ESI-TOF) *m/z* calcd for $C_{22}H_{22}F_3N_6O$ ([M+H]$^+$): 443.1807; found: 443.1812.

6,7-Bis(2-methoxyethoxy)-*N*-(4-(1-(1-(4-methoxyphenyl)-3-(trifluoromethyl)-1*H*-pyrazol-5-yl)-1*H*-1,2,3-triazol-4-yl)phenyl)quinazolin-4-amine (**1db**):

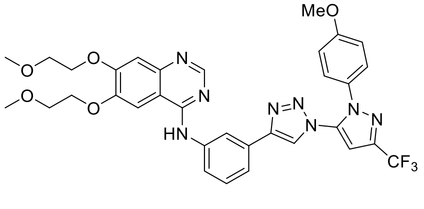

1db

Following the general procedure, azide **5c** (425 mg, 1.50 mmol) was reacted with *erlotinib* (393 mg, 1.00 mmol). Reaction time: 40 h; FCC (SiO_2, EtOAc/MeOH 9:1); yellow solid, 568 mg (84%); mp 96-100 °C (decomp). ^{1}H NMR (600 MHz, $CDCl_3$) δ 8.58 (s_{br}, 1H), 8.15 (s, 1H), 7.83-7.72 (m, 3H), 7.44-7.17 (m, 6H), 6.96 (s, 1H), 6.83-6.81 (m, 2H), 4.23-4.16 (m, 4H), 3.78-3.75 (m, 4H), 3.74 (s, 3H), 3.39 (s, 6H). ^{13}C{^{1}H} NMR (151 MHz, $CDCl_3$) δ 160.5, 156.4, 154.6, 153.4, 149.1, 148.0, 147.3, 142.7 (q, $^2J_{C-F}$ = 39.5 Hz, C-3), 139.6, 135.7, 129.9, 129.7, 129.61, 129.57, 125.9, 122.3, 122.1, 121.4, 120.6 (q, $^1J_{C-F}$ = 269.5 Hz, CF_3), 119.2, 114.8, 109.4, 108.7, 102.8 (br), 71.0, 70.5, 69.2, 68.4, 59.33, 59.30, 55.6. ^{19}F NMR (565 MHz, $CDCl_3$) δ –62.67 (s, CF_3). IR (neat) ν 2926, 1580, 1502, 1431, 1390, 1238, 1126, 1025, 973 cm^{-1}. HRMS (ESI-TOF) *m/z* calcd for $C_{33}H_{32}F_3N_8O_5$ ([M+H]$^+$): 677.2448; found: 677.2439.

(*R*)-*N*-((1-(1-(4-methoxyphenyl)-3-(trifluoromethyl)-1*H*-pyrazol-5-yl)-1*H*-1,2,3-triazol-4-yl)methyl)-2,3-dihydro-1*H*-inden-1-amine (**1dc**):

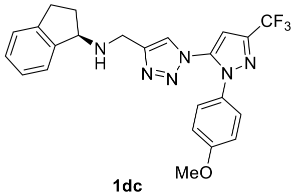

1dc

FCC (SiO_2, hexane/EtOAc 1:2); thick light yellow oil, 349 mg (77%); $[\alpha]_D^{23}$ = +1.20 (*c* = 0.37, $CHCl_3$). ^{1}H NMR (600 MHz, $CDCl_3$) δ 7.51 (s, 1H), 7.28-7.27 (m, 1H), 7.24-7.16 (m, 5H), 6.93 (s, 1H), 6.85-6.83 (m, 2H), 4.20 (t, $^3J_{H-H}$ ≈ 6.5 Hz, 1H), 4.04 (d, $^2J_{H-H}$ = 14.3 Hz, 1H), 4.01 (d, $^2J_{H-H}$ = 14.3 Hz, 1H), 3.74 (s, 3H), 3.03-2.98 (m, 1H), 2.84-2.78 (m, 1H), 2.41-2.35 (m, 1H), 2.03 (s_{br}, 1H), 1.86-1.80 (m, 1H). ^{13}C{^{1}H} NMR (151 MHz, $CDCl_3$) δ 160.5, 148.2, 144.6, 143.7, 142.8 (q, $^2J_{C-F}$ = 39.5 Hz), 136.0, 129.7, 127.8, 126.5, 126.1, 125.0, 124.22, 124.15, 120.7 (q, $^1J_{C-F}$ = 269.2 Hz, CF_3), 114.8, 102.6 (q, $^3J_{C-F}$ = 2.7 Hz), 62.8, 55.6, 42.3, 33.6, 30.5. ^{19}F NMR (565 MHz, $CDCl_3$) δ –62.75 (s, CF_3). IR (neat) ν 2933, 2848, 1580, 1502, 1461, 1238, 1182, 1133, 1092, 1029, 973 cm^{-1}. HRMS (ESI-TOF) *m/z* calcd for $C_{23}H_{22}F_3N_6O$ ([M+H]$^+$): 455.1807; found: 455.1811.

(8*S*,13*S*,14*S*,17*S*)-13-Ethyl-17-hydroxy-17-(1-(1-(4-methoxyphenyl)-3-(trifluoromethyl)-1*H*-pyrazol-5-yl)-1*H*-1,2,3-triazol-4-yl)-1,2,6,7,8,13,14,15,16,17-decahydro-3*H*-cyclopenta[a]phenanthren-3-one (**1dd**):

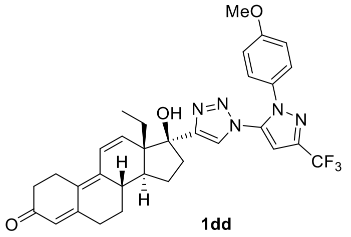

1dd

Following the general procedure, azide **5c** (425 mg, 1.5 mmol) was reacted with *gestrinone* (308 mg ,1.0 mmol). Reaction time: 16 h; FCC (SiO_2, DCM/MeOH 95:5); yellow solid, 514 mg (87%); mp 113-115 °C, $[\alpha]_D^{23}$ = +41.35 (*c* = 0.23, $CHCl_3$). ^{1}H NMR (600 MHz, $CDCl_3$) δ 7.42 (s, 1H), 7.09-7.06 (m, 2H), 6.94 (s, 1H), 6.79-6.76 (m, 2H), 6.33 (d, $^3J_{H\text{-}H}$ = 10.1 Hz, 1H), 6.18 (d, $^3J_{H\text{-}H}$ = 10.1 Hz, 1H), 5.75 (s, 1H), 3.75 (s, 3H), 3.02 (s_{br}, 1H), 2.83 (dtd, *J* ≈ 15.4, 6.7, 1.8 Hz, 1H), 2.71 (dddd, *J* = 15.4, 9.0, 6.3, 2.3 Hz, 1H), 2.61-2.39 (m, 6H), 2.23 (ddd, *J* = 14.4, 11.1, 3.3 Hz, 1H), 1.91-1.81 (m, 2H), 1.77-1.73 (m, 1H), 1.63-1.55 (m, 3H), 1.19-1.12 (m, 1H), 1.08 (t, $^3J_{H\text{-}H}$ = 7.5 Hz, 3H). $^{13}C\{^1H\}$ NMR (151 MHz, $CDCl_3$) δ 199.2, 160.6, 156.3, 154.6, 142.8 (q, $^2J_{C\text{-}F}$ = 39.5 Hz), 141.2, 139.5, 135.6, 129.6, 127.6, 125.6, 124.7, 123.9, 123.2, 120.6 (q, $^1J_{C\text{-}F}$ = 269.2 Hz, CF_3), 114.7, 102.7 (q, $^3J_{C\text{-}F}$ = 2.7 Hz), 81.5, 55.6, 51.2, 48.5, 38.2, 37.8, 36.7, 31.6, 27.0, 24.42, 24.36, 22.7, 11.4. ^{19}F NMR (565 MHz, $CDCl_3$) δ –62.73 (s, CF_3). IR (neat) *v* 3401 (OH), 2937, 1636 (C=O), 1569, 1502, 1238, 1137, 1096, 1029 cm^{-1}.HRMS (ESI-TOF) *m/z* calcd for $C_{32}H_{33}F_3N_5O_3$ ($[M+H]^+$): 592.2535; found: 592.2533.

Synthesis of 4-(1-(1-(4-methoxyphenyl)-3-(trifluoromethyl)-1*H*-pyrazol-5-yl)-1*H*-1,2,3-triazol-4-yl)-benzyl acetate (1bv): To a solution of 1,2,3-triazole **1bp** (100 mg, 0.24 mmol) in dry DCM (2.0 mL) were added Et_3N (0.25 mL), followed by acetic anhydride (0.25 mL), and the mixture was stirred at 40 °C (oil bath). After 1.5 h, the reaction was quenched with water (10 mL) and extracted with DCM (3 × 10 mL). The combined organic layers were dried over Na_2SO_4, filtered, and removed under reduced pressure. The product **1bv** was recrystallized from a mixture of DCM/hexane (1:1).

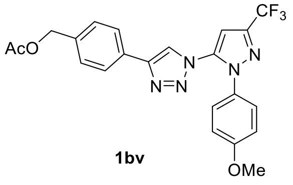

1bv

Colourless solid, 100 mg (91%); mp 87-89 °C. ^{1}H NMR (600 MHz, $CDCl_3$) δ 7.80-7.78 (m, 2H), 7.76 (s, 1H), 7.44-7.41 (m, 2H), 7.25-7.22 (m, 2H), 6.99 (s, 1H), 6.89-6.86 (m, 2H), 5.13 (s, 2H), 3.79 (s, 3H), 2.11 (s, 3H). $^{13}C\{^1H\}$ NMR (151 MHz, $CDCl_3$) δ 170.9, 160.6, 147.9, 142.8 (q, $^2J_{C\text{-}F}$ = 39.5 Hz), 137.0, 135.7, 129.7, 129.2, 129.0, 126.3, 126.0, 121.7, 120.6 (q, $^1J_{C\text{-}F}$ = 269.5 Hz), 114.9, 102.8 (q, $^3J_{C\text{-}F}$ = 2.7 Hz), 65.9, 55.7, 21.1. ^{19}F NMR (565 MHz, $CDCl_3$) δ –62.75 (s, CF_3). IR (neat) *v* 2933, 1737, 1580, 1525, 1502, 1364, 1241, 1185, 1133, 1014, 977 cm^{-1}. (+)-ESI-MS (*m/z*): 458.3 (40, $[M+H]^+$), 480.3 (100, $[M+Na]^+$). Anal. calcd. for $C_{22}H_{18}F_3N_5O_3$ (457.4): C 57.77, H 3.97, N 15.31; found: C 57.81, H 3.90, N 15.20.

Synthesis of 4-(1-(1-(4-methoxyphenyl)-3-(trifluoromethyl)-1*H*-pyrazol-5-yl)-1*H*-1,2,3-triazol-4-yl)benzaldehyde (1bw): A mixture of 1,2,3-triazole **1bp** (80 mg, 0.19 mmol), PCC (50 mg, 0.23 mmol), and Celite (5 mg) in freshly dried DCM (3.0 mL) was stirred at room temperature for 3 h (TLC monitoring). The solvent was then evaporated in vacuo. The crude product **1bw** was purified by column chromatography.

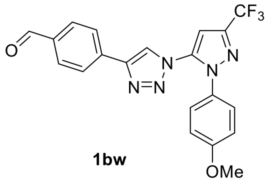

1bw

CC (SiO_2, hexane/EtOAc 2:3); yellow solid, 77 mg (98%); mp 126-128 °C. ^{1}H NMR (600 MHz, $CDCl_3$) δ 10.03 (s, 1H), 7.98-7.94 (m, 4H), 7.89 (s, 1H), 7.26-7.24 (m, 2H), 7.00 (s, 1H), 6.90-6.87 (m, 2H), 3.79 (s, 3H). ^{13}C{^{1}H} NMR (151 MHz, $CDCl_3$) δ 191.6, 160.6, 147.1, 142.9 (q, $^2J_{C-F}$ = 39.8 Hz), 136.6, 135.5, 134.9, 130.6, 129.6, 126.5, 126.1, 122.8, 120.6 (q, $^1J_{C-F}$ = 269.5 Hz), 114.9, 102.9 (q, $^3J_{C-F}$ = 2.2 Hz), 55.7. ^{19}F NMR (565 MHz, $CDCl_3$) δ –62.74 (s, CF_3). IR (neat) ν 3123, 2960, 2922, 1700, 1610, 1502, 1394, 1238, 1156, 1118, 1099, 1025, 969 cm^{-1}. (+)-ESI-MS (*m/z*): 414.3 (100, [M+H]$^+$). Anal. calcd. for $C_{20}H_{14}F_3N_5O_2$ (413.4): C 58.11, H 3.41, N 16.94; found: C 58.04, H 3.69, N 16.80.

Synthesis of methyl 4-(1-(1-(4-methoxyphenyl)-3-(trifluoromethyl)-1*H*-pyrazol-5-yl)-1*H*-1,2,3-triazol-4-yl)benzoate (1bx): To a solution of 1,2,3-triazole **1bp** (100 mg, 0.24 mmol) in MeCN (4.0 mL) were added solid Na_2CO_3 (38 mg, 0.36 mmol), followed by $KMnO_4$ (94 mg, 0.60 mmol), and the resulting mixture was stirred at 80 °C (oil bath) for 2 h. The mixture was allowed to reach room temperature and then filtered through a pad of Celite (washed with 10 mL of MeOH). The solvents were removed under reduced pressure. The residue was dissolved in MeOH (3.0 mL), concentrated H_2SO_4 (0.30 mL) was added, and the mixture was heated at 80 °C for 12 h. The solvent was then evaporated, water (10 mL) was added, and the mixture was extracted with DCM (3 × 10 mL). The combined organic layers were dried over Na_2SO_4, filtered, and concentrated in vacuo. The crude product **1bx** was purified by column chromatography.

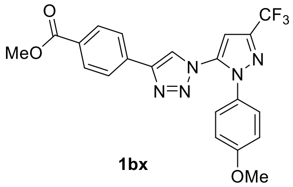

1bx

CC (SiO_2, DCM/MeOH 9:1); yellow solid, 99 mg (93%); mp 155-157 °C. ^{1}H NMR (600 MHz, $CDCl_3$) δ 8.11-8.08 (m, 2H), 7.87-7.85 (m, 3H), 7.25-7.23 (m, 2H), 6.99 (s, 1H), 6.89-6.87 (m, 2H), 3.93 (s, 3H), 3.79 (s, 3H). ^{13}C{^{1}H} NMR (151 MHz, $CDCl_3$) δ 166.6, 160.6, 147.4, 142.9 (q, $^2J_{C-F}$ = 39.5 Hz), 135.6, 133.5, 130.6, 130.5, 129.6, 126.1, 125.9, 122.5, 120.6 (q, $^1J_{C-F}$ = 269.2 Hz), 114.9, 102.9 (q, $^3J_{C-F}$ = 2.2 Hz), 55.7, 52.4. ^{19}F NMR (565 MHz, $CDCl_3$) δ –62.74 (s, CF_3). IR (neat) ν 3112, 2952, 2922, 2848, 1707, 1502, 1439, 1286, 1241, 1178, 1129, 1103, 1014, 969 cm^{-1}. (+)-ESI-MS (*m/z*): 444.2 (32, [M+H]$^+$), 466.2 (100, [M+Na]$^+$). Anal. calcd. for $C_{21}H_{16}F_3N_5O_3$ (443.4): C 56.89, H 3.64, N 15.80; found: C 56.85, H 3.84, N 15.70.

Synthesis of 4-(1-(1-(4-methoxyphenyl)-3-(trifluoromethyl)-1*H*-pyrazol-5-yl)-1*H*-1,2,3-triazol-4-yl)benzamide (1by): To a solution of 1,2,3-triazole **1bf** (80 mg, 0.20 mmol) in acetone (2.0 mL) were added an aqueous solution of Na_2CO_3 (3 M, 1.0 mL) and H_2O_2 (30%, 1.0 mL), and the mixture was stirred at room temperature. After 2 h, the precipitated **1by** was filtered off and recrystallized from MeCN.

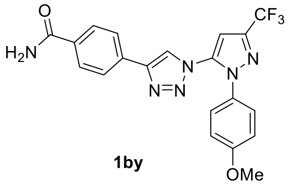

1by

Colourless solid, 81 mg (94%); mp 237-239 °C. ^{1}H NMR (600 MHz, DMSO-d_6) δ 9.14 (s, 1H), 8.04 (s$_{br}$, 1H), 8.00-7.97 (m, 2H), 7.94-7.90 (m, 2H), 7.64 (s, 1H), 7.41 (s$_{br}$, 1H), 7.35-7.30 (m, 2H), 7.02-6.98 (m, 2H), 3.76 (s, 3H). ^{13}C{^{1}H} NMR (151 MHz, DMSO-d_6) δ 167.3, 159.9, 146.0, 140.9 (q, $^2J_{C\text{-}F}$ = 38.4 Hz), 136.0, 134.2, 131.8, 129.4, 128.4, 126.3, 125.4, 125.1, 120.8 (q, $^1J_{C\text{-}F}$ = 269.2 Hz), 114.6, 103.7 (br), 55.5. ^{19}F NMR (565 MHz, DMSO-d_6) δ –61.28 (s, CF_3). IR (neat) ν 3418 (N-H), 3135 (N-H), 2931, 1651 (C=O), 1617, 1502, 1390, 1241, 1156, 1126, 1103, 1021, 969 cm^{-1}. (–)-ESI-MS (*m/z*): 400 (92), 427.0 (100, [M–H]$^-$). HRMS (ESI-TOF) *m/z* calcd for $C_{20}H_{16}F_3N_6O_2$ ([M+H]$^+$): 429.1287; found: 429.1295.

Synthesis of 1-(1-(4-Methoxyphenyl)-3-(trifluoromethyl)-1*H*-pyrazol-5-yl)-4-(*p*-tolyl)-1*H*-1,2,3-triazole (1bc) by hydrogenation of pyrazole-triazole hybrid 1bf: To a solution of 1,2,3-triazole **1bf** (80 mg, 0.2 mmol) in dry THF (5.0 mL) was added 10% Pd/C (200 mg), and the resulting mixture was hydrogenated (H_2, 70 psi) at room temperature for 5 h. The mixture was filtered through a short pad of Celite (washed with 5.0 mL of EtOAc), and the solvents were removed under reduced pressure to give spectroscopically pure **1bc** (79 mg, 99%).

Synthesis of *N*-(4-(1-(1-(4-methoxyphenyl)-3-(trifluoromethyl)-1*H*-pyrazol-5-yl)-1*H*-1,2,3-triazol-4-yl)-benzyl)isobutyramide (1bz): To 1,2,3-triazole **1bf** (100 mg, 0.24 mmol) in MeOH (6.0 mL) were added freshly prepared Raney-Ni (400 mg), followed by aqueous solution of NH_3 (25%, 1.0 mL), and mixture was hydrogenated (H_2, positive pressure from a balloon) at room temperature for 2 h. The reaction mixture was filtered through a pad of Celite (washed with 5 mL of MeOH), and the solvents were removed in vacuo. The residue was dissolved in dry THF (4.0 mL), Et_3N (0.10 mL) was added, and the mixture was stirred at 0 °C for 10 min. Next, isobutyryl chloride (0.10 mL, 0.48 mmol) was added dropwise, and the mixture was stirred at room temperature for 1 h. The reaction was quenched with water (20 mL) and extracted with DCM (3 × 10 mL). The combined organic layers were dried over anhydrous Na_2SO_4, filtered, and concentrated in vacuo. The crude product **1bz** was purified by column chromatography.

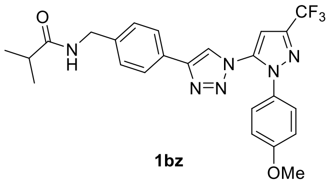

1bz

CC (SiO_2, hexane/EtOAc 1:1); colourless solid, 86 mg (74%); mp 160-162 °C. ^{1}H NMR (600 MHz, $CDCl_3$) δ 7.76-7.73 (m, 3H), 7.34-7.32 (m, 2H), 7.24-7.21 (m, 2H), 6.98 (s, 1H), 6.88-6.85 (m, 2H), 5.82 (t, $^3J_{H\text{-}H}$ = 5.9 Hz, 1H), 4.46 (d, $^3J_{H\text{-}H}$ = 5.9 Hz, 1H), 3.79 (s, 3H), 2.40 (hept, $^3J_{H\text{-}H}$ = 6.9 Hz,

1H), 1.19 (d, J = 6.9 Hz, 6H). $^{13}C\{^1H\}$ NMR (151 MHz, $CDCl_3$) δ 177.0, 160.6, 148.0, 142.8 (q, $^2J_{C-F}$ = 39.8 Hz), 139.7, 135.8, 129.7, 128.4*,126.4, 126.0, 121.5, 120.6 (q, $^1J_{C-F}$ = 269.8 Hz), 114.8, 102.8 (q, $^3J_{C-F}$ = 2.7 Hz), 55.7, 43.2, 35.8, 19.8; *higher intensity. ^{19}F NMR (565 MHz, $CDCl_3$) δ –62.74 (s, CF_3). IR (neat) ν 3351 (N-H), 2967, 2933, 1644 (C=O), 1588, 1525, 1495, 1375, 1238, 1156, 1126, 1021, 973 cm^{-1}. (+)-ESI-MS (m/z): 485.3 (100, $[M+H]^+$). Anal. calcd. for $C_{24}H_{23}F_3N_6O_2$ (484.5): C 59.50, H 4.79, N 17.35; found: C 59.52, H 4.89, N 17.12.

General procedure for synthesis of azides 5: To a solution of 1-aryl-3-(trifluoromethyl)pyrazole **3** (1.00 mmol) in anhydrous THF (10 mL), at –78 °C, under argon, *n*-BuLi (2.5M in hexane, 0.52 mL, 1.30 mmol) was added. After 5 min, a solution of tosyl azide (405 mg, 2.05 mmol) in dry THF (5 mL) was added dropwise. The reaction mixture was allowed to warm to room temperature and stirred for 4 h. The reaction was quenched with 1M NH_4Cl (15 mL) and extracted with DCM (3 × 20 mL). The combined organic layers were washed with water (3 × 10 mL), dried over Na_2SO_4, filtered, and the solvents were removed in vacuo. The crude product **5** was purified by standard column chromatography on silica gel (CC).

5-Azido-1-(p-tolyl)-3-(trifluoromethyl)-1H-pyrazole (**5a**):

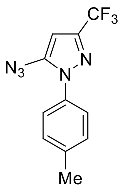

5a

CC (SiO_2, hexane/DCM 4:1); red solid, 222 mg (83%); mp 45-47 °C. ^1H NMR (600 MHz, $CDCl_3$) δ 7.48-7.45 (m, 2H), 7.29-7.27 (m, 2H), 6.45 (s, 1H), 2.41 (s, 3H). $^{13}C\{^1H\}$ NMR (151 MHz, $CDCl_3$) δ 142.9 (q, $^2J_{C-F}$ = 38.7 Hz), 139.4, 139.0, 134.9, 129.8, 124.0, 120.9 (q, $^1J_{C-F}$ = 268.7 Hz), 94.1 (q, $^3J_{C-F}$ = 2.5 Hz), 21.3. ^{19}F NMR (565 MHz, $CDCl_3$) δ –63.02 (s, CF_3). IR (neat) ν 2922, 2136 (N_3), 1517, 1472, 1282, 1233, 1162, 1107, 972, 816 cm^{-1}. (+)-ESI-MS (m/z): 268.3 (100, $[M+H]^+$). Anal. calcd. for $C_{11}H_8F_3N_5$ (267.2): C 49.44, H 3.02, N 26.21; found: C 49.40, H 3.02, N 26.19.

5-Azido-1-(4-isopropylphenyl)-3-(trifluoromethyl)-1H-pyrazole (**5b**):

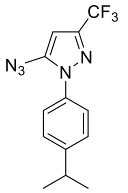

5b

CC (SiO_2, hexane/DCM 4:1), orange oil, 236 mg (80%). ^1H NMR (600 MHz, $CDCl_3$) δ 7.52-7.49 (m, 2H), 7.35-7.32 (m, 2H), 6.46 (s, 1H), 2.98 (hept, $^3J_{H-H}$ = 7.0 Hz, 1H), 1.29 (d, $^3J_{H-H}$ = 7.0 Hz, 6H). $^{13}C\{^1H\}$ NMR (151 MHz, $CDCl_3$) δ 149.8, 142.8 (q, $^2J_{C-F}$ = 39.2 Hz), 139.4, 135.1, 127.2, 124.0, 120.9 (q, $^1J_{C-F}$ = 269.2Hz), 94.1 (q, $^3J_{C-F}$ =2.2 Hz), 34.0, 24.0. ^{19}F NMR (565 MHz, $CDCl_3$) δ –63.02 (s, CF_3). IR (neat) ν 2963, 2132 (N_3), 1517, 1487, 1394, 1282, 1249, 1170, 1133, 973 cm^{-1}. (+)-ESI-MS (m/z): 296.3 (100, $[M+H]^+$),

318.2 (65, $[M+Na]^+$). Anal. calcd. for $C_{13}H_{12}F_3N_5$ (295.3): C 52.88, H 4.10, N 23.72; found: C 52.95, H 3.99, N 23.80.

5-Azido-1-(4-methoxyphenyl)-3-(trifluoromethyl)-1*H*-pyrazole (**5c**):

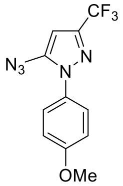

5c CC (SiO_2, hexane/DCM 4:1); orange solid, 266 mg (94%); mp 76-77 °C. ^{1}H NMR (600 MHz, $CDCl_3$) δ 7.49-7.47 (m, 2H), 6.99-6.96 (m, 2H), 6.44 (s, 1H), 3.85 (s, 3H). $^{13}C\{^{1}H\}$ NMR (151 MHz, $CDCl_3$) δ 159.9, 142.7 (q, $^{2}J_{C\text{-}F}$ = 39.2 Hz), 139.4, 130.4, 125.7, 120.9 (q, $^{1}J_{C\text{-}F}$ = 269.2 Hz), 114.4, 94.0 (q, $^{3}J_{C\text{-}F}$ = 2.5 Hz), 55.7. ^{19}F NMR (565 MHz, $CDCl_3$) δ –63.00 (s, CF_3). IR (neat) ν 2922, 2140 (N_3), 1513, 1469, 1223, 1170, 1118, 1092, 1021, 969 cm^{-1}. (+)-ESI-MS (m/z): 284.3 (100, $[M+H]^+$). Anal. calcd. for $C_{11}H_8F_3N_5O$ (283.2): C 46.65, H 2.85, N 24.73; found: C 46.66, H 2.81, N 24.76.

5-Azido-1-(4-(benzyloxy)phenyl)-3-(trifluoromethyl)-1*H*-pyrazole (**5d**):

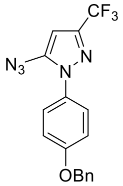

5d CC (SiO_2, hexane/DCM 1:1); red solid, 305 mg (85%); mp 61-63 °C. ^{1}H NMR (600 MHz, $CDCl_3$) δ 7.52-7.48 (m, 2H), 7.47-7.45 (m, 2H), 7.43-7.40 (m, 2H), 7.38-7.35 (m, 1H), 7.08-7.05 (m, 2H), 6.45 (s, 1H), 5.12 (s, 2H). $^{13}C\{^{1}H\}$ NMR (151 MHz, $CDCl_3$) δ 159.0, 142.7 (q, $^{2}J_{C\text{-}F}$ = 39.2 Hz), 139.4, 136.5, 130.6, 128.8, 128.3, 127.6, 125.6, 120.9 (q, $^{1}J_{C\text{-}F}$ =269.2 Hz), 115.3, 94.0 (q, $^{3}J_{C\text{-}F}$ =2.2 Hz), 70.5. ^{19}F NMR (565 MHz, $CDCl_3$) δ –62.96 (s, CF_3). IR (neat) ν 2928, 2132 (N_3), 1595, 1510, 1469, 1379, 1286, 1234, 1170, 1122, 998, 969 cm^{-1}. (+)-ESI-MS (m/z): 360.3 (100, $[M+H]^+$), 382.2 (23, $[M+Na]^+$). Anal. calcd. for $C_{17}H_{12}F_3N_5O$ (359.3): C 56.83, H 3.37, N 19.49; found: C 56.82, H 3.31, N 19.49.

5-Azido-1-(4-chlorophenyl)-3-(trifluoromethyl)-1*H*-pyrazole (**5e**):

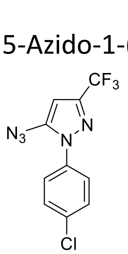

5e CC (SiO_2, hexane/DCM 4:1); orange solid, 173 mg (60%); mp 45-47 °C. ^{1}H NMR (600 MHz, $CDCl_3$) δ 7.60-7.57 (m, 2H), 7.46-7.43 (m, 2H), 6.47 (s, 1H). $^{13}C\{^{1}H\}$ NMR (151 MHz, $CDCl_3$) δ 143.3 (q, $^{2}J_{C\text{-}F}$

= 39.2 Hz), 139.7, 136.0, 134.5, 129.4, 125.0, 120.7 (q, $^{1}J_{C-F}$ = 269.2 Hz), 94.5 (q, $^{3}J_{C-F}$ = 2.5 Hz). ^{19}F NMR (565 MHz, $CDCl_3$) δ –63.18 (s, CF_3). IR (neat) ν 2925, 2136 (N_3), 1469, 1394, 1279, 1245, 1174, 1133, 1092, 1006, 972 cm^{-1}. (+)-ESI-MS (m/z): 288.2 (100, [M+H]$^+$). Anal. calcd. for $C_{10}H_5ClF_3N_5$ (287.6): C 41.76, H 1.75, N 24.35; found: C 41.97, H 1.88, N 24.40.

5-Azido-3-(trifluoromethyl)-1-(4-(trifluoromethyl)phenyl)-1*H*-pyrazole (**5f**):

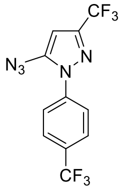

5f

CC (SiO_2, hexane/DCM 3:2); orange solid, 257 mg (80%); mp 52-54 °C. ^{1}H NMR (600 MHz, $CDCl_3$) δ 7.84-7.81 (m, 2H), 7.75-7.72 (m, 2H), 6.50 (s, 1H). $^{13}C\{^1H\}$ NMR (151 MHz, $CDCl_3$) δ 143.8 (q, $^{2}J_{C-F}$ = 39.2Hz), 140.2, 140.1, 130.4 (q, $^{2}J_{C-F}$ = 33.2 Hz), 126.5 (q, $^{3}J_{C-F}$ = 3.5 Hz), 123.8 (q, $^{1}J_{C-F}$ = 272.5 Hz), 123.6, 120.6 (q, $^{1}J_{C-F}$ = 269.2 Hz), 94.9 (q, $^{3}J_{C-F}$ = 2.5 Hz). ^{19}F NMR (565 MHz, $CDCl_3$) δ –62.72 (s, CF_3), –63.36 (s, CF_3). IR (neat) ν 2932, 2132 (N_3), 1618, 1491, 1394, 1323, 1260, 1170, 1103, 1066, 969 cm^{-1}. (+)-ESI-MS (m/z): 322.2 (100, [M+H]$^+$), 344.1 (19, [M+Na]$^+$). Anal. calcd. for $C_{11}H_5F_6N_5$ (321.0): C 41.14, H 1.57, N 21.81; found: C 41.22, H 1.64, N 21.87.

4-(5-Azido-3-(trifluoromethyl)-1*H*-pyrazol-1-yl)benzonitrile (**5g**):

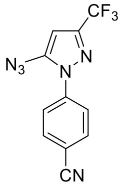

5g

CC (SiO_2, hexane/DCM 4:1); red oil, 103 mg (37%, ca. 90% purity). ^{1}H NMR (600 MHz, $CDCl_3$) δ 7.89-7.86 (m, 2H), 7.78-7.75 (m, 2H), 6.51 (s, 1H). $^{13}C\{^1H\}$ NMR (151 MHz, $CDCl_3$) δ 144.1 (q, $^{2}J_{C-F}$ = 39.2 Hz), 140.8, 140.3, 133.3, 123.5, 122.3 (q, $^{1}J_{C-F}$ = 282.3 Hz), 118.0, 111.9, 95.2 (q, $^{3}J_{C-F}$ = 2.7 Hz). ^{19}F NMR (565 MHz, $CDCl_3$) δ –63.39 (s, CF_3). IR (neat) ν 2950, 2933, 2229 (CN), 2140 (N_3), 1607, 1513, 1487, 1394, 1249, 1170, 1133, 1100 cm^{-1}. HRMS (ESI-TOF) *m/z* calcd for $C_{11}H_5F_3N_6Na$ ([M+Na]$^+$): 301.0425; found: 301.0417.

Synthesis of 4-azido-1-(*p*-tolyl)-3-(trifluoromethyl)-1*H*-pyrazole (8): Following a general literature protocol,[5] a solution of 1-aryl-3-CF_3-pyrazole **3a** (226 mg, 1.0 mmol), ceric ammonium nitrate (CAN, 603 mg, 1.1 mmol), and elemental iodine (330 mg, 1.3 mmol) in MeCN (6.0 mL) was refluxed (oil bath) overnight. After removal of the solvent in vacuo, the residue was dissolved in DCM (15 mL). The resulting solution was washed with sat. aq. $Na_2S_2O_3$ (5.0 mL), followed by water (10 mL), and the organic layer was dried over Na_2SO_4. Evaporation of the solvent afforded a crude product, which was purified by filtration through a short silica gel pad (SiO_2, hexane/DCM 3:2) to give iodide **7** (306 mg, 87%) as a yellow oil (^{1}H NMR (600 MHz, $CDCl_3$) δ 7.98 (s, 1H), 7.55-7.53 (m, 2H), 7.32-7.29 (m, 2H), 2.42 (s, 3H); data in accordance with the literature[5]). To a solution of this material (**7**, 306 mg, 0.87 mmol) in anhydrous THF (10 mL), *n*-BuLi (2.5M in hexane, 0.452 mL, 1.13 mmol) was added dropwise at −78 °C under an argon atmosphere. After 5 min, a solution of tosyl azide (256 mg, 1.3 mmol) in dry THF (4 mL) was added. The reaction mixture was allowed to reach room temperature, and stirring was continued for 4 h. The reaction was then quenched with 1M NH_4Cl (10 mL), and the mixture was extracted with DCM (3 × 15 mL). The combined organic layers were washed with water (3 × 10 mL), dried over Na_2SO_4, filtered, and concentrated in vacuo. The crude product **8** was purified by standard column chromatography.

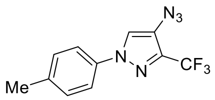

8

CC (SiO_2, hexane/DCM 4:1); thick orange oil, 128 mg (42%). ^{1}H NMR (600 MHz, $CDCl_3$) δ 7.82 (s, 1H), 7.54-7.52 (m, 2H), 7.29-7.27 (m, 2H), 2.40 (s, 3H). $^{13}C\{^1H\}$ NMR (151 MHz, $CDCl_3$) δ 138.4, 136.9, 134.7 (q, $^2J_{C-F}$ = 38.4 Hz), 130.3, 123.1, 120.8 (q, $^1J_{C-F}$ = 269.5 Hz), 120.1, 119.6, 21.1. ^{19}F NMR (565 MHz, $CDCl_3$) δ −61.07 (s, CF_3). IR (neat) ν 2930, 2125 (N_3), 1513, 1402, 1301, 1238, 1167, 1126, 1051, 962 cm^{-1}. (+)-ESI-MS (m/z): 268.2 (100, $[M+H]^+$); HRMS (ESI-TOF) *m/z* calcd. for $C_{11}H_9F_3N_5$ ($[M+H]^+$): 268.0810; found: 268.0812.

Synthesis of 4-phenyl-1-(1-(*p*-tolyl)-3-(trifluoromethyl)-1*H*-pyrazol-4-yl)-1*H*-1,2,3-triazole (9): A mixture of azide **8** (107 mg, 0.40 mmol), phenylacetylene (61 mg, 0.60 mmol), copper(II) sulfate pentahydrate (15.0 mg, 0.06 mmol), and (+)-sodium L-ascorbate (23.8 mg, 0.12 mmol) in MeOH/H_2O (10:1, 6 mL) was stirred at 55 °C (oil bath) for 5 h. The solvents were evaporated, and the crude reaction mixture was dissolved in DCM (8 mL), dried over Na_2SO_4, and filtered through a Celite pad (washed with DCM). After evaporation of the solvent, the product **9** was purified by standard column chromatography (SiO_2, hexane/AcOEt 9:1) and recrystallized from hexane.

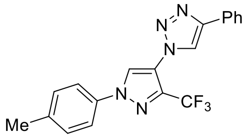

9

Yellow solid, 99 mg (68%); mp 117-119 °C. ^{1}H NMR (600 MHz, $CDCl_3$) δ 7.78-7.80 (m, 2H), 7.77 (s, 1H), 7.45-7.42 (m, 2H), 7.39-7.36 (m, 1H), 7.19-7.17 (m, 4H), 6.99 (s, 1H), 2.35 (s, 3H). $^{13}C\{^1H\}$ NMR (151 MHz, $CDCl_3$) δ 148.4, 142.9 (q, $^2J_{C-F}$ = 39.8 Hz), 140.1, 135.7, 134.4, 130.3, 129.3, 129.2,

129.1, 126.1, 124.2, 121.7, 120.6 (q, $^1J_{C\text{-}F}$ = 269.5 Hz), 103.0 (q, $^3J_{C\text{-}F}$ = 2.5 Hz), 21.3. ^{19}F NMR (565 MHz, $CDCl_3$) δ –62.74 (s, CF_3). IR (neat) v 3153, 2925, 1580, 1502, 1364, 1238, 1156, 1133, 1014, 977 cm^{-1}. (+)-ESI-MS (*m*/*z*): 370.2 (12, $[M+H]^+$), 392.4 (100, $[M+Na]^+$). HRMS (ESI-TOF) *m*/*z* calcd. for $C_{19}H_{15}F_3N_5$ ($[M+H]^+$): 370.1280; found: 370.1288.

3. Copies of NMR spectra

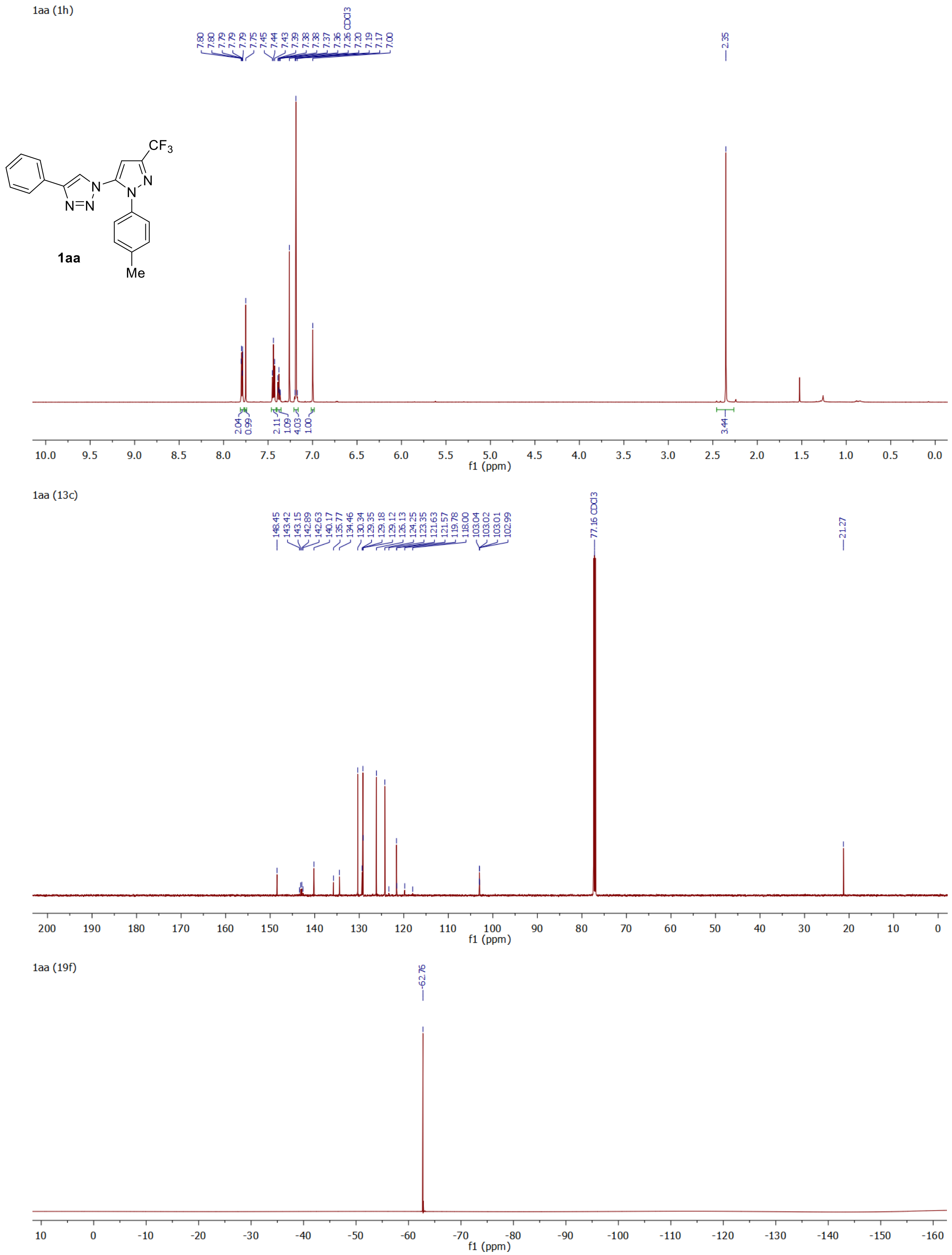

Fig S1. ^{1}H NMR (600 MHz, $CDCl_3$), $^{13}C\{^{1}H\}$ NMR (151 MHz, $CDCl_3$) and ^{19}F NMR (565 MHz, $CDCl_3$) spectra for compound **1aa**.

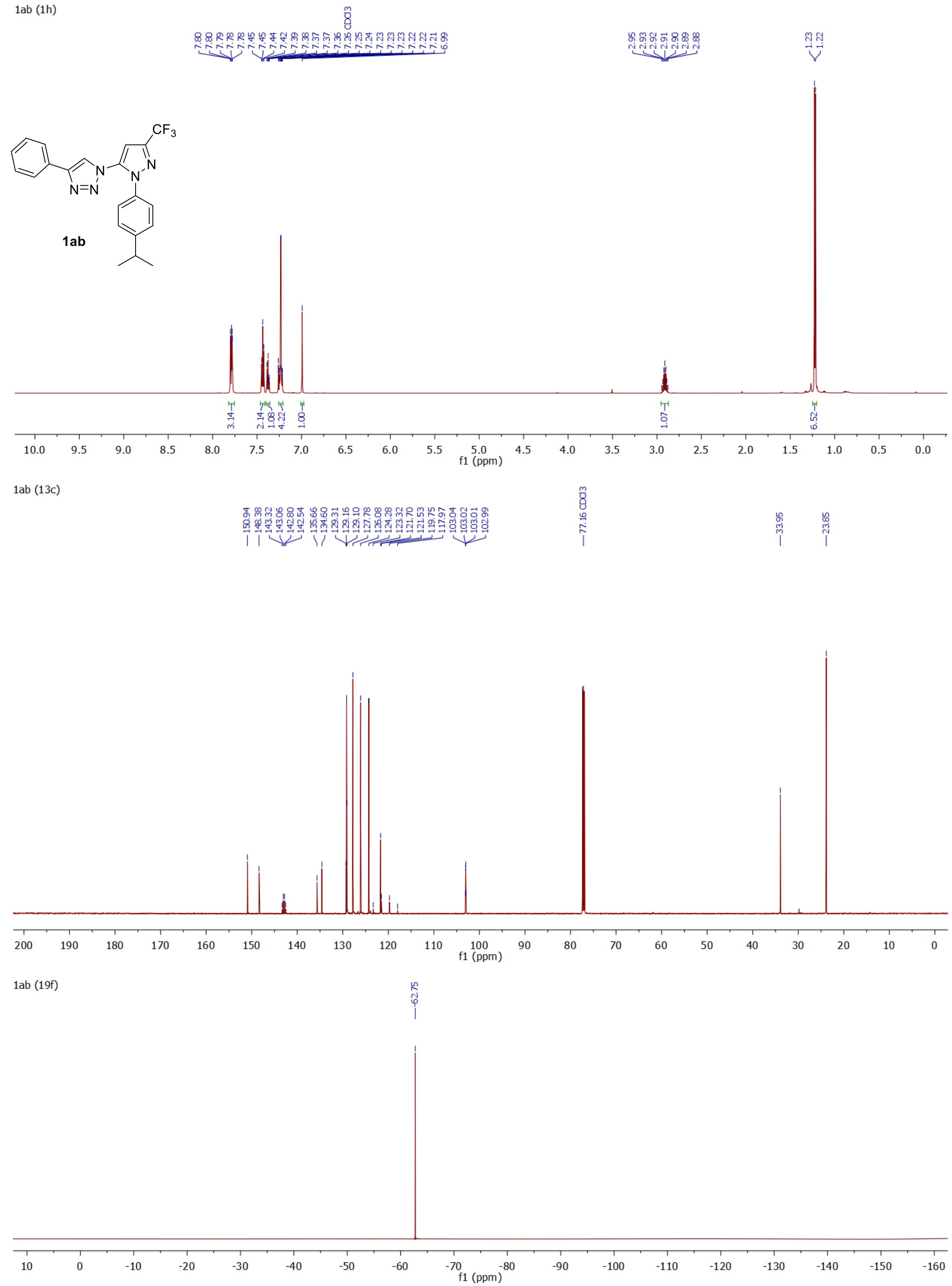

Fig S2. ^{1}H NMR (600 MHz, $CDCl_3$), $^{13}C\{^{1}H\}$ NMR (151 MHz, $CDCl_3$) and ^{19}F NMR (565 MHz, $CDCl_3$) spectra for compound **1ab**.

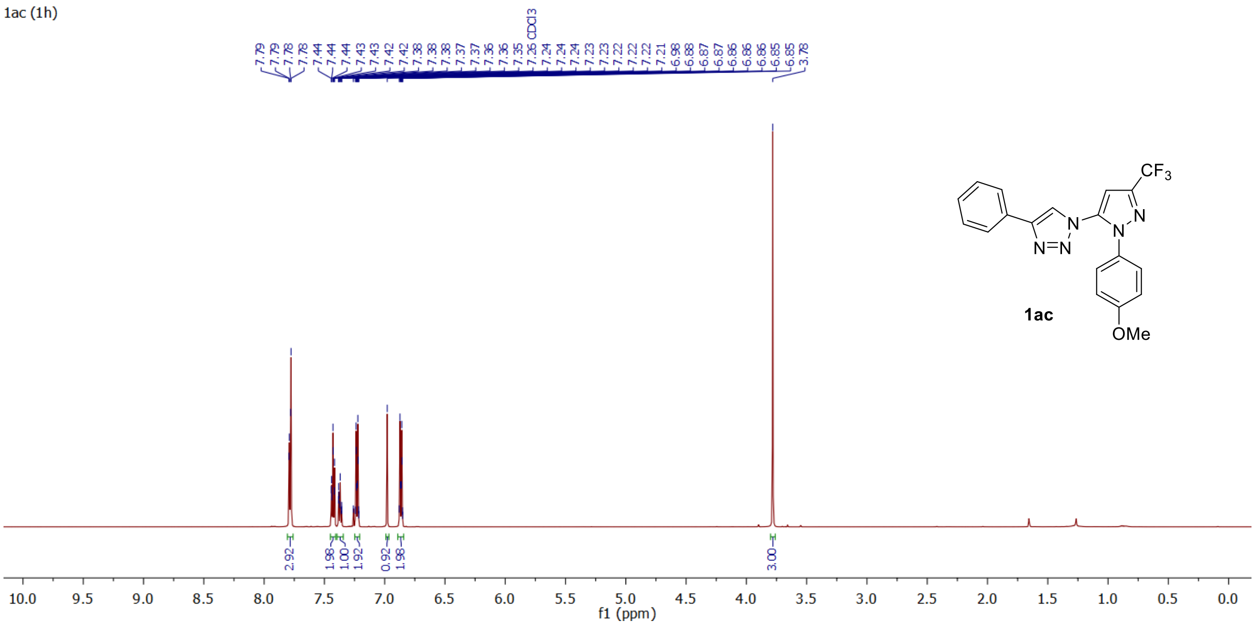

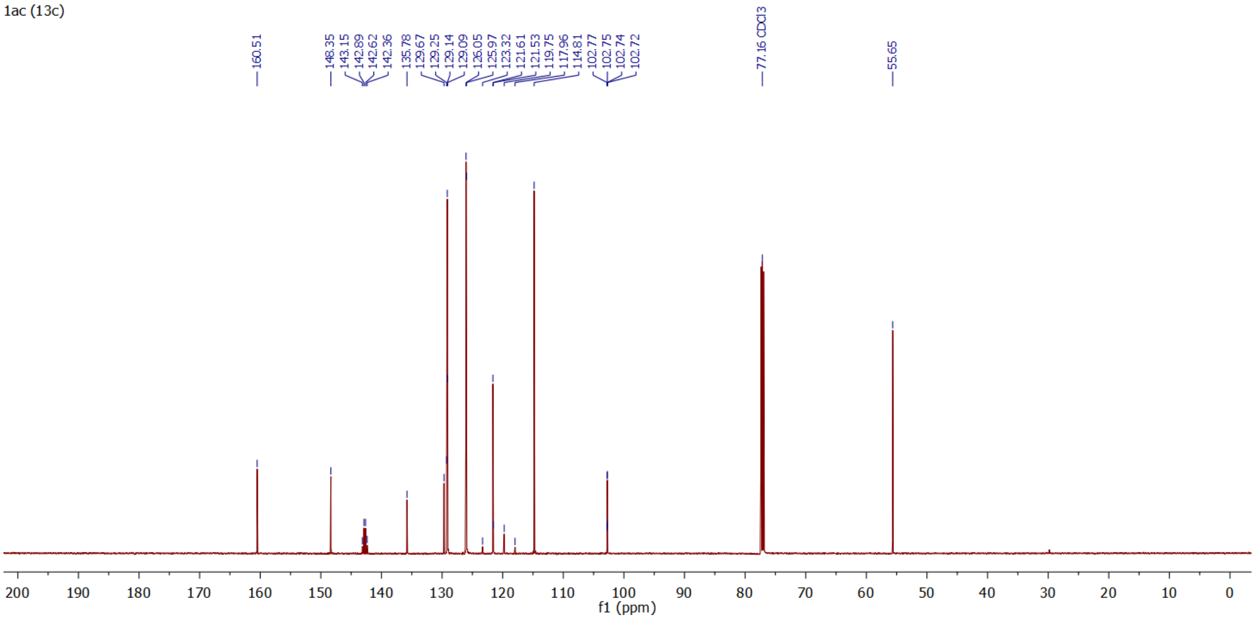

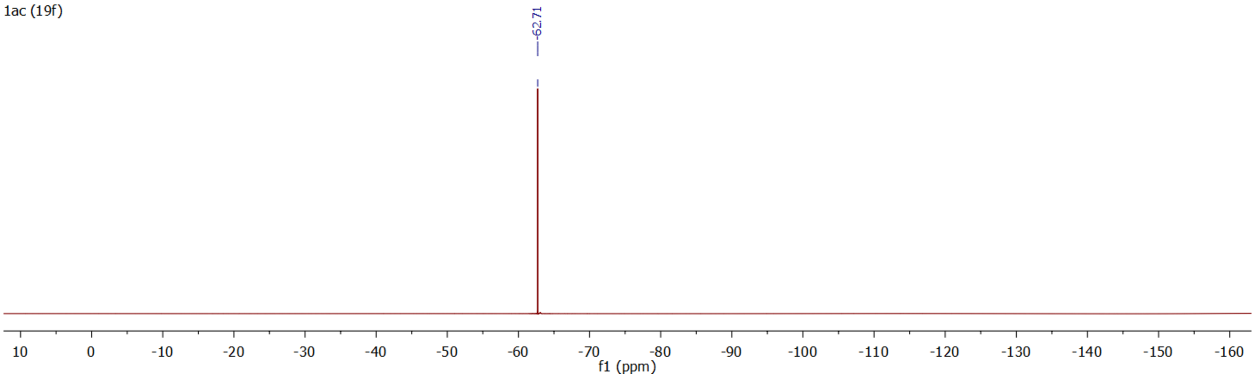

Fig S3. ^{1}H NMR (600 MHz, $CDCl_3$), $^{13}C\{^{1}H\}$ NMR (151 MHz, $CDCl_3$) and ^{19}F NMR (565 MHz, $CDCl_3$) spectra for compound **1ac**.

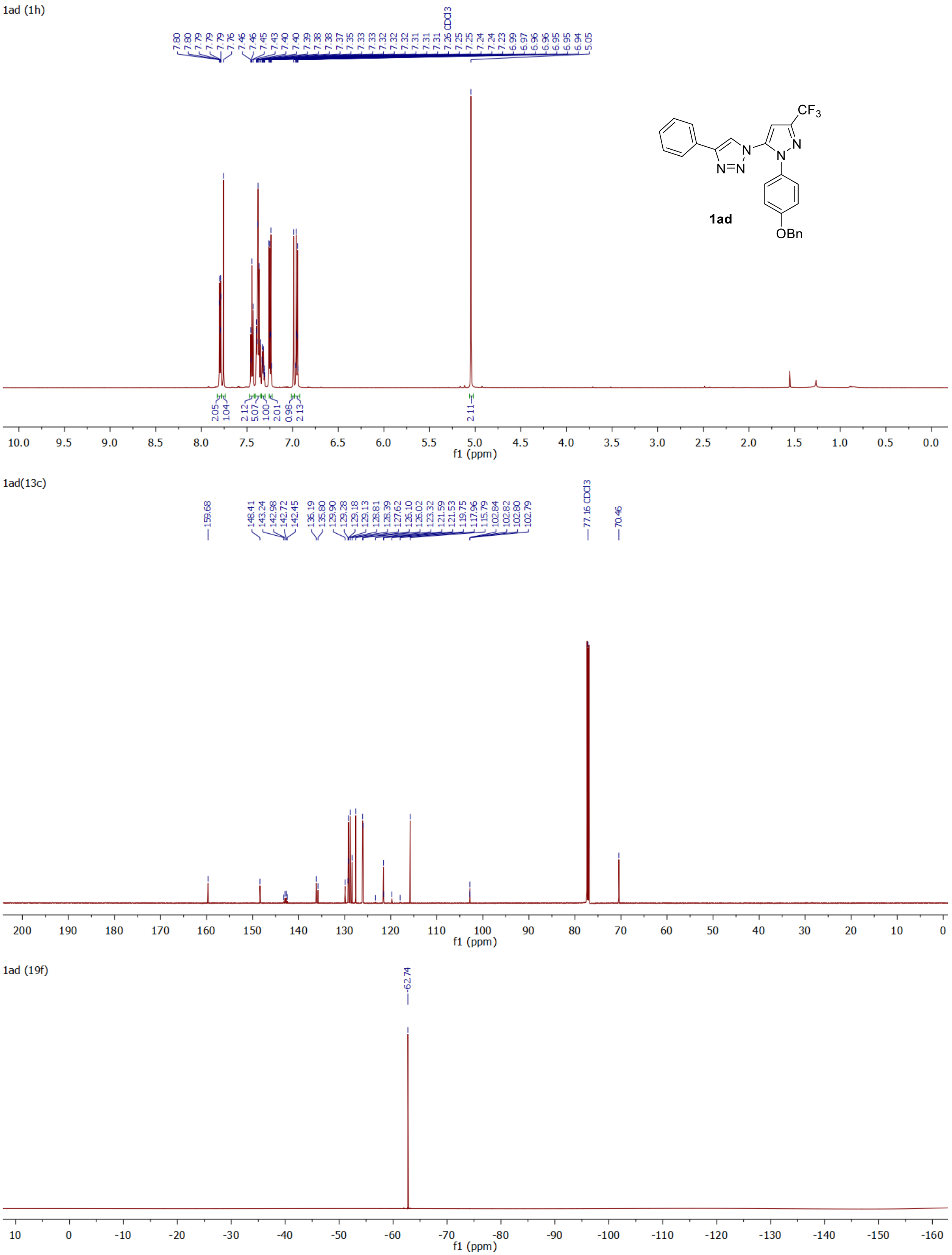

Fig S4. ^{1}H NMR (600 MHz, $CDCl_3$), $^{13}C\{^{1}H\}$ NMR (151 MHz, $CDCl_3$) and ^{19}F NMR (565 MHz, $CDCl_3$) spectra for compound **1ad**.

1ae (1h)

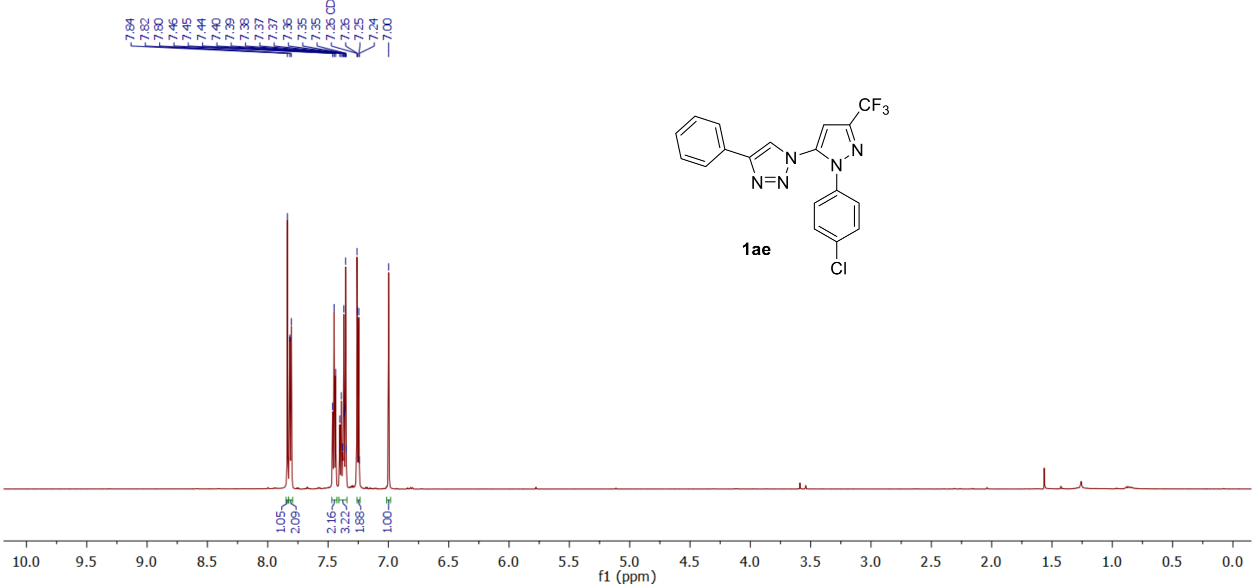

1ae (13c)

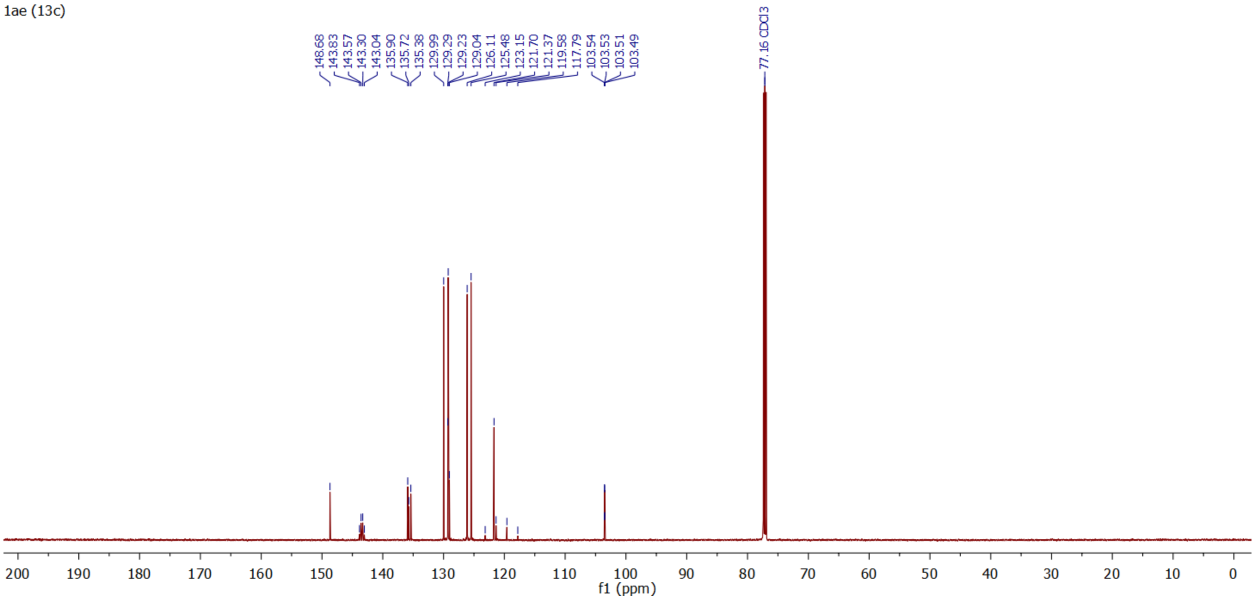

1ae (19f)

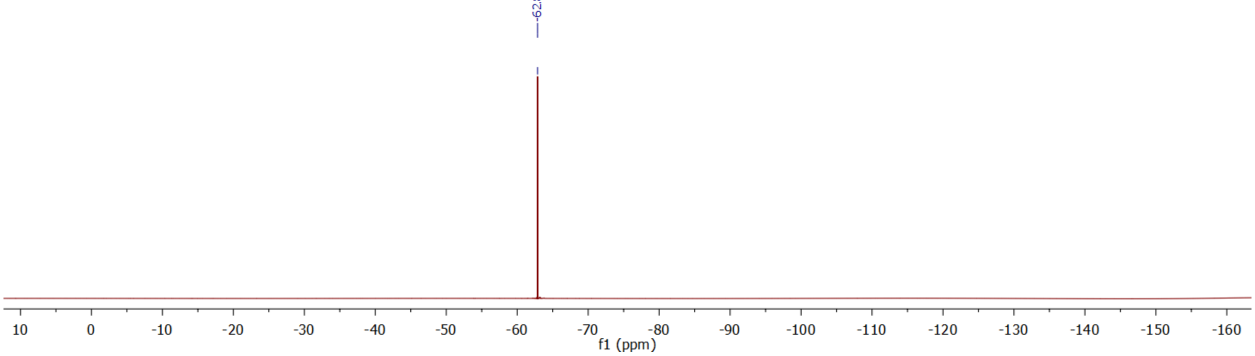

Fig S5. ^{1}H NMR (600 MHz, $CDCl_3$), $^{13}C\{^{1}H\}$ NMR (151 MHz, $CDCl_3$) and ^{19}F NMR (565 MHz, $CDCl_3$) spectra for compound **1ae**.

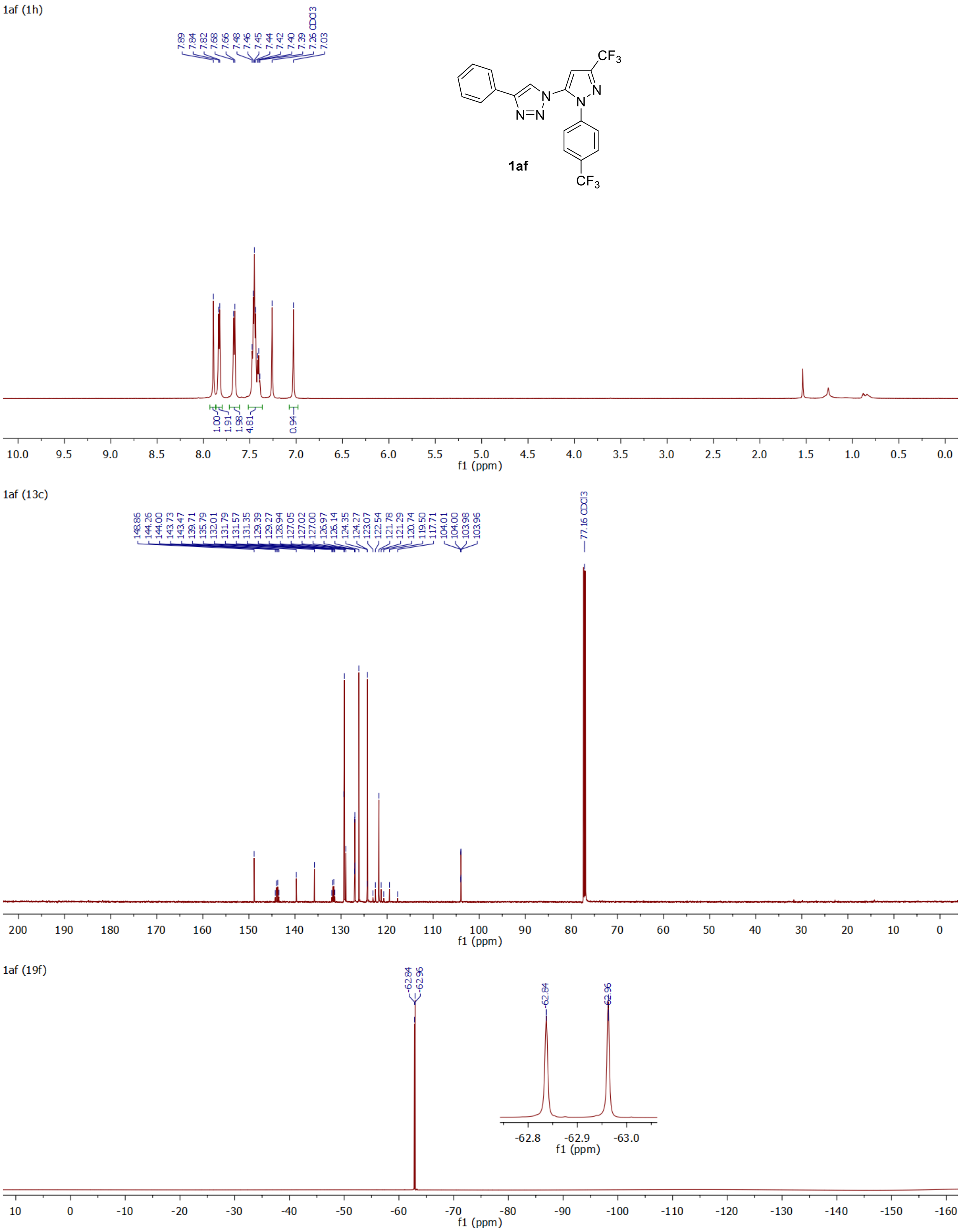

Fig S6. ^{1}H NMR (600 MHz, $CDCl_3$), $^{13}C\{^{1}H\}$ NMR (151 MHz, $CDCl_3$), and ^{19}F NMR (565 MHz, $CDCl_3$) spectra for compound **1ef**.

1ag (1h)

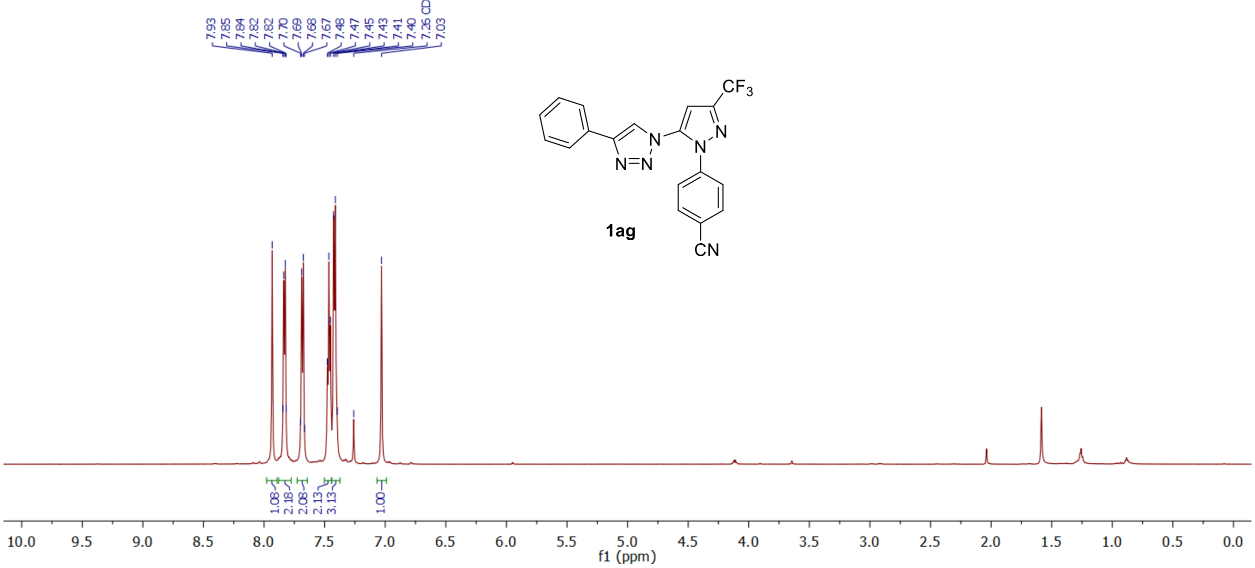

1ag (13c)

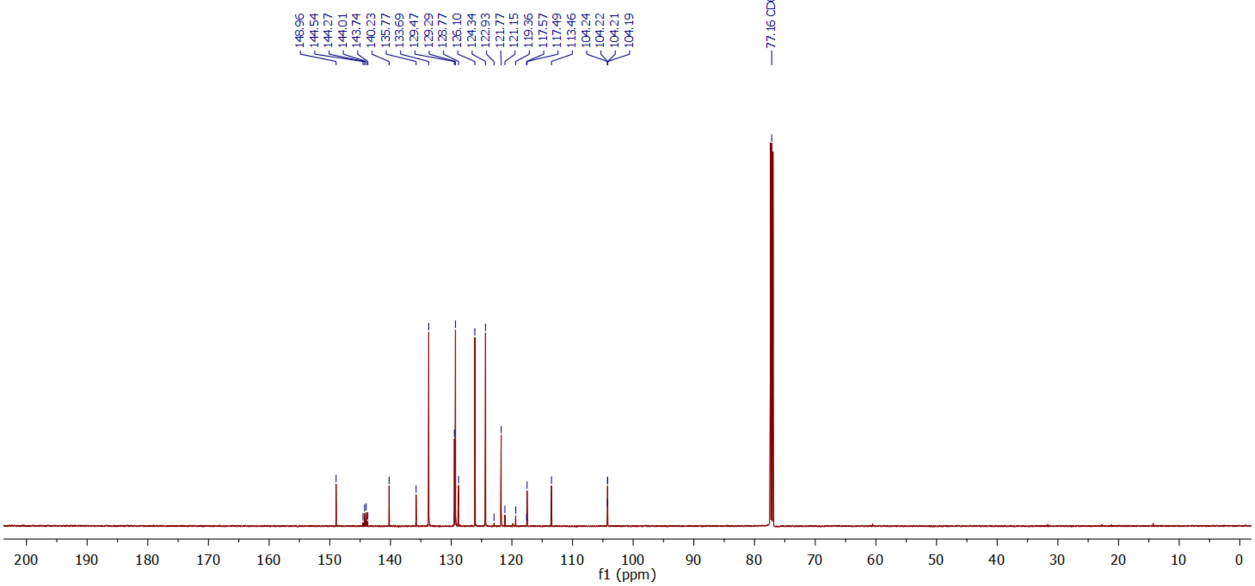

1ag (19f)

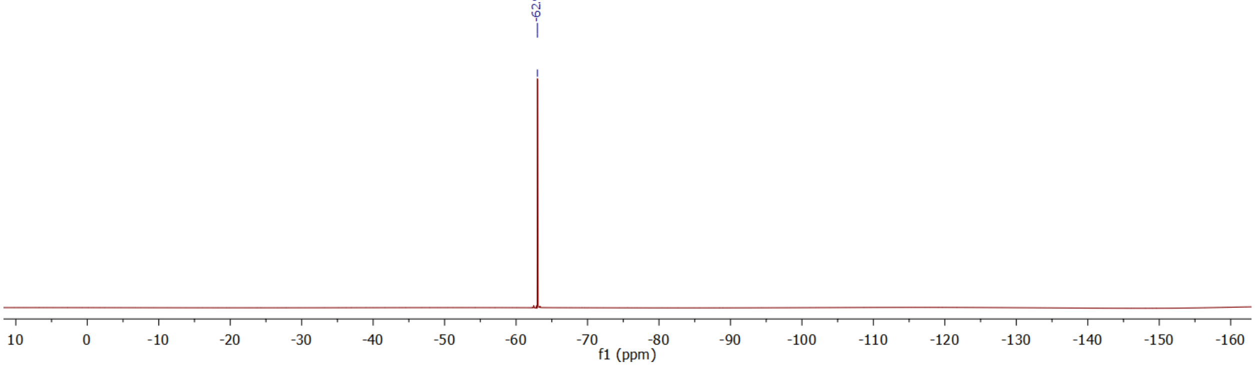

Fig S7. ^{1}H NMR (600 MHz, $CDCl_3$), $^{13}C\{^{1}H\}$ NMR (151 MHz, $CDCl_3$) and ^{19}F NMR (565 MHz, $CDCl_3$) spectra for compound **1ag**.

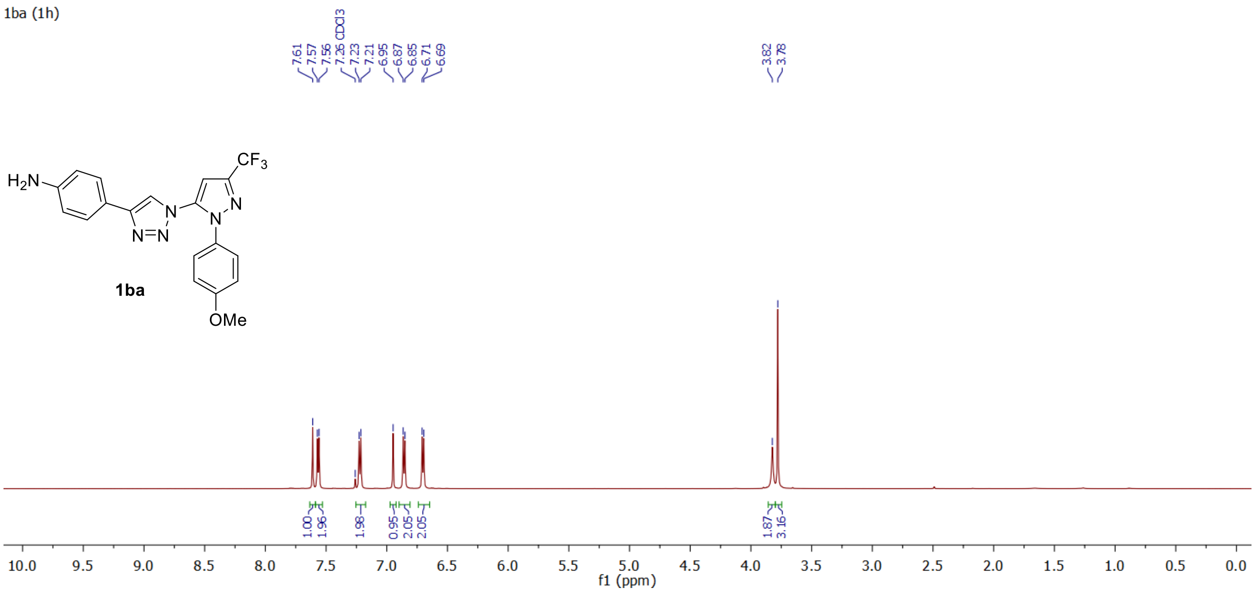

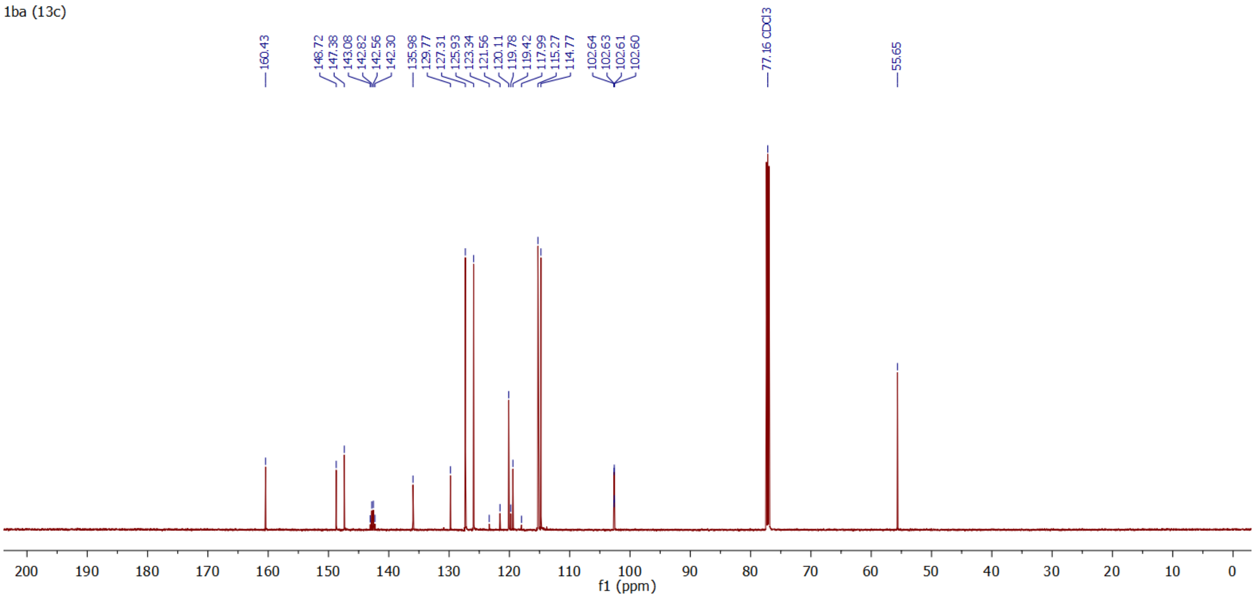

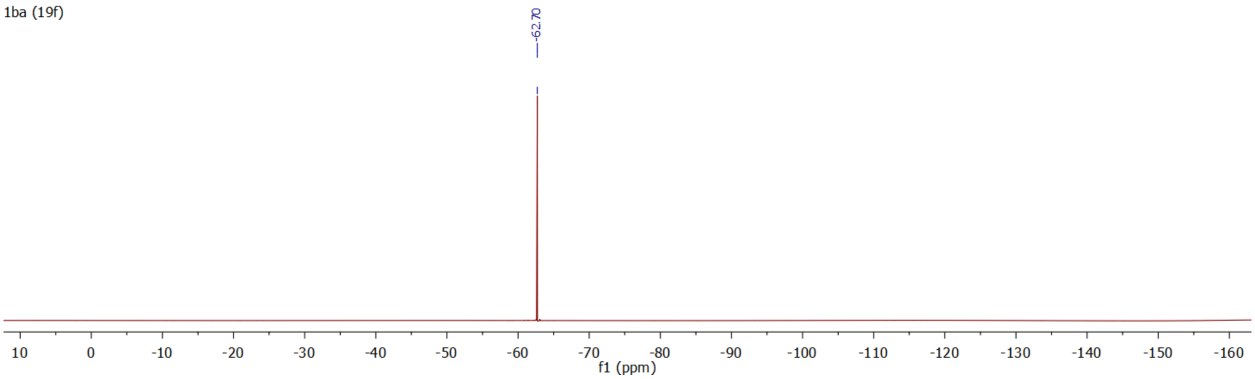

Fig S8. ^{1}H NMR (600 MHz, $CDCl_3$), ^{13}C{^{1}H} NMR (151 MHz, $CDCl_3$) and ^{19}F NMR (565 MHz, $CDCl_3$) spectra for compound **1ba**.

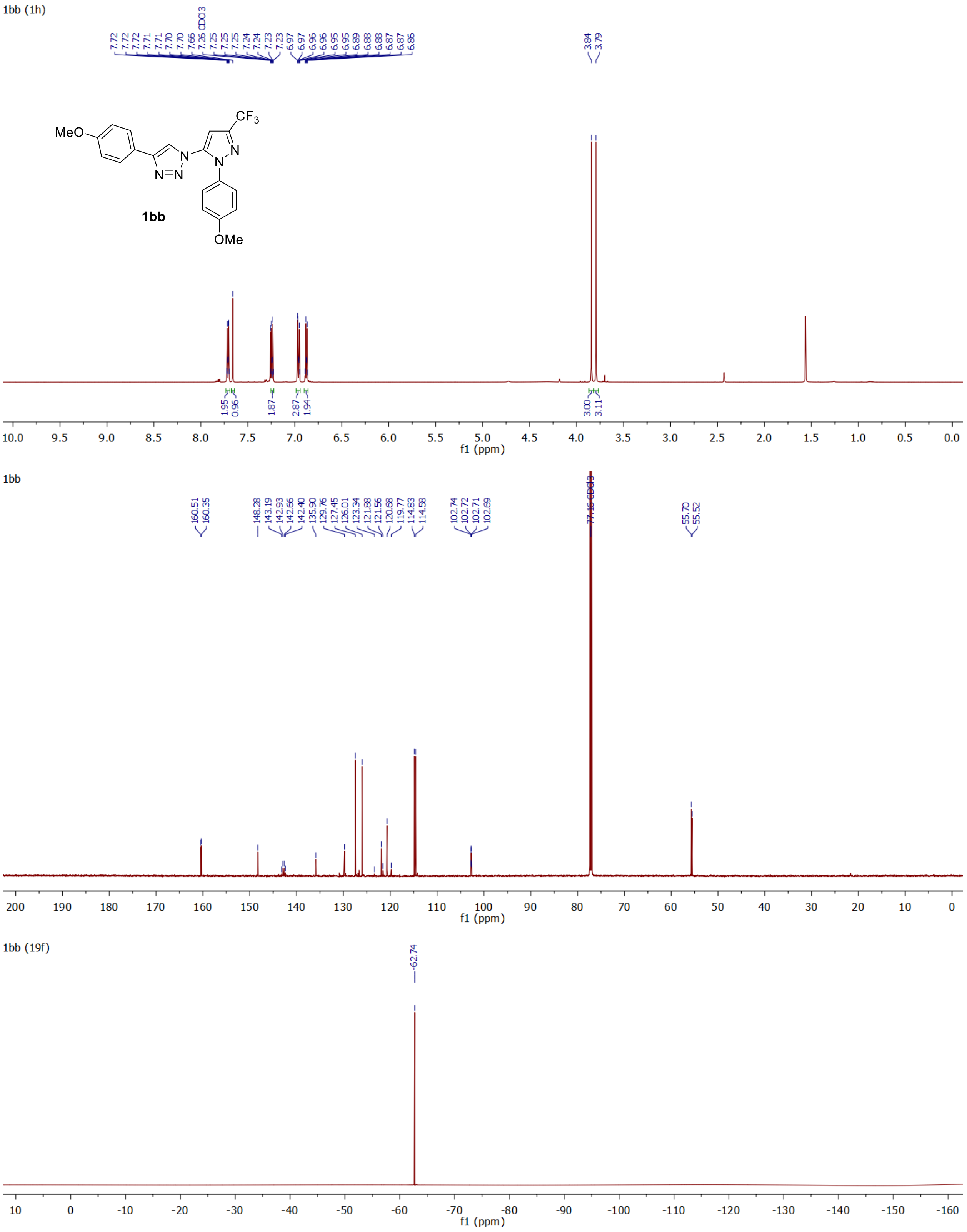

Fig S9. ^{1}H NMR (600 MHz, $CDCl_3$), $^{13}C\{^{1}H\}$ NMR (151 MHz, $CDCl_3$) and ^{19}F NMR (565 MHz, $CDCl_3$) spectra for compound **1bb**.

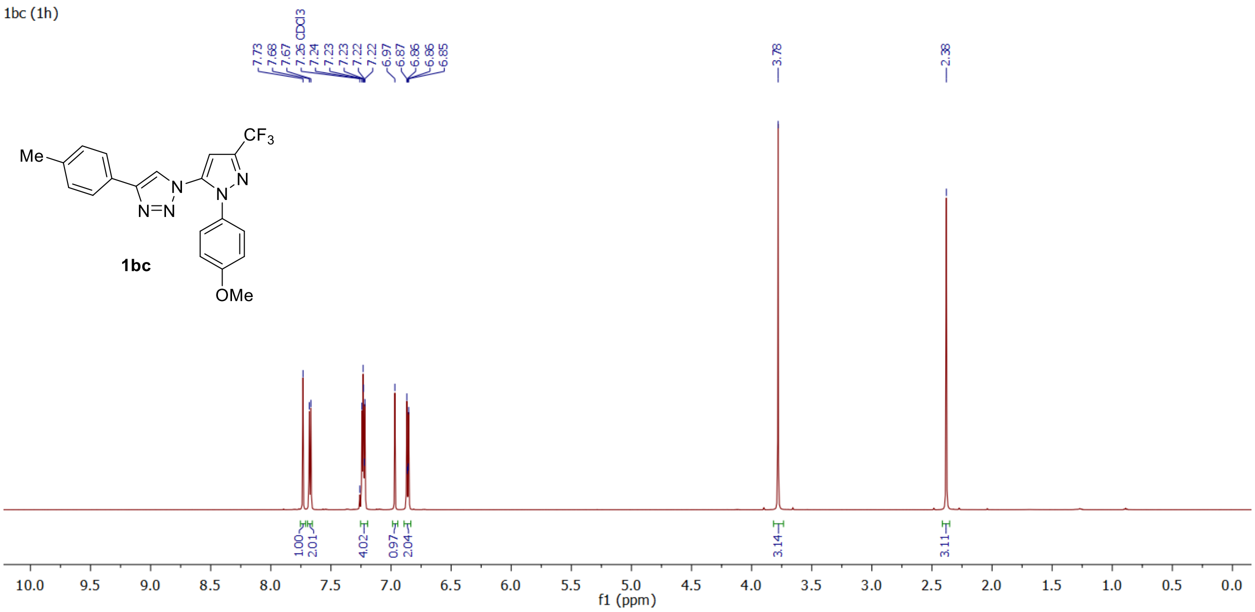

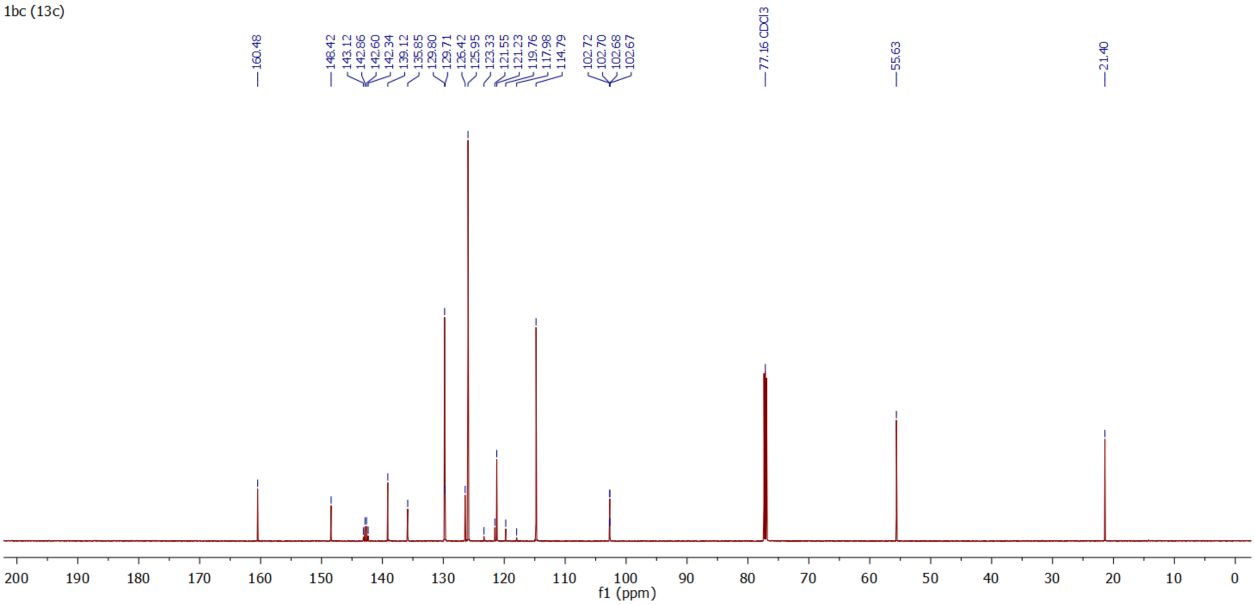

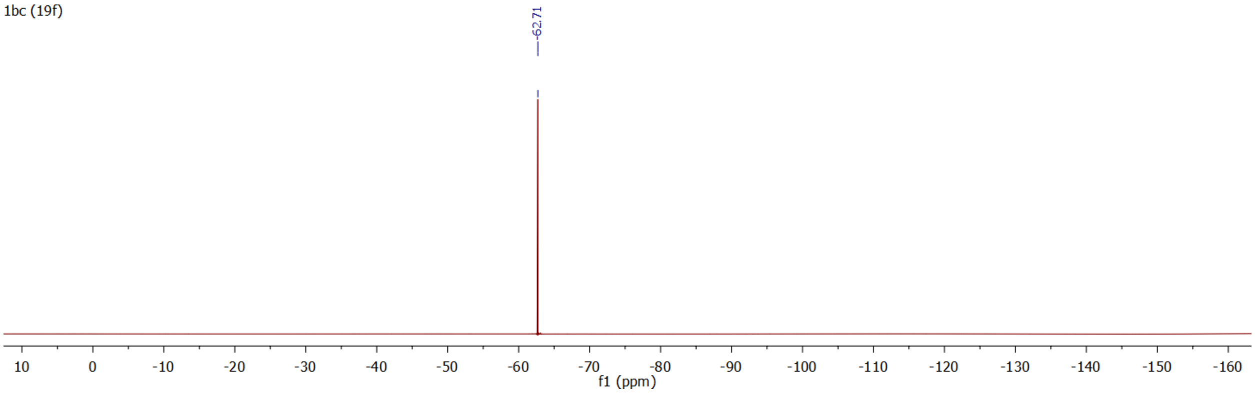

Fig S10. ^{1}H NMR (600 MHz, $CDCl_3$), $^{13}C\{^{1}H\}$ NMR (151 MHz, $CDCl_3$) and ^{19}F NMR (565 MHz, $CDCl_3$) spectra for compound **1bc**.

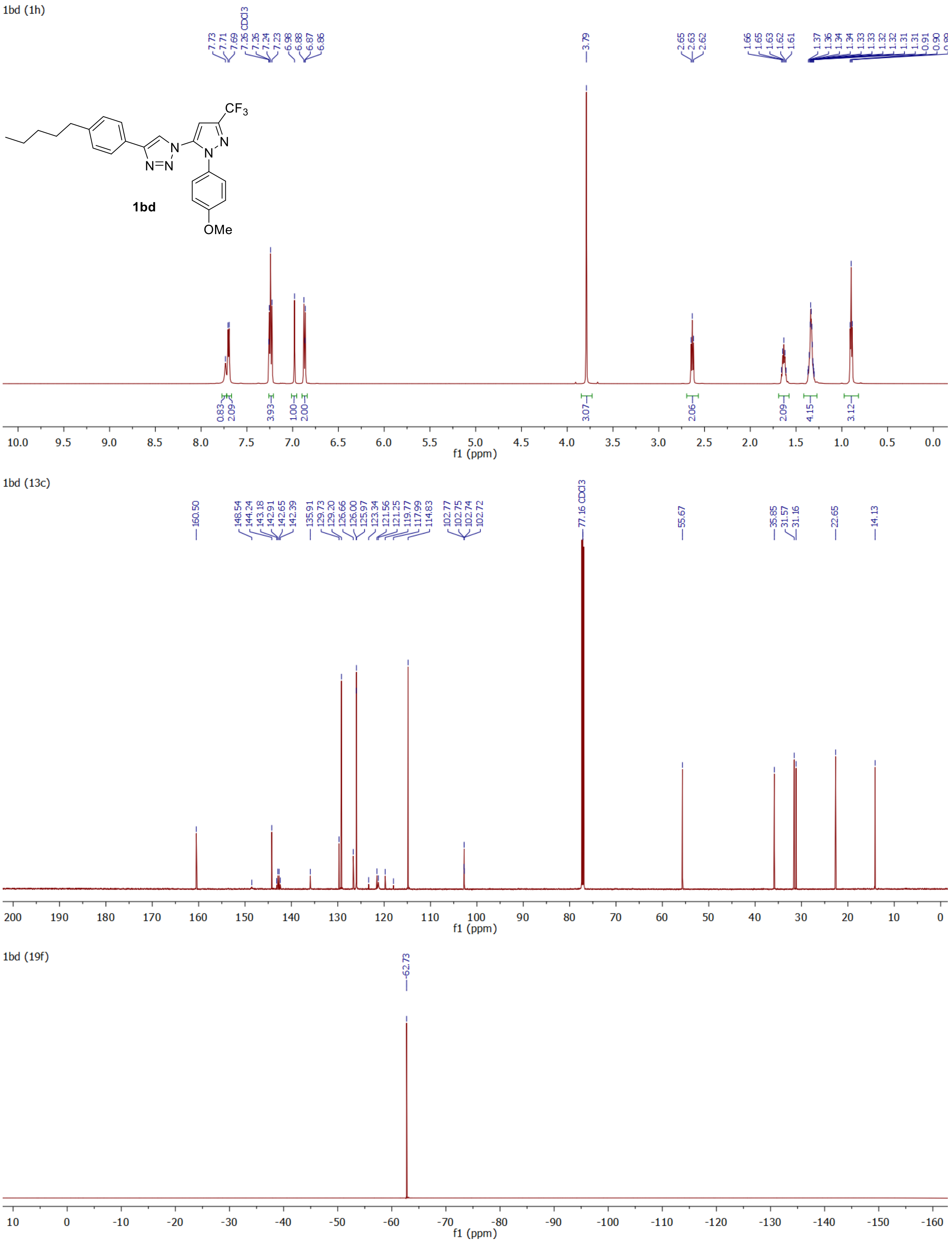

Fig S11. ^{1}H NMR (600 MHz, $CDCl_3$), $^{13}C\{^{1}H\}$ NMR (151 MHz, $CDCl_3$) and ^{19}F NMR (565 MHz, $CDCl_3$) spectra for compound **1bd**.

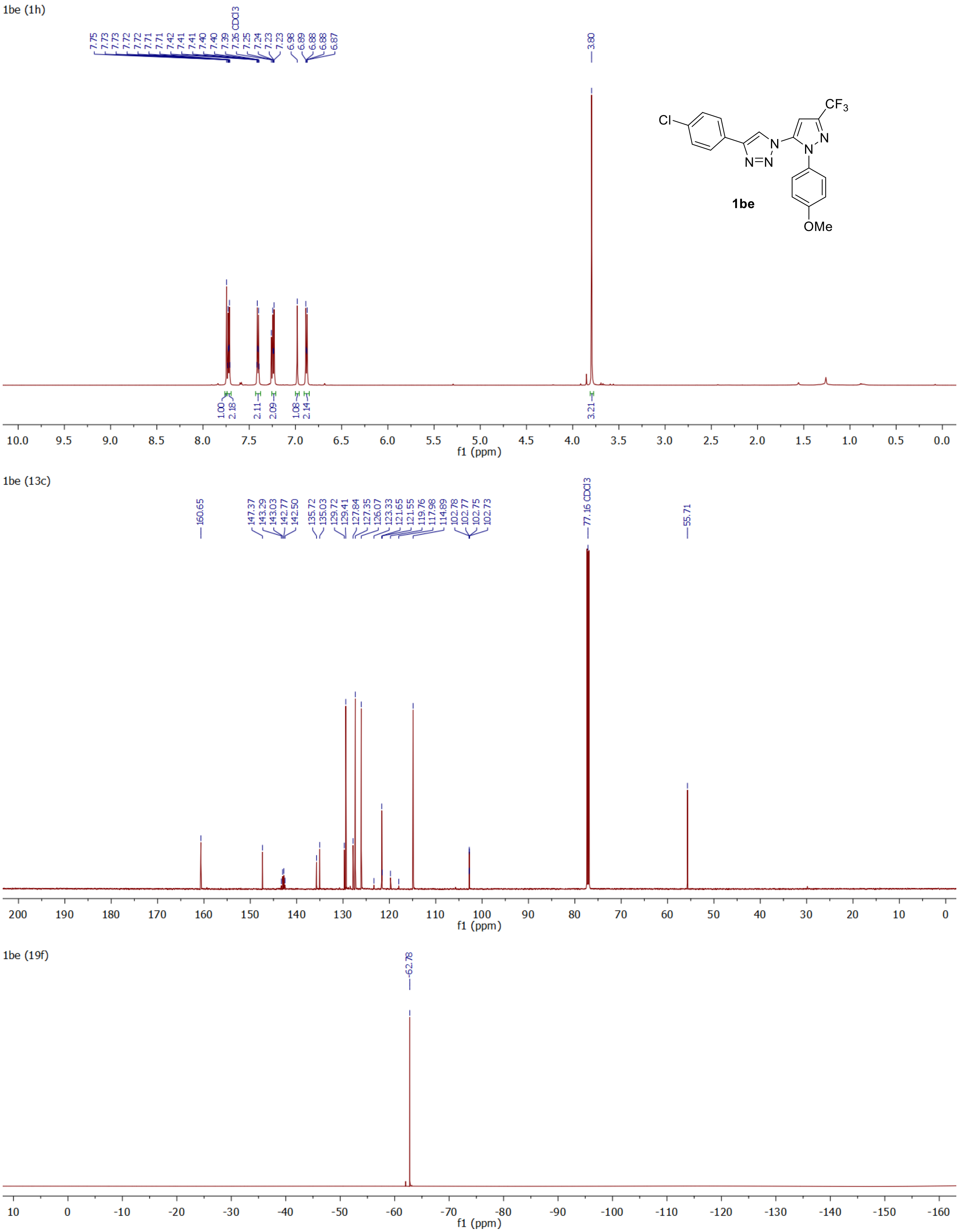

Fig S12. ^{1}H NMR (600 MHz, $CDCl_3$), $^{13}C\{^{1}H\}$ NMR (151 MHz, $CDCl_3$) and ^{19}F NMR (565 MHz, $CDCl_3$) spectra for compound **1be**.

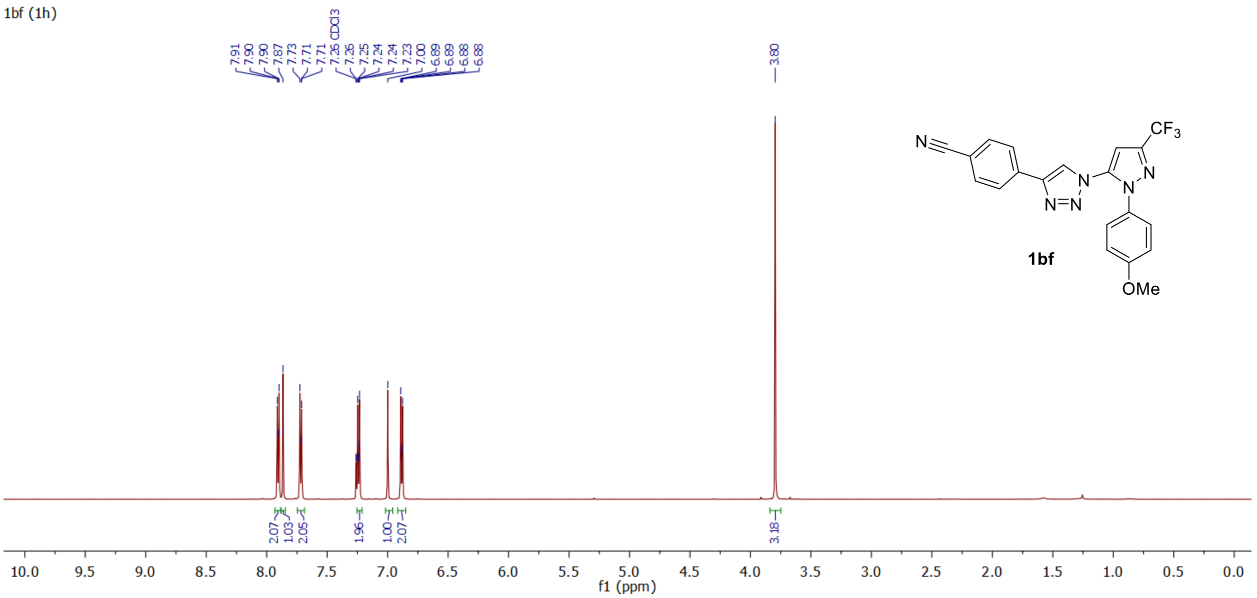

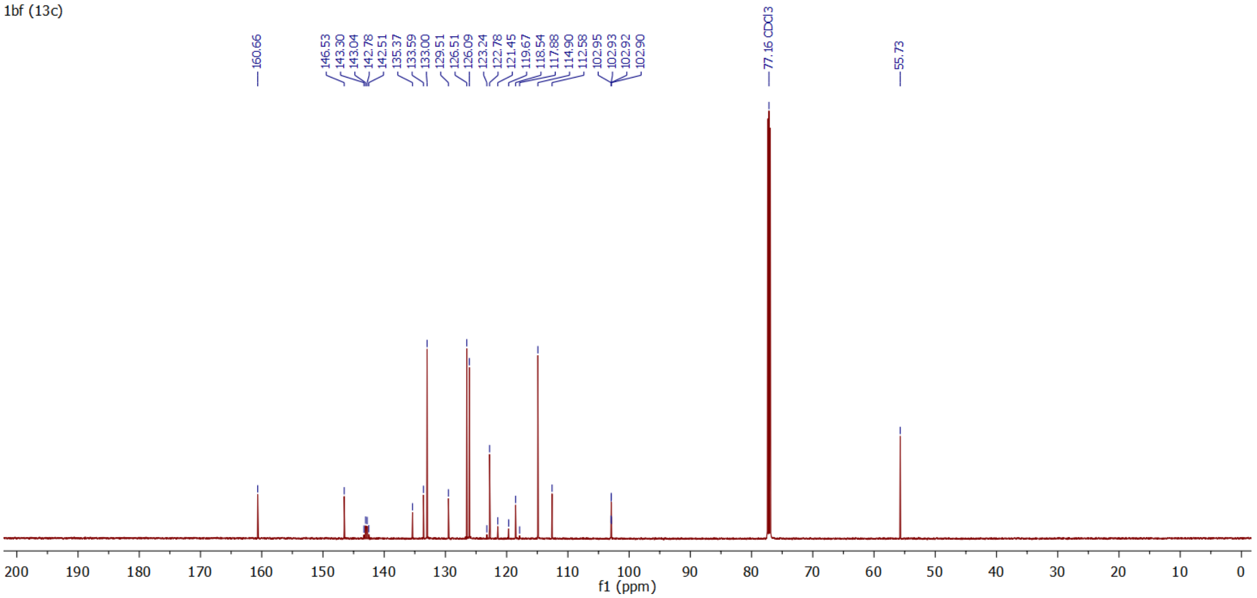

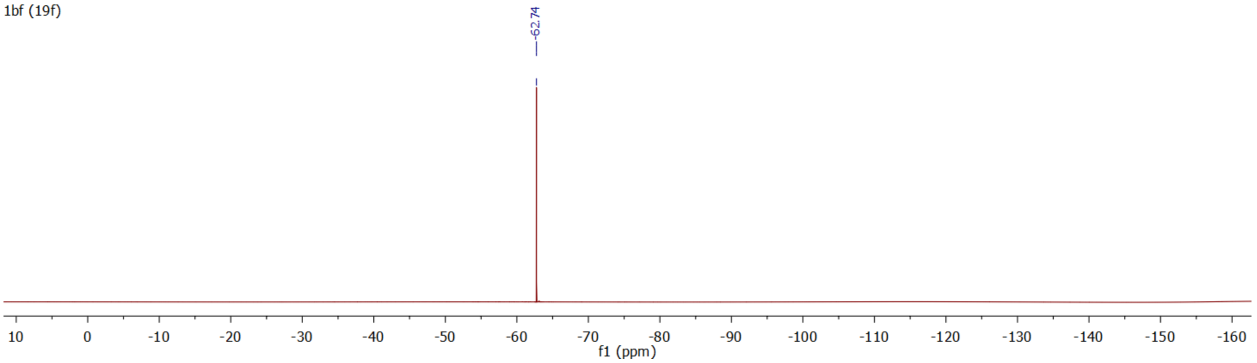

Fig S13. ^{1}H NMR (600 MHz, $CDCl_3$), $^{13}C\{^{1}H\}$ NMR (151 MHz, $CDCl_3$) and ^{19}F NMR (565 MHz, $CDCl_3$) spectra for compound **1bf**.

1bg (1h)

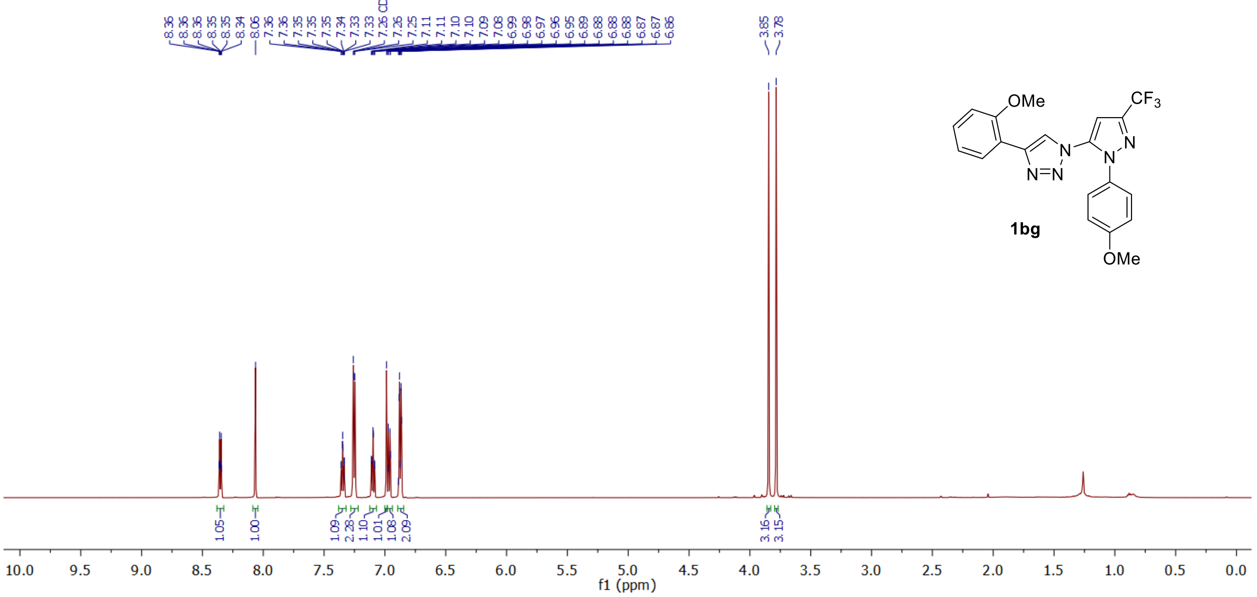

1bg (13c)

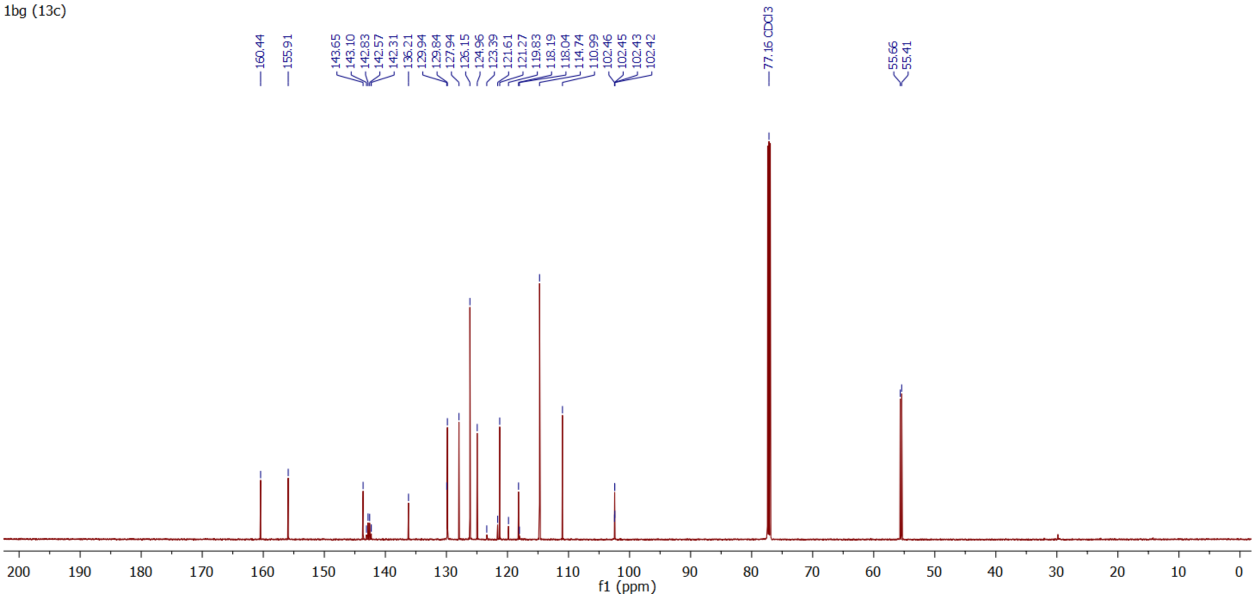

1bg (19f)

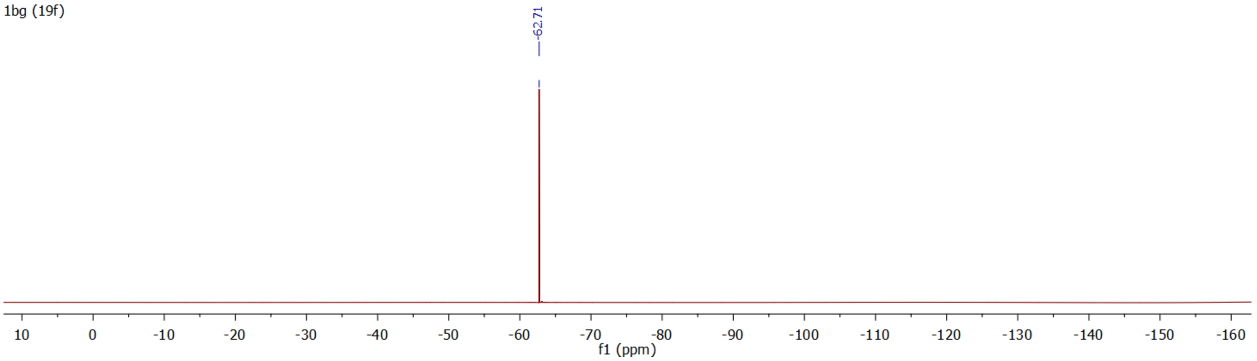

Fig S14. ^{1}H NMR (600 MHz, $CDCl_3$), $^{13}C\{^{1}H\}$ NMR (151 MHz, $CDCl_3$) and ^{19}F NMR (565 MHz, $CDCl_3$) spectra for compound **1bg**.

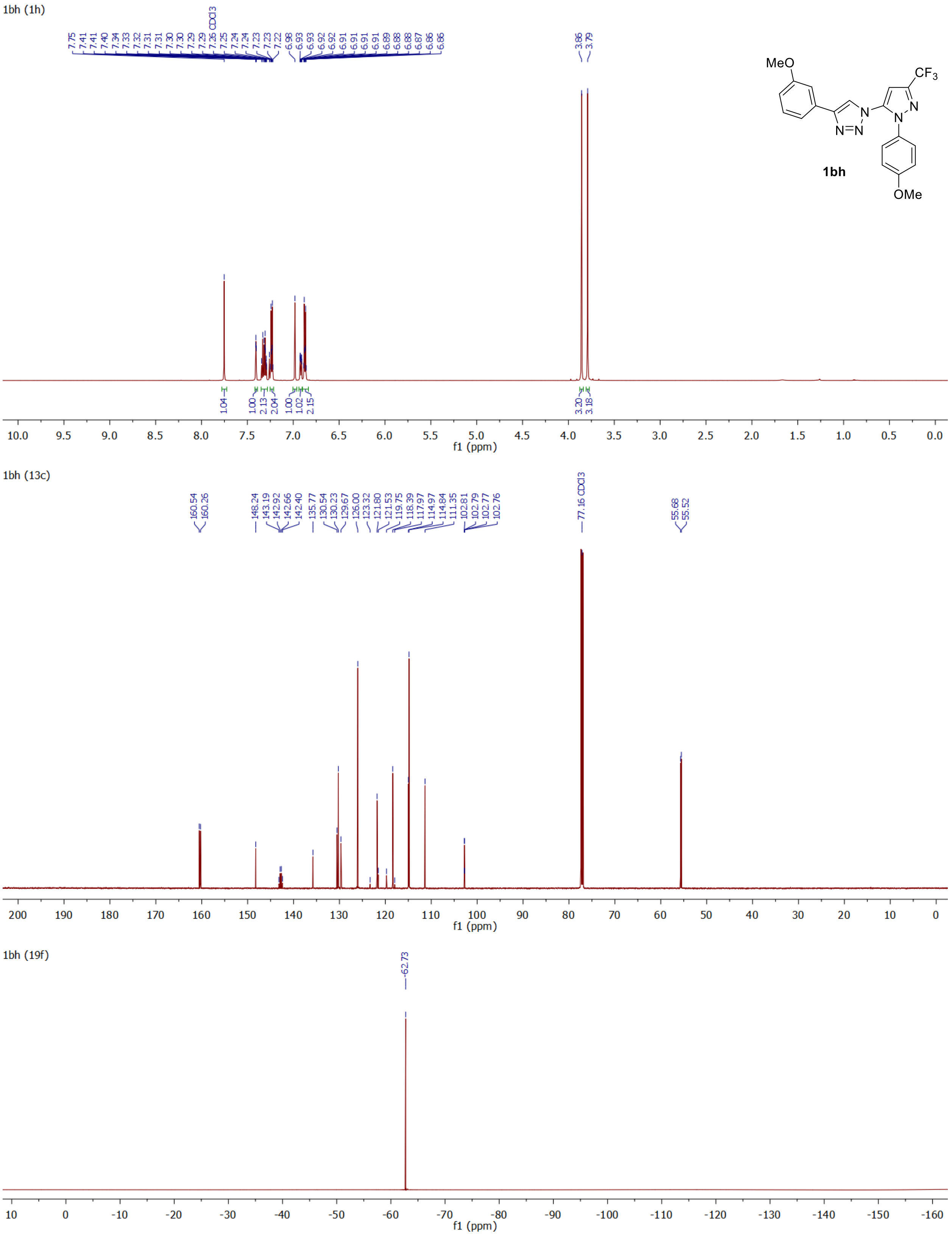

Fig S15. ^{1}H NMR (600 MHz, $CDCl_3$), $^{13}C\{^{1}H\}$ NMR (151 MHz, $CDCl_3$) and ^{19}F NMR (565 MHz, $CDCl_3$) spectra for compound **1bh**.

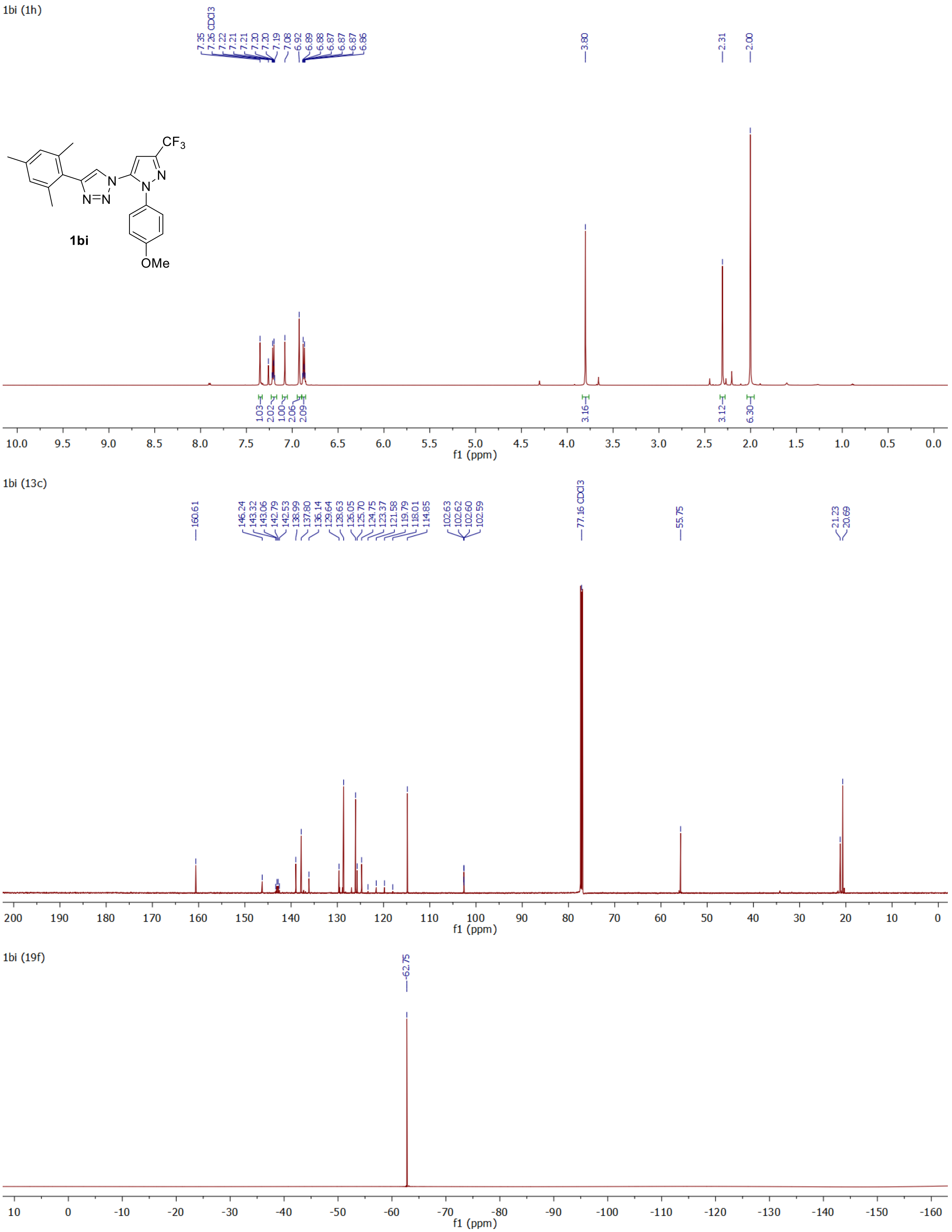

Fig S16. ^{1}H NMR (600 MHz, $CDCl_3$), $^{13}C\{^1H\}$ NMR (151 MHz, $CDCl_3$) and ^{19}F NMR (565 MHz, $CDCl_3$) spectra for compound **1bi**.

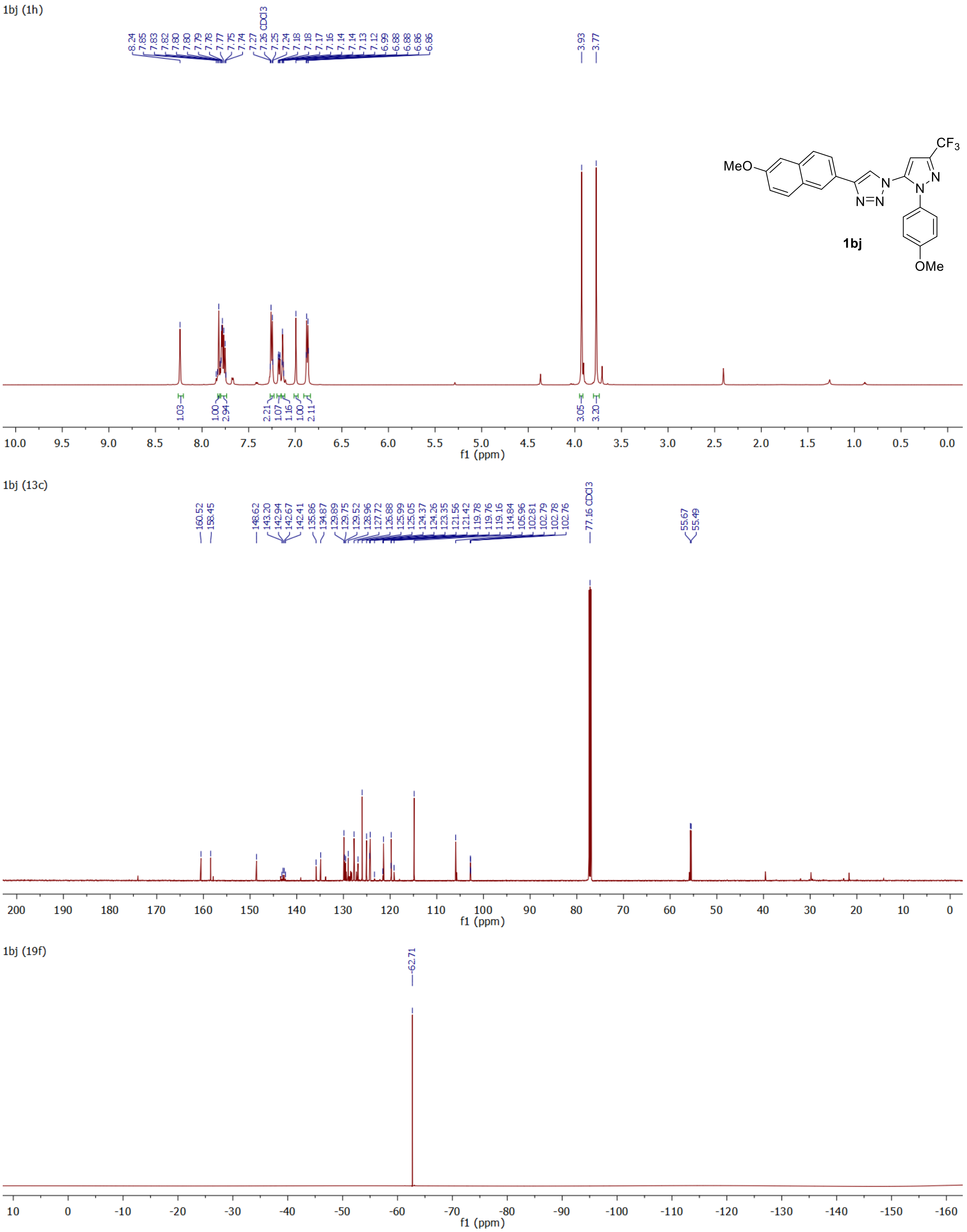

Fig S17. ^{1}H NMR (600 MHz, $CDCl_3$), $^{13}C\{^{1}H\}$ NMR (151 MHz, $CDCl_3$) and ^{19}F NMR (565 MHz, $CDCl_3$) spectra for compound **1bj**.

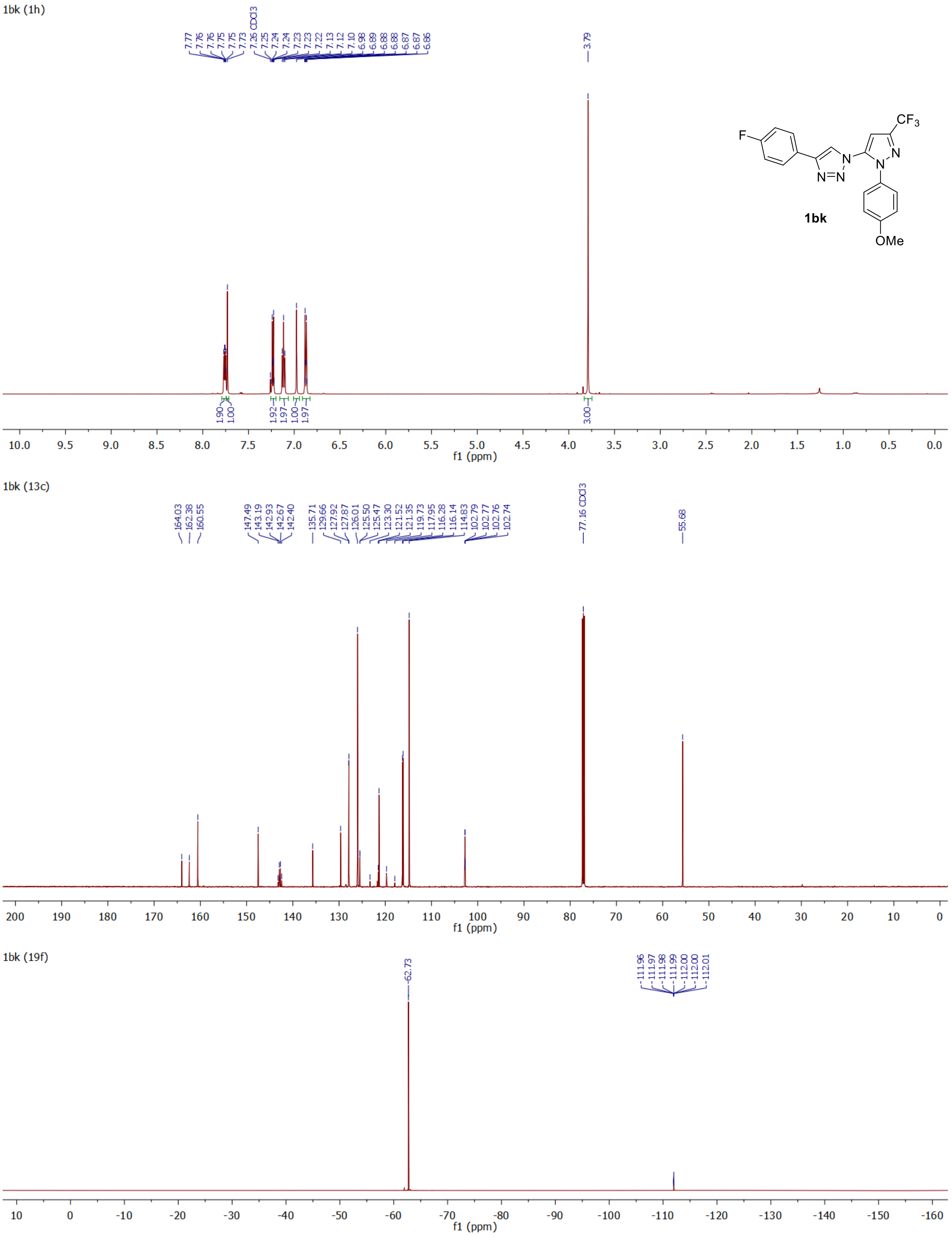

Fig S18. ^{1}H NMR (600 MHz, $CDCl_3$), $^{13}C\{^{1}H\}$ NMR (151 MHz, $CDCl_3$) and ^{19}F NMR (565 MHz, $CDCl_3$) spectra for compound **1bk**.

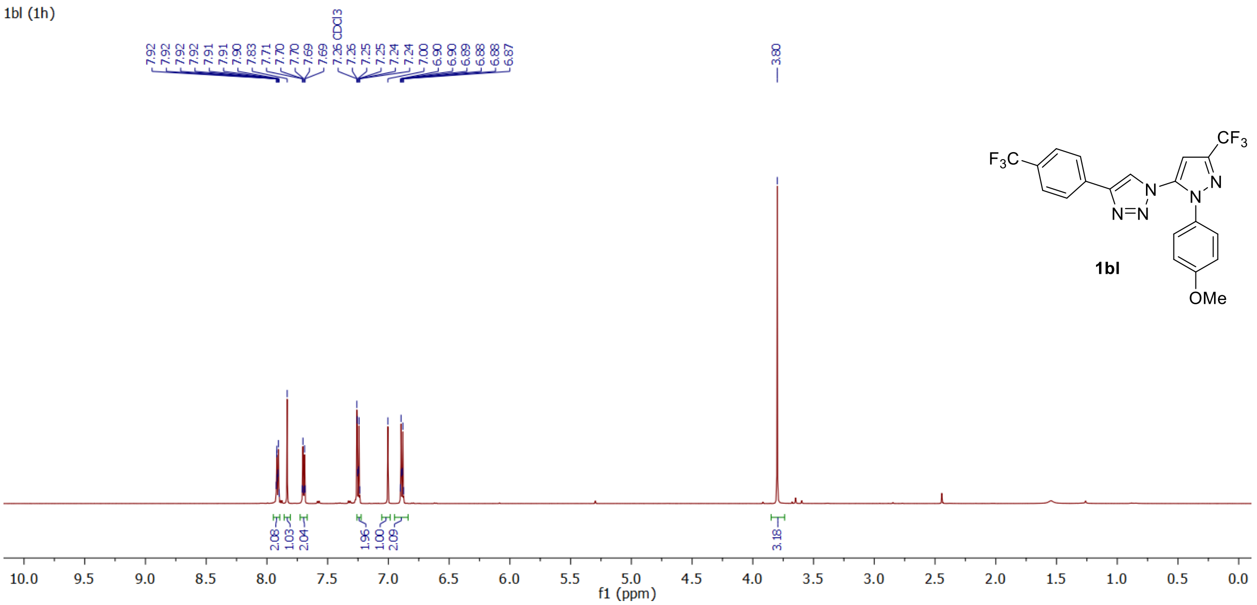

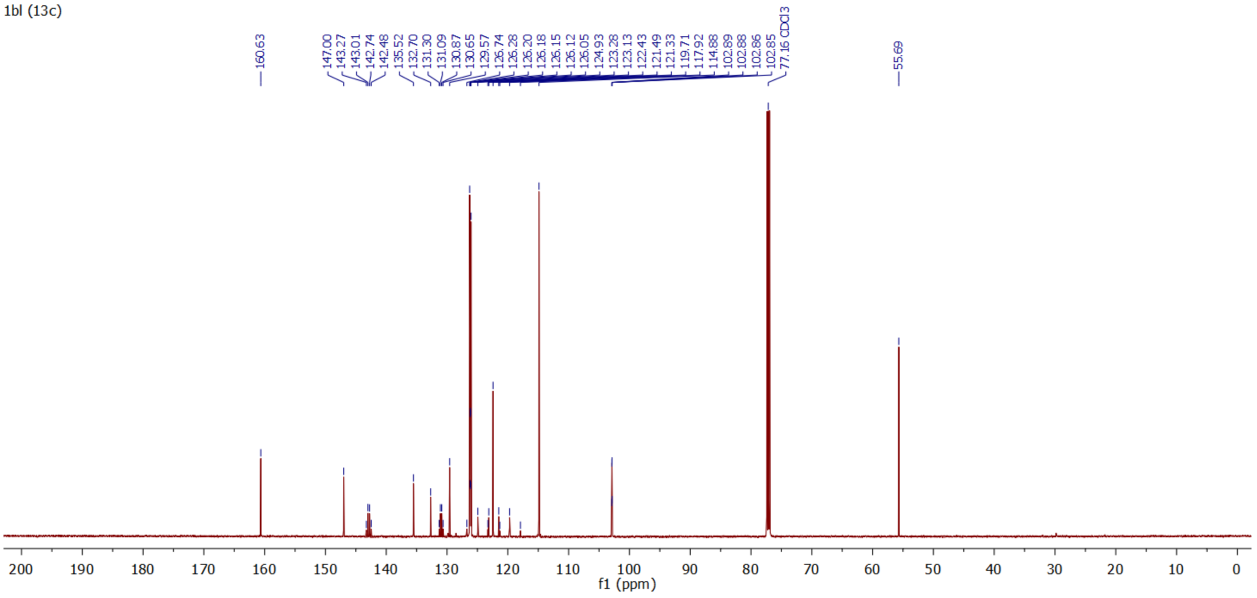

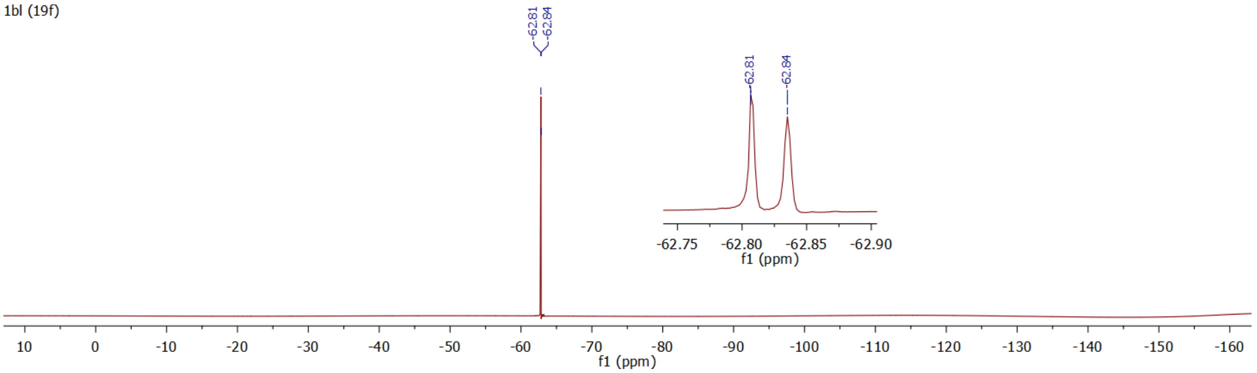

Fig S19. ^{1}H NMR (600 MHz, $CDCl_3$), $^{13}C\{^{1}H\}$ NMR (151 MHz, $CDCl_3$) and ^{19}F NMR (565 MHz, $CDCl_3$) spectra for compound **1bl**.

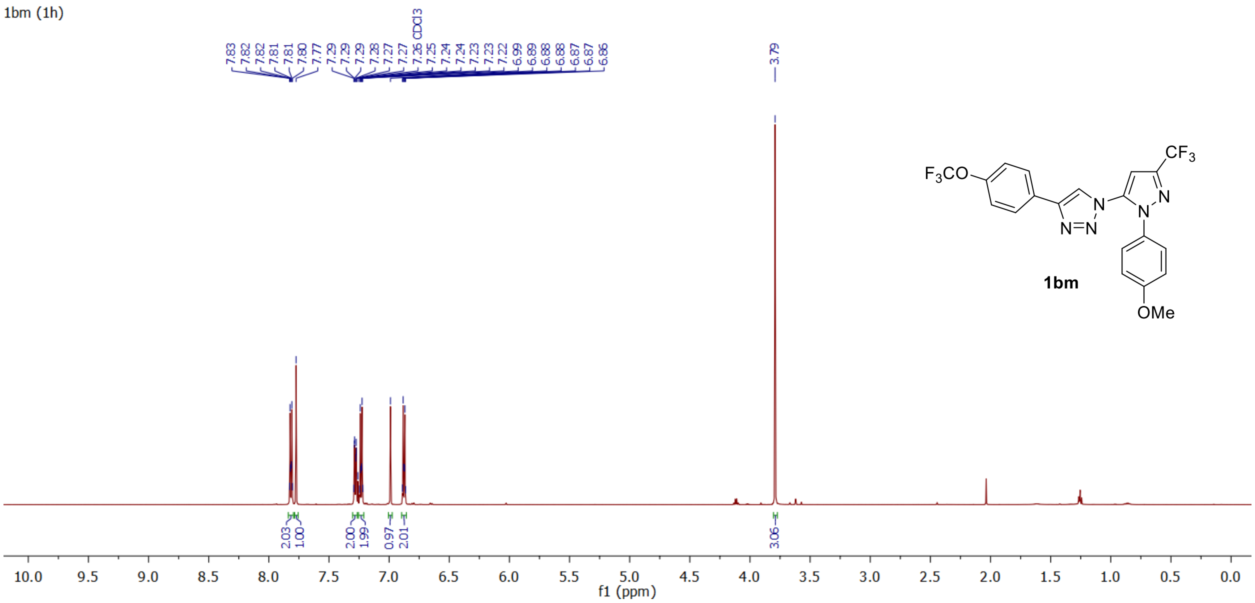

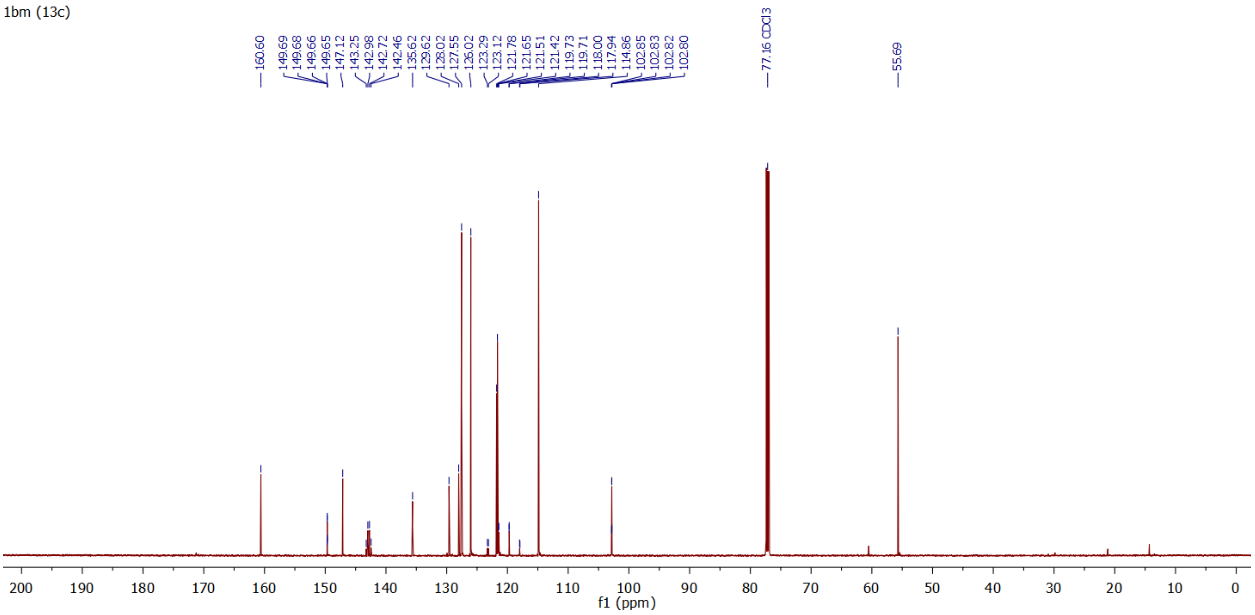

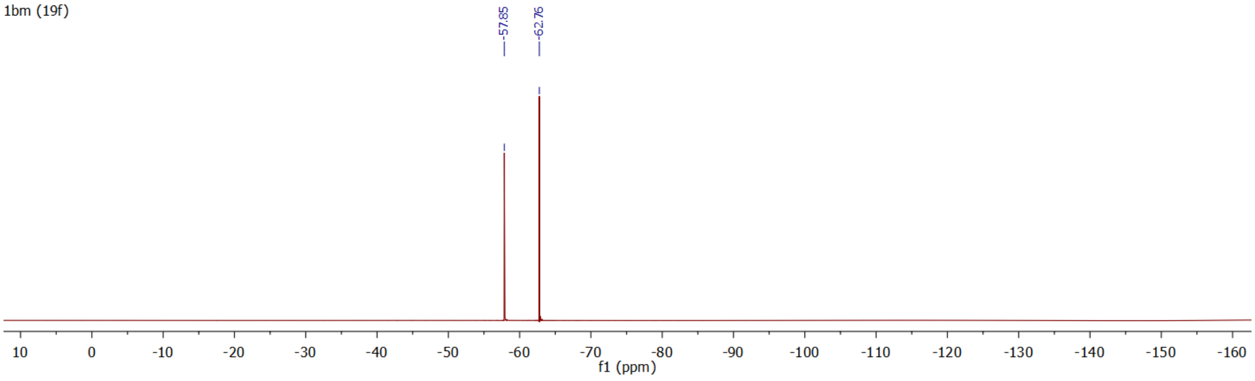

Fig S20. ^{1}H NMR (600 MHz, $CDCl_3$), $^{13}C\{^{1}H\}$ NMR (151 MHz, $CDCl_3$) and ^{19}F NMR (565 MHz, $CDCl_3$) spectra for compound **1bm**.

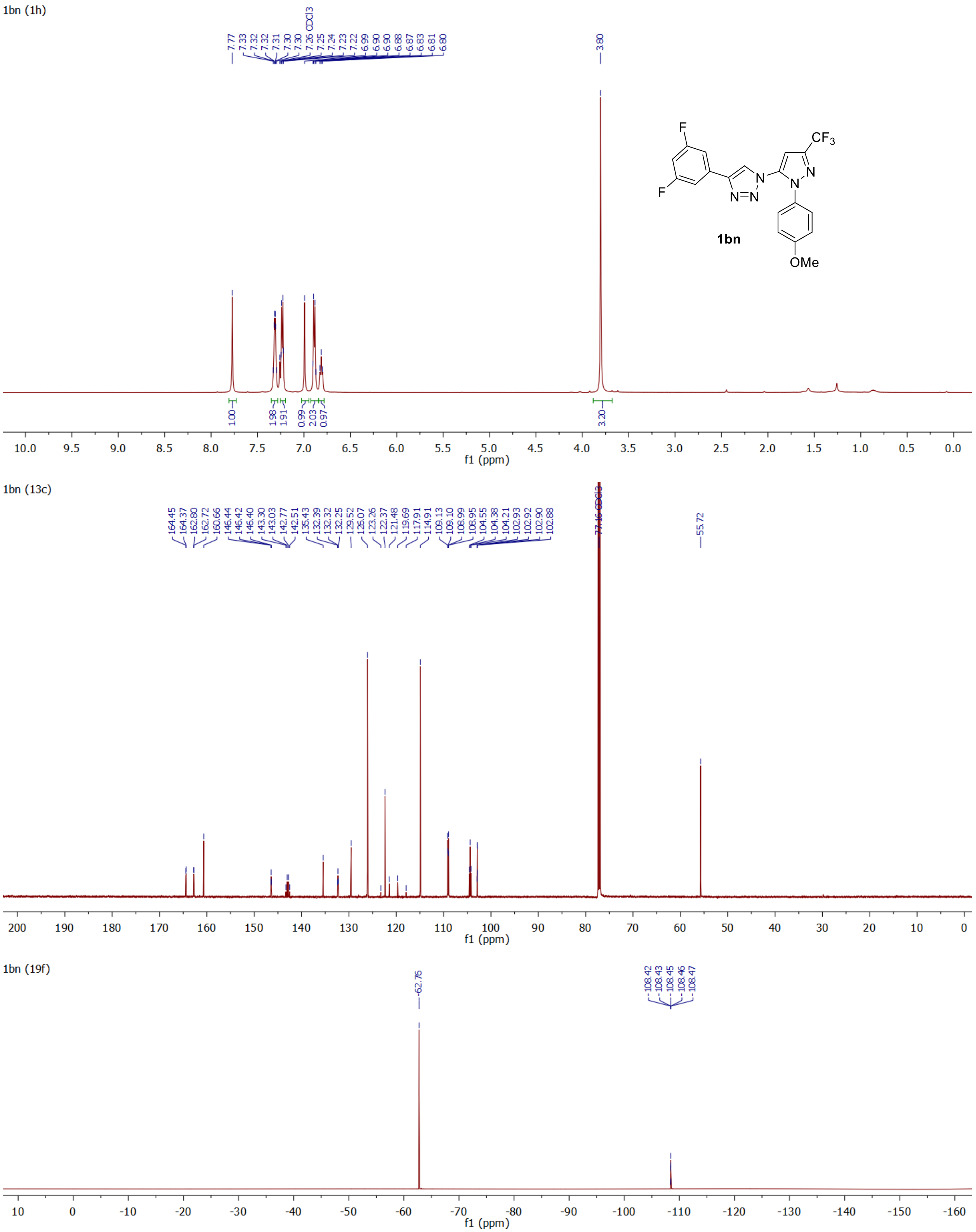

Fig S21. ^{1}H NMR (600 MHz, $CDCl_3$), $^{13}C\{^{1}H\}$ NMR (151 MHz, $CDCl_3$) and ^{19}F NMR (565 MHz, $CDCl_3$) spectra for compound **1bn**.

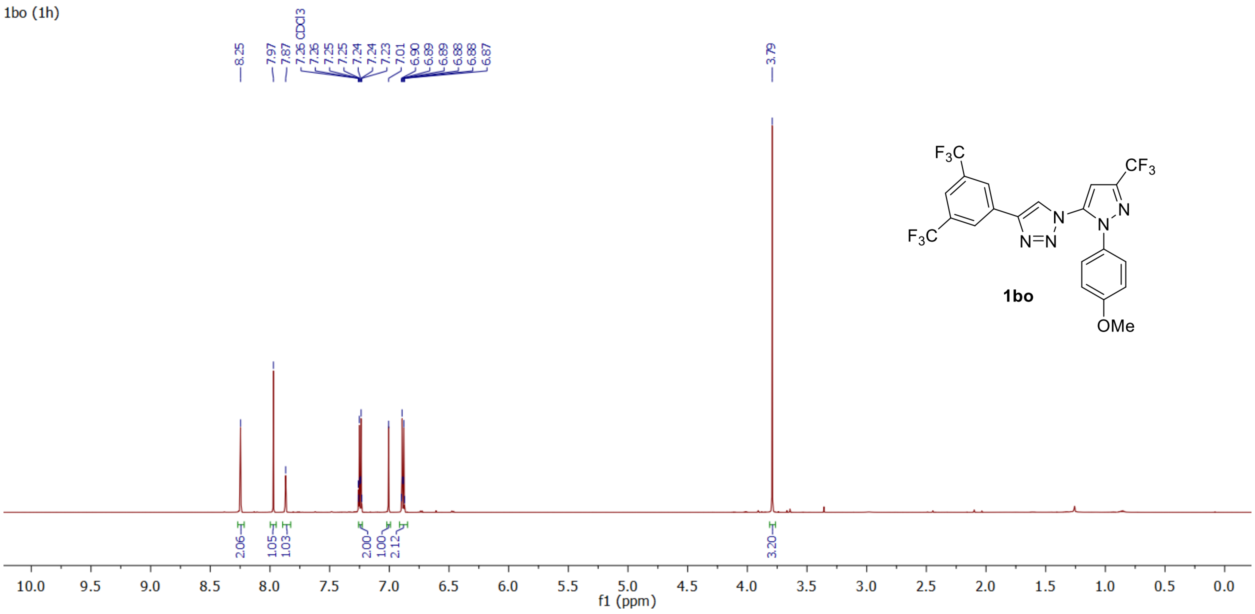

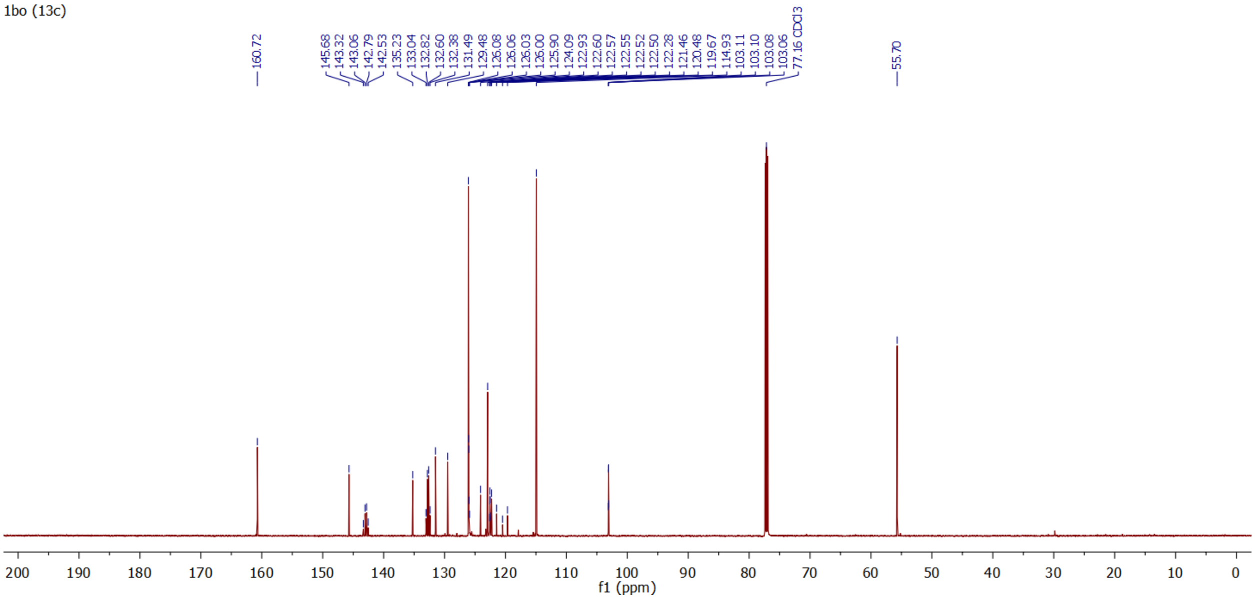

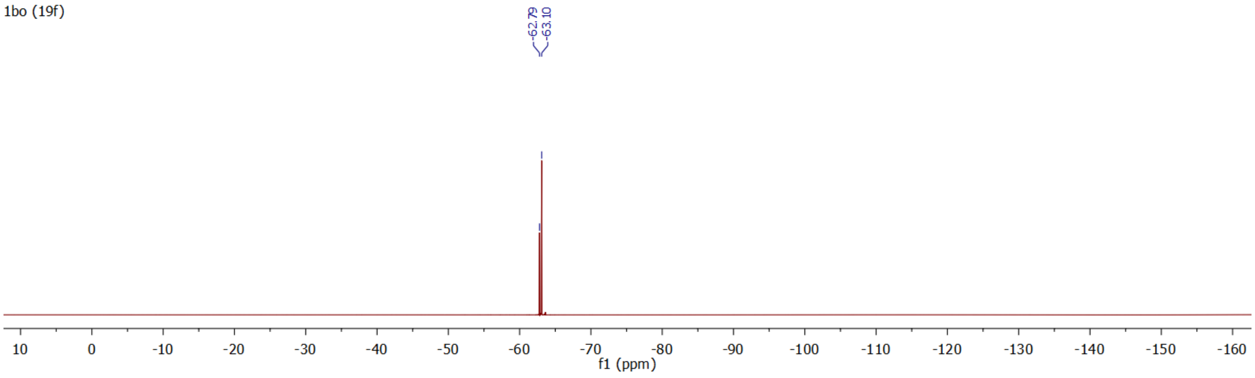

Fig S22. ^{1}H NMR (600 MHz, $CDCl_3$), $^{13}C\{^{1}H\}$ NMR (151 MHz, $CDCl_3$) and ^{19}F NMR (565 MHz, $CDCl_3$) spectra for compound **1bo**.

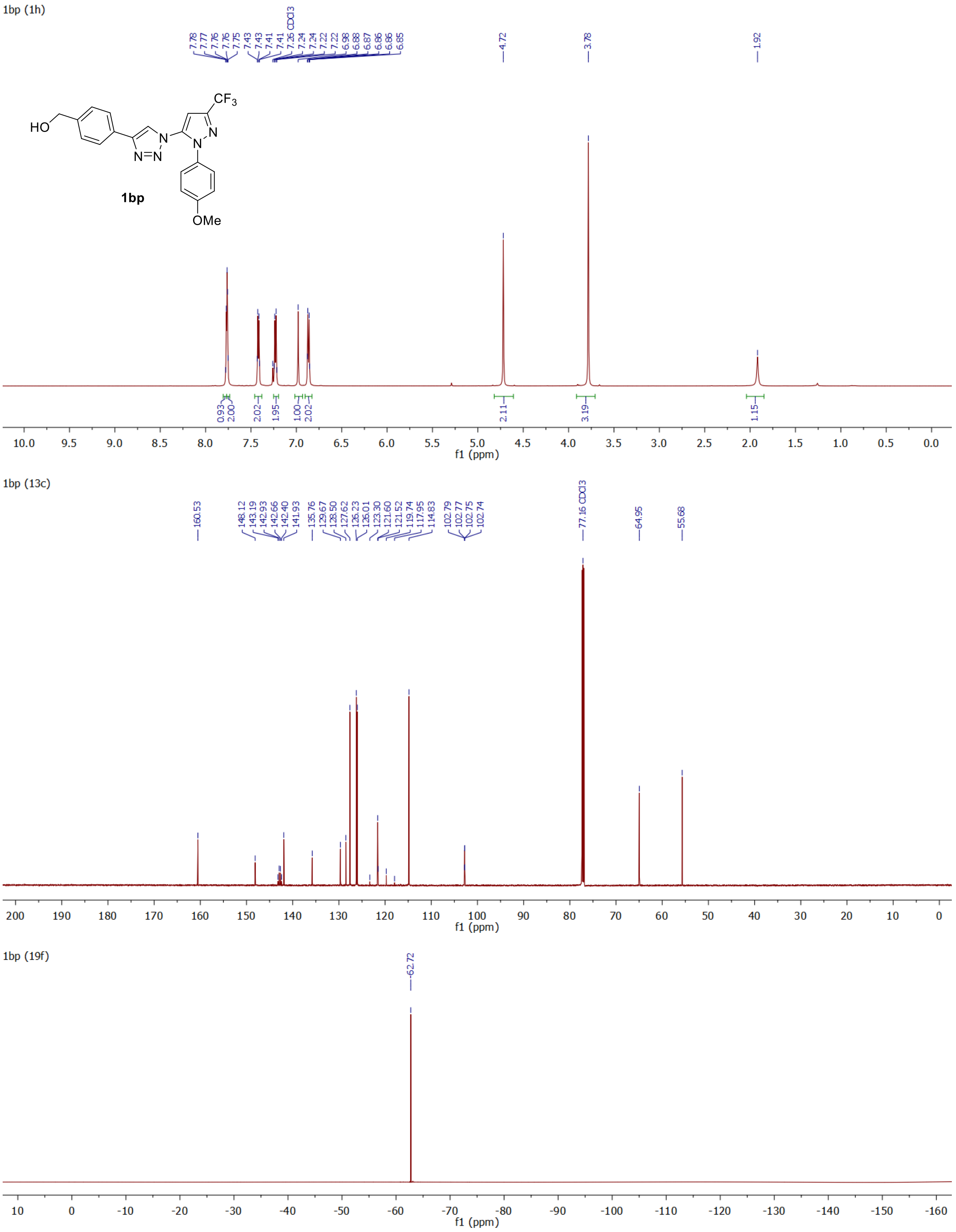

Fig S23. ^{1}H NMR (600 MHz, $CDCl_3$), $^{13}C\{^{1}H\}$ NMR (151 MHz, $CDCl_3$) and ^{19}F NMR (565 MHz, $CDCl_3$) spectra for compound **1bp**.

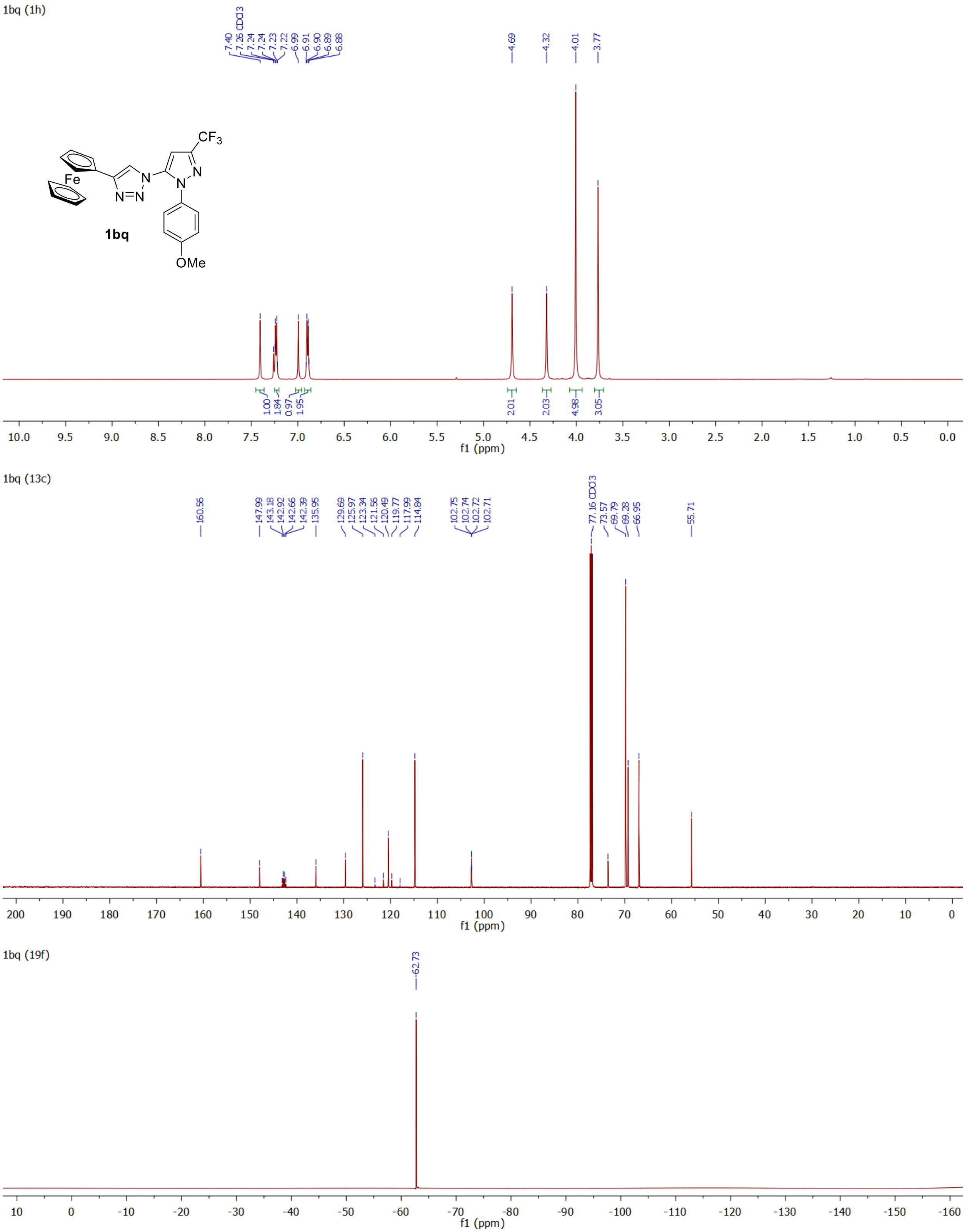

Fig S24. ^{1}H NMR (600 MHz, $CDCl_3$), $^{13}C\{^{1}H\}$ NMR (151 MHz, $CDCl_3$) and ^{19}F NMR (565 MHz, $CDCl_3$) spectra for compound **1bq**.

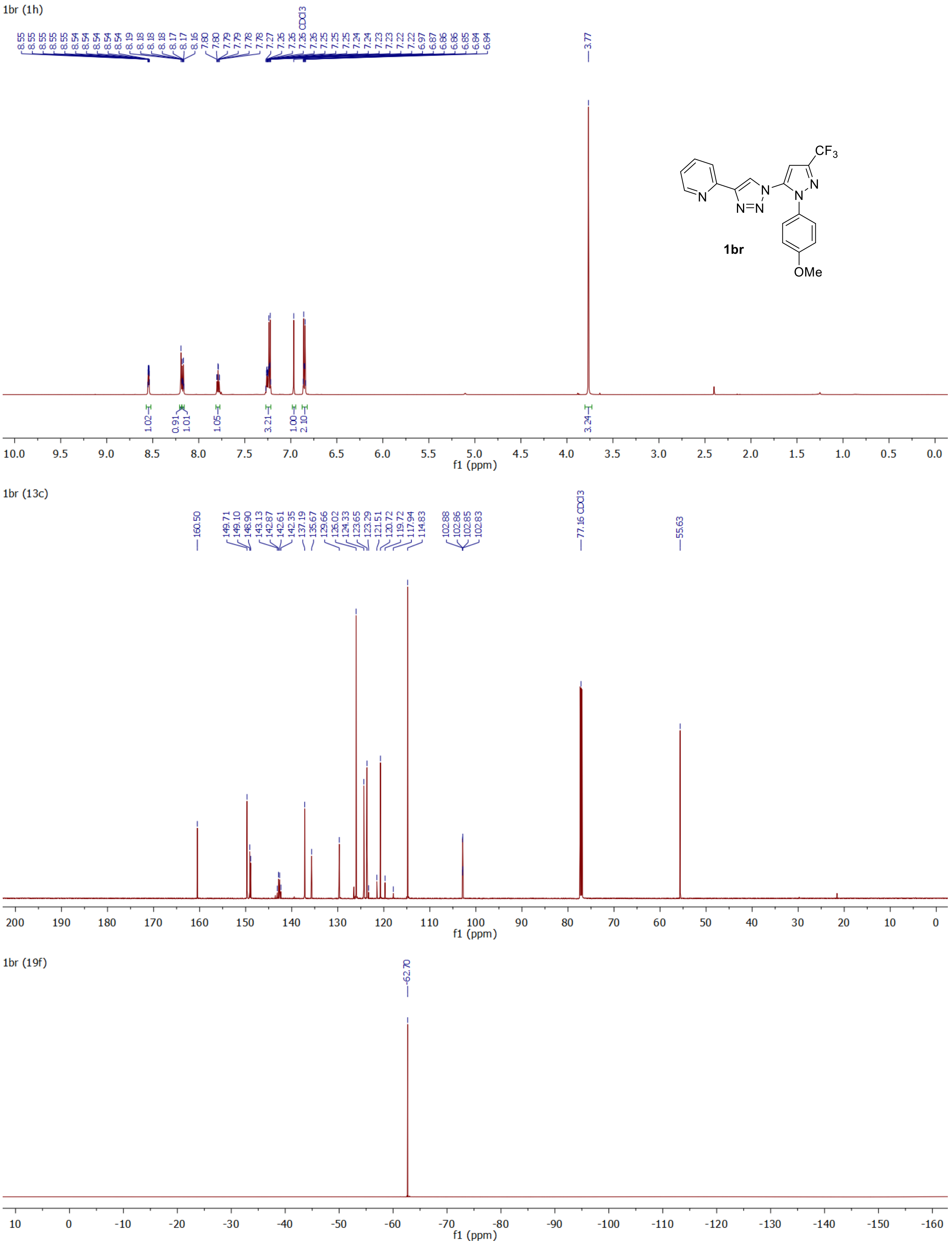

Fig S25. ^{1}H NMR (600 MHz, $CDCl_3$), $^{13}C\{^{1}H\}$ NMR (151 MHz, $CDCl_3$) and ^{19}F NMR (565 MHz, $CDCl_3$) spectra for compound **1br**.

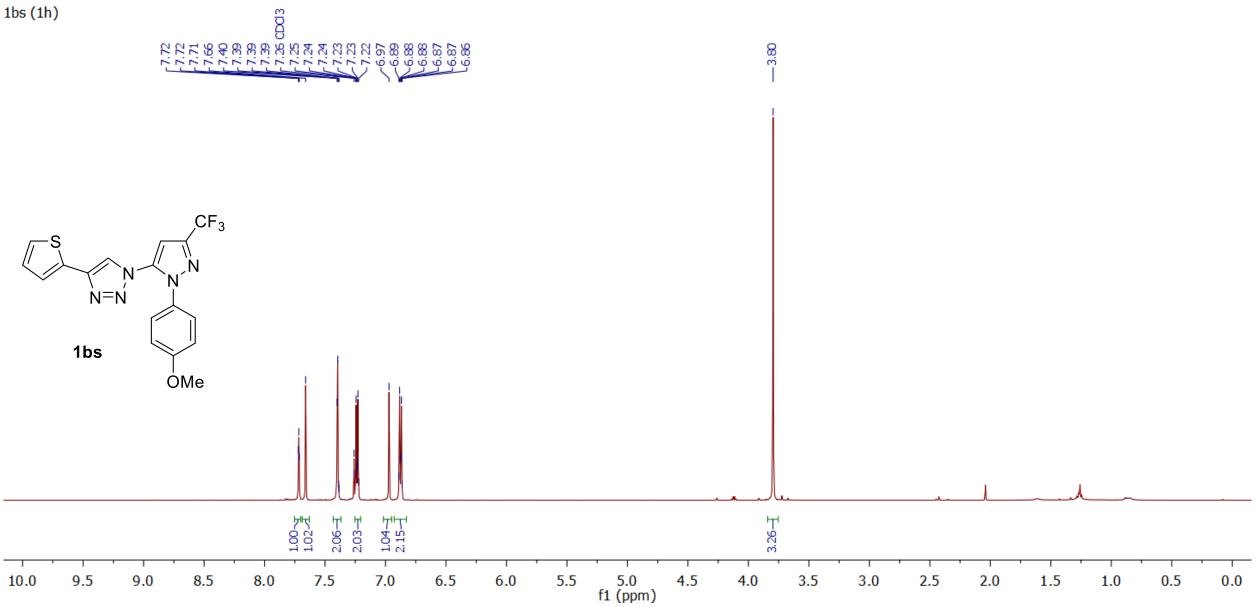

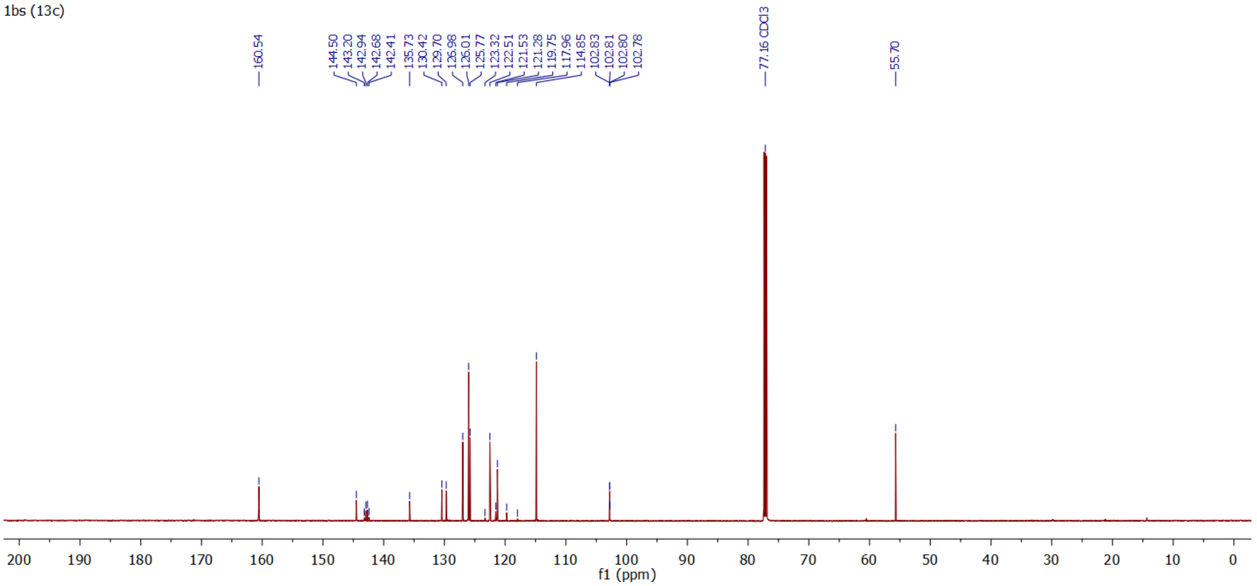

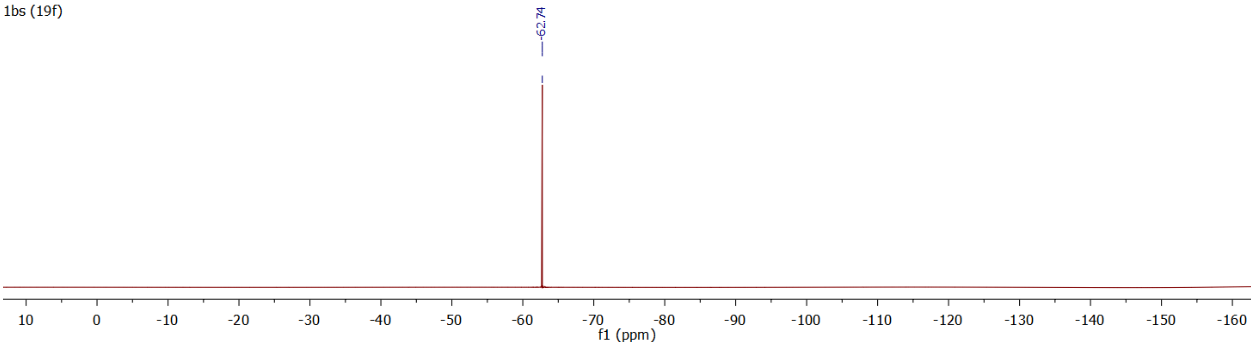

Fig S26. ^{1}H NMR (600 MHz, $CDCl_3$), $^{13}C\{^{1}H\}$ NMR (151 MHz, $CDCl_3$) and ^{19}F NMR (565 MHz, $CDCl_3$) spectra for compound **1bs**.

1bt (1h)

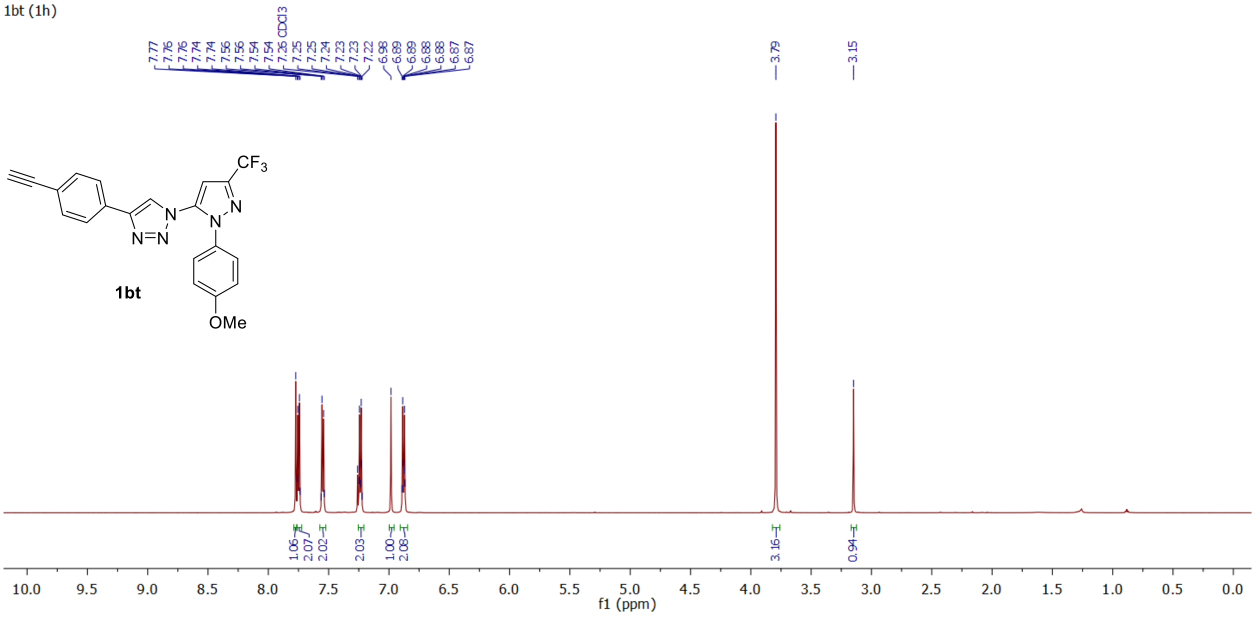

1bt (13c)

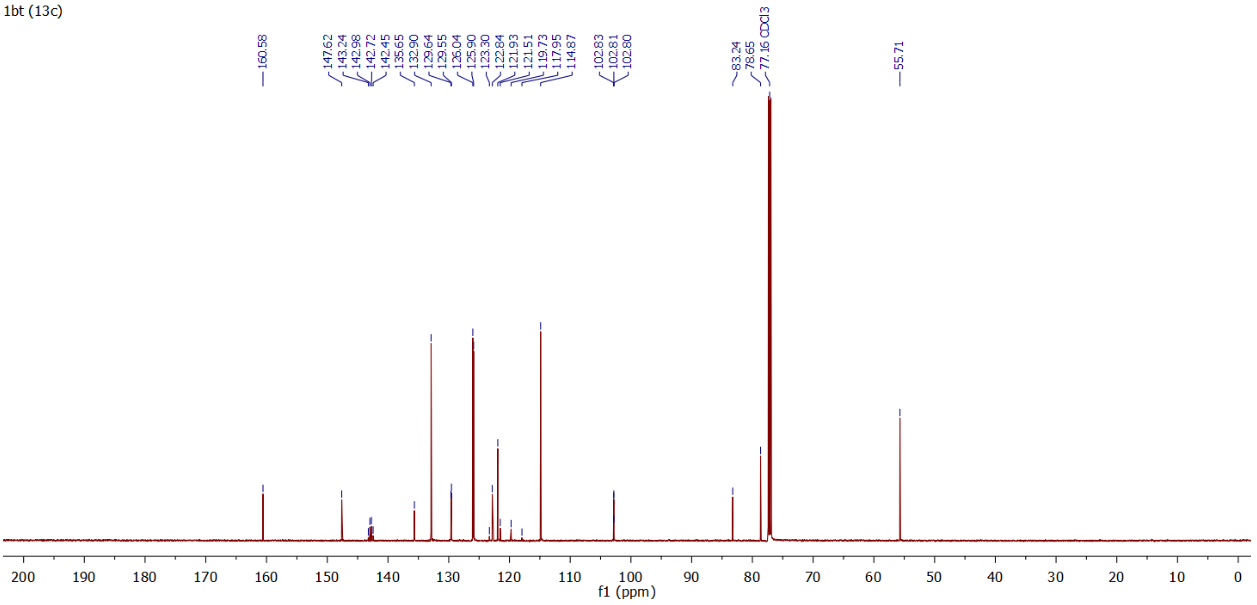

1bt (19f)

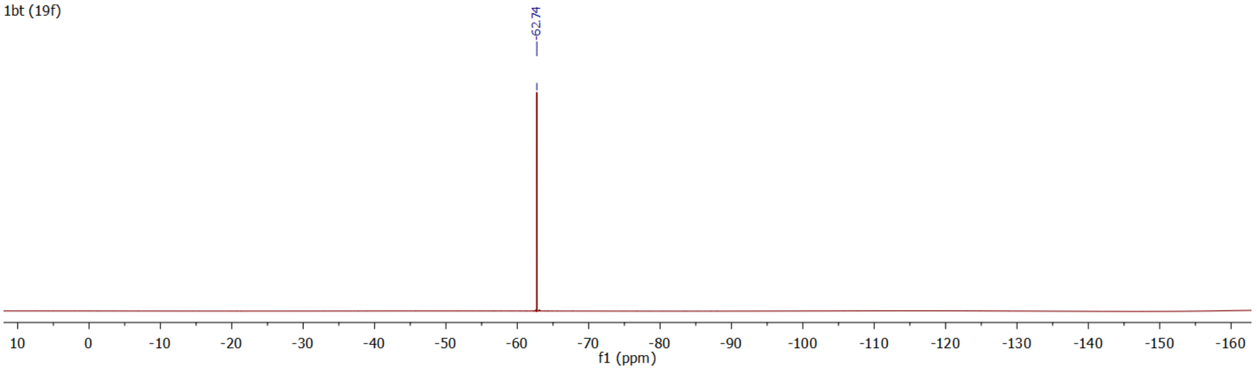

Fig S27. ^{1}H NMR (600 MHz, $CDCl_3$), $^{13}C\{^{1}H\}$ NMR (151 MHz, $CDCl_3$) and ^{19}F NMR (565 MHz, $CDCl_3$) spectra for compound **1bt**.

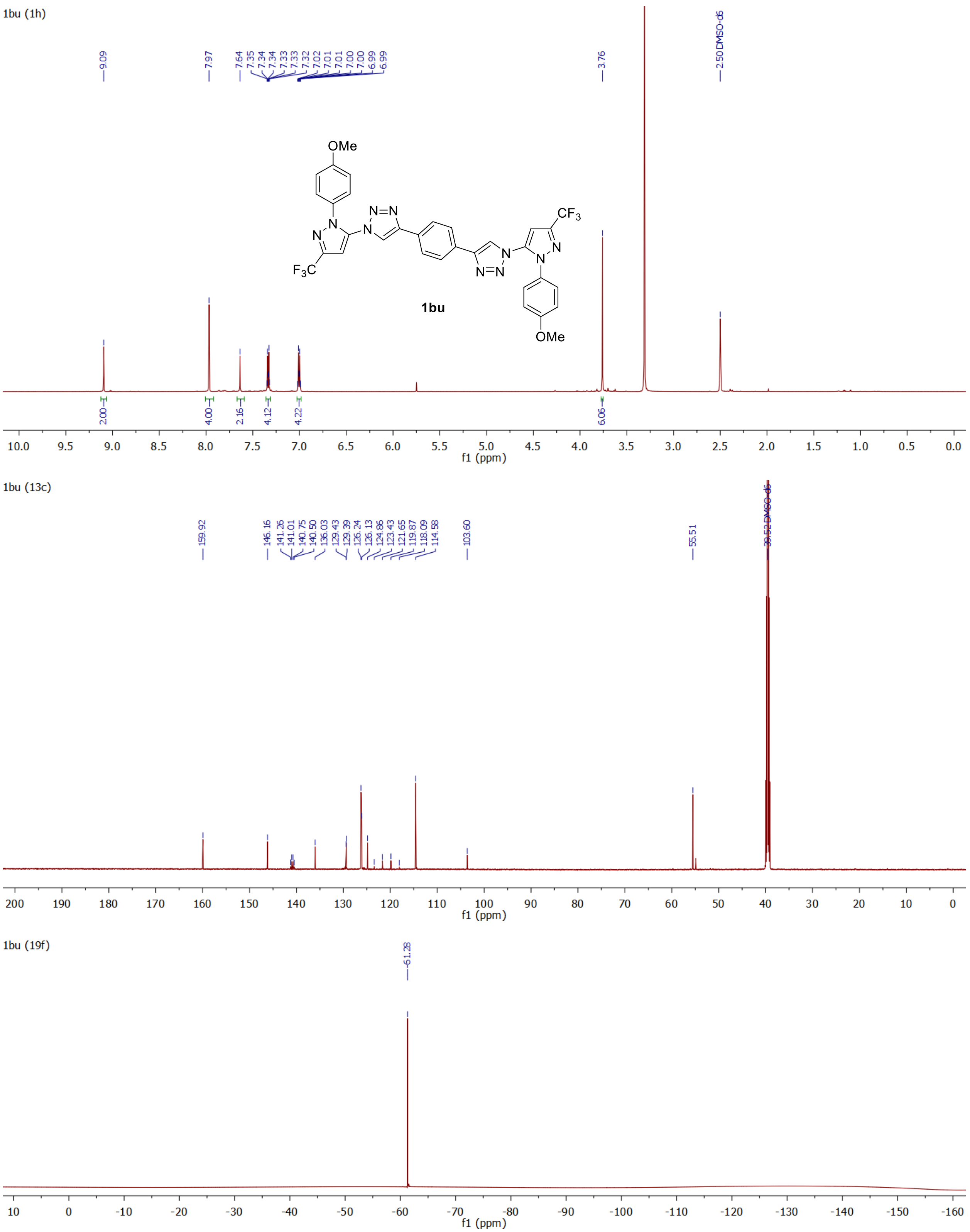

Fig S28. ^{1}H NMR (600 MHz, DMSO-d_6), $^{13}C\{^{1}H\}$ NMR (151 MHz, DMSO-d_6) and ^{19}F NMR (565 MHz, $CDCl_3$) spectra for compound **1bu**.

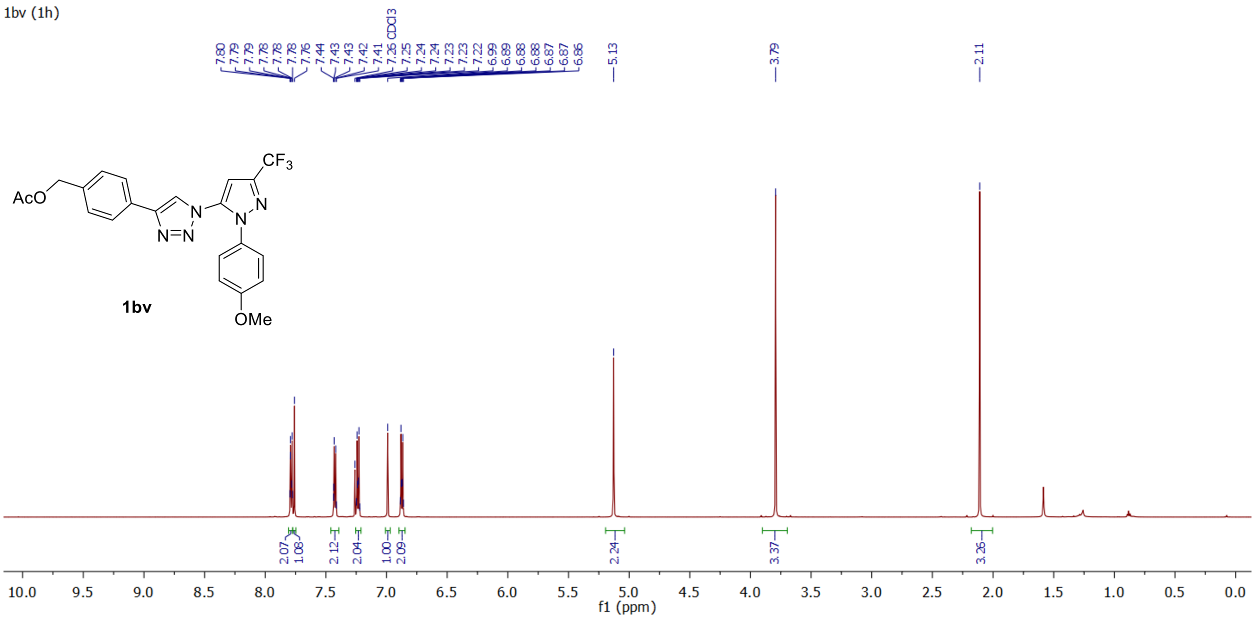

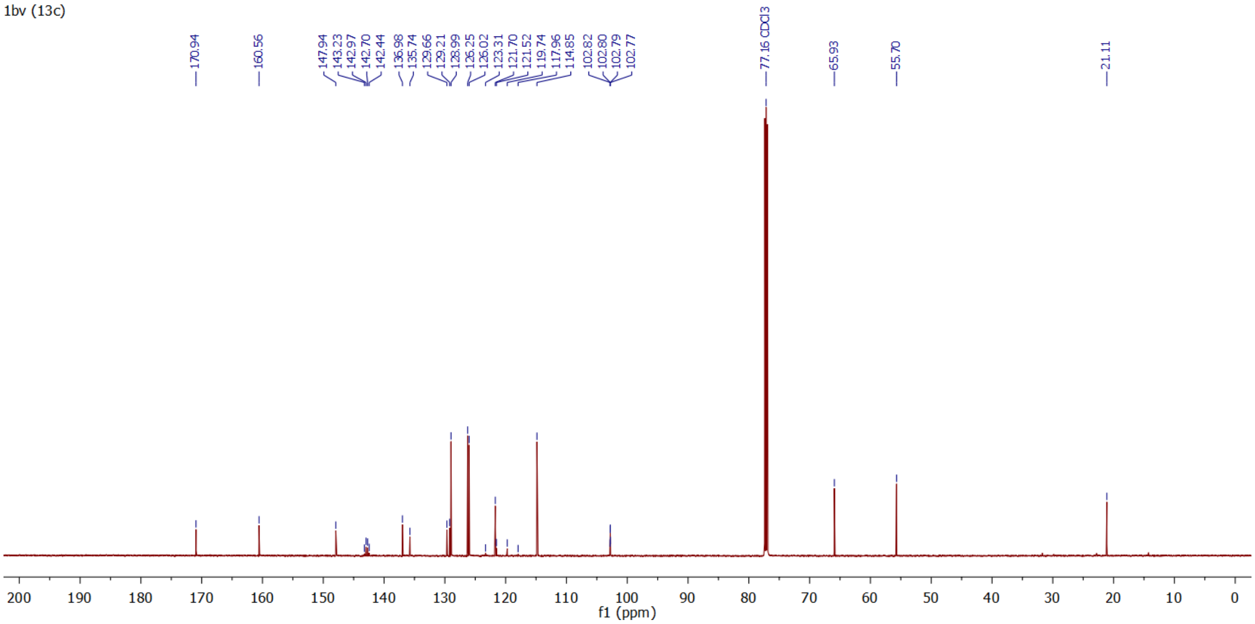

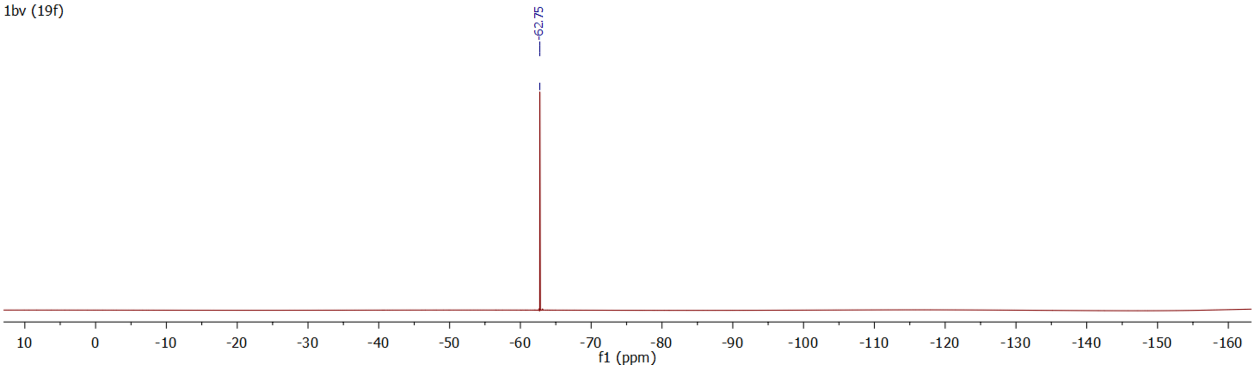

Fig S29. ^{1}H NMR (600 MHz, $CDCl_3$), $^{13}C\{^{1}H\}$ NMR (151 MHz, $CDCl_3$) and ^{19}F NMR (565 MHz, $CDCl_3$) spectra for compound **1bv**.

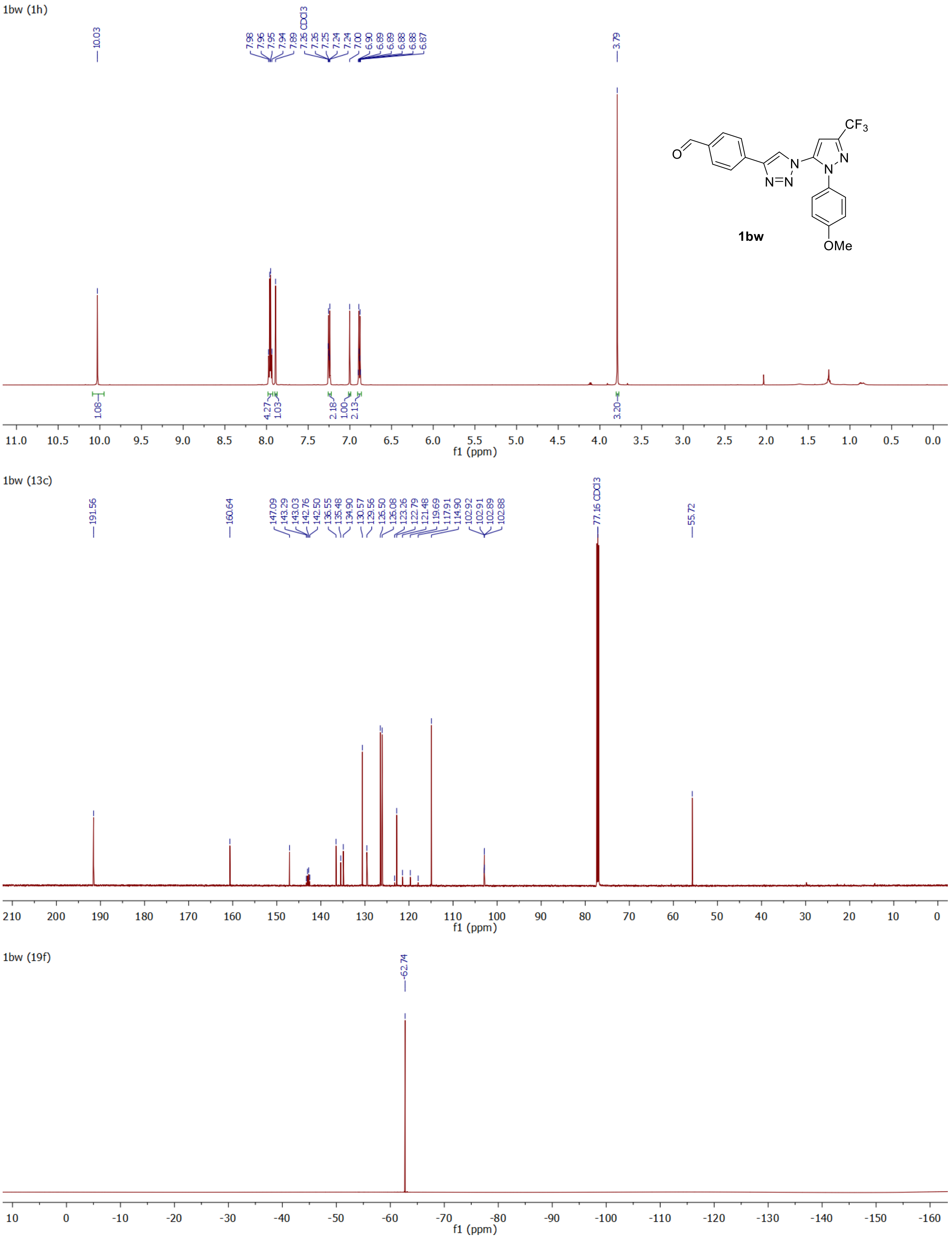

Fig S30. ^{1}H NMR (600 MHz, $CDCl_3$), $^{13}C\{^{1}H\}$ NMR (151 MHz, $CDCl_3$) and ^{19}F NMR (565 MHz, $CDCl_3$) spectra for compound **1bw**.

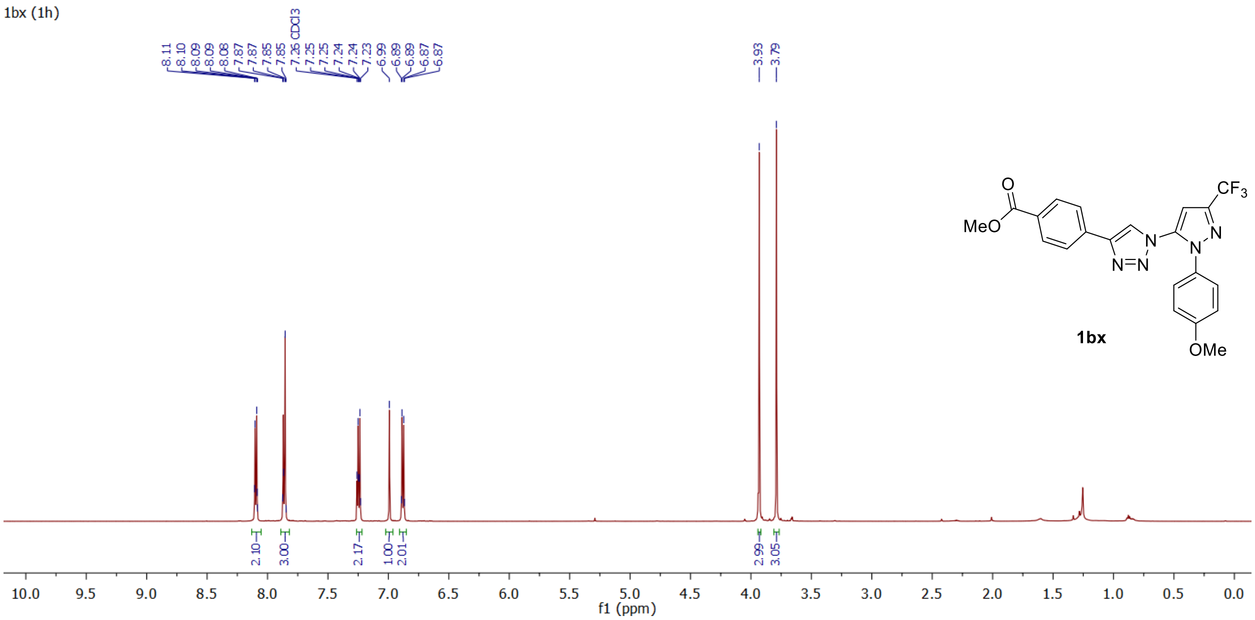

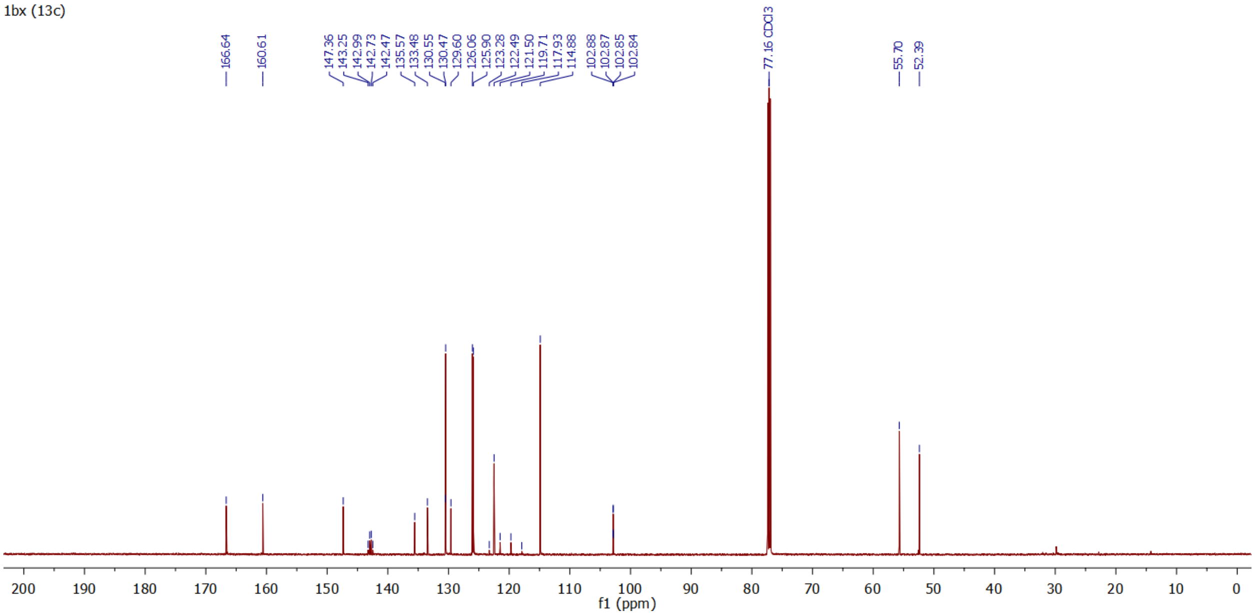

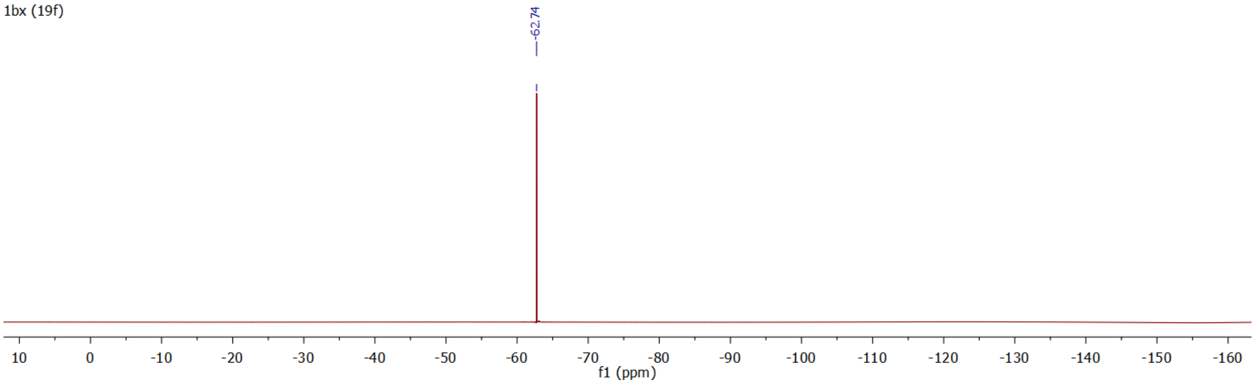

Fig S31. ^{1}H NMR (600 MHz, $CDCl_3$), $^{13}C\{^{1}H\}$ NMR (151 MHz, $CDCl_3$) and ^{19}F NMR (565 MHz, $CDCl_3$) spectra for compound **1bx**.

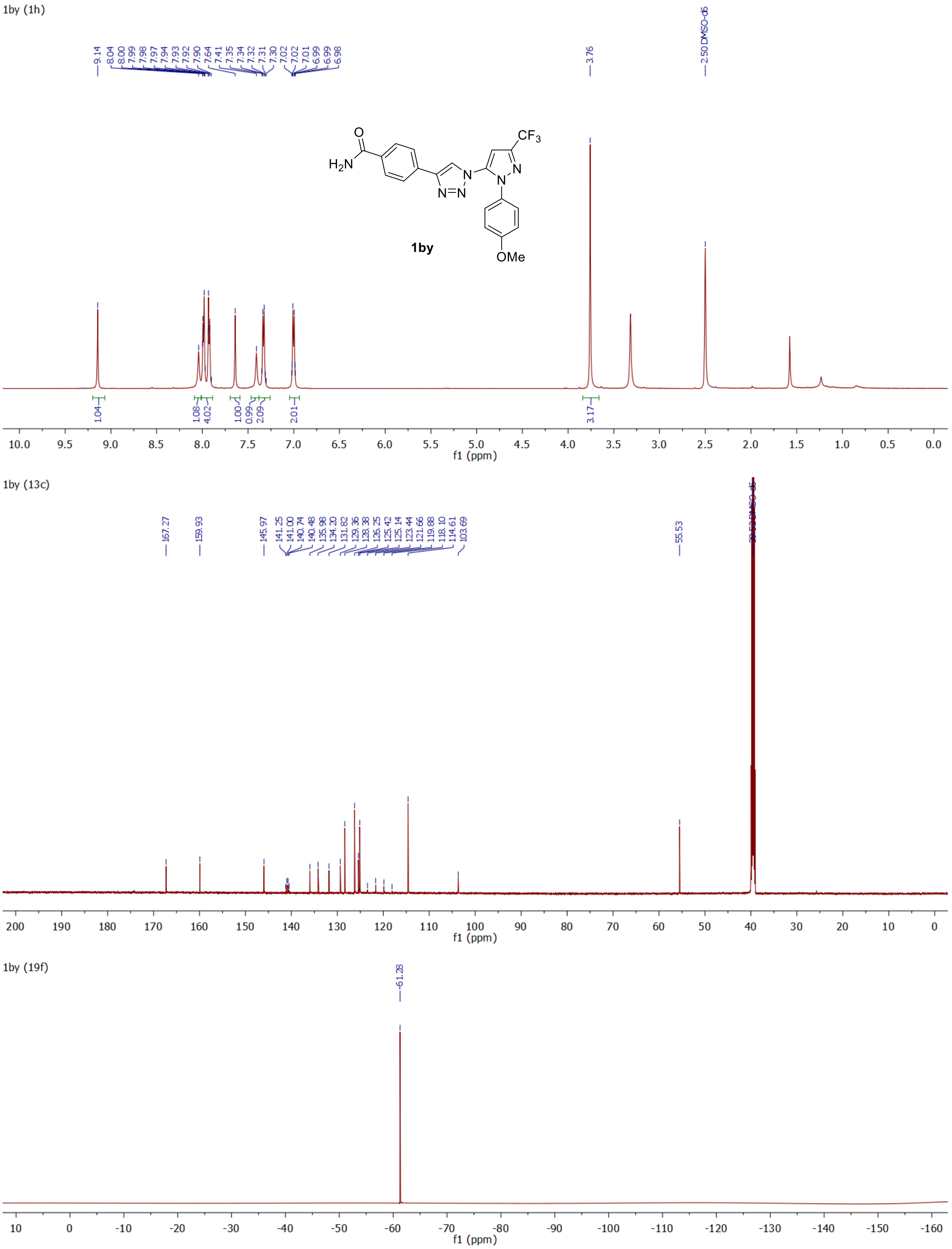

Fig S32. ^{1}H NMR (600 MHz, DMSO-d_6), $^{13}C\{^{1}H\}$ NMR (151 MHz, DMSO-d_6) and ^{19}F NMR (565 MHz, DMSO-d_6) spectra for compound **1by**.

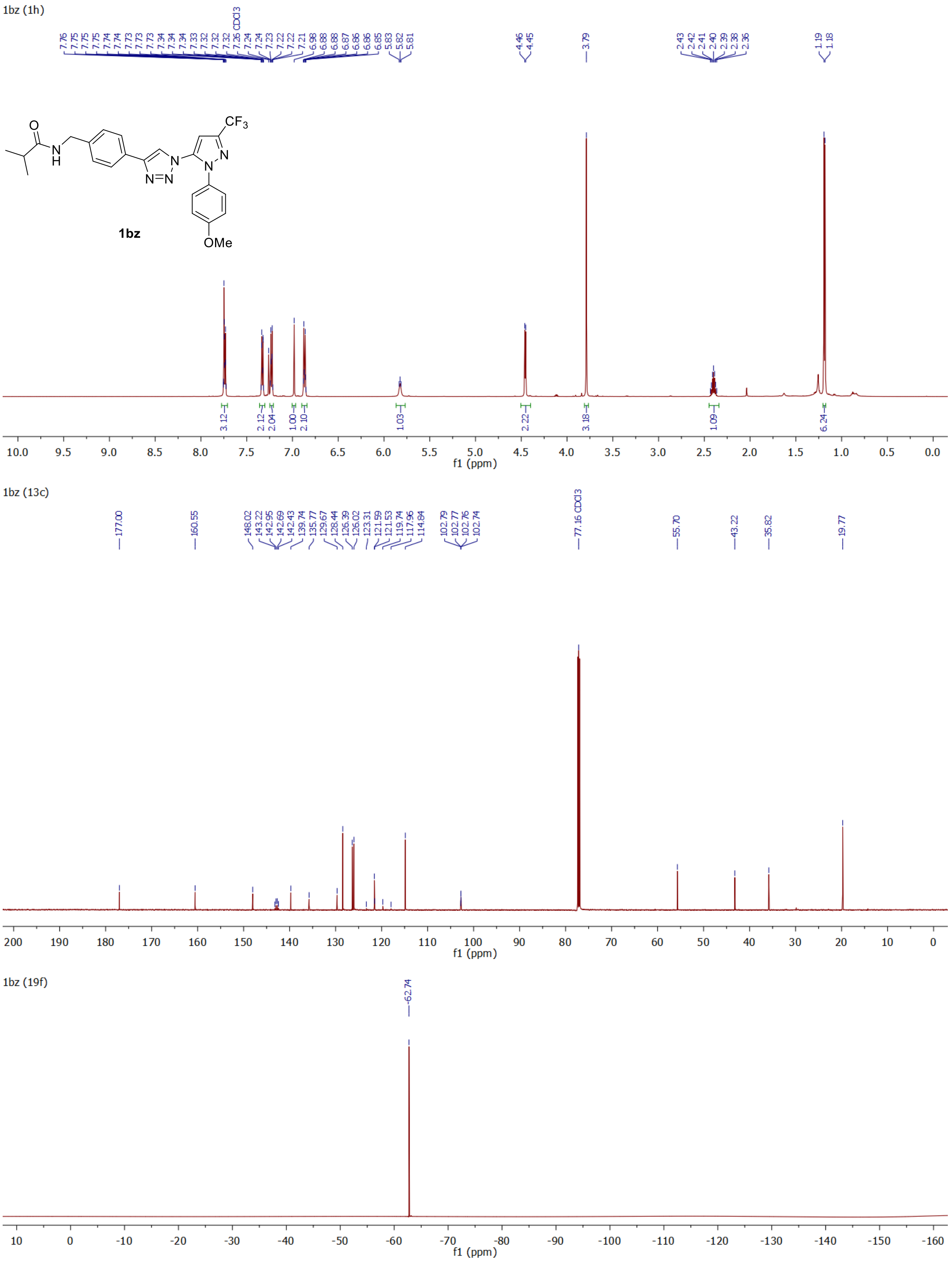

Fig S33. ^{1}H NMR (600 MHz, $CDCl_3$), $^{13}C\{^{1}H\}$ NMR (151 MHz, $CDCl_3$) and ^{19}F NMR (565 MHz, $CDCl_3$) spectra for compound **1bz**.

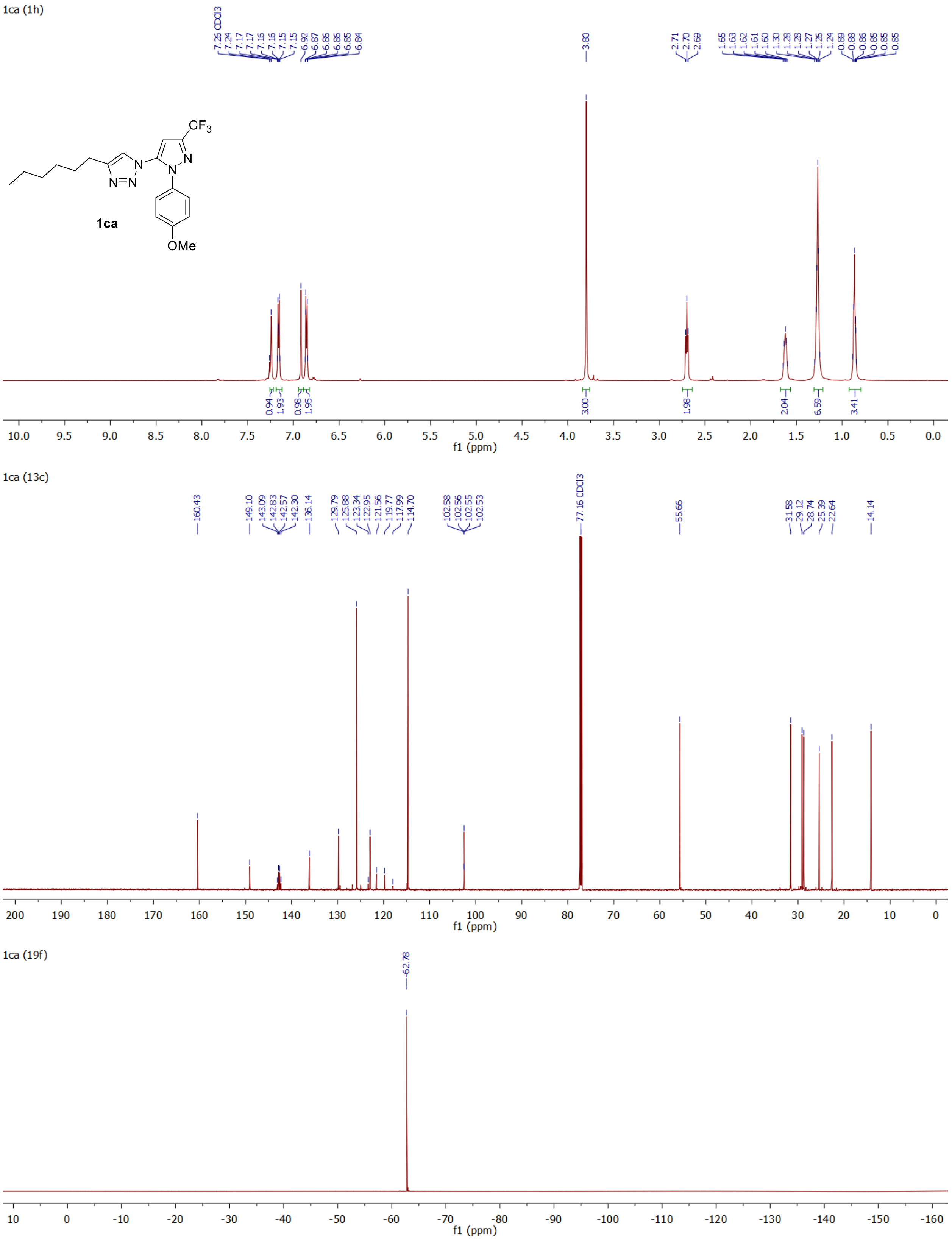

Fig S34. ^{1}H NMR (600 MHz, $CDCl_3$), $^{13}C\{^{1}H\}$ NMR (151 MHz, $CDCl_3$) and ^{19}F NMR (565 MHz, $CDCl_3$) spectra for compound **1ca**.

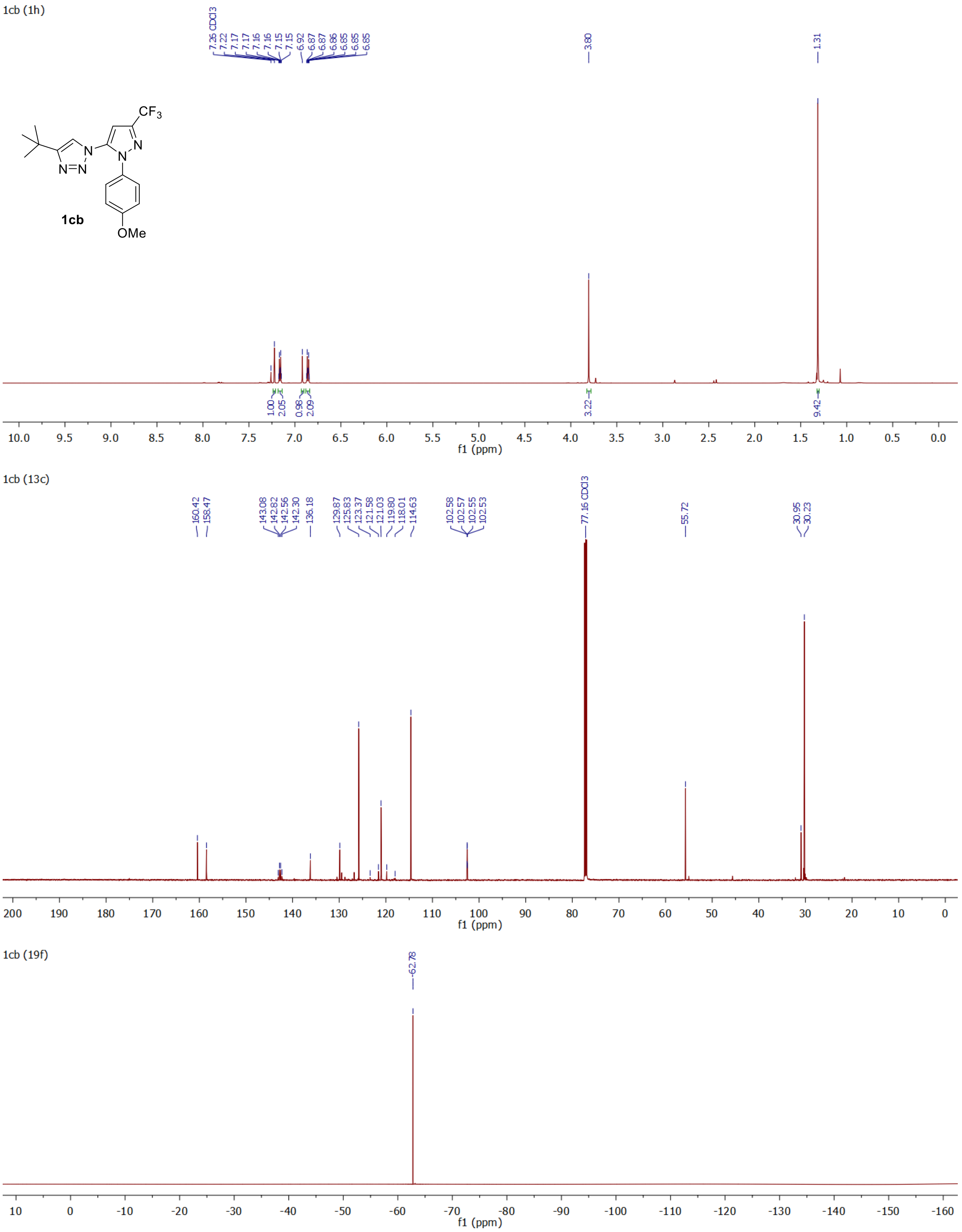

Fig S35. ^{1}H NMR (600 MHz, $CDCl_3$), $^{13}C\{^{1}H\}$ NMR (151 MHz, $CDCl_3$) and ^{19}F NMR (565 MHz, $CDCl_3$) spectra for compound **1cb**.

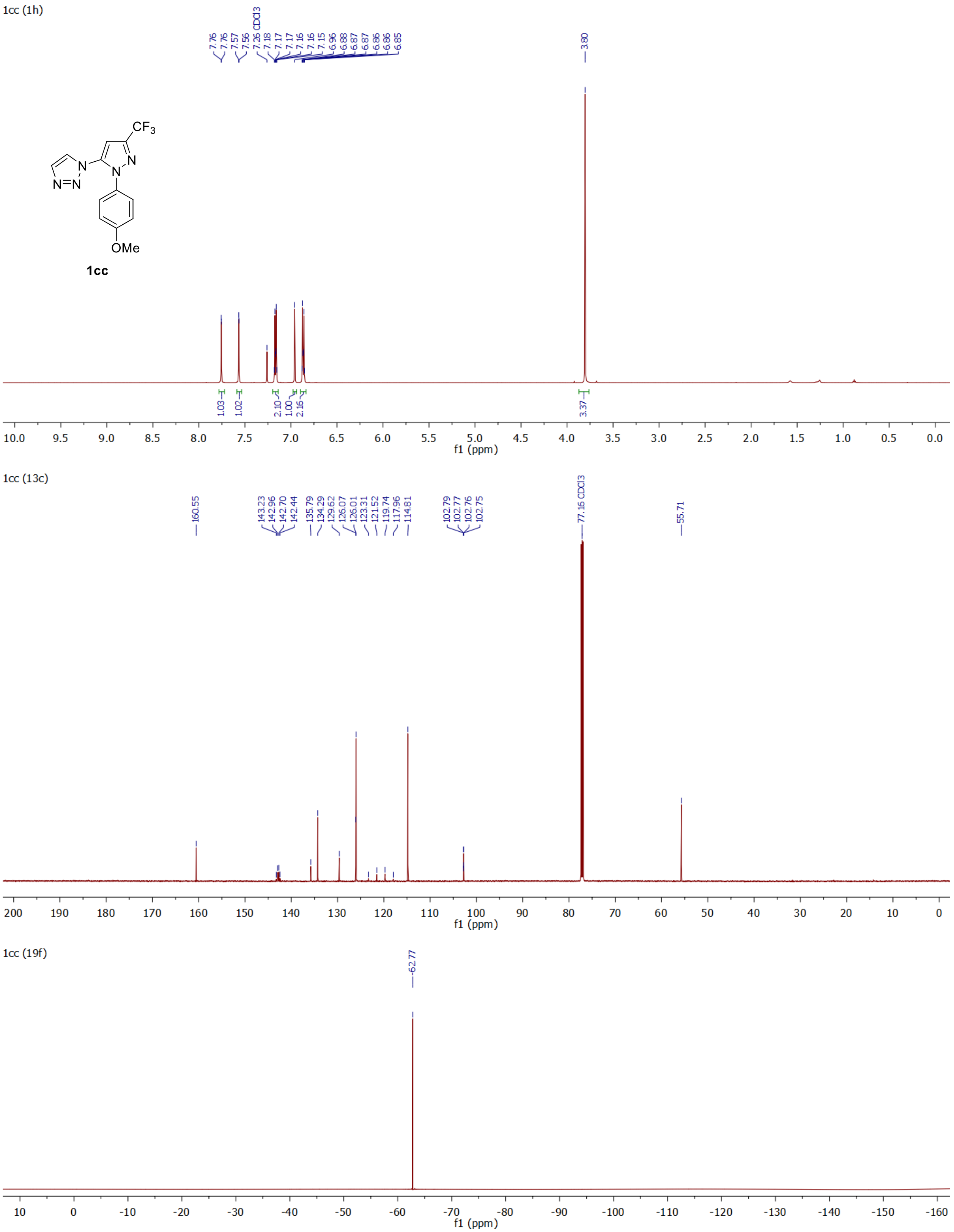

Fig S36. ^{1}H NMR (600 MHz, $CDCl_3$), $^{13}C\{^{1}H\}$ NMR (151 MHz, $CDCl_3$) and ^{19}F NMR (565 MHz, $CDCl_3$) spectra for compound **1cc**.

1cd (1h)

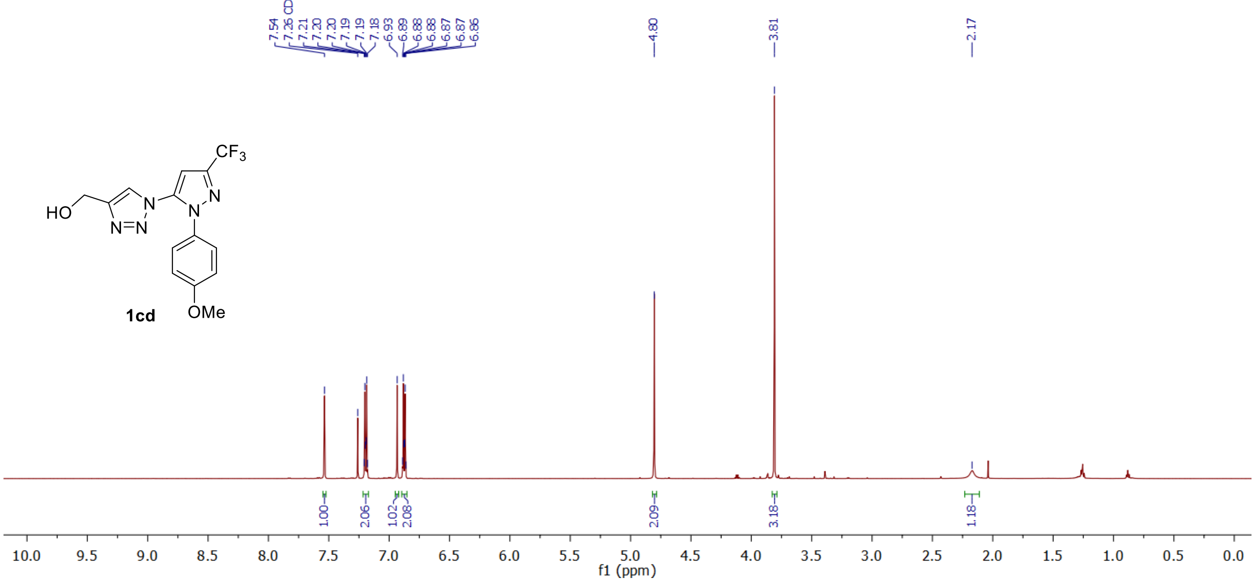

1cd (13c)

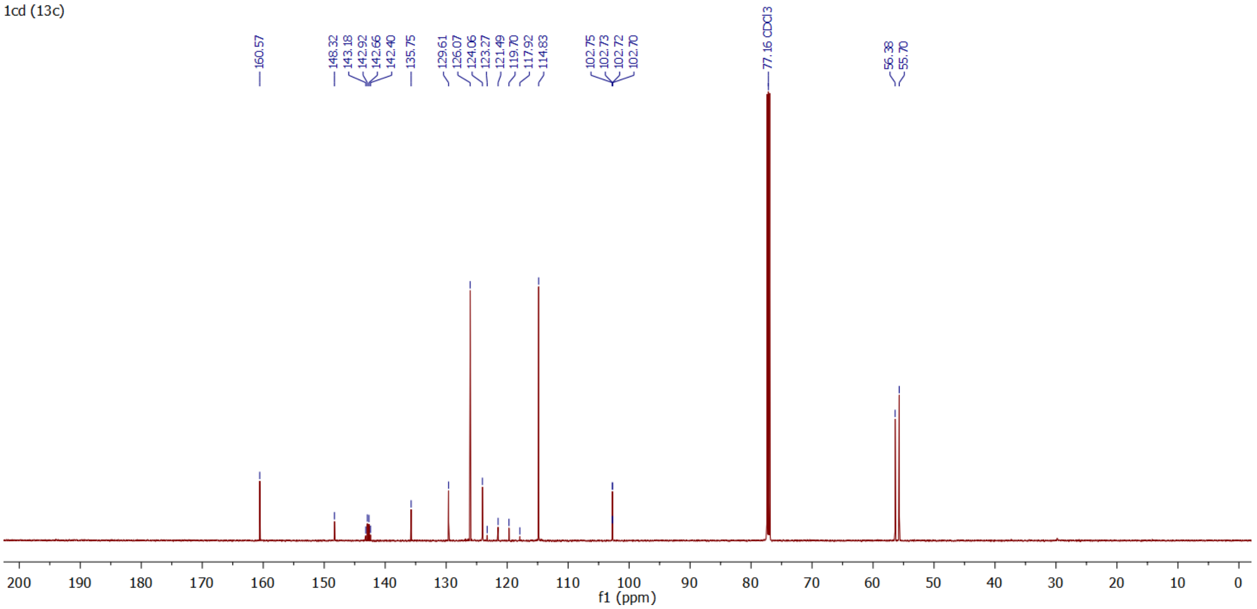

1cd (19f)

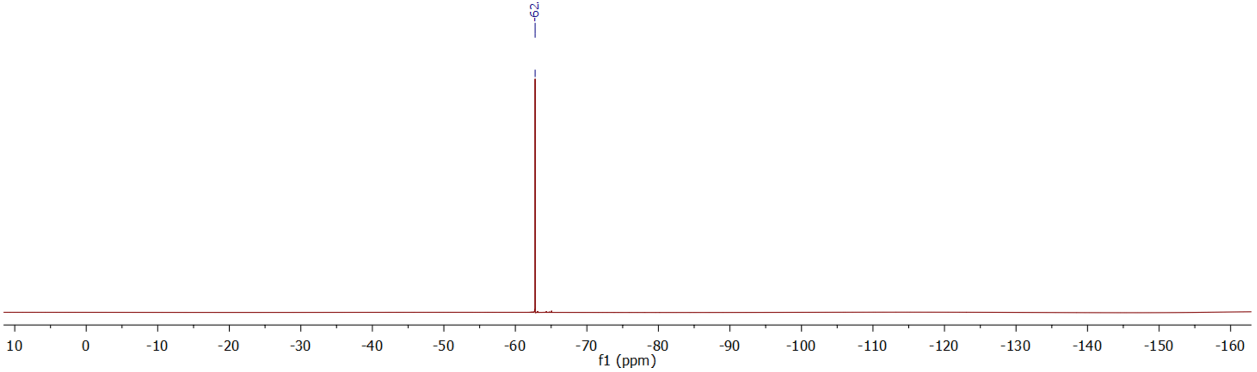

Fig S37. ^{1}H NMR (600 MHz, $CDCl_3$), $^{13}C\{^{1}H\}$ NMR (151 MHz, $CDCl_3$) and ^{19}F NMR (565 MHz, $CDCl_3$) spectra for compound **1cd**.

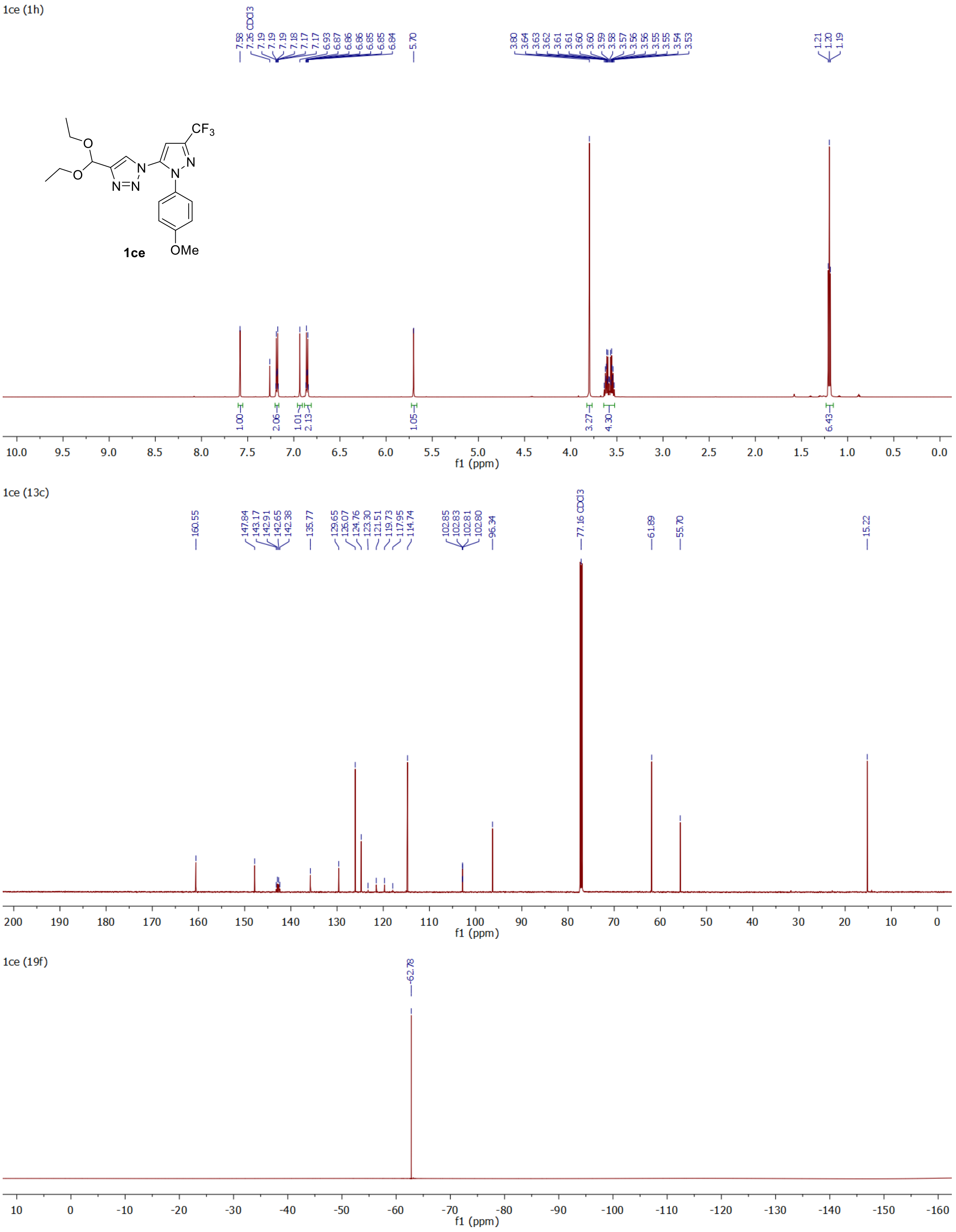

Fig S38. ^{1}H NMR (600 MHz, $CDCl_3$), $^{13}C\{^1H\}$ NMR (151 MHz, $CDCl_3$) and ^{19}F NMR (565 MHz, $CDCl_3$) spectra for compound **1ce**.

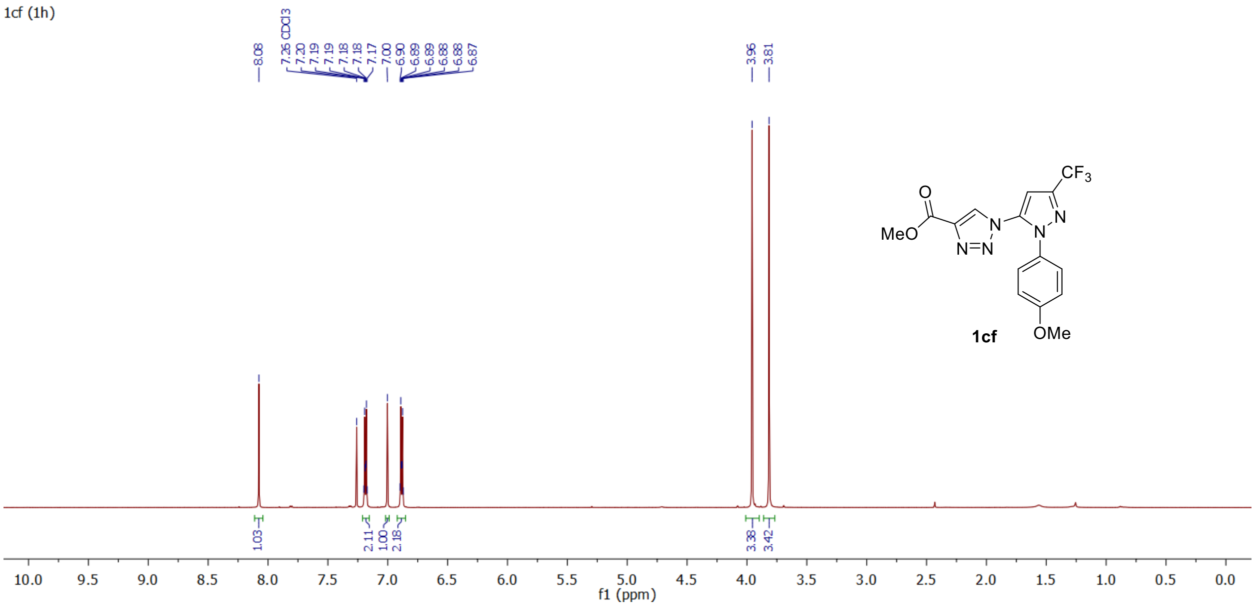

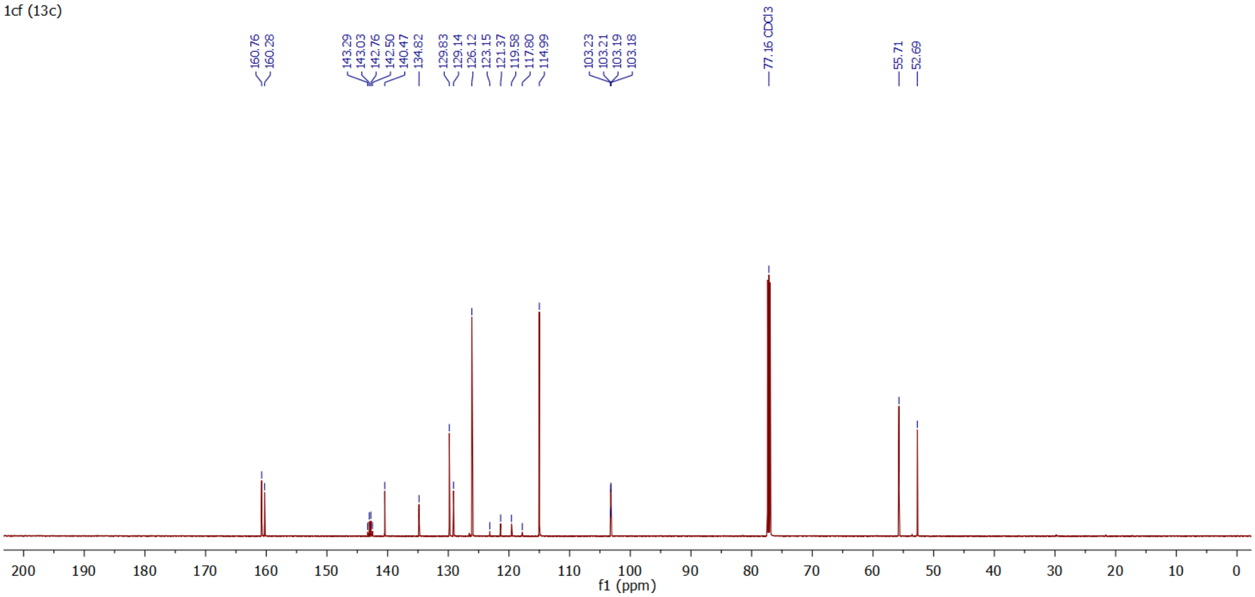

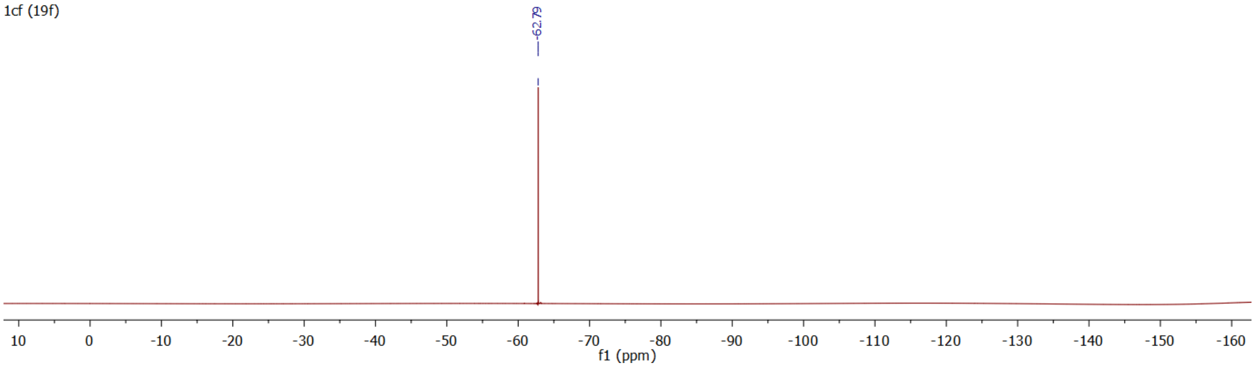

Fig S39. ^{1}H NMR (600 MHz, $CDCl_3$), $^{13}C\{^{1}H\}$ NMR (151 MHz, $CDCl_3$) and ^{19}F NMR (565 MHz, $CDCl_3$) spectra for compound **1cf**.

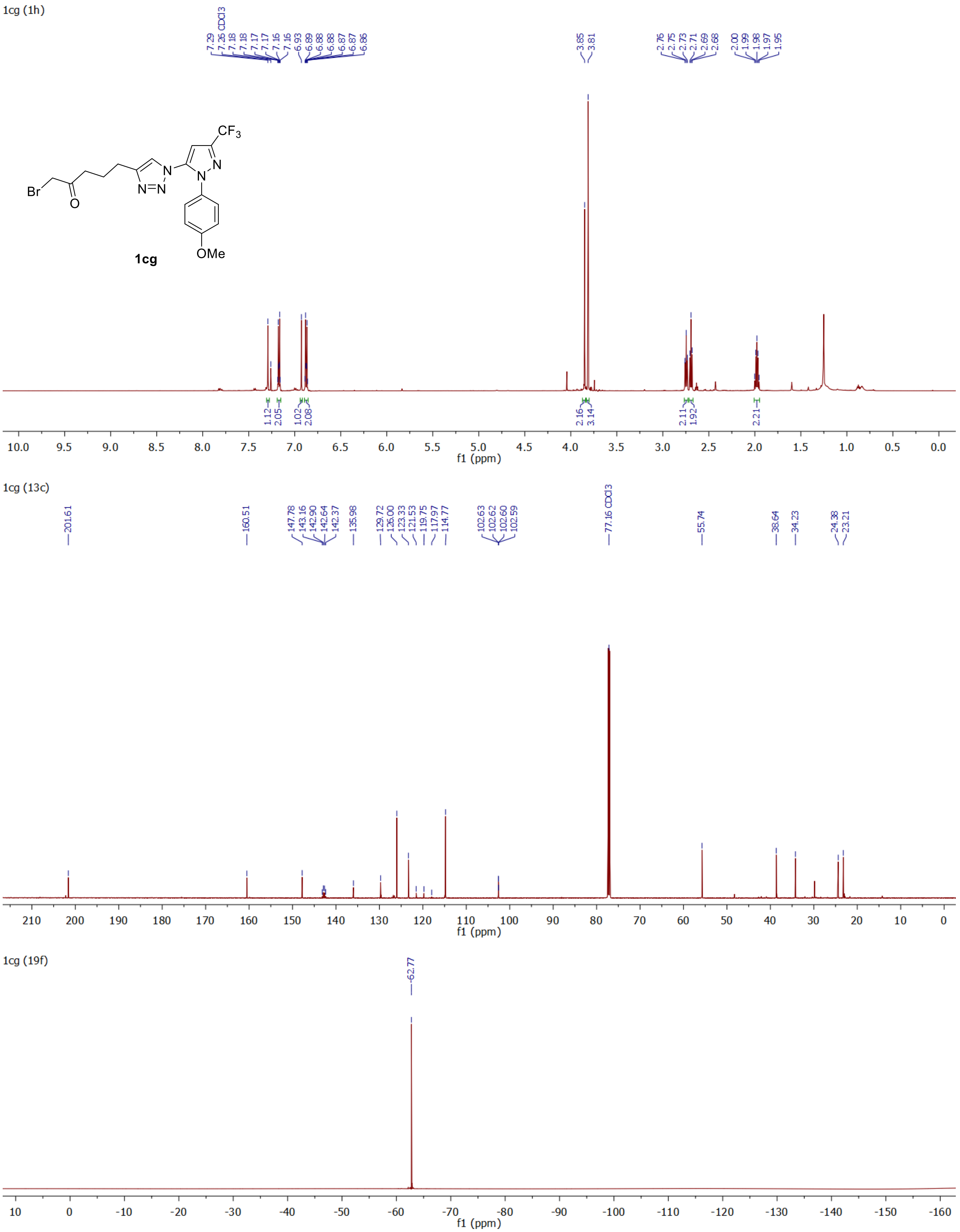

Fig S40. ^{1}H NMR (600 MHz, $CDCl_3$), $^{13}C\{^{1}H\}$ NMR (151 MHz, $CDCl_3$) and ^{19}F NMR (565 MHz, $CDCl_3$) spectra for compound **1cg**.

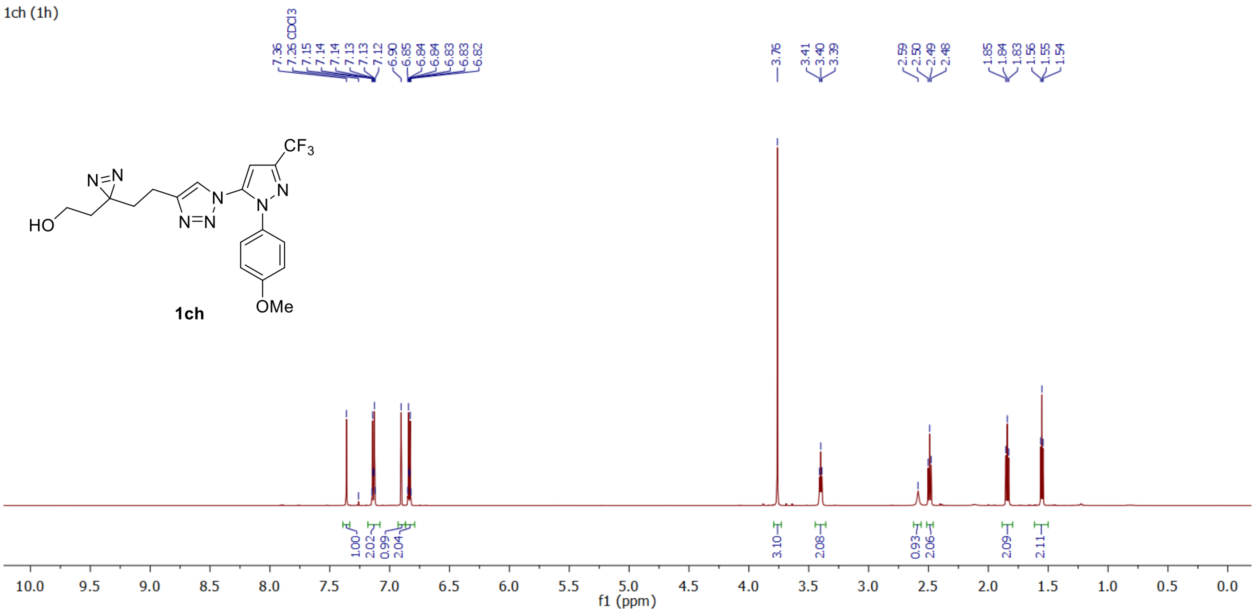

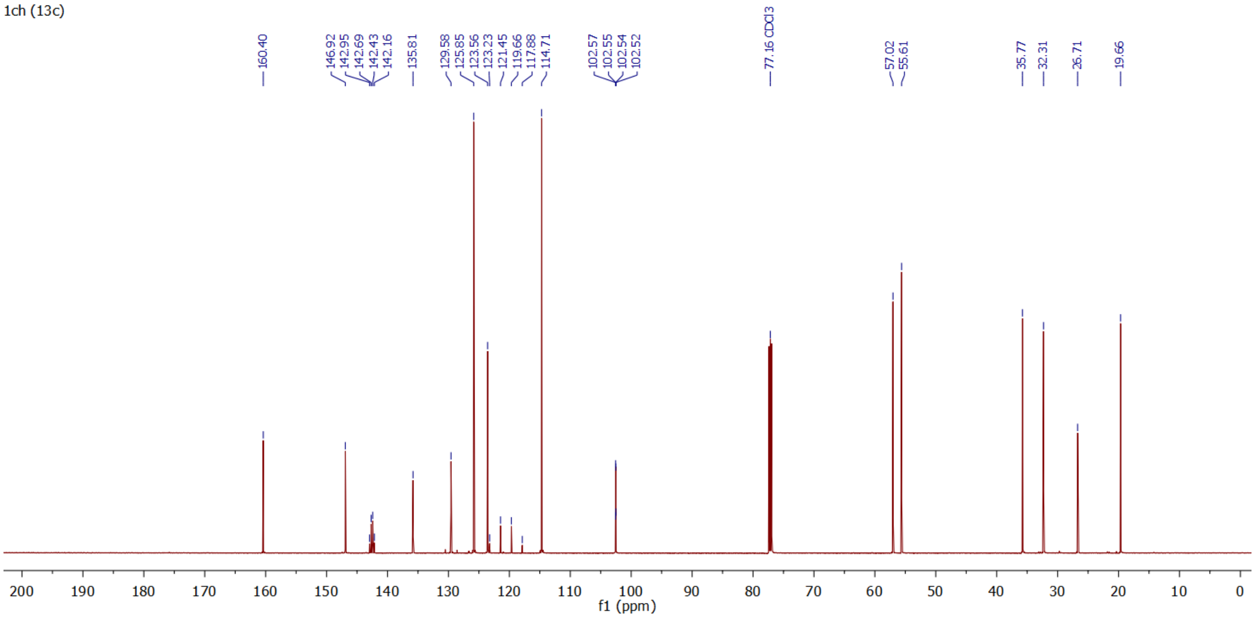

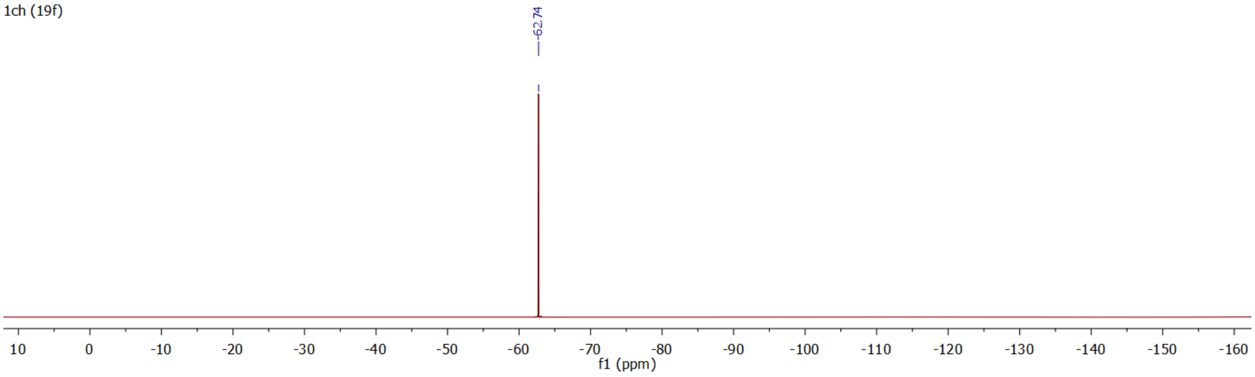

Fig S41. ^{1}H NMR (600 MHz, $CDCl_3$), $^{13}C\{^{1}H\}$ NMR (151 MHz, $CDCl_3$) and ^{19}F NMR (565 MHz, $CDCl_3$) spectra for compound **1ch**.

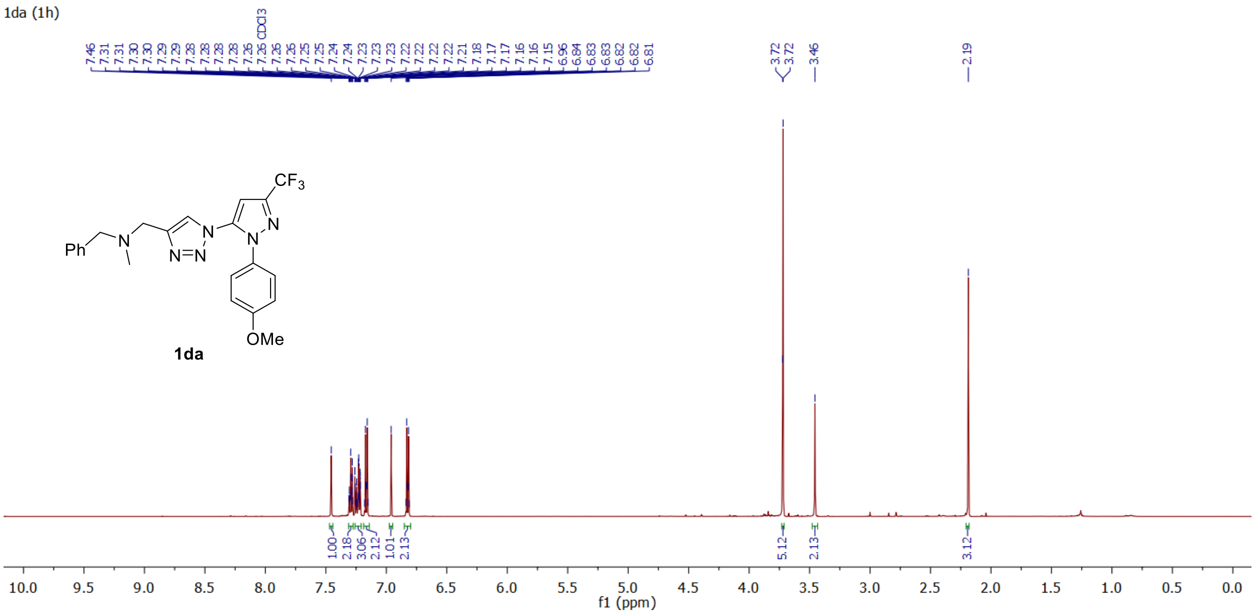

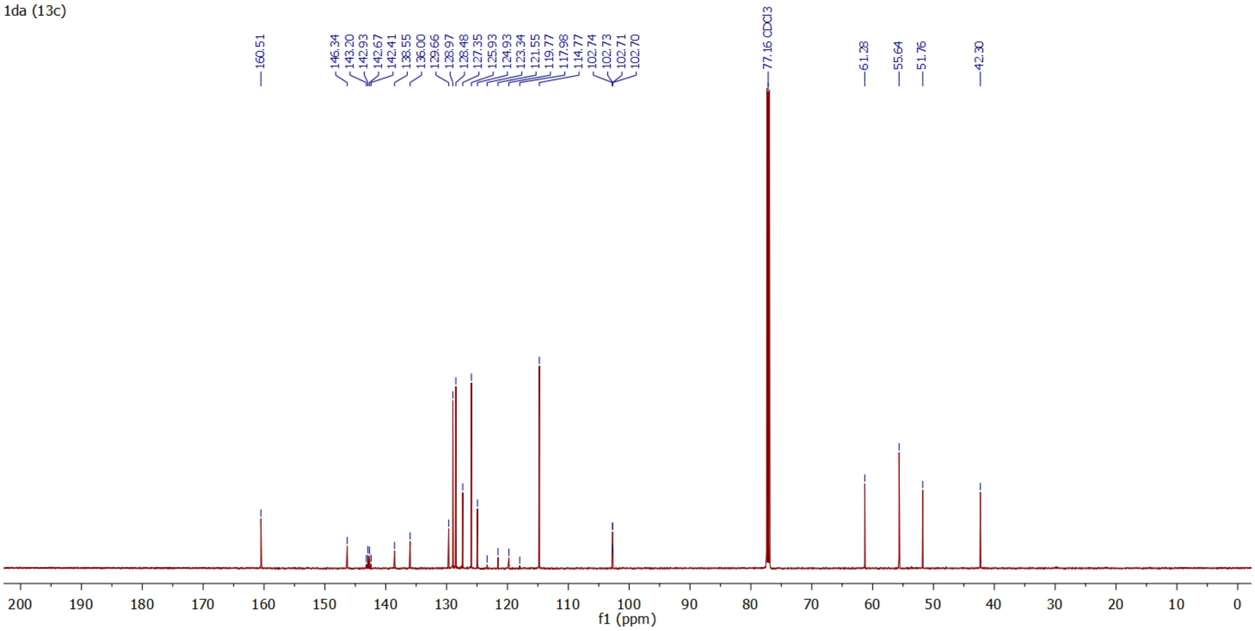

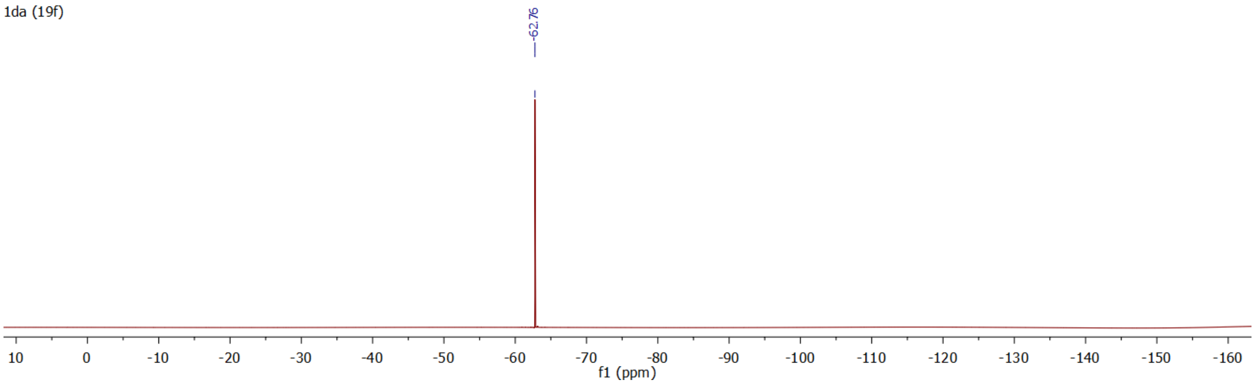

Fig S42. ^1H NMR (600 MHz, $CDCl_3$), $^{13}C\{^1H\}$ NMR (151 MHz, $CDCl_3$) and ^{19}F NMR (565 MHz, $CDCl_3$) spectra for compound **1da**.

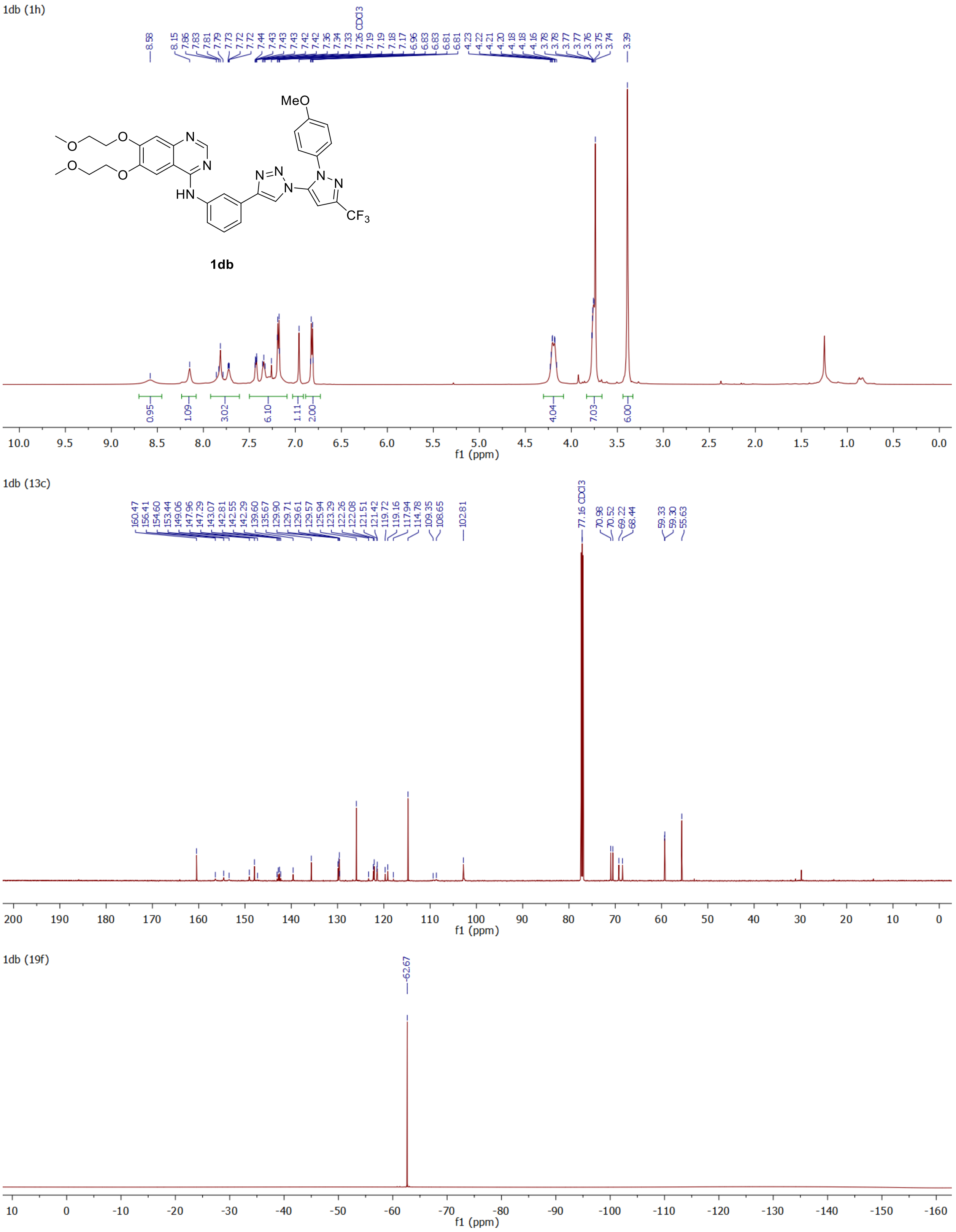

Fig S43. ^{1}H NMR (600 MHz, $CDCl_3$), $^{13}C\{^{1}H\}$ NMR (151 MHz, $CDCl_3$) and ^{19}F NMR (565 MHz, $CDCl_3$) spectra for compound **1db**.

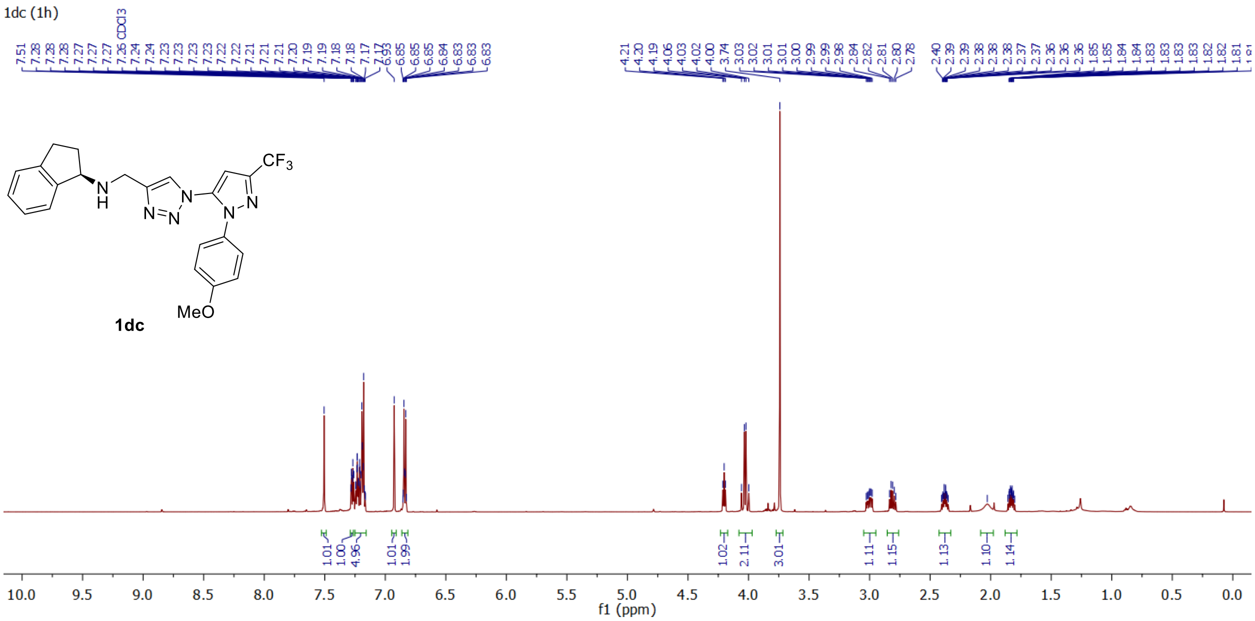

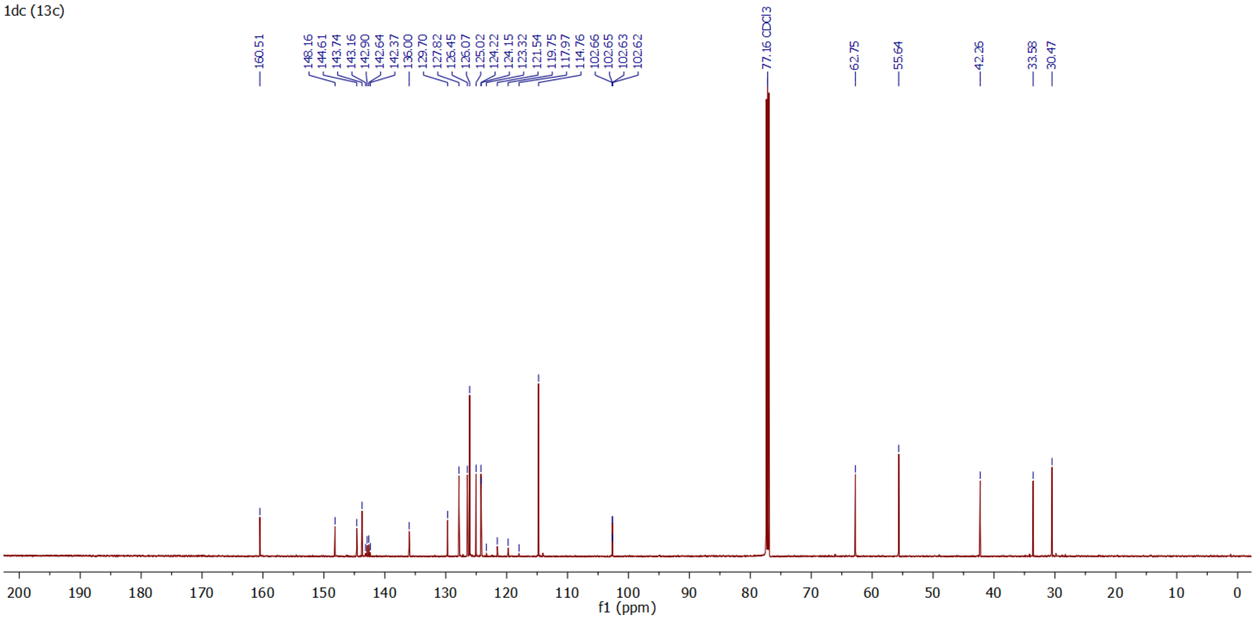

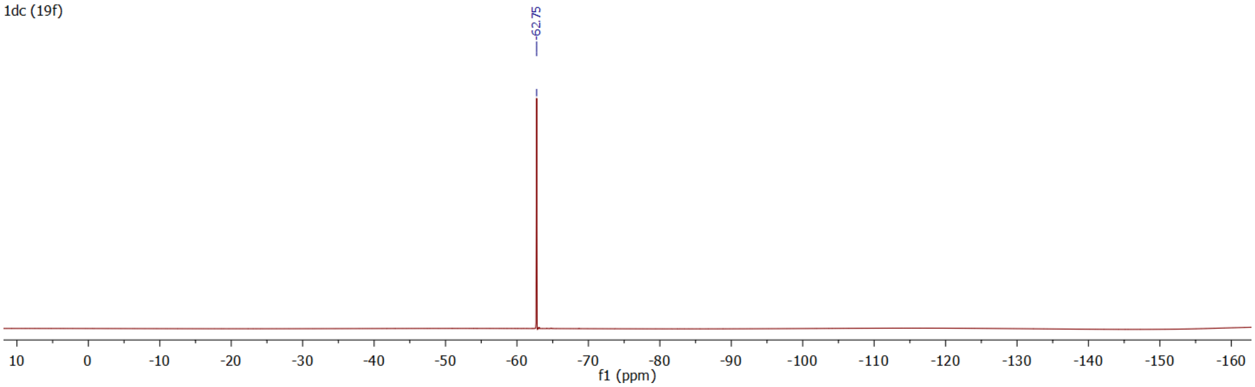

Fig S44. ^{1}H NMR (600 MHz, $CDCl_3$), $^{13}C\{^{1}H\}$ NMR (151 MHz, $CDCl_3$) and ^{19}F NMR (565 MHz, $CDCl_3$) spectra for compound **1dc**.

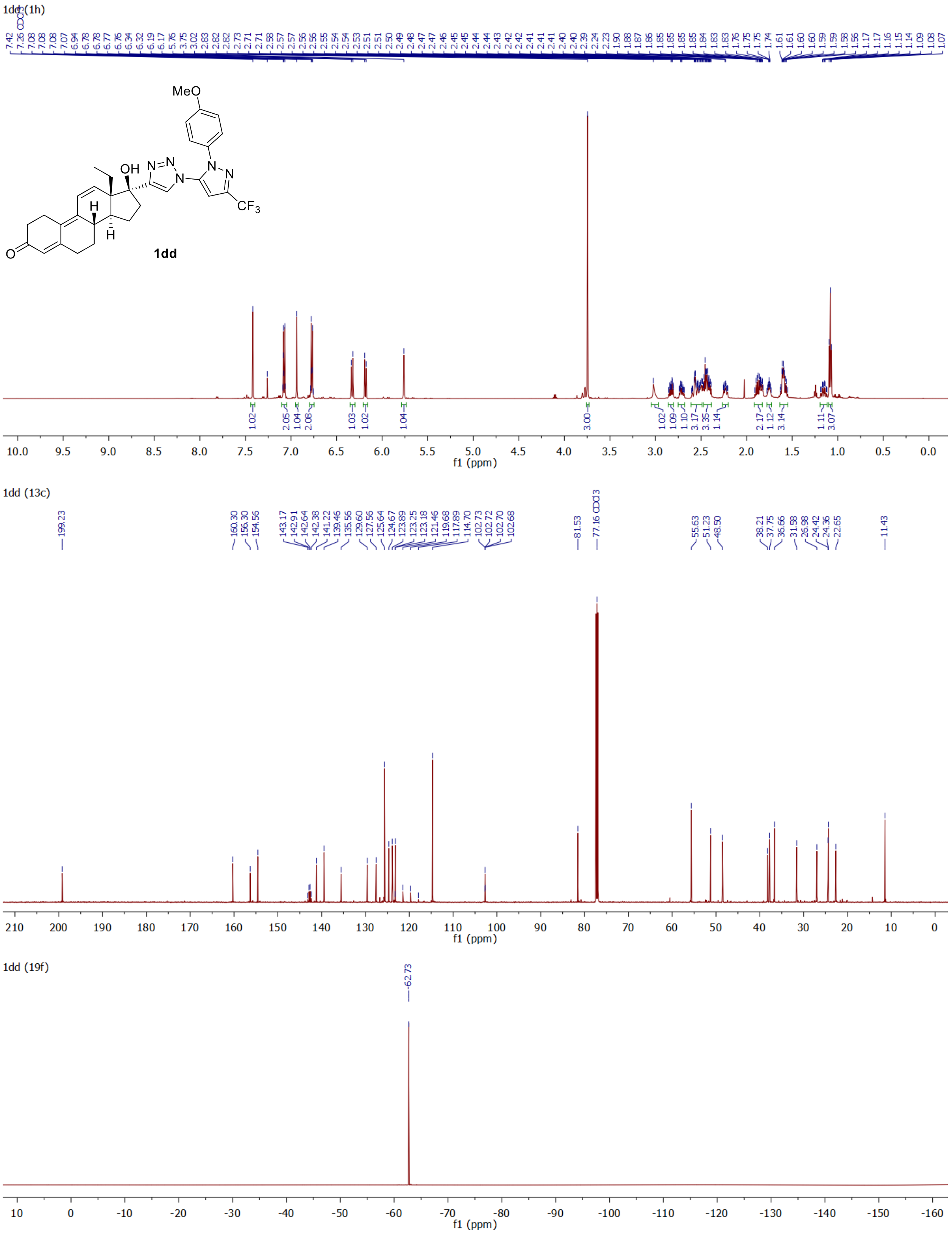

Fig S45. ^{1}H NMR (600 MHz, $CDCl_3$), $^{13}C\{^{1}H\}$ NMR (151 MHz, $CDCl_3$) and ^{19}F NMR (565 MHz, $CDCl_3$) spectra for compound **1dd**.

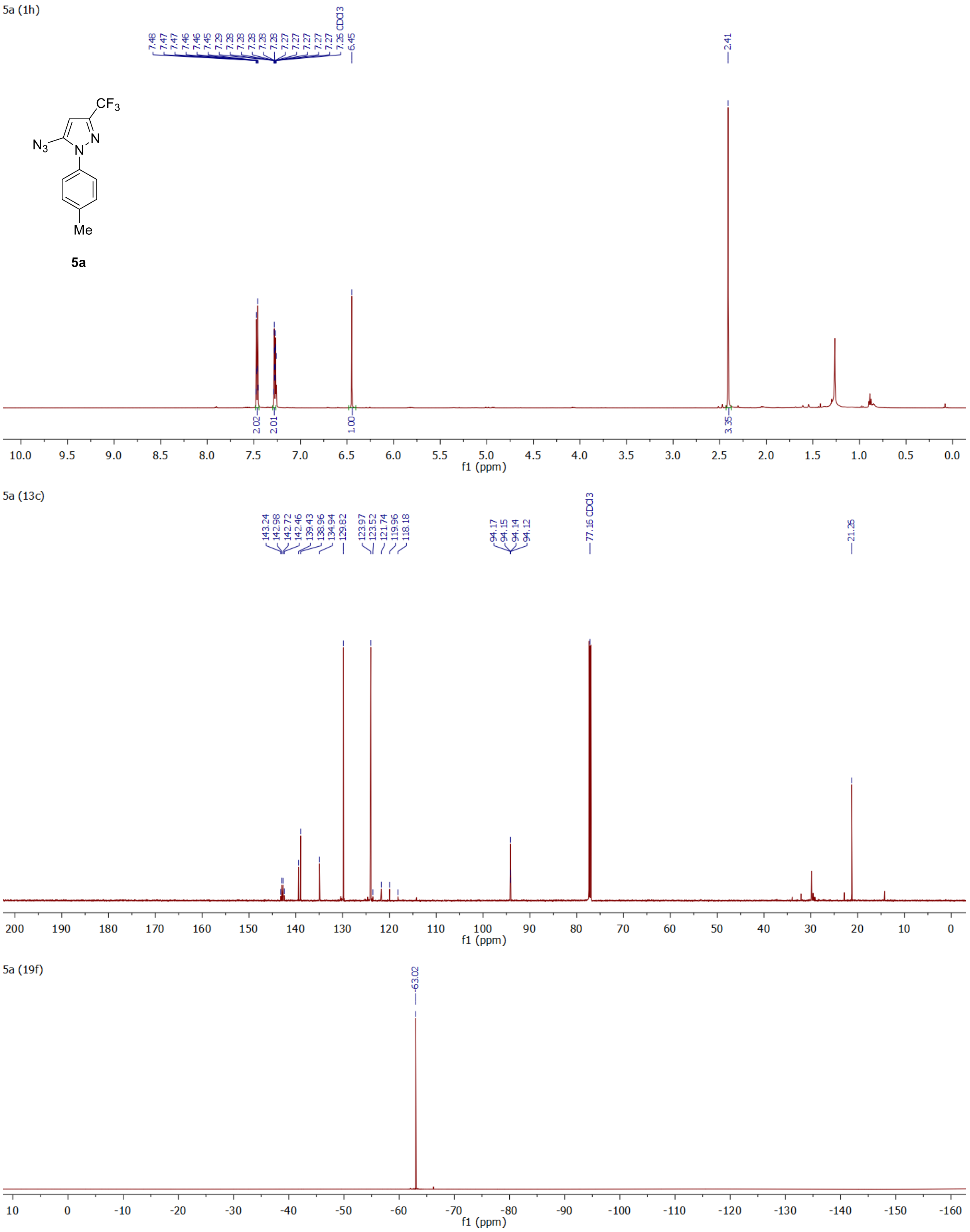

Fig S46. ^{1}H NMR (600 MHz, $CDCl_3$), $^{13}C\{^{1}H\}$ NMR (151 MHz, $CDCl_3$) and ^{19}F NMR (565 MHz, $CDCl_3$) spectra for compound **5a**.

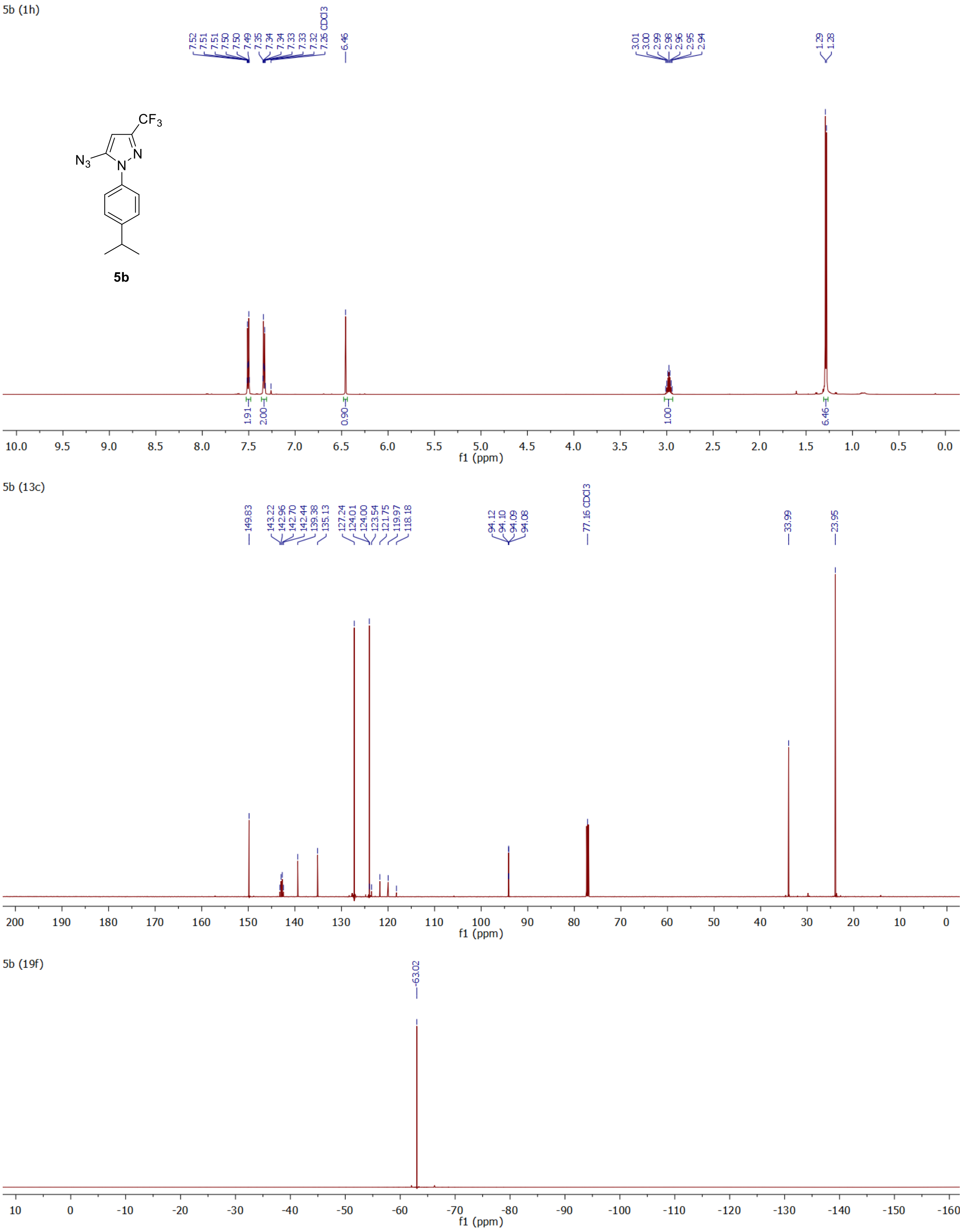

Fig S47. ^{1}H NMR (600 MHz, $CDCl_3$), $^{13}C\{^{1}H\}$ NMR (151 MHz, $CDCl_3$) and ^{19}F NMR (565 MHz, $CDCl_3$) spectra for compound **5b**.

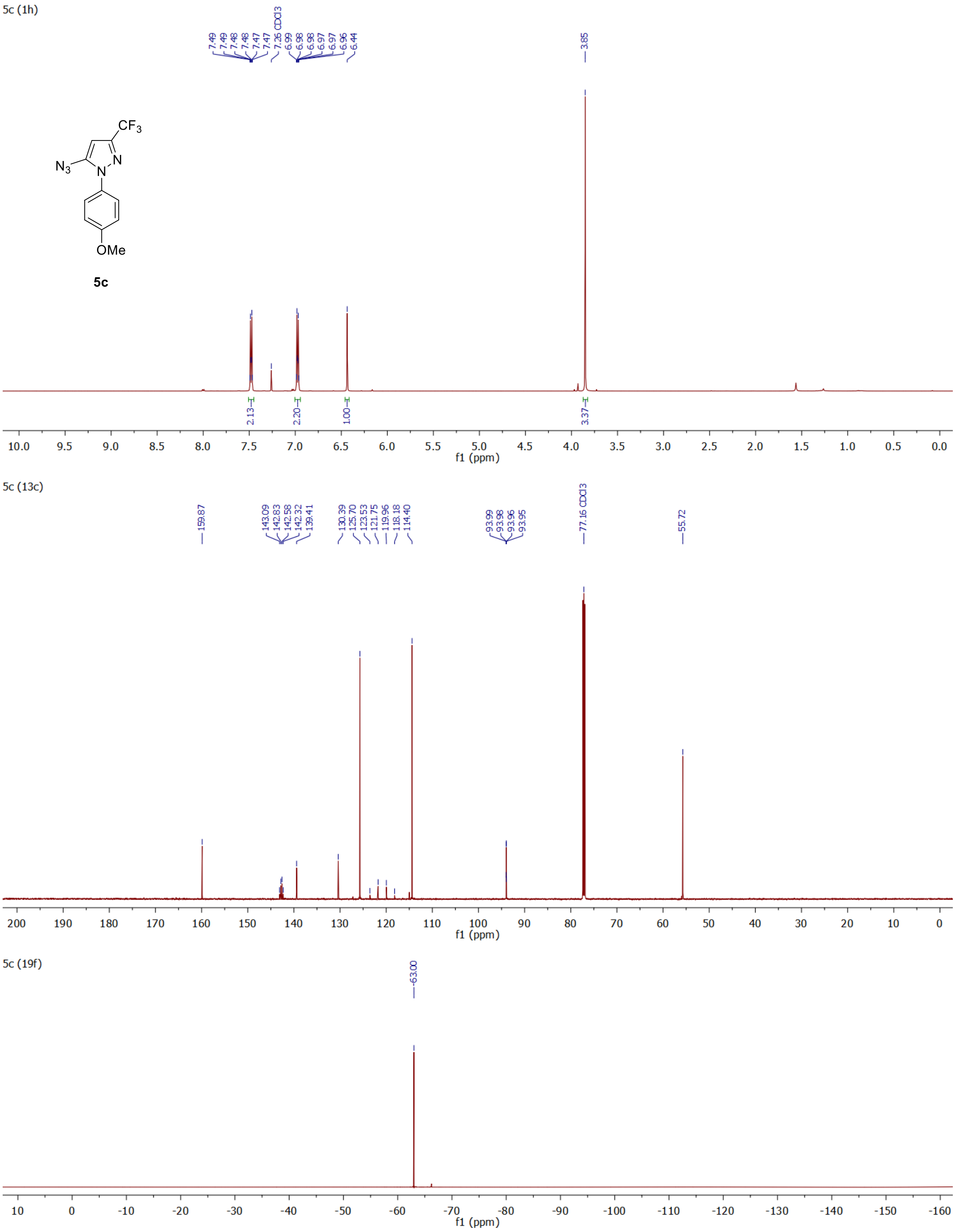

Fig S48. ^{1}H NMR (600 MHz, $CDCl_3$), $^{13}C\{^{1}H\}$ NMR (151 MHz, $CDCl_3$) and ^{19}F NMR (565 MHz, $CDCl_3$) spectra for compound **5c**.

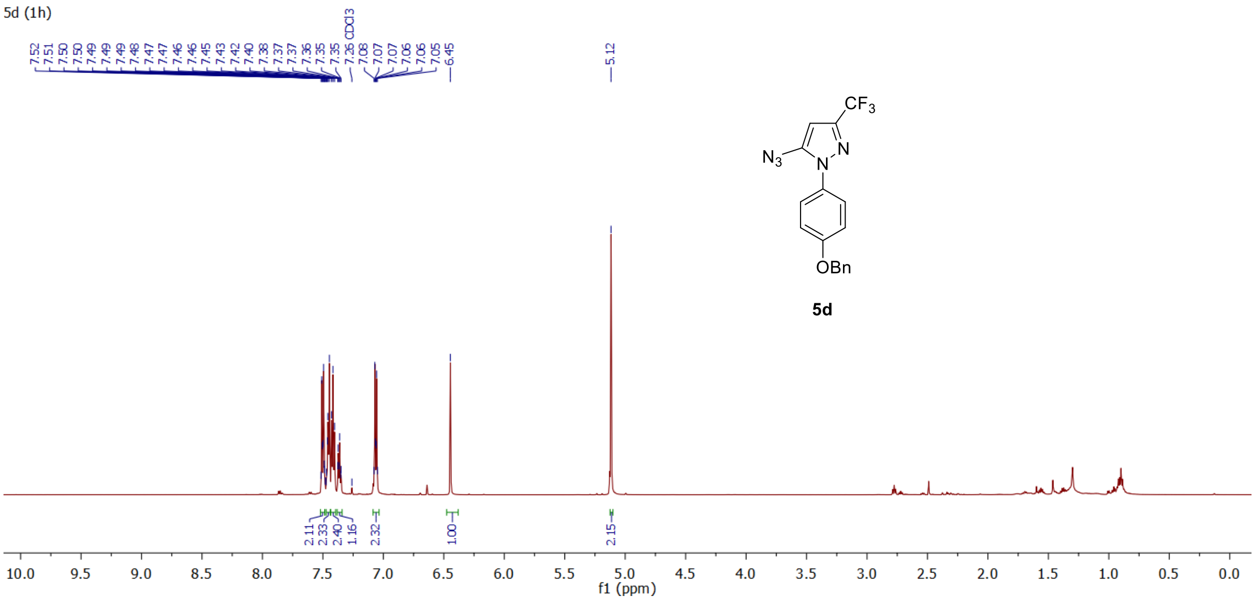

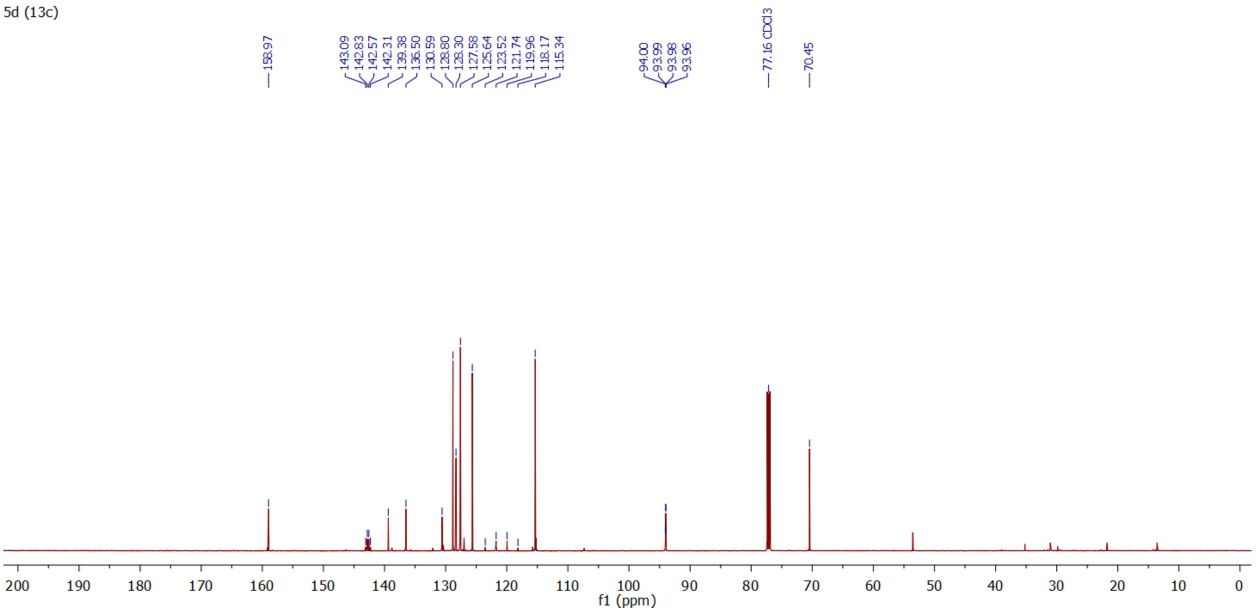

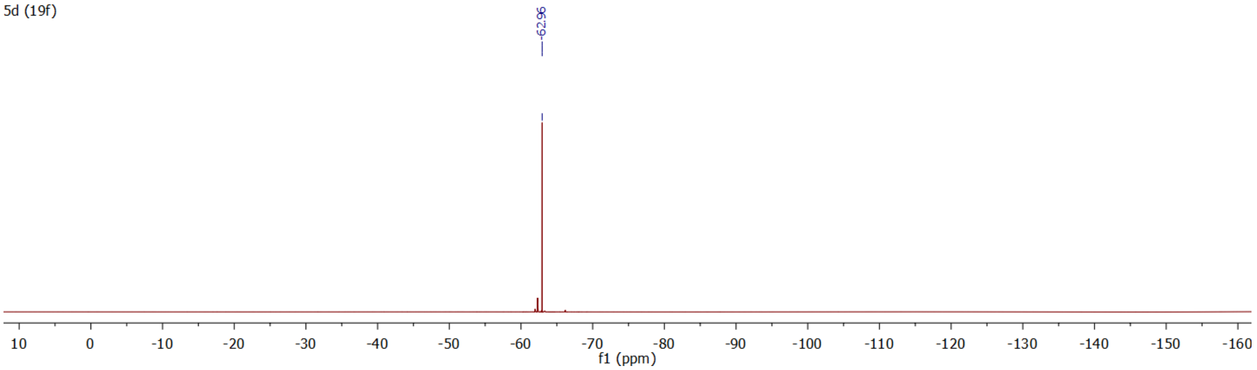

Fig S49. ^{1}H NMR (600 MHz, $CDCl_3$), $^{13}C\{^{1}H\}$ NMR (151 MHz, $CDCl_3$) and ^{19}F NMR (565 MHz, $CDCl_3$) spectra for compound **5d**.

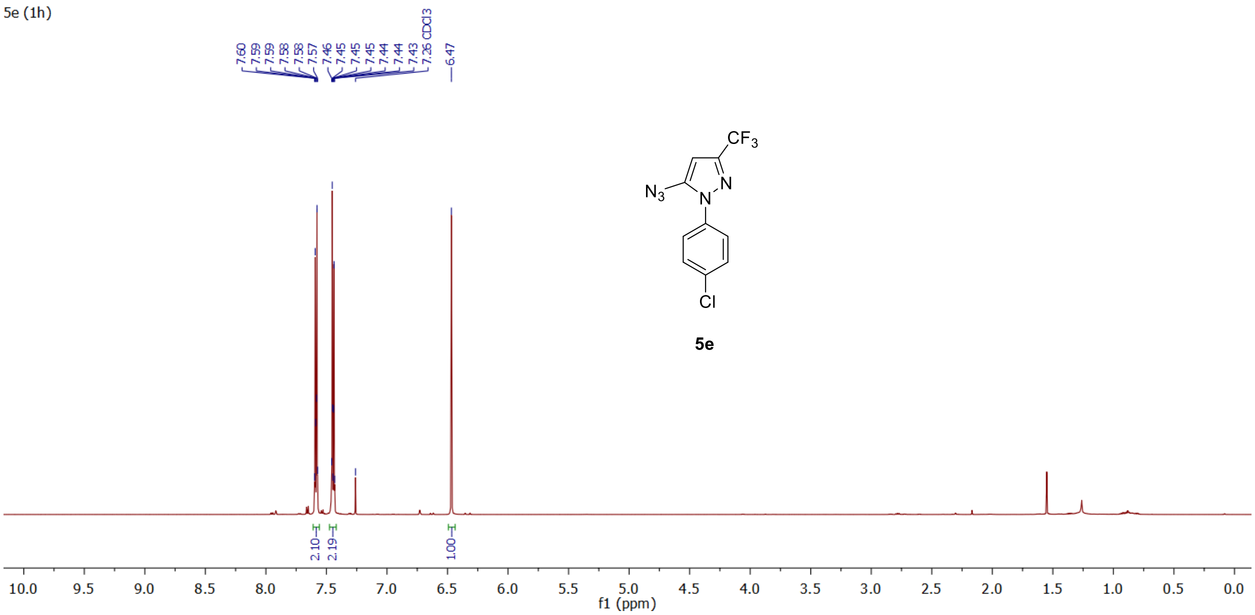

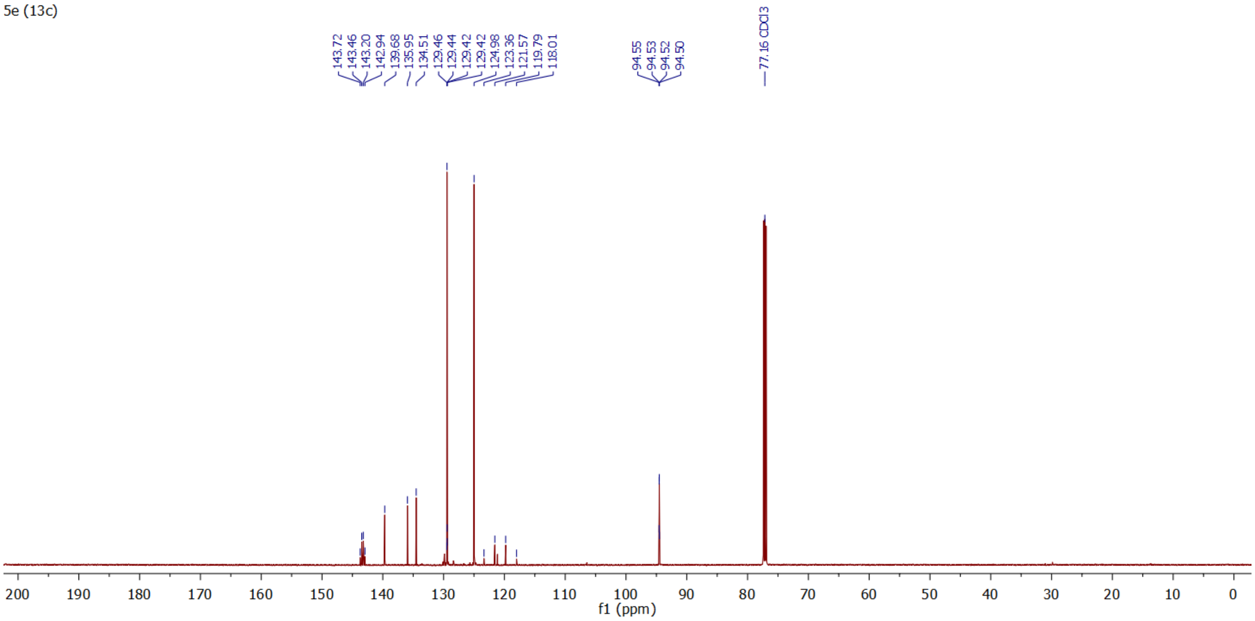

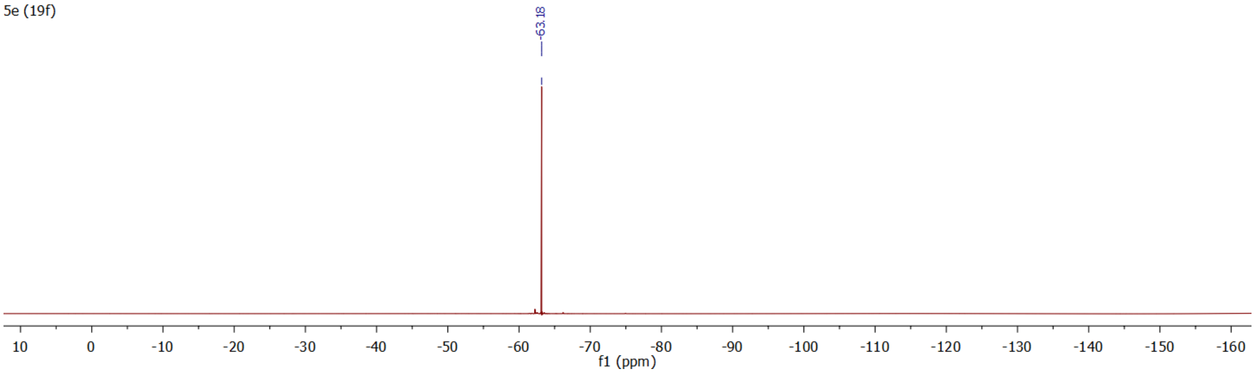

Fig S50. ^{1}H NMR (600 MHz, $CDCl_3$), $^{13}C\{^{1}H\}$ NMR (151 MHz, $CDCl_3$) and ^{19}F NMR (565 MHz, $CDCl_3$) spectra for compound **5e**.

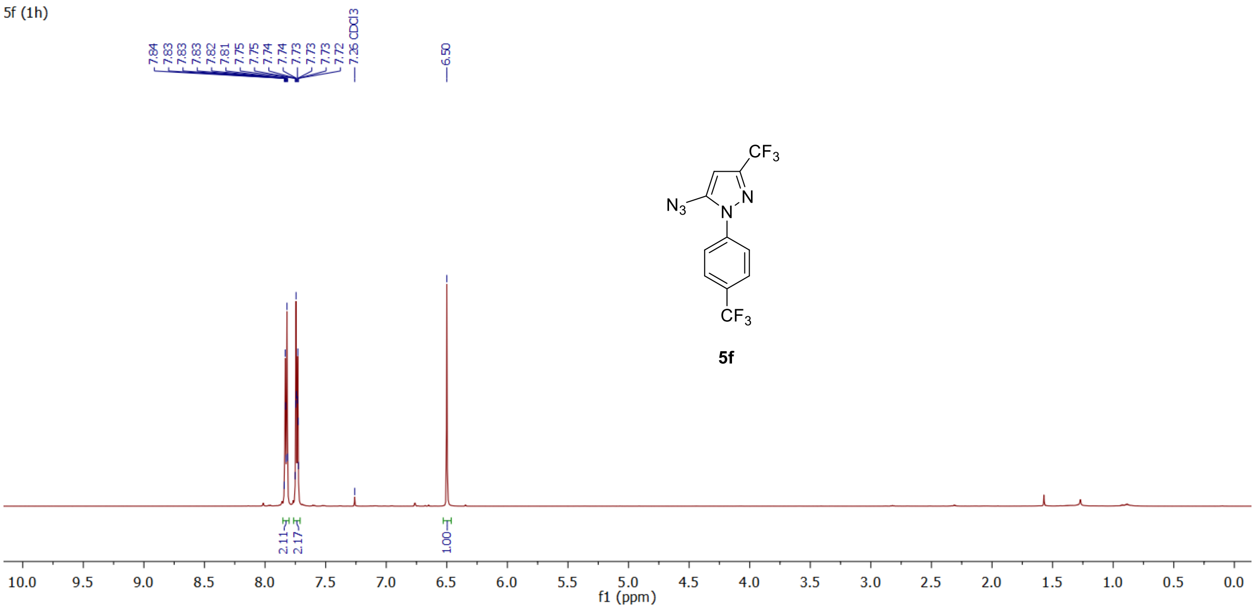

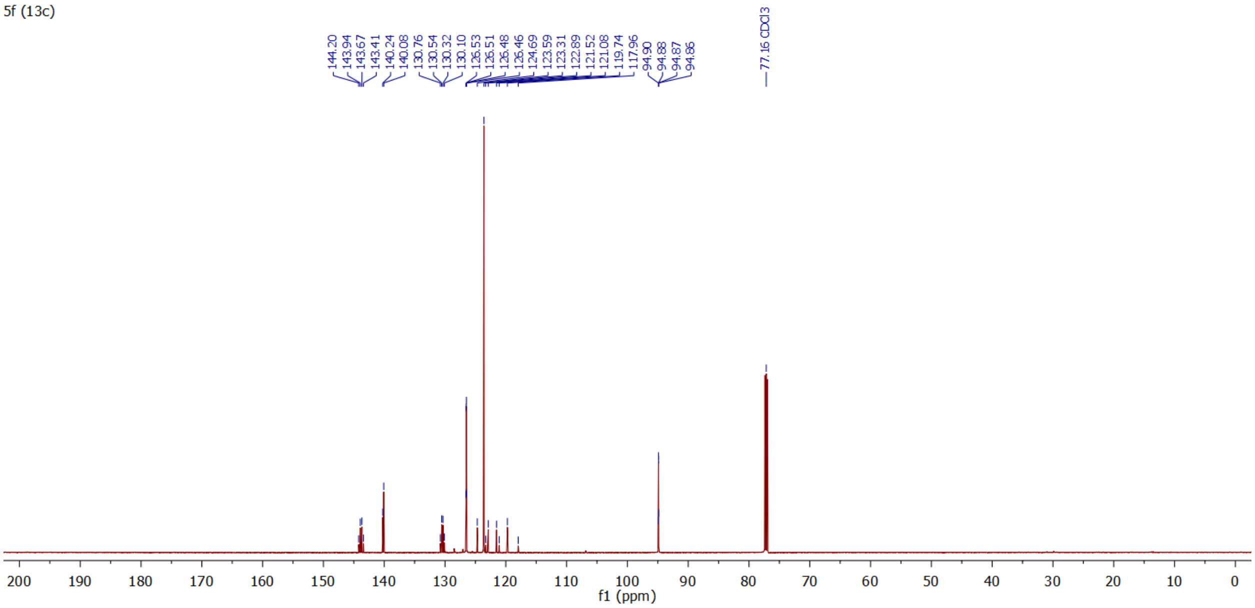

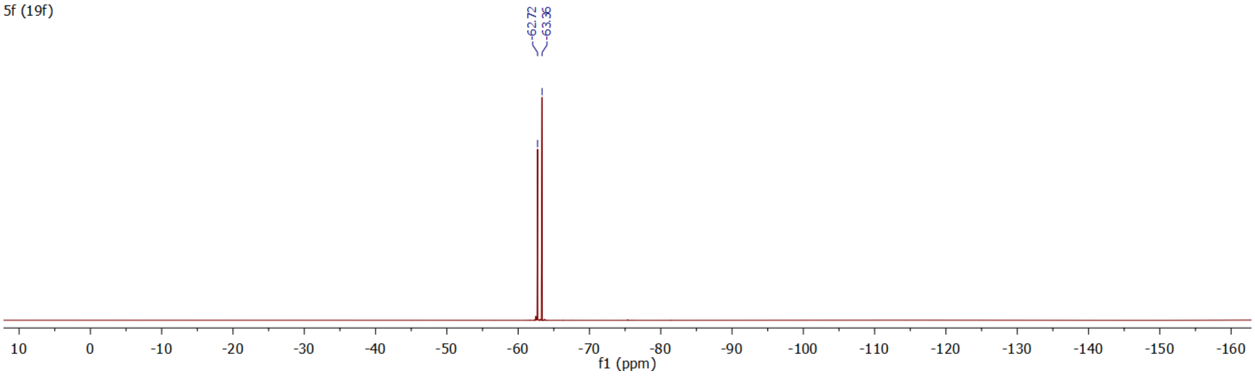

Fig S51. ^{1}H NMR (600 MHz, $CDCl_3$), $^{13}C\{^{1}H\}$ NMR (151 MHz, $CDCl_3$) and ^{19}F NMR (565 MHz, $CDCl_3$) spectra for compound **5f**.

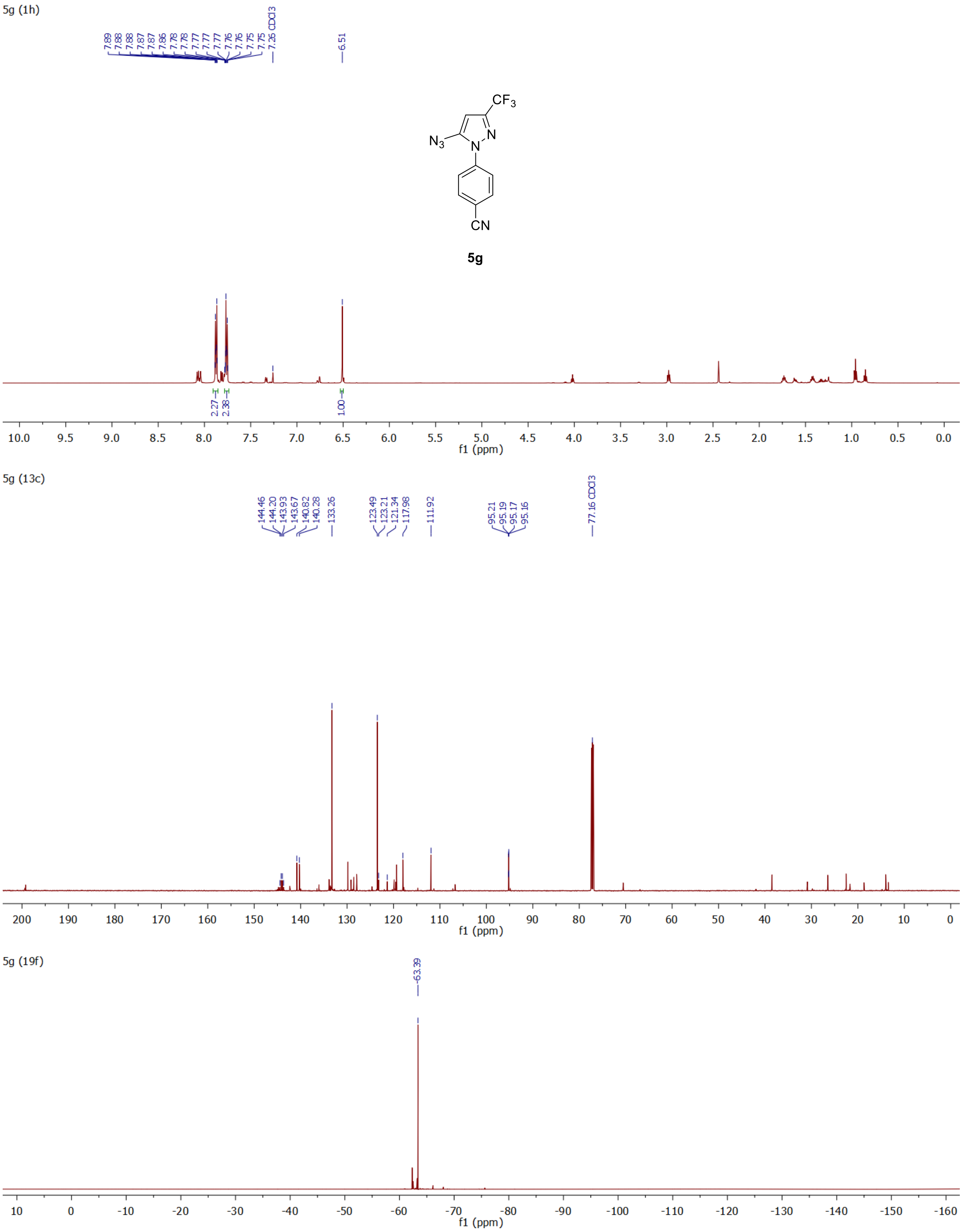

Fig S52. ^{1}H NMR (600 MHz, $CDCl_3$), $^{13}C\{^{1}H\}$ NMR (151 MHz, $CDCl_3$) and ^{19}F NMR (565 MHz, $CDCl_3$) spectra for compound **5g**.

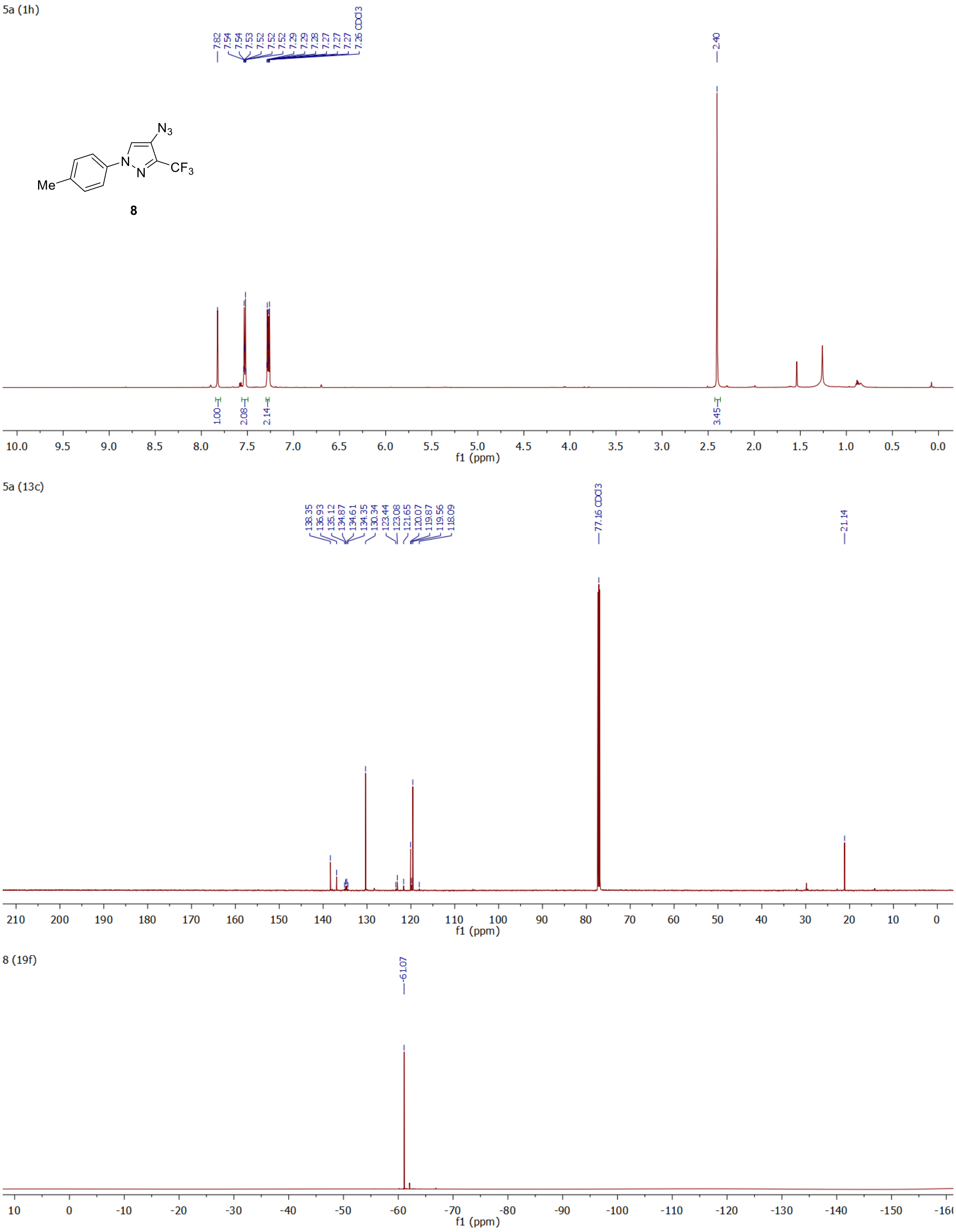

Fig S53. ^{1}H NMR (600 MHz, $CDCl_3$), $^{13}C\{^{1}H\}$ NMR (151 MHz, $CDCl_3$) and ^{19}F NMR (565 MHz, $CDCl_3$) spectra for compound **8**.

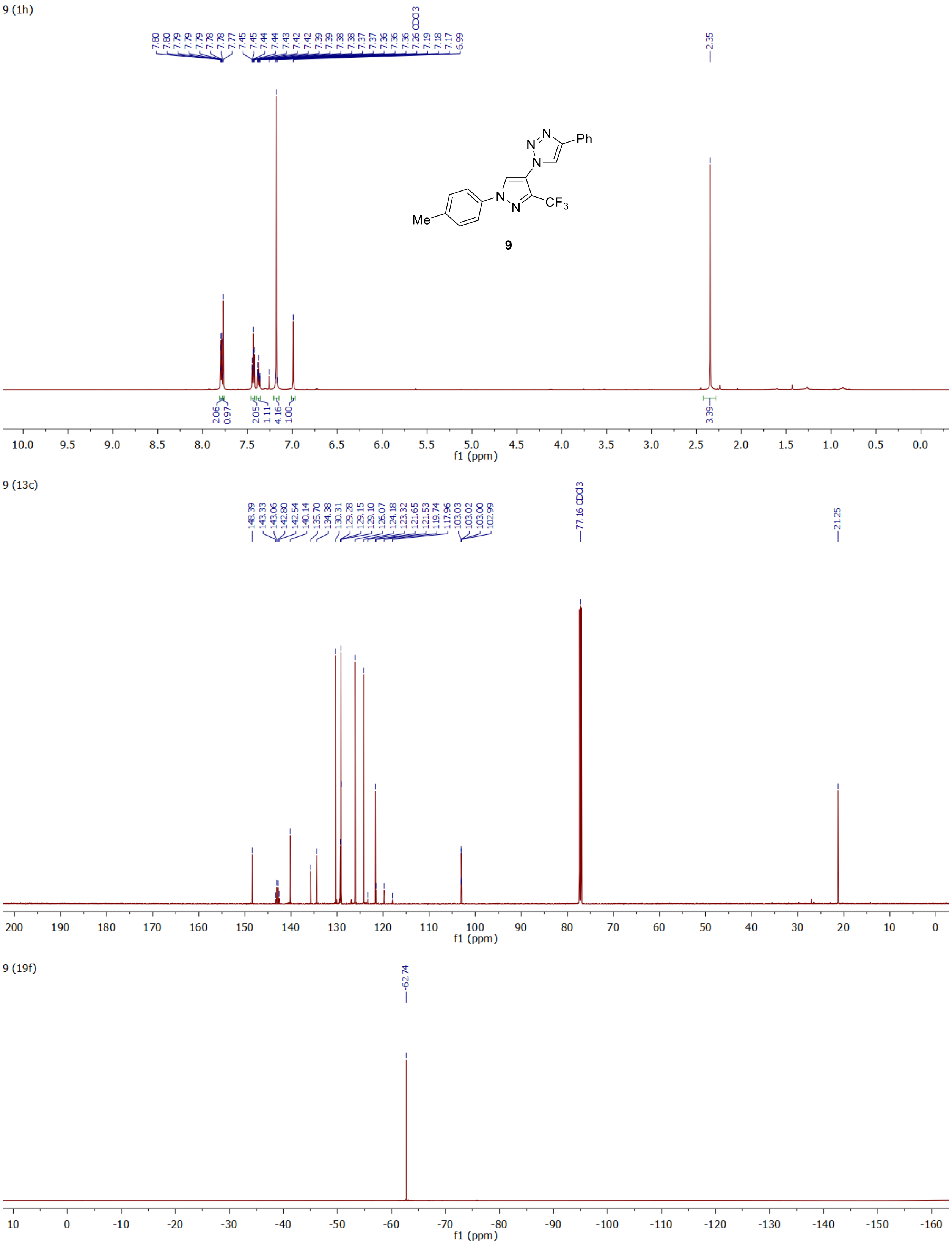

Fig S54. ^{1}H NMR (600 MHz, $CDCl_3$), $^{13}C\{^{1}H\}$ NMR (151 MHz, $CDCl_3$) and ^{19}F NMR (565 MHz, $CDCl_3$) spectra for compound **9**.

4. Crystallographic analysis

Crystallographic analysis of 1bb: Single crystals of **1bb** ($C_{20}H_{16}F_3N_5O_2$) were measured. A suitable crystal was selected and measured on a XtaLAB Synergy, Dualflex, Pilatus 300K diffractometer. The crystal was mounted in inert oil on nylon loops and kept at 100.00(10) K during data collection. Measurements for compound **1bb** were performed using mirror-focused Cu-Kα radiation. Absorption corrections were implemented on the basis of multi-scans.
Using Olex2,[6a] the structure was solved with the SHELXT[6b] structure solution program using Intrinsic Phasing and refined with the XL[6c] refinement package using Least Squares minimization. Hydrogen atoms were included using rigid methyl groups or a riding model starting from calculated positions.

Complete crystallographic data have been deposited at the Cambridge Crystallographic Data Center as supplementary publication numbers CCDC-2425718. These data can be obtained free of charge from the Cambridge Crystallographic Data Centre via https://www.ccdc.cam.ac.uk/structures/

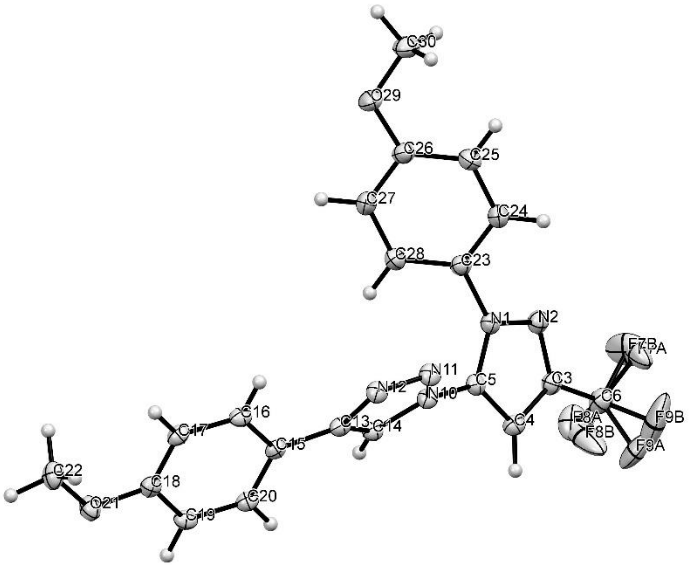

Fig S55. A view of the molecular structure of compound **1bb**. Displacement ellipsoids are drawn at the 50% probability level. X-ray data collected at the ambient temperature 100 K.

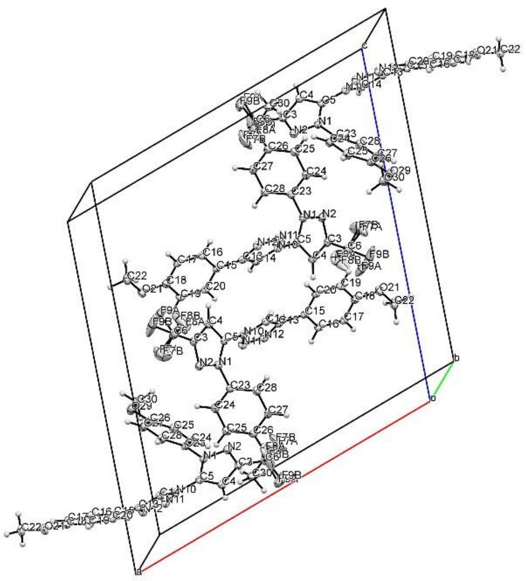

Fig S56. A view of the molecular packing in the structure of compound **1bb**.

Crystal structure determination of 1bb: Crystal Data for $C_{20}H_{16}F_3N_5O_2$ (*M* =415.38 g/mol): monoclinic, space group P21/n (no. 14), *a* = 19.3507(3) Å, *b* = 5.45900(10) Å, *c* = 19.9061(3) Å, *β* = 115.142(2)°, *V* = 1903.57(6) Å^3, *Z* = 4, *T* = 100.00(10) K, μ(Cu Kα) = 1.003 mm^{-1}, *Dcalc* = 1.449 g/cm^3, 37202 reflections measured (5.336° ≤ 2Θ ≤ 158.398°), 3961 unique (R_{int} = 0.0304, R_{sigma} = 0.0144) which were used in all calculations. The final R_1 was 0.0318 (I > 2σ(I)) and wR_2 was 0.0832 (all data).

Refinement model description
Number of restraints - 0, number of constraints - unknown.

Details:
1. Fixed Uiso
 At 1.2 times of:
  All C(H) groups
 At 1.5 times of:
  All C(H,H,H) groups
2. Others
 Sof(F9B)=Sof(F8B)=Sof(F7B)=1-FVAR(1)
 Sof(F7A)=Sof(F8A)=Sof(F9A)=FVAR(1)
3.a Aromatic/amide H refined with riding coordinates:
 C14(H14), C4(H4), C20(H20), C16(H16), C17(H17), C19(H19), C24(H24), C28(H28), C27(H27), C25(H25)
3.b Idealised Me refined as rotating group:
 C22(H22A,H22B,H22C), C30(H30A,H30B,H30C)
This report has been created with Olex2, compiled on 2024.02.14 svn.r2f8d729c for Rigaku Oxford Diffraction. Please let us know if there are any errors or if you would like to have additional features.

Table S1. Crystal data and structure refinement for **1bb**.

Identification code	ksw_260_auto
Empirical formula	$C_{20}H_{16}F_3N_5O_2$
Formula weight	415.38
Temperature/K	100.00(10)
Crystal system	monoclinic

Space group	P21/n
a/Å	19.3507(3)
b/Å	5.45900(10)
c/Å	19.9061(3)
α/°	90
β/°	115.142(2)
γ/°	90
Volume/Å^3	1903.57(6)
Z	4
ρ_{calc}g/cm^3	1.449
μ/mm^{-1}	1.003
F(000)	856.0
Crystal size/mm^3	0.5 × 0.06 × 0.06
Radiation	Cu Kα (λ = 1.54184)
2Θ range for data collection/°	5.336 to 158.398
Index ranges	-24 ≤ h ≤ 24, -6 ≤ k ≤ 6, -25 ≤ l ≤ 25
Reflections collected	37202
Independent reflections	3961 [R_{int} = 0.0304, R_{sigma} = 0.0144]
Data/restraints/parameters	3961/0/302
Goodness-of-fit on F^2	1.039
Final R indexes [I>=2σ (I)]	R_1 = 0.0318, wR_2 = 0.0810
Final R indexes [all data]	R_1 = 0.0343, wR_2 = 0.0832
Largest diff. peak/hole / e Å^{-3}	0.29/-0.20

Table S2. Fractional Atomic Coordinates (×10^4) and Equivalent Isotropic Displacement Parameters (Å^2×10^3) for **1bb**. U_{eq} is defined as 1/3 of the trace of the orthogonalised U_{IJ} tensor.

Atom	*x*	*y*	*z*	U(eq)
O(21)	8554.2(4)	4238.6(15)	6811.6(5)	25.22(19)
O(29)	4666.3(5)	9717.2(18)	8928.6(4)	31.4(2)
F(7A)	1199(3)	1417(11)	5279(4)	48.4(11)
F(8A)	1885.5(18)	-1406(5)	5148.5(16)	39.0(7)
F(9A)	1435(2)	1240(7)	4305.5(13)	47.5(8)
F(9B)	1224(6)	2230(40)	4414(7)	90(5)
F(8B)	1797(6)	-1110(20)	4905(12)	96(4)
F(7B)	1278(9)	1140(30)	5398(11)	63(3)
N(10)	4240.8(5)	5174.4(16)	5835.1(5)	17.65(19)
N(1)	3244.8(5)	4709.9(17)	6255.2(5)	20.0(2)
N(11)	4385.1(5)	7618.0(17)	5900.6(5)	21.0(2)
N(12)	5103.0(5)	7876.5(17)	6026.2(5)	20.6(2)
N(2)	2556.3(5)	3645.3(18)	6054.7(5)	22.7(2)
C(15)	6237.8(6)	5325.0(19)	6220.4(5)	17.5(2)
C(14)	4869.5(6)	3880.5(19)	5917.4(5)	17.8(2)

C(13)	5422.5(6)	5621.4(19)	6043.6(5)	17.5(2)
C(23)	3594.6(6)	6043(2)	6938.6(6)	20.5(2)
C(18)	7803.2(6)	4731(2)	6631.5(6)	19.5(2)
C(4)	3014.9(6)	2824(2)	5195.4(6)	20.5(2)
C(20)	6510.5(6)	3244.5(19)	5998.0(6)	19.3(2)
C(16)	6761.8(6)	7086.2(19)	6645.5(6)	19.3(2)
C(5)	3527.1(6)	4255.2(19)	5746.5(6)	18.2(2)
C(17)	7542.1(6)	6809(2)	6855.0(6)	20.1(2)
C(19)	7284.5(6)	2957(2)	6199.4(6)	20.4(2)
C(3)	2425.2(6)	2510(2)	5419.1(6)	21.4(2)
C(24)	3198.2(6)	7893(2)	7094.1(6)	24.7(2)
C(28)	4331.8(6)	5438(2)	7441.0(6)	25.3(2)
C(27)	4673.7(6)	6724(2)	8099.1(6)	27.1(3)
C(6)	1719.2(7)	1025(2)	5039.9(7)	28.7(3)
C(26)	4276.8(6)	8585(2)	8264.5(6)	23.9(2)
C(25)	3534.9(7)	9167(2)	7760.8(6)	26.6(2)
C(22)	9099.6(6)	6028(2)	7248.6(7)	27.7(3)
C(30)	4274.4(7)	11583(2)	9136.4(7)	30.3(3)

Table S3. Anisotropic Displacement Parameters (Å^2×10^3) for **1bb**. The Anisotropic displacement factor exponent takes the form: $-2\pi^2[h^2a^{*2}U_{11}+2hka^*b^*U_{12}+\ldots]$.

Atom	U11	U22	U33	U23	U13	U12
O(21)	19.2(4)	23.6(4)	32.1(4)	-4.4(3)	10.2(3)	-2.4(3)
O(29)	26.2(4)	41.4(5)	24.2(4)	-11.7(4)	8.3(3)	-0.2(4)
F(7A)	26.1(10)	48.8(14)	77(2)	-30.2(15)	28.1(12)	-12.3(10)
F(8A)	36.1(9)	19.6(9)	53.1(12)	-0.9(11)	10.9(8)	-9.1(6)
F(9A)	40.1(13)	65.0(15)	22.6(7)	4.8(8)	-1.0(7)	-29.3(10)
F(9B)	38(3)	133(9)	58(4)	49(5)	-18(3)	-37(4)
F(8B)	46(4)	98(6)	156(9)	-103(5)	54(5)	-33(4)
F(7B)	60(7)	78(6)	74(5)	-27(4)	50(5)	-46(5)
N(10)	19.1(4)	13.8(4)	19.2(4)	-0.4(3)	7.3(3)	-1.0(3)
N(1)	18.1(4)	21.5(5)	19.5(4)	-0.5(4)	7.2(3)	-0.4(4)
N(11)	22.1(4)	14.5(4)	24.3(4)	-0.6(3)	7.9(4)	-1.0(4)
N(12)	21.8(4)	15.6(4)	22.8(4)	-0.3(3)	7.8(4)	-0.7(4)
N(2)	18.7(4)	25.1(5)	23.0(4)	1.1(4)	7.7(4)	-1.2(4)
C(15)	20.7(5)	16.1(5)	15.4(4)	1.9(4)	7.3(4)	-1.4(4)
C(14)	20.7(5)	14.6(5)	17.9(5)	-0.3(4)	8.0(4)	0.3(4)
C(13)	21.8(5)	14.5(5)	15.4(5)	0.1(4)	7.3(4)	-0.3(4)
C(23)	21.3(5)	22.2(5)	18.4(5)	-0.5(4)	8.9(4)	-0.4(4)
C(18)	19.9(5)	20.0(5)	18.7(5)	2.5(4)	8.3(4)	-1.2(4)
C(4)	22.5(5)	18.5(5)	19.9(5)	-0.4(4)	8.4(4)	-1.4(4)
C(20)	23.2(5)	15.9(5)	18.6(5)	-1.6(4)	8.8(4)	-4.1(4)
C(16)	23.8(5)	15.3(5)	18.5(5)	-0.9(4)	8.7(4)	-0.7(4)

C(5)	19.1(5)	15.4(5)	20.0(5)	1.7(4)	8.1(4)	0.7(4)
C(17)	22.6(5)	17.3(5)	18.1(5)	-1.2(4)	6.5(4)	-4.6(4)
C(19)	25.3(5)	15.9(5)	22.2(5)	-1.4(4)	12.1(4)	-0.5(4)
C(3)	20.6(5)	20.8(5)	21.0(5)	1.9(4)	7.0(4)	-0.7(4)
C(24)	19.6(5)	29.6(6)	23.1(5)	-1.0(5)	7.4(4)	2.9(5)
C(28)	24.7(5)	27.3(6)	22.9(5)	-0.6(4)	9.2(4)	6.8(5)
C(27)	21.1(5)	35.2(7)	20.9(5)	-0.6(5)	5.2(4)	5.4(5)
C(6)	26.0(6)	34.1(7)	24.6(6)	0.6(5)	9.5(5)	-7.0(5)
C(26)	23.6(5)	29.0(6)	20.1(5)	-3.5(4)	10.3(4)	-3.0(5)
C(25)	24.6(5)	29.1(6)	27.2(6)	-4.8(5)	12.2(5)	3.3(5)
C(22)	19.7(5)	26.9(6)	33.1(6)	-1.7(5)	7.9(5)	-5.2(5)
C(30)	33.7(6)	31.6(6)	26.8(6)	-8.8(5)	13.9(5)	-2.6(5)

Table S4. Bond Lengths for **1bb**.

Atom	Atom	Length/Å	Atom	Atom	Length/Å
O(21)	C(18)	1.3673(13)	N(2)	C(3)	1.3329(14)
O(21)	C(22)	1.4300(14)	C(15)	C(13)	1.4722(14)
O(29)	C(26)	1.3604(13)	C(15)	C(20)	1.4010(15)
O(29)	C(30)	1.4318(15)	C(15)	C(16)	1.3926(15)
F(7A)	C(6)	1.301(5)	C(14)	C(13)	1.3721(15)
F(8A)	C(6)	1.361(3)	C(23)	C(24)	1.3804(16)
F(9A)	C(6)	1.331(3)	C(23)	C(28)	1.3899(15)
F(9B)	C(6)	1.374(8)	C(18)	C(17)	1.3899(15)
F(8B)	C(6)	1.220(10)	C(18)	C(19)	1.3968(15)
F(7B)	C(6)	1.325(14)	C(4)	C(5)	1.3682(15)
N(10)	N(11)	1.3578(13)	C(4)	C(3)	1.4006(15)
N(10)	C(14)	1.3553(13)	C(20)	C(19)	1.3851(15)
N(10)	C(5)	1.4082(13)	C(16)	C(17)	1.3934(15)
N(1)	N(2)	1.3490(13)	C(3)	C(6)	1.4891(16)
N(1)	C(23)	1.4338(14)	C(24)	C(25)	1.3909(16)
N(1)	C(5)	1.3623(14)	C(28)	C(27)	1.3819(16)
N(11)	N(12)	1.3104(13)	C(27)	C(26)	1.3949(16)
N(12)	C(13)	1.3714(14)	C(26)	C(25)	1.3942(16)

Table S5. Bond Angles for **1bb**.

Atom	Atom	Atom	Angle/˚	Atom	Atom	Atom	Angle/˚
C(18)	O(21)	C(22)	116.92(9)	N(1)	C(5)	N(10)	121.57(9)
C(26)	O(29)	C(30)	117.46(9)	N(1)	C(5)	C(4)	108.50(9)
N(11)	N(10)	C(5)	120.86(9)	C(4)	C(5)	N(10)	129.92(10)
C(14)	N(10)	N(11)	111.28(8)	C(18)	C(17)	C(16)	119.20(10)
C(14)	N(10)	C(5)	127.72(9)	C(20)	C(19)	C(18)	120.37(10)
N(2)	N(1)	C(23)	120.55(8)	N(2)	C(3)	C(4)	113.12(10)

N(2)	N(1)	C(5)	111.04(9)	N(2)	C(3)	C(6)	119.87(10)
C(5)	N(1)	C(23)	128.38(9)	C(4)	C(3)	C(6)	126.98(10)
N(12)	N(11)	N(10)	106.40(8)	C(23)	C(24)	C(25)	120.00(10)
N(11)	N(12)	C(13)	109.76(9)	C(27)	C(28)	C(23)	119.55(11)
C(3)	N(2)	N(1)	104.30(9)	C(28)	C(27)	C(26)	120.25(10)
C(20)	C(15)	C(13)	121.51(9)	F(7A)	C(6)	F(8A)	105.7(3)
C(16)	C(15)	C(13)	119.94(9)	F(7A)	C(6)	F(9A)	111.4(4)
C(16)	C(15)	C(20)	118.49(9)	F(7A)	C(6)	C(3)	114.2(3)
N(10)	C(14)	C(13)	104.56(9)	F(8A)	C(6)	C(3)	110.26(16)
N(12)	C(13)	C(15)	122.04(9)	F(9A)	C(6)	F(8A)	102.7(2)
N(12)	C(13)	C(14)	108.00(9)	F(9A)	C(6)	C(3)	111.74(14)
C(14)	C(13)	C(15)	129.85(10)	F(9B)	C(6)	C(3)	109.6(4)
C(24)	C(23)	N(1)	120.04(9)	F(8B)	C(6)	F(9B)	111.2(5)
C(24)	C(23)	C(28)	120.70(10)	F(8B)	C(6)	F(7B)	109.4(9)
C(28)	C(23)	N(1)	119.26(10)	F(8B)	C(6)	C(3)	117.3(5)
O(21)	C(18)	C(17)	124.53(10)	F(7B)	C(6)	F(9B)	96.0(9)
O(21)	C(18)	C(19)	115.53(10)	F(7B)	C(6)	C(3)	111.3(7)
C(17)	C(18)	C(19)	119.94(10)	O(29)	C(26)	C(27)	115.27(10)
C(5)	C(4)	C(3)	103.04(9)	O(29)	C(26)	C(25)	124.86(10)
C(19)	C(20)	C(15)	120.43(10)	C(25)	C(26)	C(27)	119.87(10)
C(15)	C(16)	C(17)	121.58(10)	C(24)	C(25)	C(26)	119.62(11)

Table S6. Torsion Angles for **1bb**.

A	B	C	D	Angle/˚	A	B	C	D	Angle/˚
O(21)	C(18)	C(17)	C(16)	-178.95(10)	C(23)	C(24)	C(25)	C(26)	0.85(18)
O(21)	C(18)	C(19)	C(20)	178.62(9)	C(23)	C(28)	C(27)	C(26)	1.19(18)
O(29)	C(26)	C(25)	C(24)	179.35(11)	C(4)	C(3)	C(6)	F(7A)	-167.3(4)
N(10)	N(11)	N(12)	C(13)	-0.10(11)	C(4)	C(3)	C(6)	F(8A)	73.9(2)
N(10)	C(14)	C(13)	N(12)	-0.45(11)	C(4)	C(3)	C(6)	F(9A)	-39.7(3)
N(10)	C(14)	C(13)	C(15)	175.69(10)	C(4)	C(3)	C(6)	F(9B)	-74.6(12)
N(1)	N(2)	C(3)	C(4)	-0.38(12)	C(4)	C(3)	C(6)	F(8B)	53.3(12)
N(1)	N(2)	C(3)	C(6)	177.55(10)	C(4)	C(3)	C(6)	F(7B)	-179.6(9)
N(1)	C(23)	C(24)	C(25)	179.29(10)	C(20)	C(15)	C(13)	N(12)	-157.72(10)
N(1)	C(23)	C(28)	C(27)	179.69(10)	C(20)	C(15)	C(13)	C(14)	26.61(16)
N(11)	N(10)	C(14)	C(13)	0.41(11)	C(20)	C(15)	C(16)	C(17)	-0.48(15)
N(11)	N(10)	C(5)	N(1)	-57.98(14)	C(16)	C(15)	C(13)	N(12)	25.10(15)
N(11)	N(10)	C(5)	C(4)	122.95(12)	C(16)	C(15)	C(13)	C(14)	-150.57(11)
N(11)	N(12)	C(13)	C(15)	-176.15(9)	C(16)	C(15)	C(20)	C(19)	0.09(15)
N(11)	N(12)	C(13)	C(14)	0.36(12)	C(5)	N(10)	N(11)	N(12)	175.70(8)
N(2)	N(1)	C(23)	C(24)	-53.27(15)	C(5)	N(10)	C(14)	C(13)	-175.14(9)
N(2)	N(1)	C(23)	C(28)	126.30(11)	C(5)	N(1)	N(2)	C(3)	0.62(12)
N(2)	N(1)	C(5)	N(10)	-179.90(9)	C(5)	N(1)	C(23)	C(24)	128.85(12)
N(2)	N(1)	C(5)	C(4)	-0.65(12)	C(5)	N(1)	C(23)	C(28)	-51.59(16)

N(2)	C(3)	C(6)	F(7A)	15.0(4)	C(5)	C(4)	C(3)	N(2)	0.00(13)
N(2)	C(3)	C(6)	F(8A)	-103.74(18)	C(5)	C(4)	C(3)	C(6)	-177.75(11)
N(2)	C(3)	C(6)	F(9A)	142.7(3)	C(17)	C(18)	C(19)	C(20)	-0.67(16)
N(2)	C(3)	C(6)	F(9B)	107.7(12)	C(19)	C(18)	C(17)	C(16)	0.28(15)
N(2)	C(3)	C(6)	F(8B)	-124.3(12)	C(3)	C(4)	C(5)	N(10)	179.54(11)
N(2)	C(3)	C(6)	F(7B)	2.8(9)	C(3)	C(4)	C(5)	N(1)	0.38(12)
C(15)	C(20)	C(19)	C(18)	0.49(16)	C(24)	C(23)	C(28)	C(27)	-0.75(18)
C(15)	C(16)	C(17)	C(18)	0.30(16)	C(28)	C(23)	C(24)	C(25)	-0.27(18)
C(14)	N(10)	N(11)	N(12)	-0.20(11)	C(28)	C(27)	C(26)	O(29)	179.60(11)
C(14)	N(10)	C(5)	N(1)	117.18(12)	C(28)	C(27)	C(26)	C(25)	-0.61(18)
C(14)	N(10)	C(5)	C(4)	-61.89(16)	C(27)	C(26)	C(25)	C(24)	-0.41(18)
C(13)	C(15)	C(20)	C(19)	-177.13(9)	C(22)	O(21)	C(18)	C(17)	-1.24(15)
C(13)	C(15)	C(16)	C(17)	176.77(9)	C(22)	O(21)	C(18)	C(19)	179.50(10)
C(23)	N(1)	N(2)	C(3)	-177.60(9)	C(30)	O(29)	C(26)	C(27)	-177.84(11)
C(23)	N(1)	C(5)	N(10)	-1.85(17)	C(30)	O(29)	C(26)	C(25)	2.39(17)
C(23)	N(1)	C(5)	C(4)	177.39(10)					

Table S7. Hydrogen Atom Coordinates (Å×10^4) and Isotropic Displacement Parameters (Å^2×10^3) for **1bb**.

Atom	*x*	*y*	*z*	U(eq)
H(14)	4916.87	2154.31	5893.12	21
H(4)	3050.55	2197.18	4766.08	25
H(20)	6162.21	2022.79	5707.27	23
H(16)	6583	8510.49	6796.17	23
H(17)	7891.39	8026.35	7147.1	24
H(19)	7463.57	1546.24	6042.68	25
H(24)	2695.41	8295.49	6745.72	30
H(28)	4598.79	4150.2	7332.82	30
H(27)	5180.96	6338.36	8440.13	32
H(25)	3260.98	10425.58	7872.22	32
H(22A)	9613.63	5488.89	7335	42
H(22B)	9071.18	6224.15	7725.61	42
H(22C)	8987.67	7597.74	6984.7	42
H(30A)	4134.91	12922.73	8775.02	45
H(30B)	4608.56	12210.43	9629.76	45
H(30C)	3811.55	10892.08	9146.67	45

Table S8. Atomic Occupancy for **1bb**.

Atom	Occupancy	Atom	Occupancy	Atom	Occupancy
F(7A)	0.699(15)	F(8A)	0.699(15)	F(9A)	0.699(15)
F(9B)	0.301(15)	F(8B)	0.301(15)	F(7B)	0.301(15)

5. References

1. G. R. Fulmer, A. J. M. Miller, N. H. Sherden, H. E. Gottlieb, A. Nudelman, B. M. Stoltz, J. E. Bercaw, K. I. Goldberg, *Organometallics* **2010**, *29*, 2176.
2. G. Mlostoń, K. Urbaniak, G. Utecht, D. Lentz, M. Jasiński, *J. Fluorine Chem.* **2016**, *192*, 147.
3. K. Świątek, G. Utecht-Jarzyńska, M. Palusiak. J.-A. Ma, M. Jasiński, *Org. Lett.* **2023**, *25*, 4462.
4. D. S. Treitler, S. Leung, *J. Org. Chem.* **2022**, *87*, 11293.
5. K. Świątek, G. Utecht-Jarzyńska, M. Jasiński, *RSC. Adv.* **2025**, *15*, 9225.
6. (a) O. V. Dolomanov, L. J. Bourhis, R. J Gildea, J. A. K. Howard, H. Puschmann, *J. Appl. Cryst.* **2009**, *42*, 339; (b) G. M. Sheldrick, *Acta Cryst.* **2015**, *C71*, 3; (c) G. M. Sheldrick, *Acta Cryst.* **2008**, *A64*, 112.
