# Supplementary figures and images for: Assembly of CF3‑Pyrazole–Triazole Hybrids through (3 + 3)-Cycloaddition/Ring Contraction and Click Chemistry
Source: J Org Chem. 2026 Feb 16;91(8):3321–8. doi: 10.1021/acs.joc.5c03120 (PMC13298894; doi:10.1021/acs.joc.5c03120)

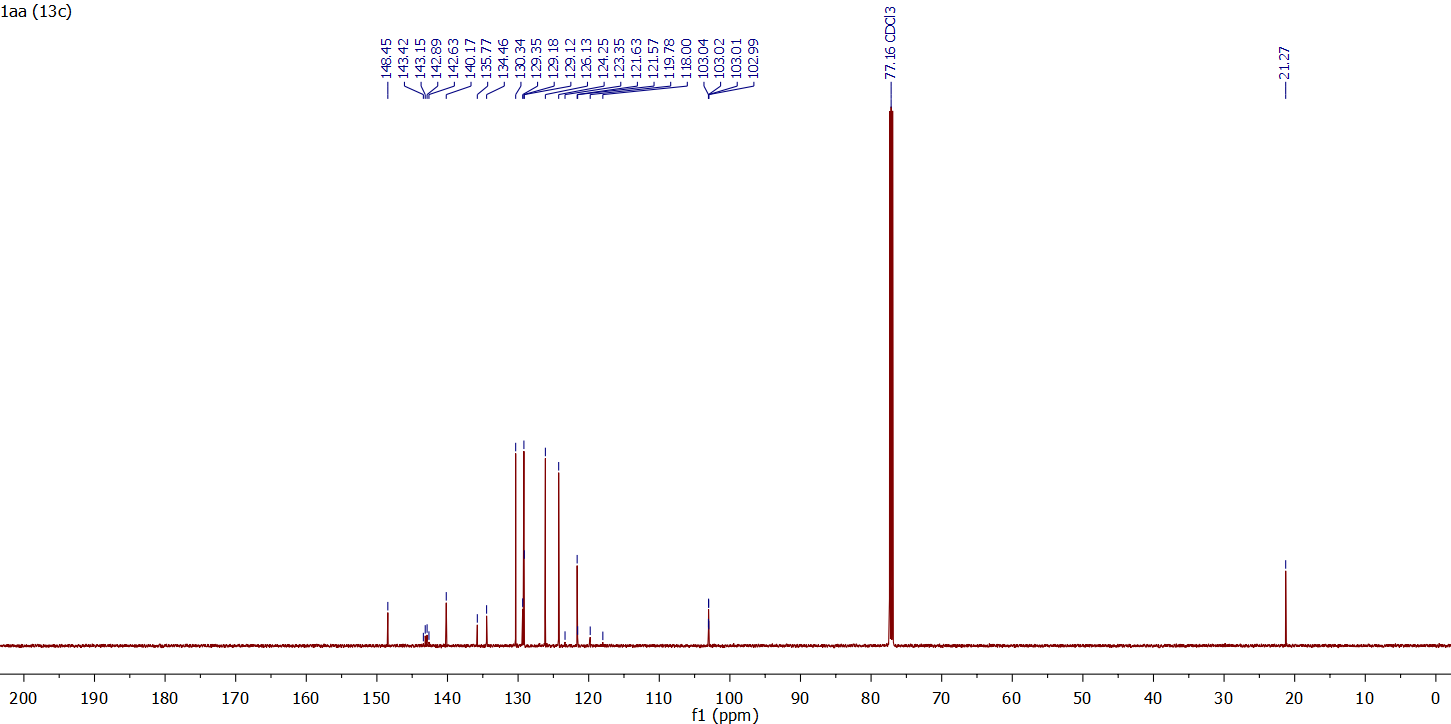

Supplement: Supplementary file 2 [file jo5c03120_si_002.zip › nmr_spectra/1aa (KSW00228B)/1aa (13c).png]

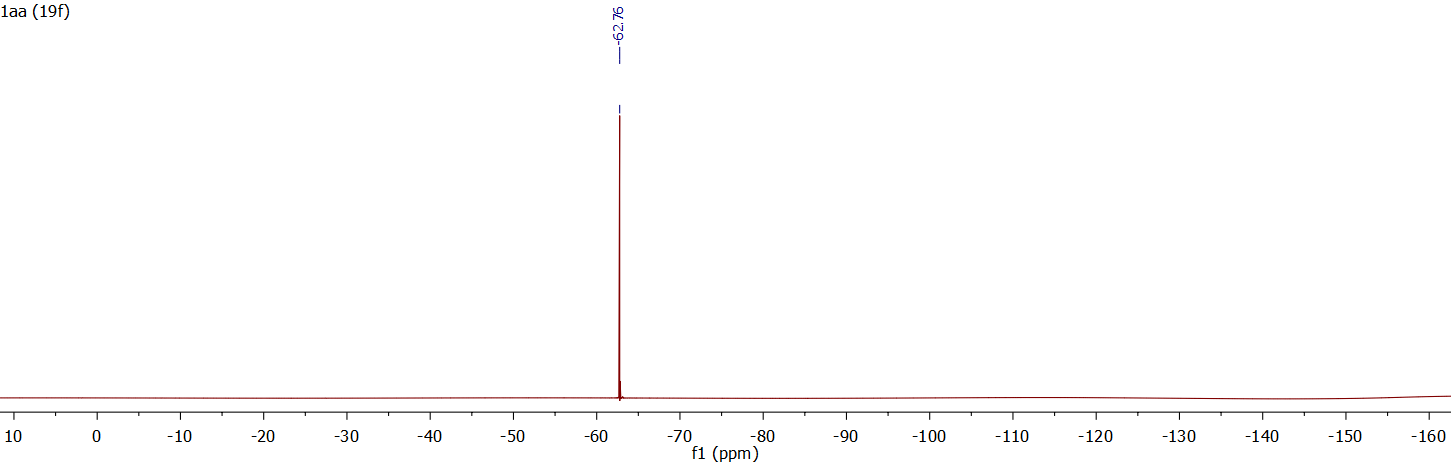

Supplement: Supplementary file 2 [file jo5c03120_si_002.zip › nmr_spectra/1aa (KSW00228B)/1aa (19f).png]

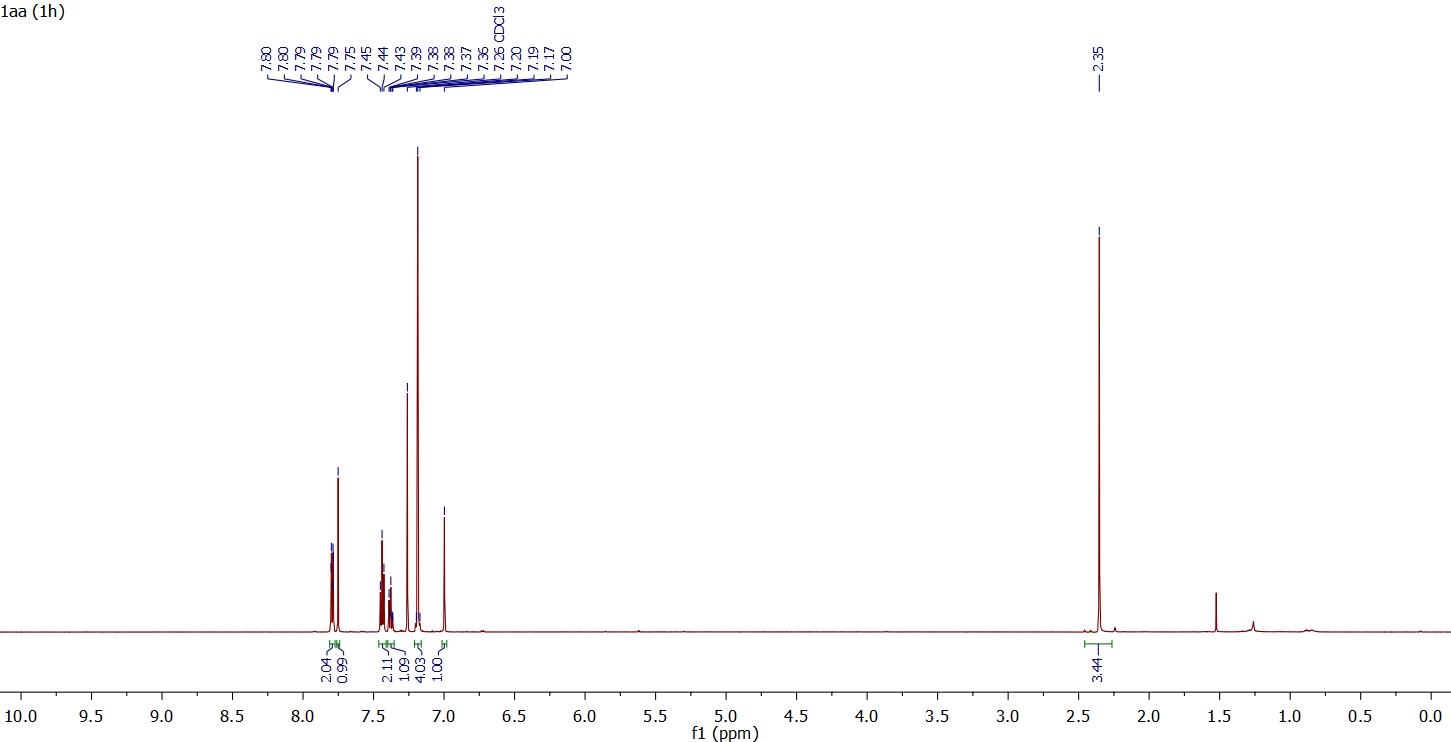

Supplement: Supplementary file 2 [file jo5c03120_si_002.zip › nmr_spectra/1aa (KSW00228B)/1aa (1h).png]

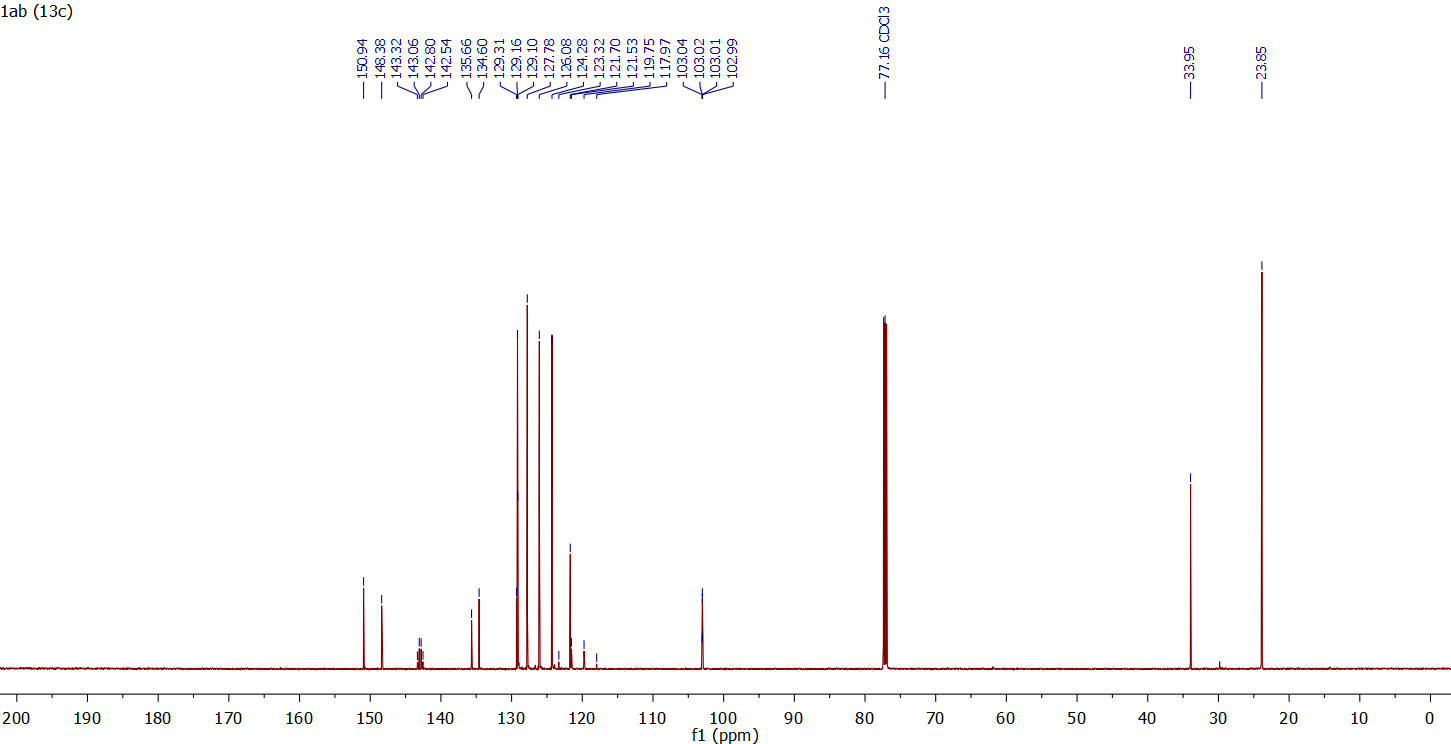

Supplement: Supplementary file 2 [file jo5c03120_si_002.zip › nmr_spectra/1ab (KSW00428B)/1ab (13c).png]

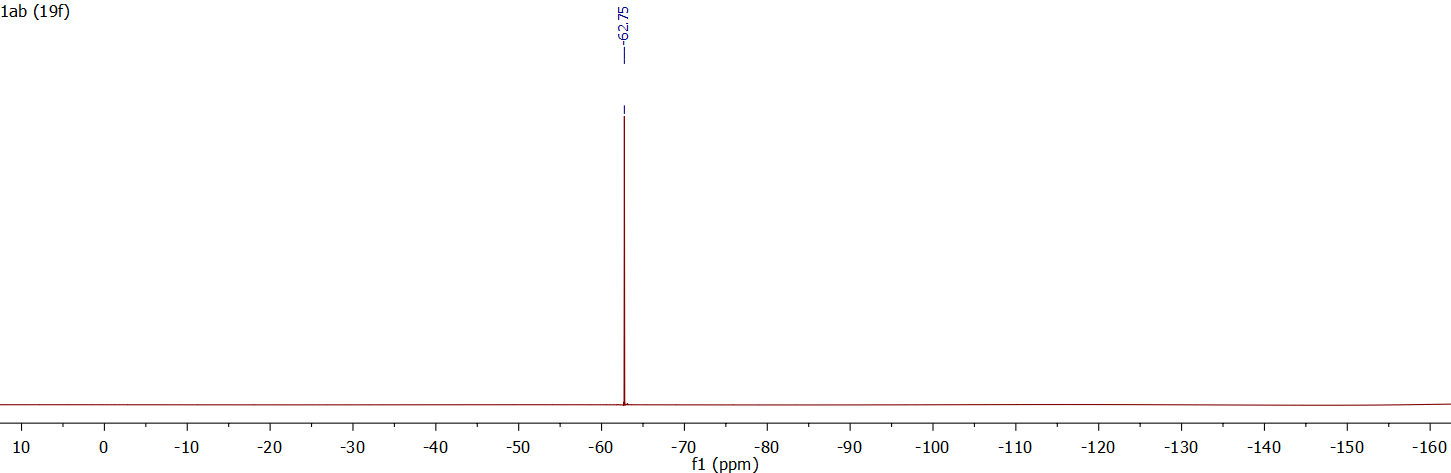

Supplement: Supplementary file 2 [file jo5c03120_si_002.zip › nmr_spectra/1ab (KSW00428B)/1ab (19f).png]

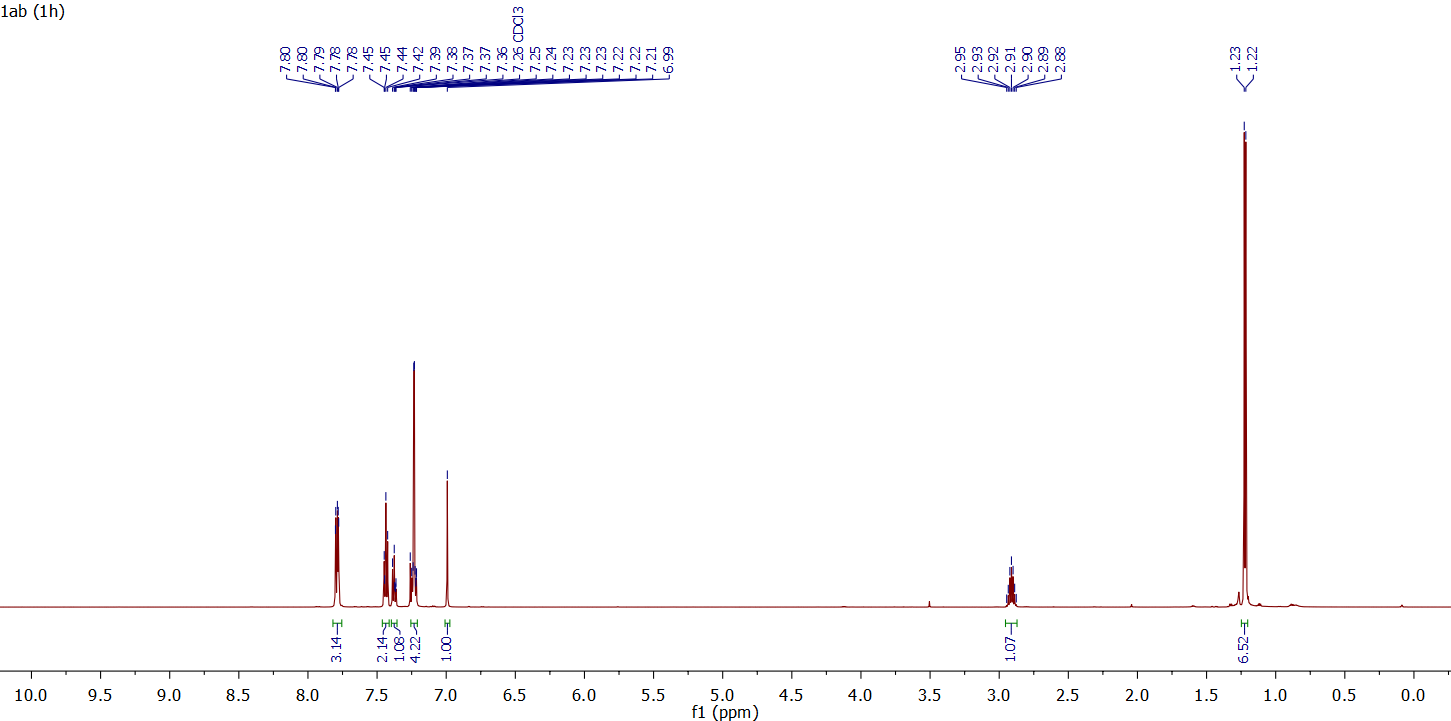

Supplement: Supplementary file 2 [file jo5c03120_si_002.zip › nmr_spectra/1ab (KSW00428B)/1ab (1h).png]
